# A Complex Network of Obesity-Risk Genes Revealed by Systematic Bioinformatics and Single-Cell Transcriptomic Analyses

**DOI:** 10.1155/jobe/7821115

**Published:** 2025-03-31

**Authors:** Yuenan Liu, Haolin Yuan, Junhui Hu, Xu Xu, Shankai Yin, Yiming Hu, Feng Liu

**Affiliations:** Department of Otolaryngology Head and Neck Surgery, Shanghai Key Laboratory of Sleep Disordered Breathing, Otolaryngological Institute of Shanghai Jiaotong University, Shanghai Jiao Tong University School of Medicine Affiliated Sixth People's Hospital, Shanghai 200233, China

**Keywords:** BMI, GWAS, obesity, PPI, single-cell RNAseq

## Abstract

The development of obesity is closely linked to genetic factors. Despite the identification of numerous genes associated with an increased risk of obesity in humans, a comprehensive understanding of their biological roles has not been achieved. In our extensive bioinformatics study, we identified 802 core genes implicated in obesity. Our protein–protein interaction (PPI) network analysis revealed that these genes form a tightly connected functional network primarily involved in neurological and metabolic regulatory processes. Moreover, our in-depth analysis of single-cell transcriptomic datasets from the human hypothalamus, pancreatic islets, adipose tissue, and liver has shed light on the distinct expression profiles of these obesity-linked genes across various tissue and cell types. This analysis also highlighted the biological processes they influence and the upstream transcriptional regulatory networks involved. Our study not only uncovers the complicated regulatory role of genetic factors in the pathogenesis and progression of obesity but also establishes a close link between the expression patterns and functional roles of these obesity-associated genes. This study provides crucial insights for advancing our understanding of the genetic mechanisms underlying obesity.

## 1. Introduction

Currently, obesity and overweight have become global public health issues, and their prevalence is continuously increasing [1]. Obesity not only causes numerous inconveniences in daily life but also serves as a significant risk factor for several noncommunicable diseases, including fatty liver disease, diabetes, cardiovascular diseases, and certain cancers [2, 3]. It also exacerbates the risk and mortality of many nonmetabolic diseases [4]. For instance, during the COVID-19 pandemic, obesity increased the risk of severe symptoms, as did the risk of hospitalization and death related to COVID-19 [5]. Unhealthy dietary habits (high consumption of energy-dense foods, transfats, and saturated fats) and unhealthy lifestyles and work habits (such as prolonged sitting, late sleeping, and lack of exercise) are the primary drivers behind the global increase in obesity rates [6–8].

However, obesity is also closely linked to the genetic variations of populations, and its development involves interactions between genetic and environmental factors [9–11]. Research in human genetics has identified specific genetic variants associated with obesity, which has greatly deepened our understanding of the biological mechanisms of obesity and facilitated the development of targeted therapies [9, 12]. This approach also contributes to the creation of disease assessment and prediction systems based on genetic risk. Studies conducted in populations over the past several years have indicated that variants in regulatory genes affecting appetite, energy balance, and the metabolism of liver and adipose tissues is significant factors contributing to human obesity [12–16]. For example, genome-wide association studies (GWASs) in populations of European descent have identified more than 700 independent variants associated with BMI involving multiple genes and pathways. These studies not only revealed genetic factors related to obesity but also explored the relationship between BMI and various diseases. Research into gene-environment interactions, such as studies from the UK Biobank, offers opportunities for a deeper understanding of the factors influencing BMI [9, 17, 18]. For instance, researchers have shown that variants in the FTO gene interact with various lifestyle risk factors, including alcohol consumption, sleep patterns, diet, and physical activity [17, 19, 20]. Moreover, mechanistic studies based on genetic research have significantly advanced the development of targeted therapeutic drugs for obesity. For instance, neurons within the hypothalamic arcuate nucleus produce the POMC precursor in response to leptin stimulation. This precursor is subsequently processed by PCSK1 into the mature *α*-melanocyte-stimulating hormone (α-MSH) polypeptide, which activates MC4 receptors (*MC4R*) on neurons within and beyond the hypothalamus. This activation suppresses appetite, enhances thermogenesis, and reduces body weight [21–23]. Leveraging this mechanism, Imcivree, an FDA-approved first-in-class targeted therapy for obesity stemming from specific genetic defects functions as an MC4R agonist [24]. It restores the impaired MC4R pathway caused by deficiencies in upstream genes such as *POMC*, *PCSK1*, or *LEPR*. Consequently, it reduces hunger, promotes metabolism, and aids in weight reduction for patients suffering from MC4R pathway deficiencies.

Despite the identification of numerous human obesity-related risk genes and the validation of the biological functions of some of these genes, especially their core regulatory roles in metabolism in animal models, many questions remain about the genetic mechanisms of human obesity [25]. For example, the biological functions of many risk genes and their direct associations with disease onset have not been determined. The mechanisms by which genes interact with diet, the environment, and lifestyle to influence disease development are also not well understood. Moreover, since obesity is a disease involving multiple systems, not only the core metabolic organs, such as adipose tissue and the liver but also nonmetabolic organs and tissues, such as the intestines, pancreas, muscles, nerves, and immune systems, are involved in the development of obesity [26, 27].

Due to the lack of precise expression information on genes in all these organs, the exact sites where these genes exert their effects are largely unknown. This also raises a broader question: is there a collaboration and association in the expression modes and biological functions of obesity-related risk genes and are there core molecular pathways or networks that influence the onset of obesity? Therefore, conducting systematic bioinformatics network analysis of obesity-risk genes is highly necessary.

## 2. Methods and Materials

### 2.1. Identification of Key Risk Genes for Obesity

In the GWAS catalog of European Bioinformatics Institute, we retrieved all genetic variation loci associated with obesity by conducting a keyword search for “body mass index (BMI)”. By setting a more stringent *p* value cutoff of 5 × 10^−11^, we retained genetic variants that were significantly associated with obesity. In further identifying linked risk genes at these loci, we adhered to the following principles: (1) the gene must be identified in at least two studies, (2) for single nucleotide polymorphisms (SNP) in intergenic sequences, we retained genes supported by Expression Quantitative Trait Loci (eQTL) data and model validation results, and (3) in cases where linked genes included both coding genes and noncoding RNAs, we prioritized the coding genes (Supporting Figure 1).

### 2.2. Protein–Protein Interaction (PPI) Network and Enrichment Analysis

We conducted PPI network analysis using the online *string* tool. Additionally, we carried out enrichment analyses for Gene Ontology (GO), Kyoto Encyclopedia of Genes and Genomes (KEGG) pathway, and WIKI pathway enrichment. Complementary biological function enrichment analyses were performed using the *ClueGO* plugin in *Cytoscape* software and the *clusterProfiler* package in *R*. Transcriptional regulatory network analysis was also conducted using the *iRegulon* plugin in *Cytoscap*e.

### 2.3. Single-Cell Expression Profiling Analysis

Datasets for human pancreatic islets (GSE251912), liver (GSE125688), and adipose tissue (GSE236708) were obtained from the GEO database. The human peripheral blood mononuclear cells (PBMCs) data were provided by 10X Genomics (https://cf.10xgenomics.com/samples/cell/pbmc3k/pbmc3k_filtered_gene_bc_matrices.tar.gz). The human hypothalamus data were obtained from the Human Brain Cell Atlas v1.0. The single-cell matrix of UMI counts of genes from these datasets was processed using the *Seurat* (v4.0.0) pipeline [28] in *R* (v4.0.5).

For clustering and cell type annotation, we selected a resolution of 0.5. Clustering was performed using graph-based methods and visualized using t-distributed stochastic neighbor embedding (*t*-SNE) or uniform manifold approximation and projection (UMAP) with the *RunTSNE* and *RunUMAP* functions from the *Seurat* package in *R*. Specific markers for each cluster were identified using the *FindMarkers* function. To unbiasedly determine cell types in the filtered datasets, we employed *SingleR* (v1.4.1), a computational framework that references bulk transcriptomes for cell type annotation. The *Human Primary Cell Atlas* (HPCA) was used as reference datasets [29].

### 2.4. Statistics

In the functional enrichment analysis, an False Discovery Rate (FDR) of less than 0.05 was considered to be statistically significant.

## 3. Results

**Figure 1:**
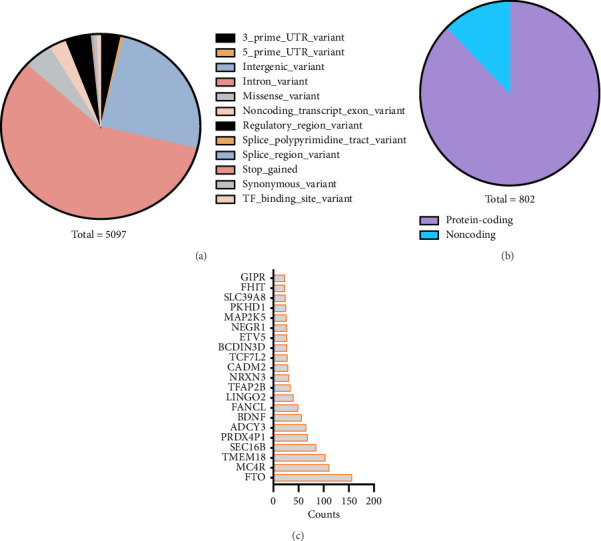
Identification of key risk genes for obesity. (a) Classification of obesity-associated SNPs at a *p* value cutoff of 5 × 10^−11^. (b) Summary of obesity-risk genes. (c) The top 20 most frequently identified genes.

In our study, we aimed to identify the key genetic loci associated with the risk of obesity. Utilizing the GWAS catalog database, we began by extracting all SNP loci linked to BMI, which is the gold standard of obesity. This initial search yielded a significant number of loci, with the most statistically significant having a *p* value of 1 × 10^−5^. To refine these results, we applied a stricter significance threshold, which reduced our list to 5097 SNP loci (Supporting Table 1). Notably, the majority of these loci were located in intronic or intergenic regions (Figure 1(a)). Our subsequent more rigorous selection methods helped us identify a core set of genes associated with the risk of obesity. Among these genes, 703 were found to encode proteins, while 99 were linked to long noncoding RNAs (lncRNAs), microRNAs, and readthroughs (Figure 1(b) and Supporting Table 2). The biological functions of several of these genes, including *FTO*, *MC4R*, and *TMEM18* (Figure 1(c)), related to obesity have been widely validated [17, 30–32].

**Figure 2:**
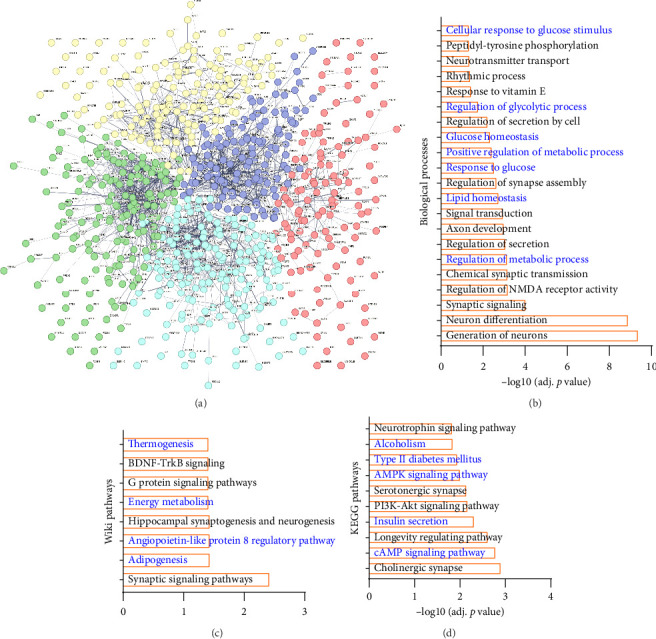
Protein–protein interaction network of obesity-risk genes. (a) PPI network of obesity-risk genes determined by String analysis. (b–d) Enriched biological processes (b), Wiki pathways (c), and KEGG pathways (d) of obesity-risk genes identified by String analysis.

In an effort to deepen our understanding of the biological functions linked to BMI risk genes, we applied String's network analysis to construct a PPI network. Subsequent functional enrichment analysis revealed the involvement of 721 proteins within this network (Figure 2(a)). These proteins demonstrated a significant functional relationship, as evidenced by enrichment analysis. Our GO analysis identified two primary categories of biological processes (BPs) associated with these key BMI risk genes. The first category pertains to neurological processes and encompasses areas such as “synaptic signaling,” “neuronal differentiation,” “generation of neurons,” and “axon development.” The second category was closely related to metabolic signaling pathways and included “lipid homeostasis”, “response to glucose,” and “regulation of glycolytic processes” (Figure 2(b)). Additionally, analyses using KEGG and WikiPathways similarly indicated that the core BMI risk genes are predominantly involved in pathways related to neural regulation and metabolic regulation. For instance, in neural regulation, there are KEGG pathways such as the cholinergic synapse, serotonergic synapse, and neurotrophin signaling pathways, along with WikiPathways such as the synaptic signaling pathways and hippocampal synaptogenesis and neurogenesis (Figure 2(c)). In terms of metabolic regulation, there are KEGG pathways related to insulin secretion, the cAMP signaling pathway, the AMPK signaling pathway, and Type 2 diabetes mellitus, as well as WikiPathways such as adipogenesis, energy metabolism, and the ANGPTL8 regulatory pathway (Figure 2(c)). Taken together, our enrichment analysis underscores the likely significant roles of neural and metabolic regulatory mechanisms in the development of obesity. These findings align with previous findings, suggesting that the onset of obesity is closely associated with the regulation of appetite, feeding, and body temperature by the central nervous system, as well as the regulation of metabolic balance by peripheral metabolic organs.

### 3.1. Subclustering Analysis of Obesity-Related Risk Genes

**Figure 3:**
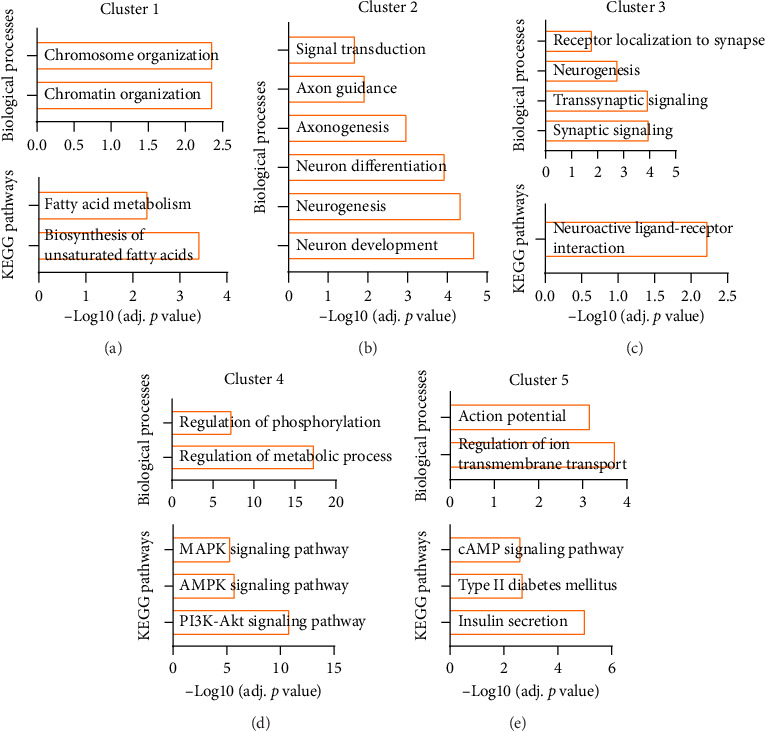
Subclustering of the network of obesity-risk genes. (a–e) Enriched biological processes or KEGG pathways of subgroups of obesity risk genes.

Further clustering analysis revealed that these obesity-risk genes were distributed across five subgroups. Subgroup 1 consisted of 121 proteins, namely, AFF, JMJD1C, and KDM4C, which were primarily associated with BPs such as “chromatin organization” and “chromosome organization,” as well as KEGG pathways such as “biosynthesis of unsaturated fatty acids” and “fatty acid metabolism” (Figure 3(a) and Supporting Figure 2a). Subgroup 2 included 142 proteins, namely, APOE, HMGB1, and ARG1, which were related mainly to BPs such as “neuronal development” and “axonogenesis” (Figure 3(b) and Supporting Figure 2b). Subgroup 3, containing 157 proteins, namely, NRXN1, MC4R, and BDNF, was primarily associated with processes such as “synaptic signaling” and “*trans*synaptic signaling,” as well as the “neuroactive ligand–receptor interaction pathway” (Figure 3(c) and Supporting Figure 2c). Subgroup 4 included 162 proteins, such as TRAF3, AKT1, and IGF1R; these proteins were significantly related to BPs such as “Regulation of metabolic process” and “Regulation of phosphorylation;” and pathways such as the “PI3K-Akt signaling pathway,” “AMPK signaling pathway,” and “MAPK signaling pathway” (Figure 3(d) and Supporting Figure 2d). Finally, Subgroup 5, composed of 168 proteins, which included HCN4, CACNA1C, and KCNQ1, was associated mainly with processes such as “Regulation of ion transmembrane transport” and “action potential,” as well as pathways such as “Insulin secretion,” “Type II diabetes mellitus,” and the “cAMP signaling pathway” (Figure 3(e) and Supporting Figure 2e).

### 3.2. Single-Cell Analysis of the Expression of Obesity-Risk Genes in Human Tissues

**Figure 4:**
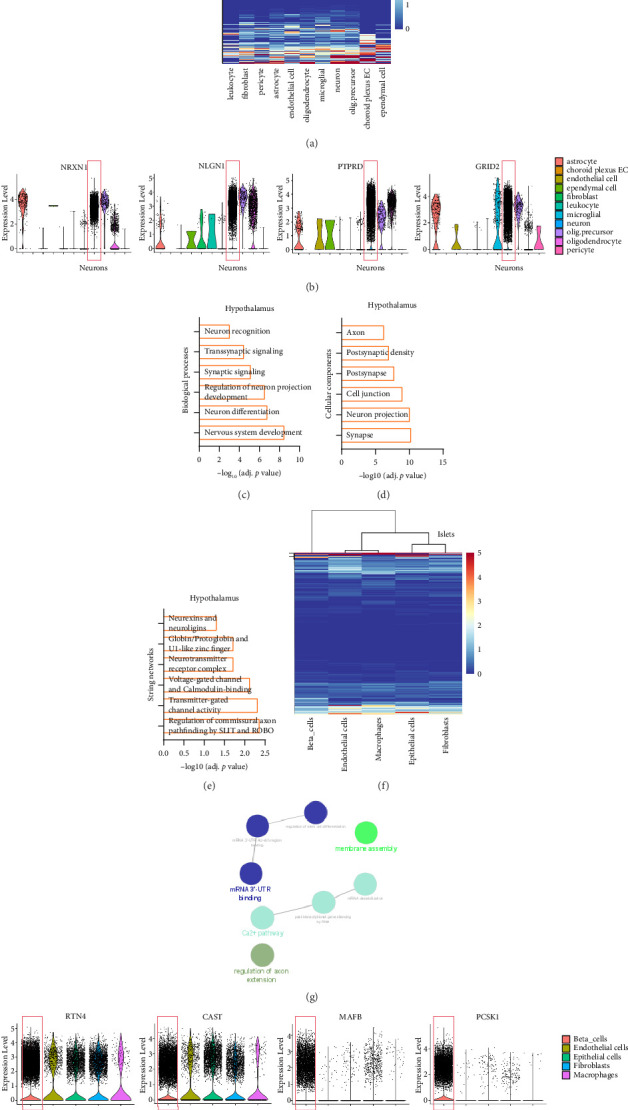
Single-cell expression analysis of obesity-risk genes in the hypothalamus and islets. (a) Heatmap showing the average expression of obesity-risk genes in hypothalamic cell clusters. (b) Violin plot showing selected obesity-risk genes that are highly expressed in hypothalamic neurons. (c–e) Enriched biological processes (c), cellular components (d), and String network (e) of obesity-risk genes that are highly expressed in hypothalamus neurons. (f) Heatmap showing the average expression of obesity-risk genes in islet cell clusters. (g) Violin plot showing selected obesity-risk genes that are highly expressed in islet β-cells. (h) ClueGO analysis of enriched genes related to the risk of obesity that is highly expressed in islet β-cells.

A current understanding of the pathogenesis of BMI suggests the involvement of the central nervous system in influencing the appetite system, the regulation of glucose metabolism by the pancreas, and the metabolism of glucose and fats by the liver and adipose tissue [26, 27]. These findings align with the enrichment of biological functions in our cellular network analysis. To further explore whether there was coexpression among the core genes associated with BMI, we utilized published single-cell sequencing data from the human hypothalamus [33], liver (GSE125688) [34], pancreatic islets (GSE251913), and adipose tissue (GSE236708) to calculate the expression levels of these core genes in different tissues at the single-cell level. Our analysis revealed that 227 genes (Figure 4(a)), including *NRXN1*, *NLGN1*, *PTPRD*, and *GRID2*, were highly expressed in neurons (Figure 4(b)). GO analysis indicated that these genes are located primarily at synapses and neurons and are related to BPs such as “neuronal differentiation” and “synaptic signaling” (Figure 4(c)). STRING network analysis revealed that the protein networks of these genes included “transmitter-gated channel activity” and “voltage gated channel and calmodulin binding” (Figure 4(d)). Furthermore, 103 genes (Figure 4(e)), including *RTN4*, *CAST*, *MAFB*, and *PCSK1*, were expressed in the β-cells of pancreatic islets (Figure 4(f)). *MAFB* and *PCSK1* are widely confirmed to be significantly related to the secretion of insulin. Enrichment analysis using ClueGO showed that these genes, which are highly expressed in β-cells, are functionally related to BPs such as the “Ca^2+^ pathway,” “membrane assembly,” and “mRNA 3′UTR binding” (Figure 4(g)).

**Figure 5:**
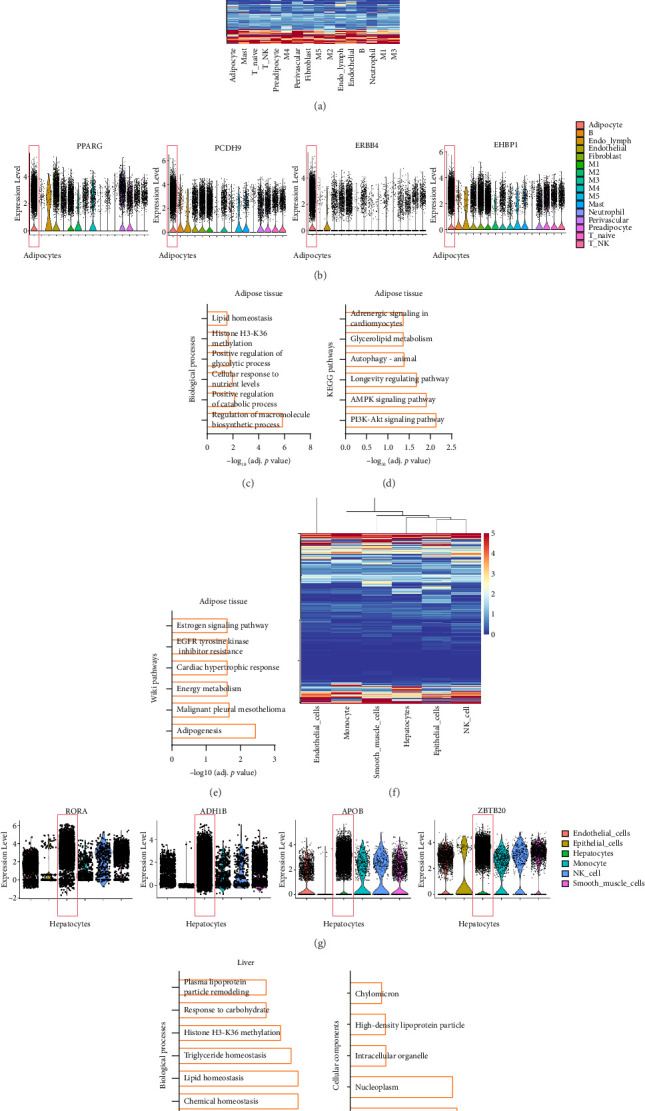
Single-cell expression analysis of obesity-risk genes in adipose and liver tissues. (a) Heatmap showing the average expression of obesity-risk genes in adipose cell clusters. (b) Violin plot showing selected obesity-risk genes that are highly expressed in adipocytes. (c–e) Enriched biological processes (c), KEGG pathways (d), and WIKI pathways (e) of obesity-risk genes that are highly expressed in adipocytes. (f) Heatmap showing the average expression of obesity-risk genes in liver cell clusters. (g) Violin plot showing selected obesity-risk genes that are highly expressed in hepatocytes. (h, i) Enriched biological processes (h) and cellular components (i) of obesity-risk genes that are highly expressed in hepatocytes.

Additionally, 178 genes (Figure 5(a)), including *PPARG*, *PCDH9*, *ERBB4*, and *EHBP1*, were expressed in adipocytes in adipose tissue (Figure 5(b)). The gene function enrichment analysis of these genes revealed that they are associated mainly with BPs such as “lipid homeostasis” and “positive regulation of catabolic process” (Figures 5(c) and 5(d)), and KEGG pathway enrichment analysis revealed significant associations with “glycerolipid metabolism,” the “AMPK signaling pathway,” and the “PI3K-AKT signaling pathway” (Figure 5(e)). Overall, 171 genes (Figure 5(f)), including *RORA*, *ADH1B*, *APOB*, and *ZBTB20* (Figure 5(g)), were highly expressed in hepatocytes and were found to be localized mainly to the “nuclear lumen” and “high-density lipoprotein particle” (Figure 5(h)), closely related to BPs such as “lipid homeostasis,” “triglyceride homeostasis,” and “plasma lipoprotein particle remodeling” (Figure 5(i)).

To verify whether obesity-risk genes exhibit tissue-specific expression, we selected a publicly available single-cell transcriptome sequencing dataset of human PBMCs provided by 10X Genomics as nonobese controls(10X Genomics, pbmc.3k dataset). The results showed that, with the exception of a few genes such as *LYZ*, *HMGB1*, *HLA-DRB1*, and *CD47*, most obesity-risk genes were either not expressed or expressed at very low levels in PBMCs (Supporting Figure 3). This suggests that obesity-risk genes indeed exhibit characteristic expression in obese tissues. In summary, this comprehensive analysis underscores the complex interplay of genetic expression across various tissues in the context of obesity, highlighting the multifaceted nature of its pathogenesis.

### 3.3. Transcriptional Regulatory Network Analysis of Obesity-Risk Genes

**Figure 6:**
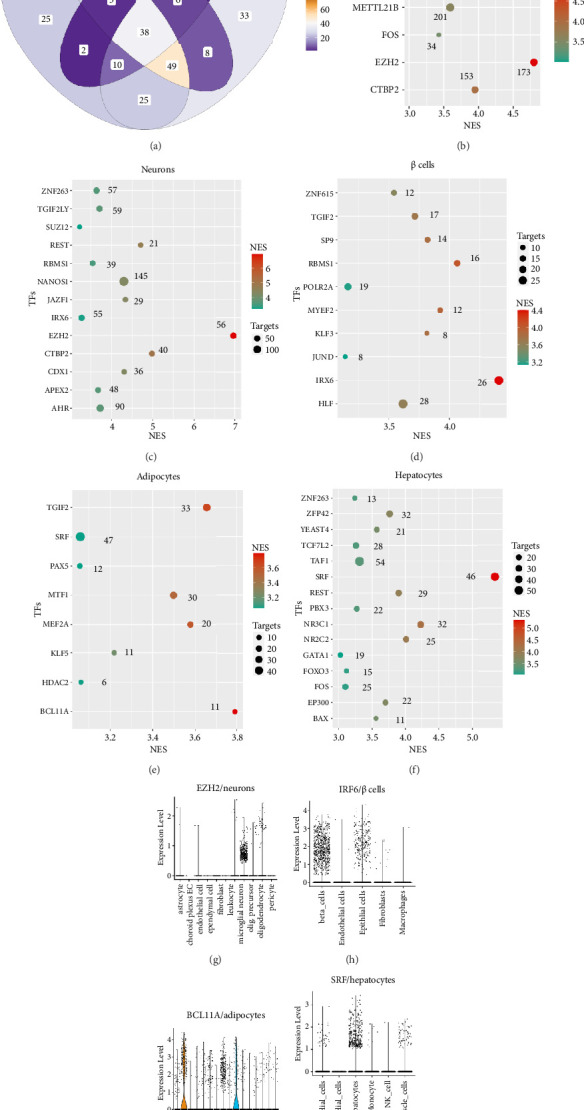
Transcriptional regulatory network of obesity-risk genes. (a) Venn plot showing the common genes expressed in neurons, β-cells, adipocytes, and hepatocytes. (b) Enriched regulons of obesity risk genes. (c–f) Enriched regulons of obesity risk genes highly expressed in neurons, β-cells, adipocytes, and hepatocytes. (g–j) Violin plot showing the expression of the leading transcription factors in neurons, β-cells, adipocytes, and hepatocytes.

A comparative analysis of obesity-risk genes in key cells of different tissues revealed that cells associated with obesity exhibit unique expression profiles and the expression of several genes. Notably, 38 genes were expressed in all four cell types, suggesting that these genes might influence obesity through different biological pathways (Figure 6(a) and Supporting Figure 4). We further focused on whether these obesity-risk genes share common transcriptional regulatory mechanisms. Through iRegulon analysis, we found that these genes are regulated by 9 transcription factors overall, including *EZH2*, *SUZ12*, and *CTBP2* (Figure 6(b)). Importantly, 173 genes were regulated by *EZH2*, and 304 genes were regulated by *MTF1* (Figure 6(b)). Further iRegulon analysis of the genes expressed in cells from different tissues revealed significant enrichment of 13 transcription factors, including *EZH2* and *CTBP2*, in neurons of the hypothalamus (Figure 6(c)); 10 transcription factors, including *IRX6* and *RBMS1*, in pancreatic β-cells (Figure 6(d)); 15 transcription factors, including *SRF* and *NR3C1*, in hepatocytes (Figure 6(e)); and 8 transcription factors, including *BCL11A* and *GTF2*, in adipocytes (Figure 6(f)). Moreover, analysis of single-cell sequencing data from the four tissues revealed specific expression of *EZH2*, *IRX6*, *BCL11A*, and *SRF* in neurons of the hypothalamus, pancreatic β-cells, adipocytes, and hepatocytes (Figures 6(g), 6(h), 6(i), 6(j)), respectively, further validating the existence of these cell-specific transcriptional regulatory networks. In summary, regulon analysis highlights the intricate cell-specific transcriptional regulatory networks governing obesity-risk genes across various tissue types, offering insights into the complex genetic underpinnings of obesity.

## 4. Discussion

The occurrence of obesity is closely regulated by genetic factors [35–37]. In recent years, a substantial number of obesity-related genetic risk genes have been identified, yet the functions of these genes have largely not been explored [36]. Whether these genes are evenly distributed across various BPs or concentrated in certain key biological pathways requires extensive research. In our study, we screened the obesity-related risk loci identified in previous GWASs and identified 802 core obesity-risk genes. Analysis of PPI networks and functional enrichment revealed that these core genes are part of an interconnected protein network that is primarily associated with functions related to neural and metabolic regulation. Furthermore, using single-cell transcriptomic sequencing datasets from various human organs, we characterized the single-cell expression patterns of these core obesity-risk genes, established a link between their expression and function, and identified their regulation by a series of transcription factors, including *EZH2*.

Our enrichment analysis indicated that obesity-related risk genes are highly correlated with neurological BPs, including axon growth and synaptic transmission [38], as well as BPs, such as insulin secretion and glycolipid metabolic balance [36, 39]. This aligns with the current understanding that the onset of obesity involves multiple organs and levels, including the central nervous system's regulation of appetite, body temperature, and basal metabolism, as well as hormonal changes produced by the endocrine system (such as insulin and glucagon) and alterations in glucose and lipid metabolism in the liver and adipose tissue. Recent studies have increasingly focused on the abnormal regulation of eating behavior by the hypothalamus, which may be a key factor in the development of various metabolic syndromes, including obesity [25, 40]. Moreover, some genes previously thought to function in peripheral metabolic organs actually exert their effects in the hypothalamus [41]. A significant mechanism of action for GLP-1 agonists, which were recently used to treat Type 2 diabetes and obesity, is through the regulation of appetite [42]. We found that a large number of obesity-risk genes are expressed in hypothalamic neurons, providing valuable insight into the molecular mechanisms regulating eating behavior.

Given the difficulties that obese individuals face in maintaining restrict diet and consistent exercise, pharmacotherapy remains a potentially optimal and primary method for obesity treatment. Human genetic research plays a crucial role in the development of antiobesity medications, as risk genes identified through genetic studies and their associated molecular pathways often serve as potential drug targets. Furthermore, individual genetic differences necessitate personalized approaches in drug therapy. Our network analysis of obesity-risk genes suggests that many of these genes (at least a subset) exhibit high druggability. Indeed, some of these genes and their mediated BPs have already become targets for effective weight-loss medications. For instance, metformin targets AMPK to increase the energy expenditure, thereby alleviating Type 2 diabetes while also improving obesity and NALFD [43, 44]. Imcivree, LB-54640, and PL-8905 target MC4R leading to appetite suppression and weight reduction [24, 45–47]. Additionally, tesofensine acts on monoamine neurotransmitter transporters, effectively inhibiting the reuptake of norepinephrine, serotonin, and dopamine in the synaptic cleft, thereby enhancing POMC neuron activity to suppress appetite and reduce weight [48, 49]. Beside of these well-accepted drug targets, our analysis suggested that, NRXN1, which is highly expressed in hypothalamic neurons and resides in the same PPI cluster as MC4R, is closely related to synapse formation and signal transduction functions [50, 51]. Drug development targeting NRXN1 could potentially enhance the synaptic strength mediated by NRXN1, thereby working synergistically with hypothalamic POMC-targeting antiobesity medications to regulate appetite. However, extensive studies using cellular and animal models are still needed to support the application of these potential target genes in the development of obesity drugs. Additionally, our analysis also highlights potential side effects of obesity-targeted drugs. Since these genes are widely expressed in various types of neurons, it is essential to consider their potential impacts on sleep, cognition, alertness, and mood.

This study has several limitations. First, concerning the risk loci, there is a lack of systematic meta-analyses assessing the true effect of the included loci on obesity [52]. Second, in our selection of risk genes, we retained only those genes with protein-coding functions for interaction network analysis, temporarily overlooking noncoding genes. Noncoding RNAs, which exert biological regulatory effects through interactions with proteins, are an important part of the genetic mechanism of obesity [53]. Furthermore, we retained risk genes that were cross-validated in at least two studies, while genes reported only once were temporarily ignored, potentially leading to a significant false-negative result. Finally, we used only the traditional String tool to analyze the PPI network, neglecting other potential network relationships between these genes, including transcriptional regulation and indirect interactions. In future research, we plan to employ novel network analysis tools, including those based on machine learning [54], to more accurately delineate the interaction networks of obesity genes.

## Data Availability

This study did not generate any new datasets. Instead, it utilized existing datasets, which are publicly available on the GEO database (accession numbers: GSE236708, GSE125688, and GSE251912) and the Human Brain Cell Atlas v1.0.

## Supporting Information

Supporting Figure 1: The graphical abstract of the analysis methods.

Supporting Figure 2: Subclustering of PPI network of obesity-risk genes.

Supporting Figure 3: Single-cell expression analysis of obesity-risk genes in human PBMCs. (a) Heatmap showing the average expression of obesity risk genes in different PBMC clusters. (b) Violin plot showing selected obesity-risk genes that are highly expressed in PBMCs.

Supporting Figure 4: Summary of commonly expressed obesity-risk genes in different cell types.

Supporting Table 1: Identification of 5097 SNPs that was associated with obesity.

Supporting Table 2: Identification of 802 obesity-risk genes.
