# Supplementary material for: A Complex Network of Obesity-Risk Genes Revealed by Systematic Bioinformatics and Single-Cell Transcriptomic Analyses
Source: J Obes. 2025 Mar 31;2025:7821115. doi: 10.1155/jobe/7821115 (PMC11976034; doi:10.1155/jobe/7821115)
Supplement: Supporting Information — Additional supporting information can be found online in the Supporting Information section. [file 7821115.f1.zip › Supplementary Table 1.pdf]

STUDY ACCESSION	CHR_ID	CHR_POS	MAPPED GENE	SNPS	CONTEXT	P-VALUE
GCST90255621	1	81913762	ADGRB3	rs284227	intron_variant	5.00E-15
GCST009003	1	81913762	ADGRL2	rs284227	intron_variant	6.00E-15
GCST009004	1	81913762	ADGRL2	rs284227	intron_variant	6.00E-15
GCST90179150	1	81913762	ADGRL2	rs284227	intron_variant	7.00E-14
GCST009001	1	82000094	ADGRL2	rs4970658	intergenic_variant	1.00E-13
GCST90255621	1	48972333	AGAP1	rs3127553	intron_variant	2.00E-32
GCST90179150	1	49124175	AGBL4	rs657452	intron_variant	2.00E-31
GCST009004	1	49244592	AGBL4	rs12121950	intron_variant	7.00E-24
GCST009003	1	49244592	AGBL4	rs12121950	intron_variant	7.00E-24
GCST009001	1	49827749	AGBL4	rs12128108	intron_variant	3.00E-22
GCST90018947	1	49244592	AGBL4	rs12121950	intron_variant	6.00E-20
GCST007039	1	49531287	AGBL4	rs1167311	intron_variant	1.00E-19
GCST009871	1	49021309	AGBL4	rs2224976	intron_variant	1.00E-16
GCST006368	1	48972333	AGBL4	rs3127553	intron_variant	3.00E-16
GCST006368	1	49124175	AGBL4	rs657452	intron_variant	5.00E-16
GCST004904	1	49124175	AGBL4	rs657452	intron_variant	2.00E-15
GCST009871	1	49634443	AGBL4	rs6665841	intron_variant	4.00E-15
GCST009871	1	48765589	AGBL4	rs7553454	intron_variant	3.00E-14
GCST002783	1	49124175	AGBL4	rs657452	intron_variant	2.00E-13
GCST002783	1	49124175	AGBL4	rs657452	intron_variant	5.00E-13
GCST90267268	1	48967331	AGBL4	rs1343431	intron_variant	2.00E-12
GCST005951	1	48972333	AGBL4	rs3127553	intron_variant	7.00E-12
GCST005951	1	49124175	AGBL4	rs657452	intron_variant	2.00E-11
GCST009004	1	77501838	AIF1	rs12049202	intron_variant	3.00E-29
GCST009001	1	77501838	AK5	rs12049202	intron_variant	3.00E-29
GCST009003	1	77514550	AK5	rs12729914	intron_variant	2.00E-27
GCST009871	1	77501838	AK5	rs12049202	intron_variant	9.00E-26
GCST90255621	1	77479047	AK5	rs11162351	intron_variant	3.00E-25
GCST90255621	1	77338557	AK5	rs17378915	intron_variant	6.00E-22
GCST90255621	1	77498584	AK5	rs7514041	intron_variant	3.00E-20
GCST90179150	1	77338557	AK5	rs17378915	intron_variant	2.00E-16
GCST90179150	1	77498584	AK5	rs7514041	intron_variant	2.00E-16
GCST009871	1	77523274	AK5	rs61777072	intron_variant	3.00E-14
GCST009871	1	77453625	AK5	rs10873942	intron_variant	2.00E-13
GCST90255621	1	243520717	AKAP8P1 - JKAMPP1	rs946824	intron_variant	4.00E-16
GCST009004	1	243520717	AKT3	rs946824	intron_variant	5.00E-15
GCST90179150	1	243520717	AKT3	rs946824	intron_variant	8.00E-15
GCST006368	1	243583332	AKT3	rs1074657	intron_variant	2.00E-14
GCST007039	1	243669258	AKT3	rs4658403	intron_variant	6.00E-12
GCST009871	1	243577143	AKT3	rs72761606	intron_variant	2.00E-11
GCST009871	1	155489364	ASCL4	rs61812092	intron_variant	4.00E-14
GCST009871	1	155333306	ASH1L	rs151026328	intergenic_variant	7.00E-12
GCST007039	1	151046385	BNC2	rs6587552	intron_variant	3.00E-14
GCST009001	1	151046704	BNIPL	rs6587553	3_prime_UTR_variant	5.00E-11
GCST90255621	1	190270777	BPTF	rs10920678	intron_variant	1.00E-32
GCST90018947	1	190308576	BRINP3	rs3098843	intron_variant	4.00E-22
GCST90179150	1	190270777	BRINP3	rs10920678	intron_variant	5.00E-20
GCST009004	1	190270777	BRINP3	rs10920678	intron_variant	7.00E-20
GCST009003	1	190270777	BRINP3	rs10920678	intron_variant	7.00E-20
GCST009871	1	190325596	BRINP3	rs815163	intron_variant	9.00E-16

GCST007039	1	190325596	BRINP3	rs815163	intron_variant	1.00E-15
GCST004904	1	190270777	BRINP3	rs10920678	intron_variant	3.00E-13
GCST006368	1	190270777	BRINP3	rs10920678	intron_variant	7.00E-11
GCST90255621	1	7667794	CAMTA1	rs1891215	intron_variant	5.00E-15
GCST009004	1	7667794	CAMTA1	rs1891215	intron_variant	7.00E-11
GCST004046	1	109275684	CELSR2	rs629301	3_prime_UTR_variant	2.00E-51
GCST004045	1	109275684	CELSR2	rs629301	3_prime_UTR_variant	1.00E-49
GCST90179150	1	1933859	CFAP74	rs2803316	intron_variant	1.00E-16
GCST009004	1	1933859	CFAP74	rs2803316	intron_variant	2.00E-16
GCST009001	1	1933859	CFAP74	rs2803316	intron_variant	2.00E-16
GCST009871	1	1933087	CFAP74	rs3121830	intron_variant	3.00E-14
GCST90255621	1	31698605	COL16A1	rs2228550	stop_gained	3.00E-17
GCST009868	1	31699492	COL16A1	rs6681149	intron_variant	3.00E-13
GCST009866	1	31699492	COL16A1	rs6681149	intron_variant	3.00E-13
GCST009004	1	31726197	COL16A1	rs945211	intergenic_variant	5.00E-13
GCST007039	1	31726197	COL16A1	rs945211	intergenic_variant	7.00E-12
GCST009871	1	31726197	COL16A1	rs945211	intergenic_variant	9.00E-12
GCST90179150	1	31707759	COL16A1	rs4295917	TF_binding_site_variant	1.00E-11
GCST90018947	1	31707657	COL16A1	rs10798886	regulatory_region_variant	2.00E-11
GCST90255621	1	31674452	COL16A1	rs4949457	intron_variant	4.00E-11
GCST90255621	1	197287960	CRB1	rs1009188	intron_variant	2.00E-16
GCST009004	1	197287960	CRB1	rs1009188	intron_variant	7.00E-11
GCST90255621	1	6655330	DNAJC11	rs6577584	intron_variant	3.00E-20
GCST009004	1	6655330	DNAJC11	rs6577584	intron_variant	3.00E-11
GCST90255621	1	97883329	DPYD	rs1801265	missense_variant	2.00E-16
GCST009004	1	97850337	DPYD	rs12072739	intron_variant	2.00E-13
GCST009871	1	97861577	DPYD	rs75641275	intron_variant	3.00E-13
GCST90179150	1	97861577	DPYD	rs75641275	intron_variant	3.00E-13
GCST009003	1	97856823	DPYD	rs4379706	non_coding_transcript_exon_	4.00E-13
GCST007039	1	97861577	DPYD	rs75641275	intron_variant	8.00E-13
GCST90255621	1	96458541	EEF1A1P11	rs11165643	intergenic_variant	3.00E-40
GCST90179150	1	96458541	EEF1A1P11	rs11165643	intergenic_variant	5.00E-30
GCST007039	1	96458541	EEF1A1P11	rs11165643	intergenic_variant	4.00E-24
GCST009004	1	96421814	EEF1A1P11	rs2968487	regulatory_region_variant	8.00E-22
GCST009003	1	96421814	EEF1A1P11	rs2968487	regulatory_region_variant	8.00E-22
GCST005951	1	96458541	EEF1A1P11	rs11165643	intergenic_variant	1.00E-17
GCST009871	1	96429769	EEF1A1P11	rs553785	regulatory_region_variant	1.00E-15
GCST90018947	1	96469707	EEF1A1P11	rs12042392	intergenic_variant	2.00E-15
GCST90255621	1	96428624	EEF1A1P11	rs1967768	intergenic_variant	1.00E-14
GCST009871	1	96525025	EEF1A1P11	rs6686005	regulatory_region_variant	5.00E-14
GCST002783	1	96458541	EEF1A1P11	rs11165643	intergenic_variant	1.00E-13
GCST90179150	1	96498603	EEF1A1P11	rs12037224	intergenic_variant	1.00E-13
GCST006368	1	96458541	EEF1A1P11	rs11165643	intergenic_variant	3.00E-13
GCST002783	1	96458541	EEF1A1P11	rs11165643	intergenic_variant	2.00E-12
GCST009371	1	96421814	EEF1A1P11	rs2968487	regulatory_region_variant	3.00E-12
GCST009871	1	96137479	EEF1A1P47	rs12130426	intergenic_variant	3.00E-12
GCST90255621	1	50094148	ELAVL4	rs11583200	intron_variant	6.00E-23
GCST006368	1	50094148	ELAVL4	rs11583200	intron_variant	4.00E-13
GCST90255621	1	92543881	EVI5	rs7515577	intron_variant	1.00E-13
GCST90179150	1	92511033	EVI5	rs6690764	3_prime_UTR_variant	4.00E-12
GCST009004	1	92754350	EVI5	rs6604015	intron_variant	1.00E-11

GCST007039	1	92566910	EVI5	rs6422513	intron_variant	2.00E-11
GCST90179150	1	151130677	GABPB2	rs4357530	non_coding_transcript_exon_	5.00E-15
GCST90018947	1	151071561	GABPB2	rs4970936	intron_variant	4.00E-11
GCST90255621	1	78157942	GIPC2	rs17391694	intergenic_variant	3.00E-64
GCST007039	1	77984833	GIPC2	rs34517439	intron_variant	2.00E-40
GCST90179150	1	77984833	GIPC2	rs34517439	intron_variant	2.00E-38
GCST90018947	1	77984833	GIPC2	rs34517439	intron_variant	4.00E-24
GCST90255621	1	78119402	GIPC2	rs540742	missense_variant	9.00E-22
GCST90179150	1	78003976	GIPC2	rs11162405	intron_variant	3.00E-18
GCST006368	1	77998184	GIPC2	rs4130548	intron_variant	2.00E-16
GCST009871	1	78129697	GIPC2	rs651413	intron_variant	1.00E-14
GCST005951	1	77981077	GIPC2	rs12401738	intron_variant	4.00E-14
GCST90179150	1	78149961	GIPC2	rs7524877	intergenic_variant	1.00E-11
GCST009004	1	109612066	GNAT2	rs17024393	intron_variant	7.00E-39
GCST009001	1	109612066	GNAT2	rs17024393	intron_variant	7.00E-39
GCST90018947	1	109612066	GNAT2	rs17024393	intron_variant	7.00E-18
GCST004557	1	109612066	GNAT2	rs17024393	intron_variant	2.00E-15
GCST004557	1	109612066	GNAT2	rs17024393	intron_variant	2.00E-15
GCST004558	1	109612066	GNAT2	rs17024393	intron_variant	7.00E-14
GCST002783	1	109612066	GNAT2	rs17024393	intron_variant	7.00E-14
GCST004558	1	109612066	GNAT2	rs17024393	intron_variant	1.00E-13
GCST002783	1	109612066	GNAT2	rs17024393	intron_variant	2.00E-13
GCST004495	1	109612066	GNAT2	rs17024393	intron_variant	5.00E-13
GCST006368	1	109612066	GNAT2	rs17024393	intron_variant	6.00E-13
GCST004559	1	109612066	GNAT2	rs17024393	intron_variant	9.00E-13
GCST004559	1	109612066	GNAT2	rs17024393	intron_variant	9.00E-13
GCST004497	1	109612066	GNAT2	rs17024393	intron_variant	2.00E-11
GCST004557	1	109612066	GNAT2	rs17024393	intron_variant	9.00E-11
GCST009003	1	1914634	GNB1	rs2803298	regulatory_region_variant	2.00E-16
GCST90255621	1	1881249	GNB1	rs6603803	intron_variant	1.00E-15
GCST90018727	1	155780079	GON4L	rs2985707	intron_variant	5.00E-11
GCST004904	1	155797917	GON4L	rs860295	intron_variant	9.00E-11
GCST90255621	1	109540264	GPR61	rs7550711	intron_variant	2.00E-53
GCST009003	1	109540264	GPR61	rs7550711	intron_variant	9.00E-39
GCST90179150	1	109540264	GPR61	rs7550711	intron_variant	2.00E-38
GCST007039	1	109539929	GPR61	rs41279738	5_prime_UTR_variant	5.00E-29
GCST009871	1	109539929	GPR61	rs41279738	5_prime_UTR_variant	2.00E-28
GCST005951	1	109540264	GPR61	rs7550711	intron_variant	8.00E-16
GCST009004	1	80346335	HMGB1P18 - HNRNPA1P64	rs6696828	intergenic_variant	6.00E-11
GCST90179150	1	80346526	HMGB1P18 - HNRNPA1P64	rs6697039	intergenic_variant	6.00E-11
GCST90179150	1	111730863	INKA2	rs1546924	intron_variant	1.00E-17
GCST009004	1	111730863	INKA2	rs1546924	intron_variant	2.00E-17
GCST009001	1	111730863	INKA2	rs1546924	intron_variant	2.00E-17
GCST009003	1	201831740	IPO9	rs2820295	intron_variant	6.00E-39
GCST009004	1	201831740	IPO9	rs2820295	intron_variant	6.00E-39
GCST009001	1	201857212	IPO9	rs2172935	intron_variant	6.00E-39
GCST90179150	1	201876447	IPO9	rs8024	3_prime_UTR_variant	1.00E-36
GCST007039	1	201831383	IPO9	rs2678204	intron_variant	1.00E-32
GCST009871	1	201831383	IPO9	rs2678204	intron_variant	5.00E-32
GCST90018947	1	201831740	IPO9	rs2820295	intron_variant	1.00E-28
GCST006368	1	201831883	IPO9	rs2494114	intron_variant	3.00E-20

GCST004495	1	201815159	IPO9	rs2820292	intron_variant	5.00E-11
GCST005951	1	201815159	IPO9	rs2820292	intron_variant	8.00E-11
GCST007039	1	45720054	IPP	rs115092994	intron_variant	5.00E-13
GCST009871	1	45720054	IPP	rs115092994	intron_variant	2.00E-12
GCST90179150	1	45699867	IPP	rs116792274	3_prime_UTR_variant	3.00E-11
GCST90255621	1	111775862	KCND3	rs12033257	3_prime_UTR_variant	5.00E-18
GCST009003	1	111775862	KCND3	rs12033257	3_prime_UTR_variant	5.00E-17
GCST007039	1	111781489	KCND3	rs2618039	non_coding_transcript_exon_	1.00E-13
GCST009871	1	111775862	KCND3	rs12033257	3_prime_UTR_variant	6.00E-13
GCST90018947	1	111770809	KCND3	rs34519355	3_prime_UTR_variant	7.00E-12
GCST90179150	1	32766924	KIAA1522	rs12022461	intron_variant	6.00E-14
GCST009004	1	32766924	KIAA1522	rs12022461	intron_variant	1.00E-13
GCST009871	1	32768527	KIAA1522	rs3737992	intron_variant	4.00E-12
GCST007039	1	32768527	KIAA1522	rs3737992	intron_variant	2.00E-11
GCST90104632	1	65525520	LEPR	rs2767486	intron_variant	6.00E-34
GCST90104633	1	65525520	LEPR	rs2767486	intron_variant	1.00E-25
GCST90104634	1	65525520	LEPR	rs2767486	intron_variant	7.00E-25
GCST90104631	1	65525520	LEPR	rs2767486	intron_variant	2.00E-24
GCST009291	1	65525520	LEPR	rs2767486	intron_variant	2.00E-21
GCST009294	1	65525520	LEPR	rs2767486	intron_variant	3.00E-20
GCST90104635	1	65525520	LEPR	rs2767486	intron_variant	3.00E-19
GCST90104634	1	65440454	LEPR	rs10889551	intron_variant	5.00E-19
GCST90104632	1	65440454	LEPR	rs10889551	intron_variant	2.00E-17
GCST90255621	1	65513597	LEPR	rs11208659	intron_variant	7.00E-17
GCST009293	1	65525520	LEPR	rs2767486	intron_variant	4.00E-15
GCST90104633	1	65440454	LEPR	rs10889551	intron_variant	4.00E-15
GCST009295	1	65525520	LEPR	rs2767486	intron_variant	7.00E-15
GCST009290	1	65525520	LEPR	rs2767486	intron_variant	7.00E-14
GCST90104635	1	65440454	LEPR	rs10889551	intron_variant	2.00E-13
GCST90104631	1	65440454	LEPR	rs10889551	intron_variant	7.00E-12
GCST009871	1	65662556	LEPR	rs6664626	intergenic_variant	4.00E-11
GCST90179150	1	65517943	LEPR	rs11208660	intron_variant	8.00E-11
GCST90255621	1	209370215	LINC01698	rs17014375	intron_variant	2.00E-12
GCST009004	1	209370215	LINC01698	rs17014375	intron_variant	4.00E-11
GCST90255621	1	95719742	LINC02607	rs11584409	intron_variant	4.00E-13
GCST90255621	1	95824178	LINC02607	rs1361739	intergenic_variant	2.00E-28
GCST009001	1	95824178	LINC02607	rs1361739	intergenic_variant	3.00E-17
GCST90179150	1	95824178	LINC02607	rs1361739	intergenic_variant	2.00E-16
GCST009871	1	95819829	LINC02607	rs1342391	intergenic_variant	1.00E-15
GCST007039	1	95814900	LINC02607	rs4472800	intergenic_variant	1.00E-11
GCST90267268	1	95809372	LINC02607	rs11165493	intergenic_variant	7.00E-11
GCST90255621	1	96137479	LINC02790 - RNU1-130P	rs12130426	intergenic_variant	3.00E-20
GCST90179150	1	96137479	LINC02790 - RNU1-130P	rs12130426	intergenic_variant	1.00E-12
GCST90255621	1	96036407	LINC02790 - RNU1-130P	rs12125523	intergenic_variant	6.00E-11
GCST008129	1	201900129	LMOD1	rs2820312	missense_variant	5.00E-36
GCST004904	1	201903136	LMOD1	rs2820315	intron_variant	3.00E-12
GCST90255621	1	219514090	LYPLAL1	rs2785990	intergenic_variant	1.00E-16
GCST90271771	1	219467338	LYPLAL1	rs2820436	intergenic_variant	2.00E-15
GCST90271770	1	219470882	LYPLAL1	rs2605100	intergenic_variant	1.00E-12
GCST009107	1	219455340	LYPLAL1	rs6689335	intron_variant	3.00E-12
GCST90271767	1	219575476	LYPLAL1	rs2820446	intergenic_variant	3.00E-12

GCST90255621	1	39336143	MACF1	rs41270807	missense_variant	2.00E-25
GCST009871	1	39096955	MACF1	rs112566467	intron_variant	8.00E-16
GCST90179150	1	39094578	MACF1	rs112646560	intron_variant	1.00E-15
GCST007039	1	39096955	MACF1	rs112566467	intron_variant	2.00E-15
GCST009004	1	39096955	MACF1	rs112566467	intron_variant	6.00E-15
GCST009003	1	39096955	MACF1	rs112566467	intron_variant	6.00E-15
GCST008129	1	39370145	MACF1	rs2296172	missense_variant	4.00E-11
GCST90255621	1	39487150	MACF1	rs1569053	3_prime_UTR_variant	5.00E-11
GCST009004	1	46039475	MAST2	rs1707322	regulatory_region_variant	2.00E-14
GCST90179150	1	46039475	MAST2	rs1707322	regulatory_region_variant	2.00E-13
GCST90255621	1	46021496	MAST2	rs946526	intron_variant	6.00E-13
GCST009001	1	46021880	MAST2	rs2275426	intron_variant	5.00E-12
GCST90255621	1	156080086	MEX3A	rs61813324	intron_variant	3.00E-27
GCST009004	1	156080086	MEX3A	rs61813324	intron_variant	3.00E-24
GCST009003	1	156080086	MEX3A	rs61813324	intron_variant	3.00E-24
GCST009001	1	156080086	MEX3A	rs61813324	intron_variant	3.00E-24
GCST90179150	1	156080086	MEX3A	rs61813324	intron_variant	4.00E-24
GCST009871	1	156080086	MEX3A	rs61813324	intron_variant	6.00E-24
GCST007039	1	156080086	MEX3A	rs61813324	intron_variant	8.00E-24
GCST90018947	1	156080086	MEX3A	rs61813324	intron_variant	5.00E-16
GCST009871	1	77787154	MIGA1	rs149049023	intron_variant	1.00E-14
GCST90179150	1	77829821	MIGA1	rs12085547	intron_variant	7.00E-12
GCST90255621	1	156436589	MIR9-1HG	rs11264483	intron_variant	2.00E-17
GCST009004	1	156437061	MIR9-1HG	rs4414033	intron_variant	4.00E-13
GCST90179150	1	156437061	MIR9-1HG	rs4414033	intron_variant	9.00E-13
GCST007039	1	156436589	MIR9-1HG	rs11264483	intron_variant	4.00E-12
GCST007039	1	175032043	MRPS14 - ENTR1P2	rs77560793	intergenic_variant	7.00E-13
GCST009871	1	175032043	MRPS14 - ENTR1P2	rs77560793	intergenic_variant	6.00E-12
GCST009001	1	175032043	MRPS14 - ENTR1P2	rs77560793	intergenic_variant	1.00E-11
GCST90255621	1	11224279	MTOR	rs10779751	intron_variant	6.00E-14
GCST90179150	1	11224279	MTOR	rs10779751	intron_variant	2.00E-13
GCST009001	1	11152401	MTOR	rs11581010	non_coding_transcript_exon_	9.00E-12
GCST007039	1	11147212	MTOR	rs2791643	intron_variant	2.00E-11
GCST009871	1	11192659	MTOR	rs1205593	intron_variant	5.00E-11
GCST009871	1	96634211	NDUFS5P2	rs11165687	intergenic_variant	2.00E-14
GCST009871	1	96650225	NDUFS5P2	rs28505555	intergenic_variant	2.00E-12
GCST90255621	1	72299433	NEGR1	rs2568958	intron_variant	3.00E-88
GCST009004	1	72285502	NEGR1	rs3101336	intron_variant	5.00E-54
GCST009003	1	72299433	NEGR1	rs2568958	intron_variant	1.00E-53
GCST007039	1	72287256	NEGR1	rs2613498	intron_variant	4.00E-40
GCST009871	1	72288631	NEGR1	rs61765651	intron_variant	2.00E-38
GCST006368	1	72285502	NEGR1	rs3101336	intron_variant	7.00E-36
GCST004904	1	72285502	NEGR1	rs3101336	intron_variant	1.00E-29
GCST90018947	1	72284787	NEGR1	rs34361149	intron_variant	2.00E-26
GCST002783	1	72285502	NEGR1	rs3101336	intron_variant	3.00E-26
GCST002783	1	72285502	NEGR1	rs3101336	intron_variant	6.00E-26
GCST002783	1	72285502	NEGR1	rs3101336	intron_variant	9.00E-17
GCST009871	1	72094057	NEGR1	rs727726	intron_variant	1.00E-16
GCST002783	1	72285502	NEGR1	rs3101336	intron_variant	2.00E-16
GCST004497	1	72285502	NEGR1	rs3101336	intron_variant	3.00E-16
GCST004495	1	72285502	NEGR1	rs3101336	intron_variant	1.00E-15

GCST001955	1	72299433	NEGR1	rs2568958	intron_variant	2.00E-14
GCST90255621	1	72274390	NEGR1	rs12127789	intron_variant	4.00E-14
GCST009371	1	72263459	NEGR1	rs1620977	intron_variant	9.00E-13
GCST005950	1	72109877	NEGR1	rs2821248	intron_variant	1.00E-12
GCST005951	1	72109877	NEGR1	rs2821248	intron_variant	1.00E-12
GCST90255621	1	71983116	NEGR1	rs2801321	intron_variant	2.00E-12
GCST004495	1	72285502	NEGR1	rs3101336	intron_variant	2.00E-12
GCST90255621	1	72191375	NEGR1	rs1342862	intron_variant	7.00E-12
GCST005953	1	72109877	NEGR1	rs2821248	intron_variant	8.00E-12
GCST004497	1	72285502	NEGR1	rs3101336	intron_variant	8.00E-12
GCST000296	1	72299433	NEGR1	rs2568958	intron_variant	1.00E-11
GCST006802	1	72285502	NEGR1	rs3101336	intron_variant	7.00E-11
GCST90255621	1	107342396	NTNG1	rs12035149	intron_variant	2.00E-20
GCST90255621	1	107434453	NTNG1	rs17531363	intron_variant	9.00E-16
GCST009004	1	107419706	NTNG1	rs11185111	intron_variant	1.00E-11
GCST009003	1	107458685	NTNG1	rs11802147	intron_variant	1.00E-11
GCST90179150	1	107447592	NTNG1	rs1444041	intron_variant	2.00E-11
GCST009871	1	107429051	NTNG1	rs77848106	intron_variant	3.00E-11
GCST009871	1	39622371	PABPC4	rs17343290	intergenic_variant	1.00E-11
GCST007039	1	39571272	PABPC4	rs61743745	synonymous_variant	3.00E-11
GCST009871	1	39571272	PABPC4	rs61743745	synonymous_variant	5.00E-11
GCST90255621	1	2512975	PANK4	rs7535528	missense_variant	4.00E-20
GCST90179150	1	2512975	PANK4	rs7535528	missense_variant	2.00E-17
GCST90255621	1	62114219	PATJ	rs12140153	missense_variant	4.00E-36
GCST007039	1	62114219	PATJ	rs12140153	missense_variant	8.00E-27
GCST009871	1	62114219	PATJ	rs12140153	missense_variant	4.00E-26
GCST009004	1	62114219	PATJ	rs12140153	missense_variant	1.00E-25
GCST009001	1	62114219	PATJ	rs12140153	missense_variant	1.00E-25
GCST009003	1	62114219	PATJ	rs12140153	missense_variant	1.00E-25
GCST90179150	1	62114219	PATJ	rs12140153	missense_variant	5.00E-24
GCST90018947	1	62114219	PATJ	rs12140153	missense_variant	6.00E-16
GCST90179150	1	62055633	PATJ	rs991191	intron_variant	2.00E-13
GCST90255621	1	62026543	PATJ	rs1332630	intron_variant	2.00E-13
GCST006368	1	62129005	PATJ	rs2481665	intron_variant	1.00E-12
GCST009871	1	62055633	PATJ	rs991191	intron_variant	2.00E-12
GCST009871	1	62163647	PATJ	rs41300331	3_prime_UTR_variant	2.00E-11
GCST004495	1	62129005	PATJ	rs2481665	intron_variant	3.00E-11
GCST90179150	1	65995718	PDE4B	rs2503185	intron_variant	1.00E-14
GCST007039	1	65969060	PDE4B	rs7519259	intron_variant	3.00E-14
GCST009004	1	65981711	PDE4B	rs6690398	intron_variant	2.00E-13
GCST009871	1	65954222	PDE4B	rs6421482	intron_variant	8.00E-13
GCST90018947	1	65925125	PDE4B	rs4655591	intron_variant	7.00E-12
GCST009001	1	65928049	PDE4B	rs2997084	intron_variant	1.00E-11
GCST90255621	1	107075085	PRMT6	rs1730859	intergenic_variant	1.00E-15
GCST90179150	1	107075085	PRMT6	rs1730859	intergenic_variant	2.00E-12
GCST009004	1	107075085	PRMT6	rs1730859	intergenic_variant	3.00E-12
GCST007039	1	171486183	PRRC2C	rs34720381	intron_variant	1.00E-13
GCST009871	1	171486183	PRRC2C	rs34720381	intron_variant	5.00E-13
GCST009004	1	171486183	PRRC2C	rs34720381	intron_variant	8.00E-12
GCST90179150	1	171486183	PRRC2C	rs34720381	intron_variant	1.00E-11
GCST90255621	1	171486183	PRRC2C	rs34720381	intron_variant	2.00E-11

GCST009004	1	151034063	PRUNE1	rs3738476	synonymous_variant	3.00E-14
GCST009871	1	151034063	PRUNE1	rs3738476	synonymous_variant	1.00E-13
GCST90255621	1	96958238	PTBP2	rs6671669	intergenic_variant	2.00E-20
GCST90179150	1	96965496	PTBP2	rs995258	intergenic_variant	4.00E-16
GCST009871	1	96822059	PTBP2	rs12066944	3_prime_UTR_variant	2.00E-13
GCST007039	1	96965496	PTBP2	rs995258	intergenic_variant	8.00E-12
GCST009871	1	78364361	PTGFR	rs12142416	intron_variant	4.00E-16
GCST90255621	1	78385016	PTGFR	rs11162479	intron_variant	1.00E-11
GCST90255621	1	202147829	PTPN7	rs4623766	3_prime_UTR_variant	7.00E-15
GCST009004	1	202147110	PTPN7	rs9077	3_prime_UTR_variant	3.00E-13
GCST90179150	1	202147110	PTPN7	rs9077	3_prime_UTR_variant	2.00E-12
GCST90255621	1	174352859	RABGAP1L	rs61828641	intron_variant	9.00E-18
GCST90018947	1	174401966	RABGAP1L	rs61826865	intron_variant	4.00E-17
GCST90255621	1	174964277	RABGAP1L	rs12122361	intron_variant	2.00E-15
GCST007039	1	174352859	RABGAP1L	rs61828641	intron_variant	1.00E-13
GCST009004	1	174352859	RABGAP1L	rs61828641	intron_variant	2.00E-13
GCST009871	1	174352859	RABGAP1L	rs61828641	intron_variant	2.00E-13
GCST009003	1	174508962	RABGAP1L	rs12564992	intron_variant	3.00E-13
GCST90179150	1	174352859	RABGAP1L	rs61828641	intron_variant	1.00E-12
GCST90179150	1	174915992	RABGAP1L	rs6691857	intron_variant	7.00E-12
GCST009871	1	174961819	RABGAP1L	rs12074821	intron_variant	2.00E-11
GCST90255621	1	118310352	RNA5SP56 - PSMC1P12	rs12731372	regulatory_region_variant	2.00E-11
GCST009004	1	118321993	RNA5SP56 - PSMC1P12	rs7534091	intergenic_variant	7.00E-11
GCST90179150	1	118321993	RNA5SP56 - PSMC1P12	rs7534091	intergenic_variant	8.00E-11
GCST90255621	1	78181124	RNFT1P2 - MGC27382	rs149600839	intergenic_variant	3.00E-12
GCST004557	1	78212446	RNFT1P2 - MGC27382	rs12406019	intergenic_variant	6.00E-11
GCST90255621	1	195069917	RNU6-983P - LINC01724	rs12141968	intergenic_variant	1.00E-14
GCST009871	1	195173715	RNU6-983P - LINC01724	rs672313	intergenic_variant	3.00E-14
GCST007039	1	195173715	RNU6-983P - LINC01724	rs672313	intergenic_variant	2.00E-13
GCST90018947	1	195060876	RNU6-983P - LINC01724	rs4657793	intergenic_variant	3.00E-13
GCST009004	1	194996070	RNU6-983P - LINC01724	rs2400414	intergenic_variant	6.00E-13
GCST90179150	1	195069917	RNU6-983P - LINC01724	rs12141968	intergenic_variant	6.00E-13
GCST009003	1	195067871	RNU6-983P - LINC01724	rs4474229	intergenic_variant	4.00E-12
GCST009001	1	72372846	RPL31P12 - RNU6-1246P	rs1993709	intron_variant	1.00E-52
GCST005951	1	72358030	RPL31P12 - RNU6-1246P	rs990871	intron_variant	1.00E-33
GCST006368	1	72478934	RPL31P12 - RNU6-1246P	rs7550173	intron_variant	3.00E-24
GCST000830	1	72346757	RPL31P12 - RNU6-1246P	rs2815752	intron_variant	2.00E-22
GCST007241	1	72342868	RPL31P12 - RNU6-1246P	rs11209947	intron_variant	5.00E-20
GCST004557	1	72371807	RPL31P12 - RNU6-1246P	rs11209951	intron_variant	3.00E-16
GCST004557	1	72371807	RPL31P12 - RNU6-1246P	rs11209951	intron_variant	7.00E-16
GCST004558	1	72371807	RPL31P12 - RNU6-1246P	rs11209951	intron_variant	7.00E-16
GCST004558	1	72371807	RPL31P12 - RNU6-1246P	rs11209951	intron_variant	1.00E-15
GCST004557	1	72478934	RPL31P12 - RNU6-1246P	rs7550173	intron_variant	4.00E-13
GCST004557	1	72478934	RPL31P12 - RNU6-1246P	rs7550173	intron_variant	7.00E-13
GCST004558	1	72478934	RPL31P12 - RNU6-1246P	rs7550173	intron_variant	8.00E-12
GCST004558	1	72478934	RPL31P12 - RNU6-1246P	rs7550173	intron_variant	9.00E-12
GCST004559	1	72371807	RPL31P12 - RNU6-1246P	rs11209951	intron_variant	2.00E-11
GCST90255621	1	72412981	RPL31P12 - RNU6-1246P	rs72684636	intron_variant	2.00E-11
GCST004557	1	72478934	RPL31P12 - RNU6-1246P	rs7550173	intron_variant	3.00E-11
GCST90255621	1	72543762	RPL31P12 - RNU6-1246P	rs79852272	intron_variant	6.00E-11
GCST004557	1	72371807	RPL31P12 - RNU6-1246P	rs11209951	intron_variant	7.00E-11

GCST004558	1	72371807	RPL31P12 - RNU6-1246P	rs11209951	intron_variant	8.00E-11
GCST004559	1	72371807	RPL31P12 - RNU6-1246P	rs11209951	intron_variant	9.00E-11
GCST90091177	1	242939598	RSL24D1P4 - SEPTIN14P21	rs12405634	intergenic_variant	8.00E-13
GCST90255621	1	242822761	RSL24D1P4 - SEPTIN14P21	rs2491864	intergenic_variant	1.00E-12
GCST90255621	1	177920345	SEC16B	rs543874	intergenic_variant	7.00E-152
GCST009004	1	177920345	SEC16B	rs543874	intergenic_variant	3.00E-125
GCST009003	1	177920345	SEC16B	rs543874	intergenic_variant	3.00E-125
GCST009001	1	177920345	SEC16B	rs543874	intergenic_variant	3.00E-125
GCST90179150	1	177920345	SEC16B	rs543874	intergenic_variant	9.00E-125
GCST90018947	1	177919890	SEC16B	rs539515	intron_variant	2.00E-92
GCST009871	1	177919890	SEC16B	rs539515	intron_variant	6.00E-90
GCST007039	1	177919890	SEC16B	rs539515	intron_variant	1.00E-85
GCST009121	1	177929986	SEC16B	rs545608	intron_variant	2.00E-63
GCST004904	1	177920345	SEC16B	rs543874	intergenic_variant	7.00E-62
GCST006368	1	177920345	SEC16B	rs543874	intergenic_variant	4.00E-52
GCST005951	1	177920345	SEC16B	rs543874	intergenic_variant	3.00E-46
GCST004519	1	177920345	SEC16B	rs543874	intergenic_variant	6.00E-46
GCST002783	1	177920345	SEC16B	rs543874	intergenic_variant	2.00E-40
GCST008129	1	177933618	SEC16B	rs591120	missense_variant	5.00E-38
GCST004495	1	177920345	SEC16B	rs543874	intergenic_variant	1.00E-35
GCST002783	1	177920345	SEC16B	rs543874	intergenic_variant	3.00E-35
GCST004497	1	177920345	SEC16B	rs543874	intergenic_variant	6.00E-35
GCST004557	1	177920345	SEC16B	rs543874	intergenic_variant	2.00E-34
GCST004558	1	177920345	SEC16B	rs543874	intergenic_variant	3.00E-34
GCST004904	1	177883445	SEC16B	rs633715	intron_variant	1.00E-33
GCST002783	1	177920345	SEC16B	rs543874	intergenic_variant	1.00E-33
GCST90018727	1	177883445	SEC16B	rs633715	intron_variant	3.00E-32
GCST004495	1	177920345	SEC16B	rs543874	intergenic_variant	3.00E-31
GCST004497	1	177920345	SEC16B	rs543874	intergenic_variant	9.00E-31
GCST009871	1	177929664	SEC16B	rs623479	3_prime_UTR_variant	4.00E-29
GCST004558	1	177920345	SEC16B	rs543874	intergenic_variant	6.00E-29
GCST004557	1	177920345	SEC16B	rs543874	intergenic_variant	8.00E-29
GCST004557	1	177920345	SEC16B	rs543874	intergenic_variant	6.00E-28
GCST004499	1	177920345	SEC16B	rs543874	intergenic_variant	6.00E-28
GCST004558	1	177883445	SEC16B	rs633715	intron_variant	2.00E-27
GCST004499	1	177920345	SEC16B	rs543874	intergenic_variant	4.00E-25
GCST004559	1	177920345	SEC16B	rs543874	intergenic_variant	2.00E-24
GCST004557	1	177883445	SEC16B	rs633715	intron_variant	5.00E-24
GCST90255621	1	177951619	SEC16B	rs3828137	intron_variant	1.00E-23
GCST90179150	1	177873027	SEC16B	rs943764	intron_variant	1.00E-23
GCST004559	1	177920345	SEC16B	rs543874	intergenic_variant	2.00E-23
GCST000830	1	177920345	SEC16B	rs543874	intergenic_variant	4.00E-23
GCST004558	1	177920345	SEC16B	rs543874	intergenic_variant	7.00E-23
GCST90179150	1	177951619	SEC16B	rs3828137	intron_variant	9.00E-23
GCST009871	1	177861186	SEC16B	rs144847043	intron_variant	2.00E-22
GCST006802	1	177920345	SEC16B	rs543874	intergenic_variant	2.00E-22
GCST90255621	1	178012425	SEC16B	rs11584600	intron_variant	3.00E-22
GCST009764	1	177920345	SEC16B	rs543874	intergenic_variant	1.00E-21
GCST004559	1	177883445	SEC16B	rs633715	intron_variant	2.00E-21
GCST009871	1	177895419	SEC16B	rs10798580	intron_variant	3.00E-21
GCST007240	1	177925152	SEC16B	rs506589	intron_variant	3.00E-20

GCST90255622	1	177920345	SEC16B	rs543874	intergenic_variant	7.00E-20
GCST003177	1	177920345	SEC16B	rs543874	intergenic_variant	2.00E-19
GCST002461	1	177904075	SEC16B	rs574367	intron_variant	2.00E-19
GCST004559	1	177920345	SEC16B	rs543874	intergenic_variant	2.00E-19
GCST90179150	1	178011085	SEC16B	rs7531837	intron_variant	3.00E-19
GCST007241	1	177839998	SEC16B	rs12735657	intron_variant	7.00E-19
GCST003177	1	177920345	SEC16B	rs543874	intergenic_variant	2.00E-17
GCST90131907	1	177883445	SEC16B	rs633715	intron_variant	2.00E-17
GCST009871	1	177997870	SEC16B	rs35308250	intron_variant	3.00E-17
GCST009107	1	177929986	SEC16B	rs545608	intron_variant	4.00E-17
GCST008025	1	177920345	SEC16B	rs543874	intergenic_variant	6.00E-17
GCST90255621	1	177960366	SEC16B	rs34246968	missense_variant	6.00E-16
GCST005950	1	177933618	SEC16B	rs591120	missense_variant	7.00E-16
GCST009871	1	177906689	SEC16B	rs72702742	intron_variant	9.00E-16
GCST90002409	1	177920345	SEC16B	rs543874	intergenic_variant	2.00E-15
GCST005951	1	177933618	SEC16B	rs591120	missense_variant	2.00E-15
GCST009871	1	178009724	SEC16B	rs79295525	intron_variant	5.00E-15
GCST009871	1	177955443	SEC16B	rs61816282	intron_variant	6.00E-15
GCST90179150	1	177797481	SEC16B	rs3922649	intron_variant	7.00E-15
GCST90255621	1	177799955	SEC16B	rs72717429	intron_variant	9.00E-15
GCST90255621	1	177797481	SEC16B	rs3922649	intron_variant	2.00E-14
GCST005953	1	177933618	SEC16B	rs591120	missense_variant	5.00E-14
GCST009871	1	177965456	SEC16B	rs6679120	intron_variant	7.00E-14
GCST90255621	1	177921252	SEC16B	rs58689292	intergenic_variant	8.00E-14
GCST004519	1	177920345	SEC16B	rs543874	intergenic_variant	2.00E-13
GCST001967	1	177920345	SEC16B	rs543874	intergenic_variant	2.00E-13
GCST90239604	1	177886382	SEC16B	rs516636	intron_variant	2.00E-13
GCST90179150	1	177799955	SEC16B	rs72717429	intron_variant	6.00E-13
GCST004560	1	177920345	SEC16B	rs543874	intergenic_variant	2.00E-12
GCST004519	1	177920345	SEC16B	rs543874	intergenic_variant	4.00E-12
GCST001955	1	177883445	SEC16B	rs633715	intron_variant	5.00E-12
GCST008025	1	177886382	SEC16B	rs516636	intron_variant	1.00E-11
GCST008025	1	177904075	SEC16B	rs574367	intron_variant	1.00E-11
GCST90255621	1	177962397	SEC16B	rs4650985	intron_variant	2.00E-11
GCST008025	1	177883445	SEC16B	rs633715	intron_variant	2.00E-11
GCST002783	1	177920345	SEC16B	rs543874	intergenic_variant	3.00E-11
GCST002021	1	177920345	SEC16B	rs543874	intergenic_variant	4.00E-11
GCST90179150	1	177870477	SEC16B	rs10489881	intron_variant	6.00E-11
GCST90255621	1	156201695	SLC25A44	rs76102184	intron_variant	8.00E-17
GCST90179150	1	156201695	SLC25A44	rs76102184	intron_variant	3.00E-16
GCST009871	1	156201695	SLC25A44	rs76102184	intron_variant	4.00E-16
GCST90255621	1	1659060	SLC35E2B	rs79113395	TF_binding_site_variant	4.00E-22
GCST009004	1	1659060	SLC35E2B	rs79113395	TF_binding_site_variant	2.00E-20
GCST009003	1	1659060	SLC35E2B	rs79113395	TF_binding_site_variant	2.00E-20
GCST009871	1	1659060	SLC35E2B	rs79113395	TF_binding_site_variant	1.00E-19
GCST90179150	1	1669613	SLC35E2B	rs72634826	intron_variant	1.00E-19
GCST007039	1	1665061	SLC35E2B	rs72634819	3_prime_UTR_variant	1.00E-19
GCST009001	1	1659220	SLC35E2B	rs9661500	TF_binding_site_variant	6.00E-19
GCST90018947	1	1668542	SLC35E2B	rs72634822	intron_variant	5.00E-15
GCST90255621	1	54264363	SSBP3	rs3766431	intron_variant	8.00E-13
GCST009004	1	54263191	SSBP3	rs630602	intron_variant	1.00E-12

GCST009003	1	54263191	SSBP3	rs630602	intron_variant	1.00E-12
GCST90179150	1	54264978	SSBP3	rs3766430	intron_variant	3.00E-12
GCST90018947	1	209904236	SYT14	rs11119364	intron_variant	2.00E-14
GCST90255621	1	209913194	SYT14	rs12140373	intron_variant	2.00E-14
GCST90179150	1	209922182	SYT14	rs6661316	intron_variant	1.00E-13
GCST009004	1	209922182	SYT14	rs6661316	intron_variant	2.00E-13
GCST009871	1	209925121	SYT14	rs6695101	intron_variant	5.00E-12
GCST007039	1	209925121	SYT14	rs6695101	intron_variant	9.00E-12
GCST90255621	1	47224766	TAL1	rs2984618	intron_variant	4.00E-31
GCST90179150	1	47224766	TAL1	rs2984618	intron_variant	4.00E-24
GCST007039	1	47204853	TAL1	rs12144626	intergenic_variant	3.00E-17
GCST90018947	1	47204853	TAL1	rs12144626	intergenic_variant	1.00E-13
GCST009004	1	47217935	TAL1	rs2070929	3_prime_UTR_variant	3.00E-13
GCST006368	1	47219005	TAL1	rs977747	3_prime_UTR_variant	2.00E-11
GCST90255621	1	118961220	TBX15	rs984222	intron_variant	5.00E-20
GCST90179150	1	119004219	TBX15	rs10923724	intergenic_variant	3.00E-15
GCST009004	1	119004219	TBX15	rs10923724	intergenic_variant	1.00E-14
GCST009001	1	118961661	TBX15	rs984225	intron_variant	1.00E-13
GCST90271770	1	118973782	TBX15	rs61806235	intron_variant	6.00E-12
GCST007039	1	119004219	TBX15	rs10923724	intergenic_variant	1.00E-11
GCST90255621	1	118953802	TBX15	rs61806207	intron_variant	5.00E-11
GCST009871	1	119004219	TBX15	rs10923724	intergenic_variant	7.00E-11
GCST009004	1	23018558	TEX46	rs561136	intron_variant	1.00E-14
GCST007039	1	22986860	TEX46	rs10799778	intergenic_variant	2.00E-12
GCST009871	1	23018558	TEX46	rs561136	intron_variant	2.00E-11
GCST009003	1	74511741	TNNI3K	rs953567	intron_variant	2.00E-28
GCST009871	1	74511741	TNNI3K	rs953567	intron_variant	1.00E-18
GCST90179150	1	74540343	TNNI3K	rs3895907	intron_variant	1.00E-33
GCST009004	1	74525718	TNNI3K	rs1514177	intron_variant	8.00E-30
GCST009001	1	74525718	TNNI3K	rs1514177	intron_variant	8.00E-30
GCST90255621	1	74525960	TNNI3K	rs1514175	intron_variant	4.00E-28
GCST90018947	1	74534734	TNNI3K	rs5775241	intron_variant	1.00E-20
GCST007039	1	74539554	TNNI3K	rs7553158	intron_variant	2.00E-19
GCST004904	1	74536509	TNNI3K	rs12566985	intron_variant	6.00E-19
GCST006368	1	74532078	TNNI3K	rs12042908	intron_variant	1.00E-17
GCST005951	1	74539383	TNNI3K	rs7553348	intron_variant	4.00E-17
GCST005950	1	74527379	TNNI3K	rs1514174	intron_variant	1.00E-15
GCST002783	1	74536509	TNNI3K	rs12566985	intron_variant	2.00E-15
GCST002783	1	74536509	TNNI3K	rs12566985	intron_variant	3.00E-15
GCST005953	1	74527379	TNNI3K	rs1514174	intron_variant	3.00E-15
GCST90002409	1	74532078	TNNI3K	rs12042908	intron_variant	3.00E-14
GCST000830	1	74525960	TNNI3K	rs1514175	intron_variant	8.00E-14
GCST005951	1	74527379	TNNI3K	rs1514174	intron_variant	4.00E-13
GCST90179150	1	74565426	TNNI3K	rs12029405	intergenic_variant	3.00E-12
GCST002783	1	74536509	TNNI3K	rs12566985	intron_variant	1.00E-11
GCST002021	1	74525960	TNNI3K	rs1514175	intron_variant	3.00E-11
GCST009004	1	2808910	TTC34 - ACTRT2	rs7537581	intergenic_variant	6.00E-16
GCST009871	1	2806649	TTC34 - ACTRT2	rs4648450	intergenic_variant	1.00E-13
GCST90255621	1	96393890	UBE2WP1	rs531190	intergenic_variant	2.00E-15
GCST009871	1	96295390	UBE2WP1	rs80067794	intron_variant	8.00E-12
GCST90255621	1	156041653	UBQLN4	rs2297792	missense_variant	6.00E-19

GCST008129	1	156041653	UBQLN4	rs2297792	missense_variant	2.00E-13
GCST008129	1	155015228	ZBTB7B	rs141845046	missense_variant	8.00E-18
GCST90255621	1	155013615	ZBTB7B	rs3753639	intron_variant	2.00E-17
GCST90255621	1	155015228	ZBTB7B	rs141845046	missense_variant	2.00E-14
GCST009871	1	155013615	ZBTB7B	rs3753639	intron_variant	3.00E-13
GCST006368	1	77582646	ZZZ3	rs17381664	intron_variant	3.00E-15
GCST005951	1	77582646	ZZZ3	rs17381664	intron_variant	1.00E-14
GCST90179150	2	57841606	ABHD5	rs1106090	intergenic_variant	2.00E-14
GCST90255621	2	57841606	ACTG1P22	rs1106090	intergenic_variant	6.00E-14
GCST009871	2	57841606	ACTG1P22	rs1106090	intergenic_variant	8.00E-11
GCST90255621	2	57692382	ACTG1P22	rs4672221	intergenic_variant	5.00E-13
GCST90255621	2	24918669	ADARB1	rs11676272	missense_variant	1.00E-96
GCST90179150	2	24918669	ADCY3	rs11676272	missense_variant	2.00E-90
GCST009004	2	24913997	ADCY3	rs10203386	intron_variant	1.00E-78
GCST009003	2	24913997	ADCY3	rs10203386	intron_variant	1.00E-78
GCST009001	2	24863958	ADCY3	rs1529897	intron_variant	1.00E-72
GCST007039	2	24927247	ADCY3	rs6752378	intergenic_variant	2.00E-70
GCST009871	2	24913997	ADCY3	rs10203386	intron_variant	7.00E-67
GCST90018947	2	24927142	ADCY3	rs6749422	intergenic_variant	2.00E-59
GCST008129	2	24918669	ADCY3	rs11676272	missense_variant	4.00E-59
GCST009121	2	24913997	ADCY3	rs10203386	intron_variant	8.00E-43
GCST006368	2	24927427	ADCY3	rs10182181	intergenic_variant	3.00E-35
GCST004904	2	24927427	ADCY3	rs10182181	intergenic_variant	3.00E-32
GCST005951	2	24927427	ADCY3	rs10182181	intergenic_variant	2.00E-30
GCST005950	2	24915171	ADCY3	rs6737082	intron_variant	5.00E-26
GCST002783	2	24927427	ADCY3	rs10182181	intergenic_variant	8.00E-26
GCST005951	2	24915171	ADCY3	rs6737082	intron_variant	3.00E-24
GCST002783	2	24927427	ADCY3	rs10182181	intergenic_variant	9.00E-24
GCST003177	2	24918669	ADCY3	rs11676272	missense_variant	7.00E-23
GCST007241	2	24912569	ADCY3	rs2384060	intron_variant	9.00E-23
GCST90104634	2	24918669	ADCY3	rs11676272	missense_variant	3.00E-22
GCST000830	2	24935139	ADCY3	rs713586	intergenic_variant	6.00E-22
GCST90002409	2	24918669	ADCY3	rs11676272	missense_variant	2.00E-21
GCST003177	2	24927427	ADCY3	rs10182181	intergenic_variant	2.00E-21
GCST002783	2	24927427	ADCY3	rs10182181	intergenic_variant	4.00E-21
GCST005953	2	24915171	ADCY3	rs6737082	intron_variant	6.00E-20
GCST90104632	2	24918669	ADCY3	rs11676272	missense_variant	2.00E-19
GCST004495	2	24927427	ADCY3	rs10182181	intergenic_variant	7.00E-19
GCST004497	2	24927427	ADCY3	rs10182181	intergenic_variant	1.00E-18
GCST004558	2	24927247	ADCY3	rs6752378	intergenic_variant	2.00E-18
GCST90131907	2	24914454	ADCY3	rs10206196	intron_variant	3.00E-18
GCST90104633	2	24918669	ADCY3	rs11676272	missense_variant	4.00E-18
GCST004557	2	24927247	ADCY3	rs6752378	intergenic_variant	7.00E-18
GCST90179150	2	24799729	ADCY3	rs1550116	missense_variant	1.00E-17
GCST004558	2	24927247	ADCY3	rs6752378	intergenic_variant	3.00E-17
GCST007240	2	24936632	ADCY3	rs6738433	intergenic_variant	3.00E-17
GCST004557	2	24927247	ADCY3	rs6752378	intergenic_variant	3.00E-16
GCST004499	2	24927427	ADCY3	rs10182181	intergenic_variant	3.00E-16
GCST90239604	2	24892374	ADCY3	rs10165451	intron_variant	4.00E-16
GCST006802	2	24927427	ADCY3	rs10182181	intergenic_variant	6.00E-16
GCST004557	2	24927247	ADCY3	rs6752378	intergenic_variant	9.00E-16

GCST004558	2	24927247	ADCY3	rs6752378	intergenic_variant	9.00E-16
GCST009871	2	24927567	ADCY3	rs13313741	intergenic_variant	2.00E-15
GCST004559	2	24927247	ADCY3	rs6752378	intergenic_variant	4.00E-15
GCST004557	2	24927247	ADCY3	rs6752378	intergenic_variant	9.00E-15
GCST90104635	2	24918669	ADCY3	rs11676272	missense_variant	1.00E-14
GCST004558	2	24927247	ADCY3	rs6752378	intergenic_variant	2.00E-14
GCST90255621	2	24822326	ADCY3	rs11691159	3_prime_UTR_variant	2.00E-14
GCST004559	2	24927247	ADCY3	rs6752378	intergenic_variant	3.00E-14
GCST90267268	2	24932550	ADCY3	rs75503394	intergenic_variant	8.00E-14
GCST001415	2	24908447	ADCY3	rs6545814	intron_variant	1.00E-13
GCST90104631	2	24918669	ADCY3	rs11676272	missense_variant	4.00E-13
GCST004904	2	24935139	ADCY3	rs713586	intergenic_variant	5.00E-13
GCST009294	2	24911140	ADCY3	rs13035244	intron_variant	8.00E-13
GCST009295	2	24911140	ADCY3	rs13035244	intron_variant	8.00E-13
GCST90018727	2	24927142	ADCY3	rs6749422	intergenic_variant	1.00E-12
GCST008158	2	24899971	ADCY3	rs4077678	intron_variant	2.00E-12
GCST004495	2	24927427	ADCY3	rs10182181	intergenic_variant	2.00E-12
GCST90104636	2	24918669	ADCY3	rs11676272	missense_variant	3.00E-12
GCST90179150	2	24822326	ADCY3	rs11691159	3_prime_UTR_variant	3.00E-12
GCST004497	2	24927427	ADCY3	rs10182181	intergenic_variant	4.00E-12
GCST004559	2	24927247	ADCY3	rs6752378	intergenic_variant	5.00E-12
GCST004559	2	24927247	ADCY3	rs6752378	intergenic_variant	9.00E-12
GCST009107	2	24913997	ADCY3	rs10203386	intron_variant	1.00E-11
GCST002894	2	24918669	ADCY3	rs11676272	missense_variant	1.00E-11
GCST004499	2	24927427	ADCY3	rs10182181	intergenic_variant	2.00E-11
GCST009004	2	100185497	ADPGK	rs13002946	intron_variant	4.00E-20
GCST009003	2	100134688	AFF3	rs11677607	intron_variant	5.00E-20
GCST009871	2	100181450	AFF3	rs12712072	intron_variant	9.00E-17
GCST009001	2	100137627	AFF3	rs73966422	non_coding_transcript_exon_	2.00E-16
GCST009871	2	100198396	AFF3	rs4556997	regulatory_region_variant	1.00E-15
GCST90255621	2	100130111	AFF3	rs11692215	intron_variant	7.00E-14
GCST90255621	2	235833244	AFF3	rs908443	intron_variant	3.00E-15
GCST90255621	2	235854171	AGAP1	rs4414632	intron_variant	2.00E-12
GCST90255621	2	235994449	AGAP1	rs2317299	intron_variant	6.00E-12
GCST009871	2	235939844	AGAP1	rs7568228	intron_variant	3.00E-11
GCST007039	2	235833244	AGAP1	rs908443	intron_variant	5.00E-11
GCST90255621	2	295255	ALDH2	rs6548221	intron_variant	2.00E-14
GCST90179150	2	295255	ALKAL2	rs6548221	intron_variant	1.00E-12
GCST009871	2	292753	ALKAL2	rs12477065	intron_variant	1.00E-11
GCST007039	2	292753	ALKAL2	rs12477065	intron_variant	2.00E-11
GCST004046	2	21065449	APC	rs562338	intergenic_variant	3.00E-25
GCST004045	2	21065449	APOB	rs562338	intergenic_variant	5.00E-21
GCST006368	2	143203024	ARG1	rs6710871	intron_variant	3.00E-19
GCST90255621	2	143203024	ARHGAP15	rs6710871	intron_variant	6.00E-19
GCST009004	2	143203024	ARHGAP15	rs6710871	intron_variant	3.00E-16
GCST90179150	2	143203024	ARHGAP15	rs6710871	intron_variant	1.00E-15
GCST009003	2	143264143	ARHGAP15	rs13002158	intron_variant	1.00E-12
GCST009871	2	143201527	ARHGAP15	rs62171698	intron_variant	4.00E-11
GCST90255621	2	53765485	AS3MT	rs36020289	missense_variant	1.00E-12
GCST90018947	2	53812428	ASB3	rs2287347	intron_variant	2.00E-12
GCST009871	2	53634252	ASB3	rs59428052	intron_variant	1.00E-11

GCST007039	2	53634252	ASB3	rs59428052	intron_variant	1.00E-11
GCST009004	2	44492754	CAMKMT	rs786420	intron_variant	2.00E-12
GCST90179150	2	44511624	CAMKMT	rs698838	intron_variant	6.00E-12
GCST90179150	2	103750966	CAPZBP1	rs1379430	intergenic_variant	8.00E-13
GCST007039	2	103804400	CAPZBP1	rs6707445	intergenic_variant	1.00E-12
GCST009003	2	103692087	CAPZBP1	rs264962	intergenic_variant	2.00E-12
GCST009004	2	103692087	CAPZBP1	rs264962	intergenic_variant	2.00E-12
GCST90018947	2	103796466	CAPZBP1	rs72820274	intergenic_variant	6.00E-12
GCST90255621	2	86539089	CHMP3	rs7596717	intron_variant	2.00E-23
GCST009004	2	86585426	CHMP3	rs12714199	intron_variant	3.00E-16
GCST009003	2	86585426	CHMP3	rs12714199	intron_variant	3.00E-16
GCST90179150	2	86585426	CHMP3	rs12714199	intron_variant	1.00E-15
GCST007039	2	86536881	CHMP3	rs4832298	intron_variant	3.00E-15
GCST009871	2	86536881	CHMP3	rs4832298	intron_variant	5.00E-15
GCST009001	2	86579233	CHMP3	rs10209325	intron_variant	1.00E-14
GCST90018947	2	86576474	CHMP3	rs113655489	intron_variant	3.00E-11
GCST001526	2	164694691	COBLL1	rs7607980	missense_variant	4.00E-20
GCST90255621	2	164672366	COBLL1	rs13389219	intron_variant	2.00E-16
GCST011334	2	164672114	COBLL1	rs1128249	intron_variant	3.00E-16
GCST011336	2	164672114	COBLL1	rs1128249	intron_variant	2.00E-14
GCST90179150	2	164701742	COBLL1	rs12692738	intron_variant	5.00E-13
GCST011335	2	164672114	COBLL1	rs1128249	intron_variant	6.00E-11
GCST009004	2	164800277	COBLL1	rs355914	intron_variant	7.00E-11
GCST90271770	2	210678331	CPS1	rs715	3_prime_UTR_variant	1.00E-20
GCST009004	2	210678331	CPS1	rs715	3_prime_UTR_variant	1.00E-16
GCST009003	2	210678331	CPS1	rs715	3_prime_UTR_variant	1.00E-16
GCST90179150	2	210678331	CPS1	rs715	3_prime_UTR_variant	2.00E-16
GCST90255621	2	211120955	CPS1	rs10174373	intergenic_variant	9.00E-16
GCST90255621	2	210743655	CPS1	rs4673553	intergenic_variant	5.00E-15
GCST009871	2	210759245	CPS1	rs35394656	intergenic_variant	4.00E-13
GCST007039	2	210759245	CPS1	rs35394656	intergenic_variant	7.00E-13
GCST90179150	2	211125375	CPS1	rs12151374	intergenic_variant	1.00E-11
GCST90179150	2	207390794	CREB1	rs17203016	intron_variant	1.00E-14
GCST90255621	2	207390794	CREB1	rs17203016	intron_variant	1.00E-14
GCST009004	2	207398555	CREB1	rs11692326	intron_variant	2.00E-14
GCST009003	2	207398555	CREB1	rs11692326	intron_variant	2.00E-14
GCST009871	2	46657685	CRIPT	rs113019802	intergenic_variant	5.00E-13
GCST009871	2	46651470	CRIPT	rs17035437	intergenic_variant	4.00E-12
GCST90255621	2	46672603	CRIPT	rs1456014	intergenic_variant	6.00E-11
GCST90179150	2	218414374	CTDSP1 - VIL1	rs7607369	intergenic_variant	7.00E-15
GCST90255621	2	218414374	CTDSP1 - VIL1	rs7607369	intergenic_variant	2.00E-14
GCST90179150	2	79255517	CTNNA2	rs934515	intron_variant	5.00E-12
GCST009004	2	79255517	CTNNA2	rs934515	intron_variant	7.00E-12
GCST90255621	2	79255517	CTNNA2	rs934515	intron_variant	9.00E-11
GCST90054789	2	231995613	DIS3L2	rs73995038	intron_variant	4.00E-16
GCST90054786	2	231995613	DIS3L2	rs73995038	intron_variant	1.00E-15
GCST90255621	2	232117547	DIS3L2	rs11677466	intron_variant	6.00E-11
GCST009871	2	24978002	DNAJC27	rs80078501	intron_variant	2.00E-25
GCST90255621	2	24998571	DNAJC27	rs4303677	intron_variant	1.00E-19
GCST009871	2	24970558	DNAJC27	rs114055620	intron_variant	2.00E-15
GCST007039	2	229760213	DNER	rs62190394	intergenic_variant	7.00E-17

GCST009871	2	229760213	DNER	rs62190394	intergenic_variant	2.00E-16
GCST009871	2	229760668	DNER	rs73099222	intergenic_variant	6.00E-13
GCST009871	2	229680941	DNER	rs7558924	intron_variant	2.00E-12
GCST009871	2	25232520	DNMT3A	rs752208	3_prime_UTR_variant	6.00E-16
GCST90179150	2	25232520	DNMT3A	rs752208	3_prime_UTR_variant	2.00E-15
GCST90255621	2	25265950	DNMT3A	rs7578575	intron_variant	9.00E-14
GCST90255621	2	204511186	DSTNP5 - PARD3B	rs4482463	intergenic_variant	3.00E-24
GCST90179150	2	204511186	DSTNP5 - PARD3B	rs4482463	intergenic_variant	1.00E-22
GCST007039	2	204511186	DSTNP5 - PARD3B	rs4482463	intergenic_variant	1.00E-20
GCST009004	2	204501128	DSTNP5 - PARD3B	rs1470545	intron_variant	6.00E-18
GCST009003	2	204501128	DSTNP5 - PARD3B	rs1470545	intron_variant	6.00E-18
GCST009871	2	204498097	DSTNP5 - PARD3B	rs62179345	intron_variant	3.00E-15
GCST009871	2	204524223	DSTNP5 - PARD3B	rs1868069	intergenic_variant	8.00E-15
GCST90018947	2	204511190	DSTNP5 - PARD3B	rs4405740	intergenic_variant	1.00E-14
GCST90255621	2	171743105	DYNC1I2	rs6738445	intron_variant	3.00E-15
GCST90179150	2	171701662	DYNC1I2	rs2138348	intron_variant	2.00E-12
GCST009871	2	171707412	DYNC1I2	rs312925	intron_variant	3.00E-11
GCST007039	2	171707511	DYNC1I2	rs312924	intron_variant	5.00E-11
GCST009871	2	25150162	EFR3B	rs13401333	intron_variant	7.00E-38
GCST90179150	2	25146133	EFR3B	rs1561288	3_prime_UTR_variant	9.00E-24
GCST90255621	2	25143261	EFR3B	rs4665766	intron_variant	8.00E-22
GCST90179150	2	25157039	EFR3B	rs6734859	3_prime_UTR_variant	8.00E-12
GCST90255621	2	62621184	EHBP1	rs13417156	intron_variant	5.00E-18
GCST009004	2	62621184	EHBP1	rs13417156	intron_variant	7.00E-16
GCST009003	2	62621184	EHBP1	rs13417156	intron_variant	7.00E-16
GCST90179150	2	62825913	EHBP1	rs11688816	intron_variant	5.00E-15
GCST007039	2	62634218	EHBP1	rs6545966	intron_variant	6.00E-12
GCST009871	2	62634218	EHBP1	rs6545966	intron_variant	1.00E-11
GCST90255621	2	212548507	ERBB4	rs7599312	regulatory_region_variant	1.00E-30
GCST009004	2	212548507	ERBB4	rs7599312	regulatory_region_variant	2.00E-23
GCST009003	2	212548507	ERBB4	rs7599312	regulatory_region_variant	2.00E-23
GCST009001	2	212548507	ERBB4	rs7599312	regulatory_region_variant	2.00E-23
GCST90179150	2	212548507	ERBB4	rs7599312	regulatory_region_variant	5.00E-23
GCST007039	2	212549541	ERBB4	rs13427822	regulatory_region_variant	2.00E-16
GCST009871	2	212549541	ERBB4	rs13427822	regulatory_region_variant	8.00E-16
GCST90018947	2	212549541	ERBB4	rs13427822	regulatory_region_variant	4.00E-15
GCST006368	2	212548507	ERBB4	rs7599312	regulatory_region_variant	4.00E-14
GCST90255621	2	211400077	ERBB4	rs4673614	intron_variant	2.00E-13
GCST90018947	2	211414215	ERBB4	rs7591494	intron_variant	3.00E-13
GCST005951	2	212548507	ERBB4	rs7599312	regulatory_region_variant	6.00E-13
GCST009004	2	211431369	ERBB4	rs6435622	intron_variant	4.00E-12
GCST90179150	2	211440116	ERBB4	rs16825008	intron_variant	9.00E-12
GCST004495	2	212548507	ERBB4	rs7599312	regulatory_region_variant	3.00E-11
GCST004904	2	212548507	ERBB4	rs7599312	regulatory_region_variant	3.00E-11
GCST002783	2	212548507	ERBB4	rs7599312	regulatory_region_variant	5.00E-11
GCST90255621	2	417167	FAM150B	rs62106258	intergenic_variant	2.00E-110
GCST009871	2	417167	FAM150B	rs62106258	intergenic_variant	3.00E-100
GCST90271767	2	417167	FAM150B	rs62106258	intergenic_variant	3.00E-98
GCST007039	2	417167	FAM150B	rs62106258	intergenic_variant	2.00E-97
GCST90271770	2	417167	FAM150B	rs62106258	intergenic_variant	2.00E-92
GCST90179150	2	417167	FAM150B	rs62106258	intergenic_variant	5.00E-88

GCST009121	2	466003	FAM150B	rs62104180	intergenic_variant	4.00E-51
GCST009871	2	450234	FAM150B	rs192754985	intergenic_variant	1.00E-35
GCST90255621	2	437664	FAM150B	rs2685230	intergenic_variant	3.00E-32
GCST009871	2	434668	FAM150B	rs12475496	intergenic_variant	2.00E-22
GCST90179150	2	437664	FAM150B	rs2685230	intergenic_variant	6.00E-22
GCST009871	2	409112	FAM150B	rs7582535	intron_variant	1.00E-21
GCST90255621	2	454985	FAM150B	rs184991221	regulatory_region_variant	1.00E-20
GCST90267268	2	375873	FAM150B	rs74495980	regulatory_region_variant	2.00E-20
GCST90255621	2	348177	FAM150B	rs13416560	regulatory_region_variant	2.00E-18
GCST009871	2	407713	FAM150B	rs75960650	intron_variant	3.00E-16
GCST007240	2	422144	FAM150B	rs62107261	non_coding_transcript_exon_	1.00E-15
GCST90255621	2	477100	FAM150B	rs2724862	intergenic_variant	9.00E-14
GCST90179150	2	355800	FAM150B	rs6740249	intergenic_variant	4.00E-13
GCST009871	2	350616	FAM150B	rs10185853	intergenic_variant	4.00E-12
GCST009107	2	466003	FAM150B	rs62104180	intergenic_variant	1.00E-11
GCST90255621	2	58708147	FANCL	rs4671328	intron_variant	3.00E-48
GCST90255621	2	59075742	FANCL	rs887912	intron_variant	3.00E-42
GCST009004	2	58708147	FANCL	rs4671328	intron_variant	3.00E-36
GCST009001	2	58708147	FANCL	rs4671328	intron_variant	3.00E-36
GCST90179150	2	58708147	FANCL	rs4671328	intron_variant	1.00E-34
GCST009003	2	58665930	FANCL	rs1861412	intron_variant	1.00E-32
GCST009004	2	59080590	FANCL	rs6545714	intron_variant	4.00E-32
GCST009003	2	59078490	FANCL	rs1016287	intron_variant	9.00E-32
GCST90179150	2	59080590	FANCL	rs6545714	intron_variant	1.00E-31
GCST009001	2	59075742	FANCL	rs887912	intron_variant	2.00E-30
GCST009871	2	58708147	FANCL	rs4671328	intron_variant	2.00E-28
GCST007039	2	58708147	FANCL	rs4671328	intron_variant	1.00E-27
GCST90018947	2	58706456	FANCL	rs1861410	intron_variant	2.00E-26
GCST007039	2	59067423	FANCL	rs10172678	intron_variant	9.00E-25
GCST009871	2	59067423	FANCL	rs10172678	intron_variant	4.00E-24
GCST90255621	2	58799087	FANCL	rs17049738	intron_variant	6.00E-21
GCST90255621	2	58601296	FANCL	rs12612428	intron_variant	1.00E-19
GCST009871	2	58590310	FANCL	rs6744844	intron_variant	3.00E-19
GCST90255621	2	58591335	FANCL	rs7571367	intron_variant	2.00E-18
GCST006368	2	58708147	FANCL	rs4671328	intron_variant	5.00E-18
GCST009121	2	58738076	FANCL	rs1641155	intron_variant	4.00E-17
GCST90179150	2	58799087	FANCL	rs17049738	intron_variant	5.00E-16
GCST90179150	2	58601296	FANCL	rs12612428	intron_variant	1.00E-15
GCST004904	2	58708147	FANCL	rs4671328	intron_variant	2.00E-15
GCST009871	2	58812863	FANCL	rs12617233	intron_variant	2.00E-15
GCST005951	2	58630284	FANCL	rs13011109	intron_variant	1.00E-14
GCST006368	2	58565242	FANCL	rs929641	intron_variant	3.00E-14
GCST90255621	2	58814297	FANCL	rs2862733	intron_variant	3.00E-14
GCST90255621	2	58912372	FANCL	rs17049820	intron_variant	1.00E-13
GCST006368	2	59075742	FANCL	rs887912	intron_variant	1.00E-13
GCST005951	2	59078490	FANCL	rs1016287	intron_variant	4.00E-13
GCST009871	2	58756990	FANCL	rs2058625	intron_variant	6.00E-13
GCST90018727	2	58756990	FANCL	rs2058625	intron_variant	1.00E-12
GCST90255621	2	58923821	FANCL	rs12328778	intron_variant	1.00E-12
GCST009871	2	58601296	FANCL	rs12612428	intron_variant	2.00E-12
GCST009871	2	58620301	FANCL	rs11892323	intron_variant	2.00E-12

GCST90179150	2	58912372	FANCL	rs17049820	intron_variant	2.00E-12
GCST000830	2	59075742	FANCL	rs887912	intron_variant	2.00E-12
GCST002783	2	59078490	FANCL	rs1016287	intron_variant	4.00E-12
GCST90255621	2	58838673	FANCL	rs7593324	intron_variant	9.00E-12
GCST90267268	2	58534464	FANCL	rs764975	intron_variant	1.00E-11
GCST90267268	2	58799252	FANCL	rs6545700	intron_variant	1.00E-11
GCST90255621	2	58906378	FANCL	rs1861100	intron_variant	1.00E-11
GCST002783	2	59078490	FANCL	rs1016287	intron_variant	2.00E-11
GCST009871	2	58619554	FANCL	rs77527549	intron_variant	3.00E-11
GCST90179150	2	58906378	FANCL	rs1861100	intron_variant	4.00E-11
GCST009871	2	58773468	FANCL	rs75786554	intron_variant	5.00E-11
GCST90179150	2	58814297	FANCL	rs2862733	intron_variant	8.00E-11
GCST90179150	2	58957977	FANCL	rs13002470	intron_variant	9.00E-11
GCST90179150	2	163724731	FIGN	rs10192119	intron_variant	1.00E-12
GCST009004	2	163711179	FIGN	rs1460676	intron_variant	2.00E-12
GCST009003	2	163711179	FIGN	rs1460676	intron_variant	2.00E-12
GCST90255621	2	163724731	FIGN	rs10192119	intron_variant	1.00E-11
GCST011334	2	27508073	GCKR	rs1260326	missense_variant	2.00E-262
GCST004046	2	27518370	GCKR	rs780094	intron_variant	8.00E-101
GCST004045	2	27518370	GCKR	rs780094	intron_variant	4.00E-91
GCST001527	2	27518370	GCKR	rs780094	intron_variant	4.00E-24
GCST90179150	2	27508073	GCKR	rs1260326	missense_variant	8.00E-19
GCST90255621	2	164572725	GRB14	rs11677541	intron_variant	4.00E-11
GCST90255621	2	164409156	GRB14	rs569675	intergenic_variant	6.00E-11
GCST90255621	2	48727766	GTF2A1L,LHCGR	rs7561278	intron_variant	2.00E-18
GCST009004	2	48727766	GTF2A1L,LHCGR	rs7561278	intron_variant	5.00E-16
GCST90179150	2	48727766	GTF2A1L,LHCGR	rs7561278	intron_variant	2.00E-15
GCST007039	2	48727766	GTF2A1L,LHCGR	rs7561278	intron_variant	1.00E-13
GCST009871	2	48727766	GTF2A1L,LHCGR	rs7561278	intron_variant	2.00E-13
GCST009003	2	48726840	GTF2A1L,LHCGR	rs72618637	intron_variant	2.00E-13
GCST90018947	2	48726840	GTF2A1L,LHCGR	rs72618637	intron_variant	8.00E-13
GCST006368	2	41446605	HNRNPA1P57 - LDHAP3	rs1396141	intergenic_variant	7.00E-13
GCST009004	2	41485709	HNRNPA1P57 - LDHAP3	rs2063177	intergenic_variant	2.00E-11
GCST90255621	2	41410548	HNRNPA1P57 - LDHAP3	rs10169594	intergenic_variant	2.00E-11
GCST90179150	2	41476243	HNRNPA1P57 - LDHAP3	rs12712767	regulatory_region_variant	6.00E-11
GCST90255621	2	26717426	KCNK3	rs12468863	intron_variant	3.00E-21
GCST90179150	2	26717426	KCNK3	rs12468863	intron_variant	2.00E-20
GCST007039	2	26730714	KCNK3	rs1731259	3_prime_UTR_variant	3.00E-19
GCST90018947	2	26718617	KCNK3	rs397934918	intron_variant	1.00E-16
GCST004904	2	26705943	KCNK3	rs11126666	intron_variant	1.00E-13
GCST006368	2	26705943	KCNK3	rs11126666	intron_variant	5.00E-11
GCST90255621	2	207090753	KLF7	rs1271272	intron_variant	3.00E-13
GCST009871	2	207072187	KLF7	rs1263629	regulatory_region_variant	5.00E-12
GCST007039	2	207072187	KLF7	rs1263629	regulatory_region_variant	1.00E-11
GCST009004	2	207112529	KLF7	rs1263627	intron_variant	7.00E-11
GCST007039	2	147146234	LINC01911 - RNU6-692P	rs778094	intergenic_variant	1.00E-19
GCST009871	2	147090322	LINC01911 - RNU6-692P	rs2381977	intergenic_variant	6.00E-19
GCST009004	2	147143639	LINC01911 - RNU6-692P	rs1451077	intergenic_variant	1.00E-18
GCST009001	2	147143639	LINC01911 - RNU6-692P	rs1451077	intergenic_variant	1.00E-18
GCST90179150	2	147143639	LINC01911 - RNU6-692P	rs1451077	intergenic_variant	3.00E-18
GCST009003	2	147090322	LINC01911 - RNU6-692P	rs2381977	intergenic_variant	2.00E-17

GCST90255621	2	147149634	LINC01911 - RNU6-692P	rs453520	intergenic_variant	8.00E-16
GCST90018947	2	147133709	LINC01911 - RNU6-692P	rs778100	regulatory_region_variant	3.00E-12
GCST90255621	2	67610421	LINC02831	rs2861685	intron_variant	3.00E-24
GCST009871	2	67611553	LINC02831	rs2861690	intron_variant	8.00E-20
GCST007039	2	67610421	LINC02831	rs2861685	intron_variant	1.00E-19
GCST009003	2	67610421	LINC02831	rs2861685	intron_variant	8.00E-18
GCST009004	2	67610421	LINC02831	rs2861685	intron_variant	8.00E-18
GCST90179150	2	67610421	LINC02831	rs2861685	intron_variant	5.00E-17
GCST009001	2	67638833	LINC02831	rs10196304	intron_variant	4.00E-16
GCST90018947	2	67638833	LINC02831	rs10196304	intron_variant	9.00E-14
GCST90255621	2	100299310	LONRF2	rs4851287	missense_variant	3.00E-22
GCST007039	2	100276651	LONRF2	rs6542924	3_prime_UTR_variant	3.00E-18
GCST009871	2	100278425	LONRF2	rs4851284	3_prime_UTR_variant	2.00E-17
GCST008129	2	100299310	LONRF2	rs4851287	missense_variant	2.00E-12
GCST90255621	2	141535577	LRP1B	rs17551974	intron_variant	5.00E-13
GCST90179150	2	142202362	LRP1B	rs2890652	intergenic_variant	1.00E-11
GCST005951	2	142202362	LRP1B	rs2890652	intergenic_variant	2.00E-11
GCST009004	2	142074189	LRP1B	rs13396415	intron_variant	5.00E-11
GCST90255621	2	141810341	LRP1B	rs12990449	missense_variant	6.00E-11
GCST009871	2	174301908	LRRC2P1 - HNRNPA1P39	rs34234296	intergenic_variant	8.00E-17
GCST007039	2	174301908	LRRC2P1 - HNRNPA1P39	rs34234296	intergenic_variant	8.00E-17
GCST90255621	2	174301908	LRRC2P1 - HNRNPA1P39	rs34234296	intergenic_variant	2.00E-15
GCST90179150	2	174301908	LRRC2P1 - HNRNPA1P39	rs34234296	intergenic_variant	1.00E-12
GCST90271767	2	33207791	LTBP1	rs4671010	intron_variant	4.00E-12
GCST90271770	2	33207791	LTBP1	rs4671010	intron_variant	9.00E-12
GCST90179150	2	135851076	MCM6	rs4988235	intron_variant	8.00E-13
GCST011335	2	135851076	MCM6	rs4988235	intron_variant	2.00E-12
GCST009004	2	172025679	METAP1D	rs10930502	intron_variant	3.00E-13
GCST009003	2	172025679	METAP1D	rs10930502	intron_variant	3.00E-13
GCST90255621	2	60438940	MIR4432HG	rs1011406	intron_variant	2.00E-11
GCST009004	2	60438633	MIR4432HG	rs1011407	intron_variant	7.00E-11
GCST90179150	2	60438633	MIR4432HG	rs1011407	intron_variant	9.00E-11
GCST90255621	2	104947387	MRPS9-AS2	rs6722268	intron_variant	9.00E-13
GCST90179150	2	104974032	MRPS9-AS2	rs13395380	intron_variant	1.00E-12
GCST009871	2	104974455	MRPS9-AS2	rs72832230	intron_variant	1.00E-11
GCST90255621	2	203141914	NBEAL1	rs6751448	intron_variant	1.00E-14
GCST90179150	2	203105560	NBEAL1	rs10497870	intron_variant	6.00E-14
GCST009004	2	203105560	NBEAL1	rs10497870	intron_variant	2.00E-13
GCST009001	2	203099414	NBEAL1	rs7574314	non_coding_transcript_exon_	4.00E-12
GCST009004	2	132766032	NCKAP5	rs13033310	intron_variant	3.00E-11
GCST90179150	2	132766032	NCKAP5	rs13033310	intron_variant	4.00E-11
GCST009871	2	24767213	NCOA1	rs11892043	intron_variant	3.00E-24
GCST009871	2	24475029	NCOA1	rs76066823	intergenic_variant	2.00E-11
GCST90255621	2	49973972	NRXN1	rs9636391	stop_gained	2.00E-20
GCST009004	2	50006214	NRXN1	rs930295	intron_variant	2.00E-19
GCST009003	2	49973972	NRXN1	rs9636391	stop_gained	7.00E-19
GCST90179150	2	50006214	NRXN1	rs930295	intron_variant	8.00E-19
GCST007039	2	49973972	NRXN1	rs9636391	stop_gained	8.00E-17
GCST009871	2	49990857	NRXN1	rs1421567	intron_variant	2.00E-16
GCST90255621	2	50486193	NRXN1	rs6724631	intron_variant	1.00E-15
GCST90179150	2	50465030	NRXN1	rs1520455	intron_variant	3.00E-15

GCST90255621	2	50075840	NRXN1	rs4971552	intron_variant	6.00E-13
GCST009871	2	50465030	NRXN1	rs1520455	intron_variant	1.00E-12
GCST90018947	2	50896545	NRXN1	rs1544690	intron_variant	4.00E-12
GCST009871	2	50070067	NRXN1	rs13401630	intron_variant	2.00E-11
GCST90255621	2	50632045	NRXN1	rs10172718	intron_variant	4.00E-11
GCST011336	2	226243730	NYAP2	rs2943652	intergenic_variant	7.00E-20
GCST90255621	2	226203364	NYAP2	rs2943634	intergenic_variant	3.00E-19
GCST011334	2	226243730	NYAP2	rs2943652	intergenic_variant	1.00E-16
GCST001526	2	226203364	NYAP2	rs2943634	intergenic_variant	2.00E-14
GCST011329	2	226243730	NYAP2	rs2943652	intergenic_variant	1.00E-12
GCST003658	2	226146604	NYAP2	rs13422522	intergenic_variant	1.00E-11
GCST003659	2	226146604	NYAP2	rs13422522	intergenic_variant	9.00E-11
GCST90255621	2	174096760	OLA1	rs2044469	intron_variant	9.00E-15
GCST009004	2	174096760	OLA1	rs2044469	intron_variant	1.00E-13
GCST009003	2	174152986	OLA1	rs2119137	intron_variant	2.00E-13
GCST90179150	2	174096760	OLA1	rs2044469	intron_variant	8.00E-13
GCST009871	2	174071453	OLA1	rs2884442	intergenic_variant	2.00E-12
GCST90018947	2	174104165	OLA1	rs397765214	intron_variant	8.00E-11
GCST90255621	2	104838132	PANTR1	rs10197031	intron_variant	2.00E-23
GCST009004	2	104838132	PANTR1	rs10197031	intron_variant	5.00E-18
GCST009001	2	104838132	PANTR1	rs10197031	intron_variant	5.00E-18
GCST009003	2	104835868	PANTR1	rs6739199	intron_variant	1.00E-17
GCST90179150	2	104835868	PANTR1	rs6739199	intron_variant	4.00E-17
GCST007039	2	104838132	PANTR1	rs10197031	intron_variant	1.00E-16
GCST009871	2	104838132	PANTR1	rs10197031	intron_variant	4.00E-16
GCST90018947	2	104798021	PANTR1	rs6754968	intron_variant	5.00E-13
GCST90255621	2	198085516	PLCL1	rs1064213	missense_variant	1.00E-23
GCST90018947	2	198079547	PLCL1	rs6716898	intron_variant	1.00E-17
GCST007039	2	198085516	PLCL1	rs1064213	missense_variant	9.00E-14
GCST009871	2	198033994	PLCL1	rs17520121	intron_variant	2.00E-13
GCST009871	2	198085516	PLCL1	rs1064213	missense_variant	3.00E-13
GCST004046	2	27412596	PPM1G	rs1728918	regulatory_region_variant	5.00E-35
GCST004045	2	27412596	PPM1G	rs1728918	regulatory_region_variant	1.00E-31
GCST90255621	2	28751017	PPP1CB	rs4372836	non_coding_transcript_exon_	4.00E-18
GCST90179150	2	28751017	PPP1CB	rs4372836	non_coding_transcript_exon_	2.00E-13
GCST009004	2	28751017	PPP1CB	rs4372836	non_coding_transcript_exon_	7.00E-13
GCST009003	2	28773429	PPP1CB	rs7579277	intron_variant	4.00E-12
GCST009871	2	28755208	PPP1CB	rs6547872	intron_variant	2.00E-11
GCST007039	2	28755208	PPP1CB	rs6547872	intron_variant	7.00E-11
GCST007039	2	219296584	PTPRN	rs2292604	intron_variant	3.00E-13
GCST009871	2	219291809	PTPRN	rs11886864	non_coding_transcript_exon_	2.00E-11
GCST009004	2	160409399	RBMS1	rs12692596	intron_variant	1.00E-12
GCST009003	2	160409399	RBMS1	rs12692596	intron_variant	1.00E-12
GCST90179150	2	160409399	RBMS1	rs12692596	intron_variant	3.00E-12
GCST90255621	2	160425180	RBMS1	rs4664325	intron_variant	2.00E-11
GCST90255621	2	219340424	RESP18	rs6725931	intergenic_variant	3.00E-16
GCST90179150	2	219333525	RESP18	rs908194	intergenic_variant	2.00E-15
GCST90179150	2	197652868	RFTN2	rs7603283	intron_variant	9.00E-13
GCST009004	2	197653985	RFTN2	rs6733834	intron_variant	2.00E-12
GCST90255621	2	60057965	RNA5SP94 - MIR4432HG	rs980329	intron_variant	2.00E-16
GCST90255621	2	59976782	RNA5SP94 - MIR4432HG	rs12476772	intron_variant	2.00E-14

GCST007039	2	59976782	RNA5SP94 - MIR4432HG	rs12476772	intron_variant	1.00E-13
GCST009871	2	59976782	RNA5SP94 - MIR4432HG	rs12476772	intron_variant	2.00E-13
GCST90179150	2	60057479	RNA5SP94 - MIR4432HG	rs1861151	intron_variant	2.00E-12
GCST90179150	2	59976782	RNA5SP94 - MIR4432HG	rs12476772	intron_variant	2.00E-11
GCST90255621	2	55054765	RTN4	rs7601895	intron_variant	7.00E-19
GCST009004	2	55054765	RTN4	rs7601895	intron_variant	2.00E-16
GCST009871	2	55054765	RTN4	rs7601895	intron_variant	5.00E-16
GCST007039	2	55054765	RTN4	rs7601895	intron_variant	7.00E-15
GCST90179150	2	55052400	RTN4	rs6760379	intron_variant	6.00E-14
GCST009003	2	55059545	RTN4	rs7582359	intron_variant	2.00E-12
GCST90018947	2	55055790	RTN4	rs6757907	intron_variant	2.00E-12
GCST90255621	2	180742168	SCHLAP1	rs11679338	intron_variant	9.00E-28
GCST009004	2	180710554	SCHLAP1	rs7588437	intron_variant	2.00E-22
GCST009003	2	180710554	SCHLAP1	rs7588437	intron_variant	2.00E-22
GCST009001	2	180737799	SCHLAP1	rs1019612	intron_variant	1.00E-21
GCST90179150	2	180710554	SCHLAP1	rs7588437	intron_variant	2.00E-21
GCST90018947	2	180705780	SCHLAP1	rs12479357	intron_variant	2.00E-18
GCST009871	2	180742949	SCHLAP1	rs9630985	intron_variant	3.00E-18
GCST007039	2	180737799	SCHLAP1	rs1019612	intron_variant	2.00E-17
GCST009871	2	180637211	SCHLAP1	rs2368178	intron_variant	4.00E-17
GCST90255621	2	180575354	SCHLAP1	rs1358520	intron_variant	8.00E-15
GCST004904	2	180686235	SCHLAP1	rs1528435	intron_variant	2.00E-13
GCST90255621	2	180601126	SCHLAP1	rs6739798	intron_variant	3.00E-12
GCST90179150	2	180649002	SCHLAP1	rs918959	intron_variant	8.00E-12
GCST006368	2	180742168	SCHLAP1	rs11679338	intron_variant	2.00E-11
GCST005951	2	180710554	SCHLAP1	rs7588437	intron_variant	2.00E-11
GCST009004	2	165291814	SCN2A	rs61051952	intron_variant	7.00E-12
GCST90179150	2	165288340	SCN2A	rs12477385	intron_variant	8.00E-12
GCST90255621	2	165329197	SCN2A	rs3754963	intron_variant	3.00E-11
GCST009871	2	40055062	SLC8A1	rs10185199	intron_variant	2.00E-12
GCST90179150	2	40055062	SLC8A1	rs10185199	intron_variant	2.00E-12
GCST007039	2	40055062	SLC8A1	rs10185199	intron_variant	3.00E-12
GCST90018947	2	40065532	SLC8A1	rs13403962	non_coding_transcript_exon_	3.00E-12
GCST90255621	2	40299988	SLC8A1	rs727477	intron_variant	6.00E-12
GCST90255621	2	40064800	SLC8A1	rs6713781	intron_variant	1.00E-11
GCST90255621	2	35287117	SMIM7P1	rs17327461	intergenic_variant	4.00E-14
GCST90179150	2	35287117	SMIM7P1	rs17327461	intergenic_variant	9.00E-14
GCST009004	2	35184631	SMIM7P1	rs1561554	intron_variant	5.00E-11
GCST90255621	2	6015425	SOX11	rs10929925	intergenic_variant	4.00E-22
GCST90179150	2	6015425	SOX11	rs10929925	intergenic_variant	2.00E-18
GCST009004	2	6015425	SOX11	rs10929925	intergenic_variant	3.00E-18
GCST009001	2	6015425	SOX11	rs10929925	intergenic_variant	3.00E-18
GCST009003	2	6019849	SOX11	rs12468070	intergenic_variant	5.00E-16
GCST004495	2	6015425	SOX11	rs10929925	intergenic_variant	2.00E-13
GCST007039	2	6004949	SOX11	rs2091378	intergenic_variant	2.00E-13
GCST009871	2	6004949	SOX11	rs2091378	intergenic_variant	3.00E-13
GCST004497	2	6015425	SOX11	rs10929925	intergenic_variant	1.00E-12
GCST004499	2	6015425	SOX11	rs10929925	intergenic_variant	2.00E-12
GCST90255621	2	228112512	SPHKAP	rs4455149	intron_variant	1.00E-18
GCST007039	2	228120789	SPHKAP	rs4500930	intron_variant	1.00E-17
GCST009871	2	228146244	SPHKAP	rs4605363	intron_variant	3.00E-17

GCST009004	2	228137904	SPHKAP	rs4973618	intron_variant	1.00E-16
GCST90179150	2	228118782	SPHKAP	rs12476312	intron_variant	7.00E-16
GCST90255621	2	228072280	SPHKAP	rs4533465	intron_variant	3.00E-14
GCST009003	2	228134975	SPHKAP	rs6436755	intron_variant	3.00E-13
GCST009871	2	228055251	SPHKAP	rs62201071	intron_variant	1.00E-11
GCST004046	2	21176344	TDRD15	rs478442	regulatory_region_variant	8.00E-21
GCST004045	2	21176344	TDRD15	rs478442	regulatory_region_variant	4.00E-16
GCST90255621	2	634905	TMEM18	rs6548238	TF_binding_site_variant	3.00E-214
GCST90271770	2	632348	TMEM18	rs13021737	intergenic_variant	1.00E-169
GCST90179150	2	632348	TMEM18	rs13021737	intergenic_variant	2.00E-163
GCST009004	2	632348	TMEM18	rs13021737	intergenic_variant	3.00E-161
GCST009003	2	621461	TMEM18	rs6548237	intergenic_variant	2.00E-158
GCST009001	2	651430	TMEM18	rs12714415	regulatory_region_variant	1.00E-157
GCST007039	2	628504	TMEM18	rs6744646	intergenic_variant	4.00E-111
GCST009871	2	628504	TMEM18	rs6744646	intergenic_variant	1.00E-110
GCST90271769	2	629510	TMEM18	rs6743060	intergenic_variant	1.00E-94
GCST90018947	2	651430	TMEM18	rs12714415	regulatory_region_variant	2.00E-81
GCST005950	2	622827	TMEM18	rs2867125	intergenic_variant	5.00E-75
GCST005951	2	630323	TMEM18	rs6725549	intergenic_variant	1.00E-74
GCST005951	2	622827	TMEM18	rs2867125	intergenic_variant	1.00E-72
GCST006368	2	632348	TMEM18	rs13021737	intergenic_variant	4.00E-69
GCST009121	2	635864	TMEM18	rs6751993	intergenic_variant	3.00E-65
GCST004904	2	632348	TMEM18	rs13021737	intergenic_variant	4.00E-65
GCST002783	2	632348	TMEM18	rs13021737	intergenic_variant	5.00E-54
GCST004558	2	622827	TMEM18	rs2867125	intergenic_variant	5.00E-52
GCST002783	2	632348	TMEM18	rs13021737	intergenic_variant	1.00E-50
GCST004557	2	622827	TMEM18	rs2867125	intergenic_variant	1.00E-49
GCST000830	2	622827	TMEM18	rs2867125	intergenic_variant	3.00E-49
GCST005953	2	622827	TMEM18	rs2867125	intergenic_variant	6.00E-49
GCST004558	2	622827	TMEM18	rs2867125	intergenic_variant	9.00E-48
GCST004557	2	622827	TMEM18	rs2867125	intergenic_variant	5.00E-45
GCST004495	2	632348	TMEM18	rs13021737	intergenic_variant	9.00E-41
GCST90271560	2	651105	TMEM18	rs2867110	regulatory_region_variant	3.00E-40
GCST002783	2	632348	TMEM18	rs13021737	intergenic_variant	7.00E-40
GCST004497	2	632348	TMEM18	rs13021737	intergenic_variant	8.00E-40
GCST90255621	2	677591	TMEM18	rs11893030	intron_variant	4.00E-37
GCST004559	2	622827	TMEM18	rs2867125	intergenic_variant	7.00E-36
GCST004558	2	622827	TMEM18	rs2867125	intergenic_variant	7.00E-35
GCST007241	2	630070	TMEM18	rs66906321	intergenic_variant	1.00E-34
GCST004559	2	622827	TMEM18	rs2867125	intergenic_variant	1.00E-33
GCST90255621	2	621006	TMEM18	rs6710140	intergenic_variant	3.00E-33
GCST004557	2	622827	TMEM18	rs2867125	intergenic_variant	4.00E-33
GCST004558	2	622827	TMEM18	rs2867125	intergenic_variant	5.00E-32
GCST004557	2	622827	TMEM18	rs2867125	intergenic_variant	1.00E-31
GCST90255621	2	554109	TMEM18	rs13411762	regulatory_region_variant	4.00E-31
GCST009871	2	677591	TMEM18	rs11893030	intron_variant	7.00E-31
GCST005952	2	622827	TMEM18	rs2867125	intergenic_variant	2.00E-30
GCST004499	2	632348	TMEM18	rs13021737	intergenic_variant	3.00E-30
GCST90179150	2	663483	TMEM18	rs12999373	intergenic_variant	2.00E-29
GCST004519	2	633660	TMEM18	rs62105306	intergenic_variant	3.00E-28
GCST004495	2	632348	TMEM18	rs13021737	intergenic_variant	3.00E-28

GCST004497	2	632348	TMEM18	rs13021737	intergenic_variant	3.00E-28
GCST009871	2	621115	TMEM18	rs10180960	intergenic_variant	4.00E-28
GCST009871	2	619641	TMEM18	rs10153796	intergenic_variant	3.00E-27
GCST004559	2	622827	TMEM18	rs2867125	intergenic_variant	1.00E-26
GCST006802	2	632348	TMEM18	rs13021737	intergenic_variant	2.00E-26
GCST90179150	2	554109	TMEM18	rs13411762	regulatory_region_variant	6.00E-26
GCST90002409	2	621558	TMEM18	rs939584	intergenic_variant	9.00E-26
GCST90179150	2	621006	TMEM18	rs6710140	intergenic_variant	1.00E-25
GCST009871	2	663483	TMEM18	rs12999373	intergenic_variant	2.00E-25
GCST004559	2	622827	TMEM18	rs2867125	intergenic_variant	2.00E-24
GCST004499	2	632348	TMEM18	rs13021737	intergenic_variant	2.00E-23
GCST004904	2	621558	TMEM18	rs939584	intergenic_variant	6.00E-23
GCST004558	2	638144	TMEM18	rs4854344	regulatory_region_variant	9.00E-23
GCST004557	2	638144	TMEM18	rs4854344	regulatory_region_variant	2.00E-22
GCST002783	2	632348	TMEM18	rs13021737	intergenic_variant	4.00E-22
GCST003177	2	647861	TMEM18	rs4854349	intergenic_variant	5.00E-22
GCST007240	2	629601	TMEM18	rs6748821	intergenic_variant	8.00E-21
GCST90018727	2	625057	TMEM18	rs7576635	intergenic_variant	9.00E-21
GCST009871	2	621006	TMEM18	rs6710140	intergenic_variant	6.00E-20
GCST004560	2	638144	TMEM18	rs4854344	regulatory_region_variant	2.00E-19
GCST009764	2	631759	TMEM18	rs10865549	intergenic_variant	3.00E-19
GCST004557	2	622827	TMEM18	rs2867125	intergenic_variant	5.00E-19
GCST000298	2	634905	TMEM18	rs6548238	TF_binding_site_variant	1.00E-18
GCST009107	2	635864	TMEM18	rs6751993	intergenic_variant	4.00E-18
GCST003177	2	632348	TMEM18	rs13021737	intergenic_variant	5.00E-18
GCST009871	2	557123	TMEM18	rs10192669	intergenic_variant	9.00E-18
GCST002021	2	629244	TMEM18	rs12463617	intergenic_variant	3.00E-17
GCST004495	2	632348	TMEM18	rs13021737	intergenic_variant	3.00E-17
GCST000296	2	644953	TMEM18	rs7561317	intergenic_variant	4.00E-17
GCST004560	2	622827	TMEM18	rs2867125	intergenic_variant	7.00E-17
GCST90131907	2	623216	TMEM18	rs4854340	intergenic_variant	4.00E-16
GCST90239604	2	623216	TMEM18	rs4854340	intergenic_variant	7.00E-16
GCST004497	2	632348	TMEM18	rs13021737	intergenic_variant	1.00E-15
GCST90255622	2	631183	TMEM18	rs7579427	intergenic_variant	1.00E-15
GCST90179150	2	688856	TMEM18	rs75275918	intron_variant	2.00E-15
GCST008158	2	622225	TMEM18	rs1320330	intergenic_variant	4.00E-15
GCST001955	2	624678	TMEM18	rs2903492	intergenic_variant	6.00E-15
GCST009871	2	597626	TMEM18	rs10208700	intergenic_variant	1.00E-14
GCST004559	2	622827	TMEM18	rs2867125	intergenic_variant	2.00E-14
GCST004558	2	622827	TMEM18	rs2867125	intergenic_variant	2.00E-14
GCST009871	2	678386	TMEM18	rs11127493	intron_variant	7.00E-14
GCST90255621	2	674963	TMEM18	rs12990777	intron_variant	2.00E-13
GCST90267268	2	666466	TMEM18	rs76847828	3_prime_UTR_variant	6.00E-13
GCST008025	2	622827	TMEM18	rs2867125	intergenic_variant	2.00E-12
GCST008025	2	624034	TMEM18	rs6711012	intergenic_variant	2.00E-12
GCST002461	2	629244	TMEM18	rs12463617	intergenic_variant	2.00E-12
GCST004498	2	632348	TMEM18	rs13021737	intergenic_variant	2.00E-12
GCST008025	2	624678	TMEM18	rs2903492	intergenic_variant	3.00E-12
GCST90179150	2	710539	TMEM18	rs10208465	intergenic_variant	3.00E-12
GCST008025	2	622531	TMEM18	rs939583	intergenic_variant	5.00E-12
GCST90179150	2	597626	TMEM18	rs10208700	intergenic_variant	6.00E-12

GCST008025	2	632028	TMEM18	rs11127485	intergenic_variant	7.00E-12
GCST008025	2	632348	TMEM18	rs13021737	intergenic_variant	7.00E-12
GCST008025	2	634905	TMEM18	rs6548238	TF_binding_site_variant	8.00E-12
GCST004499	2	632348	TMEM18	rs13021737	intergenic_variant	8.00E-12
GCST004560	2	638144	TMEM18	rs4854344	regulatory_region_variant	2.00E-11
GCST009871	2	598782	TMEM18	rs10495481	intergenic_variant	3.00E-11
GCST004560	2	622827	TMEM18	rs2867125	intergenic_variant	7.00E-11
GCST009871	2	674963	TMEM18	rs12990777	intron_variant	9.00E-11
GCST009004	2	229798860	TRIP12	rs6720868	intron_variant	2.00E-17
GCST009003	2	229798860	TRIP12	rs6720868	intron_variant	2.00E-17
GCST90179150	2	229798860	TRIP12	rs6720868	intron_variant	5.00E-17
GCST90255621	2	229852946	TRIP12	rs10933311	intron_variant	6.00E-16
GCST90018947	2	229840801	TRIP12	rs34481385	intron_variant	4.00E-14
GCST90179150	2	229764422	TRIP12	rs1044822	3_prime_UTR_variant	7.00E-12
GCST009004	2	218722568	TTLL4	rs1541777	intron_variant	2.00E-13
GCST009001	2	218722568	TTLL4	rs1541777	intron_variant	2.00E-13
GCST90255621	2	206380059	ZDBF2 - ACER2P1	rs972540	intergenic_variant	5.00E-16
GCST009004	2	206380059	ZDBF2 - ACER2P1	rs972540	intergenic_variant	4.00E-13
GCST90179150	2	206380059	ZDBF2 - ACER2P1	rs972540	intergenic_variant	7.00E-13
GCST009001	2	206368391	ZDBF2 - ACER2P1	rs10497882	intergenic_variant	2.00E-12
GCST007039	3	88205670	AARS1	rs4858940	intergenic_variant	2.00E-16
GCST009871	3	88205670	ABCF2P1	rs4858940	intergenic_variant	1.00E-15
GCST90255621	3	43860678	ABHD17C	rs9824340	intergenic_variant	3.00E-16
GCST90255621	3	44004489	ABHD5	rs3852012	intergenic_variant	2.00E-15
GCST90271772	3	44007622	ABHD5	rs6441814	intergenic_variant	3.00E-15
GCST90179150	3	43998397	ABHD5	rs2302351	non_coding_transcript_exon_	1.00E-11
GCST007039	3	44002852	ABHD5	rs1554654	intergenic_variant	5.00E-11
GCST011329	3	64716599	ACTG1P22	rs4616635	intron_variant	2.00E-12
GCST90255621	3	64732582	ADAMTS9	rs2371767	intron_variant	3.00E-11
GCST90255621	3	123374694	ADCY3	rs2124499	intron_variant	4.00E-16
GCST90104629	3	123346931	ADCY5	rs11708067	intron_variant	5.00E-16
GCST011329	3	123346931	ADCY5	rs11708067	intron_variant	5.00E-12
GCST90255621	3	123346931	ADCY5	rs11708067	intron_variant	1.00E-11
GCST90255621	3	35625565	ARL15	rs13062093	intergenic_variant	7.00E-14
GCST009004	3	35625565	ARPP21	rs13062093	intergenic_variant	3.00E-12
GCST90179150	3	35625565	ARPP21	rs13062093	intergenic_variant	4.00E-12
GCST009871	3	35641612	ARPP21	rs9860326	5_prime_UTR_variant	9.00E-12
GCST007039	3	35641612	ARPP21	rs9860326	5_prime_UTR_variant	1.00E-11
GCST009871	3	35510735	ARPP21	rs17033321	intergenic_variant	8.00E-11
GCST90255621	3	53743149	CACNA1D	rs2680648	intron_variant	3.00E-16
GCST90179150	3	53743149	CACNA1D	rs2680648	intron_variant	5.00E-15
GCST009004	3	53765933	CACNA1D	rs3774573	non_coding_transcript_exon_	3.00E-14
GCST009003	3	53765933	CACNA1D	rs3774573	non_coding_transcript_exon_	3.00E-14
GCST007039	3	53755145	CACNA1D	rs719260	intron_variant	3.00E-11
GCST009871	3	53765933	CACNA1D	rs3774573	non_coding_transcript_exon_	4.00E-11
GCST005951	3	53713347	CACNA1D	rs13076366	intron_variant	5.00E-11
GCST009871	3	50385287	CACNA2D2	rs2236952	intron_variant	8.00E-12
GCST009871	3	50412873	CACNA2D2	rs80094684	intron_variant	1.00E-11
GCST90255621	3	85817185	CADM2	rs2122042	intron_variant	1.00E-34
GCST009004	3	85811914	CADM2	rs9818122	intron_variant	4.00E-30
GCST90179150	3	85758440	CADM2	rs13078960	intron_variant	1.00E-29

GCST009003	3	85771031	CADM2	rs13098327	intron_variant	4.00E-29
GCST009001	3	85836927	CADM2	rs12495178	intron_variant	2.00E-28
GCST90255621	3	85122152	CADM2	rs9309970	intron_variant	4.00E-28
GCST90179150	3	85609080	CADM2	rs1375561	intron_variant	9.00E-25
GCST90255621	3	85609080	CADM2	rs1375561	intron_variant	3.00E-23
GCST007039	3	85757163	CADM2	rs9876664	intron_variant	7.00E-20
GCST90018947	3	85632937	CADM2	rs12493563	intron_variant	9.00E-20
GCST009871	3	85757163	CADM2	rs9876664	intron_variant	1.00E-19
GCST009871	3	85811914	CADM2	rs9818122	intron_variant	3.00E-19
GCST009871	3	85118614	CADM2	rs6775464	intron_variant	2.00E-17
GCST007039	3	85061957	CADM2	rs7630382	intron_variant	2.00E-17
GCST006368	3	85758440	CADM2	rs13078960	intron_variant	4.00E-17
GCST90179150	3	85092413	CADM2	rs13095644	intron_variant	8.00E-17
GCST005951	3	85760275	CADM2	rs13068138	intron_variant	1.00E-16
GCST006811	3	85463965	CADM2	rs6762267	intron_variant	2.00E-15
GCST002783	3	85758440	CADM2	rs13078960	intron_variant	1.00E-14
GCST90179150	3	86122703	CADM2	rs7651114	intergenic_variant	1.00E-14
GCST002783	3	85758440	CADM2	rs13078960	intron_variant	2.00E-14
GCST009871	3	85476173	CADM2	rs12637791	intron_variant	5.00E-14
GCST009871	3	86201274	CADM2	rs35384579	intergenic_variant	1.00E-13
GCST90255621	3	86217258	CADM2	rs2137492	intergenic_variant	1.00E-12
GCST90267268	3	85532620	CADM2	rs62252504	intron_variant	6.00E-12
GCST002783	3	85758440	CADM2	rs13078960	intron_variant	1.00E-11
GCST009871	3	86122703	CADM2	rs7651114	intergenic_variant	3.00E-11
GCST000830	3	85835000	CADM2	rs13078807	intron_variant	4.00E-11
GCST006368	3	85649427	CADM2	rs62261676	intron_variant	9.00E-11
GCST90255621	3	62727468	CADPS	rs925018	intron_variant	4.00E-20
GCST90255621	3	62495388	CADPS	rs1452075	intron_variant	3.00E-17
GCST90255621	3	62448236	CADPS	rs13097150	intron_variant	2.00E-12
GCST009004	3	62495388	CADPS	rs1452075	intron_variant	3.00E-12
GCST009003	3	62495388	CADPS	rs1452075	intron_variant	3.00E-12
GCST90179150	3	62495388	CADPS	rs1452075	intron_variant	3.00E-12
GCST009871	3	62474144	CADPS	rs76824303	non_coding_transcript_exon_	4.00E-12
GCST007039	3	62474144	CADPS	rs76824303	non_coding_transcript_exon_	1.00E-11
GCST007039	3	62727588	CADPS	rs557951	intron_variant	1.00E-11
GCST90179150	3	62727588	CADPS	rs557951	intron_variant	8.00E-11
GCST90271767	3	49860840	CAMKV	rs2681781	synonymous_variant	7.00E-52
GCST90271772	3	49860840	CAMKV	rs2681781	synonymous_variant	2.00E-49
GCST90271769	3	49869678	CAMKV	rs6446187	intron_variant	1.00E-27
GCST009871	3	49860885	CAMKV	rs62260755	non_coding_transcript_exon_	1.00E-20
GCST90255621	3	42267243	CCK	rs10460960	intergenic_variant	4.00E-18
GCST009004	3	42291132	CCK	rs9839267	regulatory_region_variant	2.00E-17
GCST009003	3	42291132	CCK	rs9839267	regulatory_region_variant	2.00E-17
GCST90179150	3	42267243	CCK	rs10460960	intergenic_variant	2.00E-17
GCST90018947	3	42283205	CCK	rs75128851	intergenic_variant	5.00E-16
GCST009871	3	42287621	CCK	rs111768603	intergenic_variant	2.00E-14
GCST007039	3	42287621	CCK	rs111768603	intergenic_variant	2.00E-13
GCST004904	3	42267243	CCK	rs10460960	intergenic_variant	3.00E-11
GCST90179150	3	157144252	CCNL1	rs6809307	intergenic_variant	3.00E-13
GCST009004	3	157144252	CCNL1	rs6809307	intergenic_variant	8.00E-13
GCST90255621	3	108101216	CD47	rs7640424	intergenic_variant	3.00E-14

GCST90179150	3	108101216	CD47	rs7640424	intergenic_variant	1.00E-13
GCST90255621	3	88055261	CGGBP1	rs1006896	3_prime_UTR_variant	7.00E-25
GCST90179150	3	88055261	CGGBP1	rs1006896	3_prime_UTR_variant	6.00E-17
GCST90255621	3	132039233	CPNE4	rs1320903	intron_variant	1.00E-42
GCST009004	3	132032784	CPNE4	rs7631156	intron_variant	3.00E-32
GCST009001	3	132039233	CPNE4	rs1320903	intron_variant	4.00E-32
GCST90179150	3	132042937	CPNE4	rs7645921	intron_variant	1.00E-31
GCST009003	3	131860966	CPNE4	rs7428670	intron_variant	6.00E-31
GCST007039	3	132039233	CPNE4	rs1320903	intron_variant	6.00E-28
GCST009871	3	131832183	CPNE4	rs11709402	intron_variant	2.00E-27
GCST90255621	3	131909373	CPNE4	rs1358704	intron_variant	8.00E-24
GCST009871	3	132031622	CPNE4	rs1406778	intron_variant	7.00E-21
GCST90018947	3	131998951	CPNE4	rs7613261	intron_variant	3.00E-19
GCST90179150	3	131803708	CPNE4	rs1112706	intron_variant	1.00E-18
GCST009871	3	131788093	CPNE4	rs17295394	intron_variant	2.00E-17
GCST009121	3	131924008	CPNE4	rs1225053	intron_variant	3.00E-17
GCST90255621	3	131874489	CPNE4	rs77430764	intron_variant	4.00E-15
GCST009107	3	131924008	CPNE4	rs1225053	intron_variant	2.00E-12
GCST90255621	3	131887766	CPNE4	rs9289408	intron_variant	4.00E-12
GCST90179150	3	131887766	CPNE4	rs9289408	intron_variant	4.00E-11
GCST90255621	3	131714188	CPNE4	rs1393558	intron_variant	4.00E-11
GCST009871	3	132011398	CPNE4	rs10512839	intron_variant	8.00E-11
GCST90255621	3	82597368	CYP51A1P1	rs716764	intergenic_variant	8.00E-18
GCST009871	3	82660296	CYP51A1P1	rs34234711	intergenic_variant	2.00E-14
GCST90179150	3	82593583	CYP51A1P1	rs7647242	intergenic_variant	2.00E-13
GCST007039	3	82580372	CYP51A1P1	rs4123668	intergenic_variant	6.00E-13
GCST009871	3	82623056	CYP51A1P1	rs67338294	intergenic_variant	2.00E-12
GCST90255621	3	82566886	CYP51A1P1	rs13097360	intergenic_variant	2.00E-12
GCST90179150	3	82655537	CYP51A1P1	rs56131118	intergenic_variant	5.00E-11
GCST90255621	3	186116710	DGKG	rs9816226	intron_variant	7.00E-74
GCST009004	3	186116710	DGKG	rs9816226	intron_variant	1.00E-50
GCST009001	3	186116710	DGKG	rs9816226	intron_variant	1.00E-50
GCST007039	3	186110676	DGKG	rs73052033	intron_variant	3.00E-34
GCST009871	3	186110676	DGKG	rs73052033	intron_variant	5.00E-33
GCST006368	3	186116501	DGKG	rs7647305	intron_variant	7.00E-31
GCST000830	3	186116710	DGKG	rs9816226	intron_variant	2.00E-18
GCST004557	3	186116501	DGKG	rs7647305	intron_variant	3.00E-17
GCST004557	3	186116501	DGKG	rs7647305	intron_variant	6.00E-15
GCST004558	3	186116501	DGKG	rs7647305	intron_variant	2.00E-14
GCST90179150	3	186131401	DGKG	rs9839150	intron_variant	9.00E-13
GCST000296	3	186116501	DGKG	rs7647305	intron_variant	7.00E-11
GCST90255621	3	154301098	DHX36	rs9438	missense_variant	6.00E-21
GCST009004	3	154317161	DHX36	rs355777	intron_variant	2.00E-18
GCST009001	3	154317161	DHX36	rs355777	intron_variant	2.00E-18
GCST90179150	3	154320623	DHX36	rs171390	intron_variant	4.00E-18
GCST009003	3	154306376	DHX36	rs355754	intron_variant	7.00E-18
GCST009871	3	154317161	DHX36	rs355777	intron_variant	3.00E-15
GCST007039	3	154317161	DHX36	rs355777	intron_variant	3.00E-15
GCST008129	3	154301098	DHX36	rs9438	missense_variant	2.00E-13
GCST90255621	3	51274143	DOCK3	rs10433609	intron_variant	6.00E-12
GCST90255621	3	50734193	DOCK3	rs73072483	intron_variant	4.00E-11

GCST90255621	3	184277553	EEF1AKMT4	rs1001817	intron_variant	7.00E-29
GCST90179150	3	184278280	EEF1AKMT4	rs3752904	synonymous_variant	6.00E-13
GCST009004	3	184278280	EEF1AKMT4	rs3752904	synonymous_variant	9.00E-13
GCST008129	3	184258315	EEF1AKMT4	rs11546878	missense_variant	2.00E-12
GCST007039	3	184258315	EEF1AKMT4	rs11546878	missense_variant	8.00E-11
GCST90255621	3	42376490	EIF4BP4	rs33485	regulatory_region_variant	2.00E-20
GCST90179150	3	42376490	EIF4BP4	rs33485	regulatory_region_variant	6.00E-15
GCST009004	3	42376954	EIF4BP4	rs28350	regulatory_region_variant	1.00E-14
GCST009001	3	42376490	EIF4BP4	rs33485	regulatory_region_variant	1.00E-14
GCST009871	3	42376954	EIF4BP4	rs28350	regulatory_region_variant	3.00E-12
GCST009004	3	170910120	EIF5A2	rs6804915	intron_variant	2.00E-16
GCST009871	3	170910120	EIF5A2	rs6804915	intron_variant	3.00E-15
GCST90267268	3	170892527	EIF5A2	rs6792607	3_prime_UTR_variant	4.00E-12
GCST009003	3	186104564	ETV5	rs10513801	intron_variant	6.00E-49
GCST90179150	3	186104564	ETV5	rs10513801	intron_variant	1.00E-47
GCST011336	3	186101093	ETV5	rs4234589	intron_variant	4.00E-33
GCST005951	3	186106215	ETV5	rs1516725	intron_variant	2.00E-31
GCST90018947	3	186108951	ETV5	rs869400	5_prime_UTR_variant	5.00E-25
GCST009371	3	186106215	ETV5	rs1516725	intron_variant	1.00E-24
GCST004904	3	186106215	ETV5	rs1516725	intron_variant	1.00E-24
GCST002783	3	186106215	ETV5	rs1516725	intron_variant	1.00E-24
GCST002783	3	186106215	ETV5	rs1516725	intron_variant	2.00E-22
GCST004557	3	186096853	ETV5	rs6809651	intron_variant	3.00E-17
GCST004558	3	186096853	ETV5	rs6809651	intron_variant	9.00E-17
GCST004558	3	186096853	ETV5	rs6809651	intron_variant	1.00E-16
GCST004497	3	186106215	ETV5	rs1516725	intron_variant	3.00E-16
GCST002783	3	186106215	ETV5	rs1516725	intron_variant	2.00E-15
GCST004495	3	186106215	ETV5	rs1516725	intron_variant	6.00E-15
GCST90255621	3	186045430	ETV5	rs4461451	regulatory_region_variant	1.00E-14
GCST004557	3	186096853	ETV5	rs6809651	intron_variant	6.00E-14
GCST004558	3	186101093	ETV5	rs4234589	intron_variant	3.00E-13
GCST004559	3	186096853	ETV5	rs6809651	intron_variant	9.00E-13
GCST004559	3	186096853	ETV5	rs6809651	intron_variant	1.00E-12
GCST002783	3	186106215	ETV5	rs1516725	intron_variant	1.00E-12
GCST004497	3	186106215	ETV5	rs1516725	intron_variant	8.00E-12
GCST90131907	3	186080236	ETV5	rs77805826	intron_variant	1.00E-11
GCST004495	3	186106215	ETV5	rs1516725	intron_variant	2.00E-11
GCST004559	3	186096853	ETV5	rs6809651	intron_variant	3.00E-11
GCST90179150	3	186045430	ETV5	rs4461451	regulatory_region_variant	3.00E-11
GCST90255622	3	186080236	ETV5	rs77805826	intron_variant	4.00E-11
GCST90179150	3	104887286	EZRP1 - ALCAM	rs1436343	regulatory_region_variant	8.00E-18
GCST009004	3	104887300	EZRP1 - ALCAM	rs1436344	regulatory_region_variant	1.00E-17
GCST006368	3	104899129	EZRP1 - ALCAM	rs1436351	intergenic_variant	2.00E-17
GCST90255621	3	104887300	EZRP1 - ALCAM	rs1436344	regulatory_region_variant	5.00E-17
GCST009001	3	104899129	EZRP1 - ALCAM	rs1436351	intergenic_variant	5.00E-16
GCST007039	3	104912759	EZRP1 - ALCAM	rs9830592	intergenic_variant	1.00E-15
GCST009871	3	104912759	EZRP1 - ALCAM	rs9830592	intergenic_variant	6.00E-15
GCST90255621	3	35076284	FECHP1 - KRT8P18	rs11921432	intergenic_variant	6.00E-16
GCST90179150	3	35076284	FECHP1 - KRT8P18	rs11921432	intergenic_variant	9.00E-12
GCST90255621	3	61250788	FHIT	rs2365389	intron_variant	1.00E-35
GCST90179150	3	61250788	FHIT	rs2365389	intron_variant	6.00E-25

GCST009004	3	61201372	FHIT	rs1916801	intron_variant	2.00E-24
GCST009003	3	61201372	FHIT	rs1916801	intron_variant	2.00E-24
GCST009001	3	61201372	FHIT	rs1916801	intron_variant	2.00E-24
GCST007039	3	61278410	FHIT	rs815715	intergenic_variant	3.00E-18
GCST009871	3	61222945	FHIT	rs17668356	intron_variant	6.00E-18
GCST004497	3	61250788	FHIT	rs2365389	intron_variant	2.00E-16
GCST006368	3	61250788	FHIT	rs2365389	intron_variant	3.00E-15
GCST004495	3	61250788	FHIT	rs2365389	intron_variant	4.00E-15
GCST004557	3	61250788	FHIT	rs2365389	intron_variant	2.00E-14
GCST004558	3	61250788	FHIT	rs2365389	intron_variant	4.00E-14
GCST004557	3	61250788	FHIT	rs2365389	intron_variant	7.00E-14
GCST004904	3	61250788	FHIT	rs2365389	intron_variant	9.00E-14
GCST004558	3	61250788	FHIT	rs2365389	intron_variant	2.00E-13
GCST005951	3	61250788	FHIT	rs2365389	intron_variant	4.00E-13
GCST90018947	3	61278870	FHIT	rs148535477	intergenic_variant	6.00E-13
GCST009871	3	61174553	FHIT	rs13320646	intron_variant	1.00E-12
GCST004559	3	61250788	FHIT	rs2365389	intron_variant	3.00E-12
GCST006802	3	61250788	FHIT	rs2365389	intron_variant	4.00E-12
GCST004559	3	61250788	FHIT	rs2365389	intron_variant	5.00E-12
GCST004497	3	61250788	FHIT	rs2365389	intron_variant	1.00E-11
GCST004499	3	61250788	FHIT	rs2365389	intron_variant	7.00E-11
GCST90255621	3	81742961	GBE1	rs3849570	intron_variant	3.00E-16
GCST009001	3	81742961	GBE1	rs3849570	intron_variant	7.00E-15
GCST90179150	3	81742961	GBE1	rs3849570	intron_variant	3.00E-14
GCST005951	3	81824858	GBE1	rs6792696	intergenic_variant	7.00E-11
GCST90179150	3	123492217	HACD2	rs10668	3_prime_UTR_variant	1.00E-11
GCST90255621	3	123492217	HACD2	rs10668	3_prime_UTR_variant	2.00E-11
GCST009004	3	123567009	HACD2	rs16834431	intron_variant	3.00E-11
GCST007039	3	123492217	HACD2	rs10668	3_prime_UTR_variant	7.00E-11
GCST009871	3	108305118	HHLA2	rs2016469	intron_variant	2.00E-11
GCST007039	3	108305118	HHLA2	rs2016469	intron_variant	2.00E-11
GCST90179150	3	90315787	HSPE1P19	rs13097401	intergenic_variant	3.00E-14
GCST009871	3	90445366	HSPE1P19	rs73143793	intergenic_variant	2.00E-12
GCST009871	3	93799185	HSPE1P19	rs9713501	intergenic_variant	6.00E-12
GCST90018947	3	90281404	HSPE1P19	rs9821024	intergenic_variant	6.00E-11
GCST90179150	3	50299991	HYAL3	rs1283	3_prime_UTR_variant	7.00E-12
GCST90255621	3	50295266	HYAL3	rs13100173	missense_variant	4.00E-15
GCST011329	3	185787999	IGF2BP2	rs4481184	intron_variant	5.00E-22
GCST004904	3	185806293	IGF2BP2	rs4686392	intron_variant	1.00E-18
GCST90018727	3	185830129	IGF2BP2	rs764129	intergenic_variant	7.00E-18
GCST90255621	3	185826178	IGF2BP2	rs142611765	regulatory_region_variant	2.00E-11
GCST90271771	3	52781889	ITIH1	rs2710323	intron_variant	3.00E-27
GCST90179150	3	52781889	ITIH1	rs2710323	intron_variant	7.00E-19
GCST009871	3	52780240	ITIH1	rs2302417	non_coding_transcript_exon_	3.00E-16
GCST90255621	3	52799789	ITIH3	rs3617	missense_variant	1.00E-30
GCST004904	3	52796993	ITIH3	rs2240920	intron_variant	2.00E-12
GCST007039	3	45331950	LARS2	rs9852062	intergenic_variant	2.00E-12
GCST009871	3	45331950	LARS2	rs9852062	intergenic_variant	5.00E-12
GCST90255621	3	45333004	LARS2	rs4683096	regulatory_region_variant	6.00E-11
GCST90179150	3	153974160	LINC02006	rs1357079	intron_variant	1.00E-14
GCST009871	3	153940162	LINC02006	rs1568488	intron_variant	2.00E-14

GCST90255621	3	42451242	LYZL4	rs9828788	regulatory_region_variant	1.00E-11
GCST009871	3	42449393	LYZL4	rs9876105	regulatory_region_variant	6.00E-11
GCST90018947	3	49912401	MON1A	rs7634084	non_coding_transcript_exon_	9.00E-40
GCST009871	3	49928176	MON1A	rs35917071	intron_variant	2.00E-16
GCST90255621	3	49928176	MON1A	rs35917071	intron_variant	7.00E-15
GCST006368	3	49934081	MON1A	rs7613875	intergenic_variant	7.00E-14
GCST009004	3	138372298	MRAS	rs1199334	intron_variant	2.00E-12
GCST90255621	3	138372298	MRAS	rs1199334	intron_variant	3.00E-12
GCST90179150	3	138394511	MRAS	rs253664	intron_variant	6.00E-12
GCST90255621	3	138403078	MRAS	rs3732837	3_prime_UTR_variant	2.00E-11
GCST90255621	3	49898669	MST1R	rs2230590	missense_variant	3.00E-56
GCST007039	3	49883138	MST1R	rs9843653	intergenic_variant	3.00E-47
GCST90179150	3	49883138	MST1R	rs9843653	intergenic_variant	4.00E-46
GCST009004	3	49878073	MST1R	rs9862795	regulatory_region_variant	4.00E-44
GCST009003	3	49878073	MST1R	rs9862795	regulatory_region_variant	4.00E-44
GCST008129	3	49898669	MST1R	rs2230590	missense_variant	9.00E-26
GCST90255621	3	49902645	MST1R	rs2230593	missense_variant	3.00E-11
GCST009004	3	108400224	MYH15	rs4273371	intron_variant	4.00E-12
GCST009001	3	108400224	MYH15	rs4273371	intron_variant	4.00E-12
GCST90179150	3	108400224	MYH15	rs4273371	intron_variant	5.00E-12
GCST90255621	3	108400224	MYH15	rs4273371	intron_variant	5.00E-11
GCST007039	3	88684137	NDUFA5P5 - ICE2P2	rs11128058	intergenic_variant	1.00E-11
GCST009871	3	88700549	NDUFA5P5 - ICE2P2	rs2345404	intergenic_variant	2.00E-11
GCST90179150	3	88684137	NDUFA5P5 - ICE2P2	rs11128058	intergenic_variant	3.00E-11
GCST90255621	3	88719126	NDUFA5P5 - ICE2P2	rs9823212	intergenic_variant	6.00E-11
GCST009871	3	173396515	NLGN1	rs529200	5_prime_UTR_variant	1.00E-17
GCST007039	3	173396515	NLGN1	rs529200	5_prime_UTR_variant	1.00E-17
GCST90018947	3	173396515	NLGN1	rs529200	5_prime_UTR_variant	8.00E-15
GCST90255621	3	173937430	NLGN1	rs1388475	intron_variant	3.00E-12
GCST90255621	3	94319241	NSUN3	rs1454687	intergenic_variant	9.00E-37
GCST007039	3	94319241	NSUN3	rs1454687	intergenic_variant	1.00E-30
GCST009871	3	94266256	NSUN3	rs897186	intergenic_variant	3.00E-28
GCST009004	3	94284759	NSUN3	rs1580099	intergenic_variant	7.00E-27
GCST009003	3	94284759	NSUN3	rs1580099	intergenic_variant	7.00E-27
GCST90179150	3	94254369	NSUN3	rs7640233	intergenic_variant	5.00E-26
GCST009001	3	94352637	NSUN3	rs1492014	intergenic_variant	1.00E-23
GCST90018947	3	94312995	NSUN3	rs13073568	intergenic_variant	1.00E-22
GCST90179150	3	48996052	P4HTM	rs62262471	intron_variant	4.00E-16
GCST90255621	3	48996052	P4HTM	rs62262471	intron_variant	1.00E-15
GCST90255621	3	136207780	PCCB	rs645040	regulatory_region_variant	5.00E-25
GCST90271770	3	136213517	PCCB	rs687339	intergenic_variant	7.00E-25
GCST90179150	3	136213517	PCCB	rs687339	intergenic_variant	2.00E-22
GCST009001	3	136207780	PCCB	rs645040	regulatory_region_variant	1.00E-21
GCST90255621	3	136255374	PCCB	rs9844666	5_prime_UTR_variant	4.00E-20
GCST90255621	3	12351626	PPARG	rs1801282	missense_variant	5.00E-26
GCST008129	3	12351626	PPARG	rs1801282	missense_variant	1.00E-16
GCST011329	3	12355456	PPARG	rs2881654	intron_variant	9.00E-14
GCST90255621	3	12282731	PPARG	rs2920503	intergenic_variant	2.00E-13
GCST009004	3	12385437	PPARG	rs10510419	intron_variant	2.00E-13
GCST90179150	3	12295008	PPARG	rs11709077	intron_variant	1.00E-12
GCST90179150	3	12385437	PPARG	rs10510419	intron_variant	1.00E-12

GCST007039	3	12282731	PPARG	rs2920503	intergenic_variant	3.00E-11
GCST009871	3	136085708	PPP2R3A	rs61791721	intron_variant	7.00E-16
GCST009871	3	136127989	PPP2R3A	rs13082532	intron_variant	2.00E-13
GCST90255621	3	58410136	PXK	rs56384862	missense_variant	3.00E-14
GCST008129	3	58410136	PXK	rs56384862	missense_variant	8.00E-12
GCST90255621	3	25064946	RARB	rs6804842	intron_variant	6.00E-28
GCST90179150	3	25064946	RARB	rs6804842	intron_variant	9.00E-19
GCST009004	3	25064946	RARB	rs6804842	intron_variant	8.00E-18
GCST009001	3	25064946	RARB	rs6804842	intron_variant	8.00E-18
GCST007039	3	25068924	RARB	rs7619139	intron_variant	3.00E-14
GCST009871	3	25068924	RARB	rs7619139	intron_variant	9.00E-14
GCST90018947	3	25038387	RARB	rs10628337	intron_variant	9.00E-14
GCST004904	3	25058285	RARB	rs10510554	intron_variant	3.00E-12
GCST006368	3	25064946	RARB	rs6804842	intron_variant	6.00E-12
GCST004558	3	25058285	RARB	rs10510554	intron_variant	2.00E-11
GCST004557	3	25058285	RARB	rs10510554	intron_variant	6.00E-11
GCST005951	3	25058285	RARB	rs10510554	intron_variant	8.00E-11
GCST90255621	3	141556594	RASA2	rs16851483	intron_variant	1.00E-36
GCST009004	3	141556594	RASA2	rs16851483	intron_variant	5.00E-25
GCST009003	3	141556594	RASA2	rs16851483	intron_variant	5.00E-25
GCST009001	3	141616279	RASA2	rs2640017	intergenic_variant	6.00E-24
GCST90179150	3	141556594	RASA2	rs16851483	intron_variant	7.00E-24
GCST90018947	3	141484092	RASA2	rs138685654	intergenic_variant	1.00E-18
GCST009871	3	141561431	RASA2	rs3821709	intron_variant	3.00E-18
GCST007039	3	141561431	RASA2	rs3821709	intron_variant	3.00E-18
GCST90255621	3	141488733	RASA2	rs11917587	intron_variant	1.00E-17
GCST005951	3	141556594	RASA2	rs16851483	intron_variant	4.00E-15
GCST008129	3	141607760	RASA2	rs295322	splice_region_variant	4.00E-14
GCST004495	3	141556594	RASA2	rs16851483	intron_variant	3.00E-12
GCST90179150	3	141605714	RASA2	rs295320	intron_variant	1.00E-11
GCST004499	3	141556594	RASA2	rs16851483	intron_variant	2.00E-11
GCST004497	3	141556594	RASA2	rs16851483	intron_variant	2.00E-11
GCST004904	3	141556594	RASA2	rs16851483	intron_variant	8.00E-11
GCST004557	3	141587171	RASA2	rs2035935	intron_variant	8.00E-11
GCST90255621	3	50042741	RBM6	rs12631248	intron_variant	4.00E-18
GCST90179150	3	50067275	RBM6	rs11924670	intron_variant	2.00E-14
GCST009871	3	53106587	RFT1	rs1004807	intron_variant	3.00E-14
GCST90179150	3	53110347	RFT1	rs9838517	intron_variant	6.00E-13
GCST90255621	3	78409778	RN7SKP61 - MRPS17P3	rs6419734	intergenic_variant	3.00E-12
GCST90179150	3	78409778	RN7SKP61 - MRPS17P3	rs6419734	intergenic_variant	3.00E-11
GCST009004	3	78409778	RN7SKP61 - MRPS17P3	rs6419734	intergenic_variant	4.00E-11
GCST90255621	3	78610906	ROBO1	rs3773192	intron_variant	2.00E-12
GCST90179150	3	78610906	ROBO1	rs3773192	intron_variant	2.00E-11
GCST006368	3	77597712	ROBO2	rs200870675	intron_variant	7.00E-16
GCST90255621	3	77590191	ROBO2	rs1523769	intron_variant	6.00E-14
GCST90179150	3	77617893	ROBO2	rs1523768	intron_variant	1.00E-13
GCST009004	3	77617893	ROBO2	rs1523768	intron_variant	4.00E-13
GCST009004	3	45181466	RPS24P8 - TMEM158	rs55676934	intergenic_variant	5.00E-12
GCST90179150	3	45181466	RPS24P8 - TMEM158	rs55676934	intergenic_variant	7.00E-12
GCST90255621	3	158189168	RSRC1	rs827156	intron_variant	1.00E-13
GCST009004	3	158308700	RSRC1	rs2682406	intron_variant	5.00E-13

GCST009001	3	158169483	RSRC1	rs9857883	intron_variant	2.00E-12
GCST90018947	3	158127380	RSRC1	rs9860241	intron_variant	4.00E-12
GCST90179150	3	158321816	RSRC1	rs827120	intron_variant	5.00E-12
GCST009871	3	158291688	RSRC1	rs13066362	intron_variant	2.00E-11
GCST007039	3	158183000	RSRC1	rs1730028	intron_variant	2.00E-11
GCST90255621	3	70490408	SAMMSON	rs11915371	intron_variant	1.00E-13
GCST009004	3	70490408	SAMMSON	rs11915371	intron_variant	2.00E-13
GCST009001	3	70490408	SAMMSON	rs11915371	intron_variant	2.00E-13
GCST90179150	3	70490408	SAMMSON	rs11915371	intron_variant	1.00E-12
GCST007039	3	70491614	SAMMSON	rs12487131	intron_variant	4.00E-12
GCST009871	3	70543930	SAMMSON	rs11708540	intergenic_variant	2.00E-11
GCST009001	3	50159664	SEMA3F	rs1046953	synonymous_variant	9.00E-38
GCST009871	3	50159664	SEMA3F	rs1046953	synonymous_variant	2.00E-37
GCST90179150	3	50137411	SEMA3F	rs71326918	intron_variant	1.00E-14
GCST90255621	3	9475685	SETD5	rs11542009	missense_variant	1.00E-15
GCST007039	3	9456459	SETD5	rs62246311	intron_variant	4.00E-13
GCST009871	3	9456459	SETD5	rs62246311	intron_variant	7.00E-13
GCST009004	3	9465630	SETD5	rs59302296	3_prime_UTR_variant	9.00E-12
GCST90179150	3	9465573	SETD5	rs60771966	3_prime_UTR_variant	4.00E-11
GCST006368	3	20673088	SGO1	rs4857968	intron_variant	1.00E-16
GCST90271767	3	20539657	SGO1	rs4858223	intron_variant	3.00E-15
GCST90271769	3	20539657	SGO1	rs4858223	intron_variant	8.00E-14
GCST90255621	3	20399558	SGO1	rs4858193	intron_variant	5.00E-13
GCST90179150	3	20399558	SGO1	rs4858193	intron_variant	4.00E-12
GCST009004	3	20673088	SGO1	rs4857968	intron_variant	7.00E-12
GCST90255621	3	171007094	SLC2A2	rs8192675	intron_variant	2.00E-22
GCST90179150	3	171007094	SLC2A2	rs8192675	intron_variant	1.00E-16
GCST007039	3	171016588	SLC2A2	rs61791109	intron_variant	1.00E-16
GCST009003	3	171017310	SLC2A2	rs11924032	intron_variant	3.00E-16
GCST009001	3	171014810	SLC2A2	rs7356034	intron_variant	2.00E-15
GCST001527	3	170999732	SLC2A2	rs11920090	intron_variant	8.00E-11
GCST009871	3	47775517	SMARCC1	rs72906474	intron_variant	2.00E-18
GCST90179150	3	47775517	SMARCC1	rs72906474	intron_variant	1.00E-17
GCST90255621	3	47775517	SMARCC1	rs72906474	intron_variant	3.00E-17
GCST007039	3	47599521	SMARCC1	rs4858802	intron_variant	1.00E-11
GCST90255621	3	181611894	SOX2-OT	rs6443750	intron_variant	1.00E-17
GCST90255621	3	181018465	SOX2-OT	rs10049181	intron_variant	6.00E-14
GCST009004	3	181611894	SOX2-OT	rs6443750	intron_variant	7.00E-13
GCST90179150	3	181611894	SOX2-OT	rs6443750	intron_variant	4.00E-12
GCST90179150	3	181235103	SOX2-OT	rs2718786	intron_variant	4.00E-11
GCST90255621	3	181449797	SOX2-OT	rs9841616	intron_variant	6.00E-11
GCST90271772	3	173389653	SPATA16	rs247975	intergenic_variant	1.00E-24
GCST009004	3	173377333	SPATA16	rs39654	intergenic_variant	2.00E-21
GCST009003	3	173389653	SPATA16	rs247975	intergenic_variant	3.00E-21
GCST90179150	3	173377333	SPATA16	rs39654	intergenic_variant	6.00E-21
GCST90255621	3	173377333	SPATA16	rs39654	intergenic_variant	2.00E-17
GCST90267268	3	173395890	SPATA16	rs556464	regulatory_region_variant	2.00E-11
GCST90255621	3	84221726	SRRM1P2	rs4276195	intergenic_variant	4.00E-17
GCST009871	3	84064340	SRRM1P2	rs114593013	intergenic_variant	2.00E-15
GCST90179150	3	84162542	SRRM1P2	rs76062229	intergenic_variant	2.00E-15
GCST009871	3	84579560	SRRM1P2	rs116032709	intergenic_variant	2.00E-14

GCST009871	3	83419768	SRRM1P2	rs115700790	intron_variant	7.00E-14
GCST007039	3	83396421	SRRM1P2	rs76162423	intron_variant	8.00E-12
GCST90255621	3	156581524	SSR3	rs7615297	regulatory_region_variant	9.00E-13
GCST009004	3	156577552	SSR3	rs9826775	regulatory_region_variant	7.00E-11
GCST009004	3	136553404	STAG1	rs7621025	intron_variant	8.00E-24
GCST009003	3	136553404	STAG1	rs7621025	intron_variant	8.00E-24
GCST009871	3	136609428	STAG1	rs1471740	non_coding_transcript_exon_	5.00E-18
GCST007039	3	136609428	STAG1	rs1471740	non_coding_transcript_exon_	7.00E-18
GCST90018947	3	136536327	STAG1	rs833752	intron_variant	2.00E-16
GCST009871	3	136634232	STAG1	rs13434116	intron_variant	9.00E-13
GCST90179150	3	136626977	STAG1	rs13066401	intron_variant	4.00E-11
GCST90271771	3	52234850	TWF2	rs353547	intron_variant	7.00E-18
GCST009871	3	52234850	TWF2	rs353547	intron_variant	1.00E-12
GCST90255621	3	196422294	UBXN7	rs7372674	intron_variant	9.00E-15
GCST007039	3	196444114	UBXN7	rs6583310	regulatory_region_variant	9.00E-12
GCST009871	3	196388626	UBXN7	rs34801745	intron_variant	2.00E-11
GCST009004	3	196388626	UBXN7	rs34801745	intron_variant	7.00E-11
GCST90255621	3	11599007	VGLL4	rs17776719	intron_variant	8.00E-12
GCST90018947	3	11600640	VGLL4	rs1561073	intron_variant	2.00E-11
GCST90255621	3	195156264	XXYLT1	rs11721261	intron_variant	4.00E-13
GCST90179150	3	195136857	XXYLT1	rs1009642	intron_variant	4.00E-12
GCST009004	3	195161027	XXYLT1	rs7616009	intron_variant	4.00E-11
GCST90018947	3	195156264	XXYLT1	rs11721261	intron_variant	8.00E-11
GCST009004	3	114680449	ZBTB20	rs17681451	intron_variant	7.00E-13
GCST90179150	3	114680449	ZBTB20	rs17681451	intron_variant	2.00E-12
GCST90255621	3	114680449	ZBTB20	rs17681451	intron_variant	7.00E-12
GCST002829	4	88131171	ABCF2P1	rs2231142	missense_variant	2.00E-30
GCST002830	4	88131171	ABCG2	rs2231142	missense_variant	2.00E-29
GCST002830	4	88124179	ABCG2	rs2199936	intron_variant	1.00E-22
GCST002829	4	88124179	ABCG2	rs2199936	intron_variant	1.00E-22
GCST90179150	4	88133515	ABCG2	rs4148155	intron_variant	8.00E-14
GCST90255621	4	88133515	ABCG2	rs4148155	intron_variant	9.00E-14
GCST009004	4	88117930	ABCG2	rs1481012	intron_variant	5.00E-13
GCST002830	4	88131171	ABCG2	rs2231142	missense_variant	6.00E-13
GCST009871	4	88124179	ABCG2	rs2199936	intron_variant	4.00E-12
GCST007039	4	88133515	ABCG2	rs4148155	intron_variant	1.00E-11
GCST002829	4	88131171	ABCG2	rs2231142	missense_variant	2.00E-11
GCST90018947	4	99318162	ADGRL2	rs1229984	missense_variant	5.00E-17
GCST90255621	4	99318162	ADH1B	rs1229984	missense_variant	3.00E-16
GCST007039	4	99318162	ADH1B	rs1229984	missense_variant	1.00E-12
GCST90018727	4	99323162	ADH1B	rs3811801	intergenic_variant	3.00E-11
GCST90255621	4	25407216	ALKBH3	rs34811474	missense_variant	8.00E-62
GCST90271767	4	25407216	ANAPC4	rs34811474	missense_variant	3.00E-39
GCST009004	4	25407216	ANAPC4	rs34811474	missense_variant	9.00E-38
GCST009003	4	25407216	ANAPC4	rs34811474	missense_variant	9.00E-38
GCST009001	4	25407216	ANAPC4	rs34811474	missense_variant	9.00E-38
GCST90179150	4	25407216	ANAPC4	rs34811474	missense_variant	4.00E-36
GCST007039	4	25407216	ANAPC4	rs34811474	missense_variant	1.00E-32
GCST90018947	4	25407216	ANAPC4	rs34811474	missense_variant	1.00E-31
GCST008129	4	25407216	ANAPC4	rs34811474	missense_variant	3.00E-30
GCST90271769	4	25407216	ANAPC4	rs34811474	missense_variant	1.00E-26

GCST90255621	4	25447603	ANAPC4	rs7671107	intergenic_variant	2.00E-11
GCST90255621	4	80028675	ANKS1B	rs4333130	intron_variant	5.00E-11
GCST009871	4	79887666	ANTXR2	rs11724750	intergenic_variant	7.00E-11
GCST90255621	4	101787840	BAIAP2	rs7377083	intron_variant	7.00E-32
GCST009871	4	101787840	BANK1	rs7377083	intron_variant	2.00E-27
GCST90179150	4	101787840	BANK1	rs7377083	intron_variant	2.00E-27
GCST90271771	4	102192193	BANK1	rs457134	intergenic_variant	5.00E-21
GCST009871	4	102192193	BANK1	rs457134	intergenic_variant	1.00E-15
GCST90255621	4	101514108	BANK1	rs17248480	intron_variant	7.00E-14
GCST90271767	4	101759884	BANK1	rs201081507	intron_variant	1.00E-13
GCST90179150	4	101759884	BANK1	rs201081507	intron_variant	4.00E-13
GCST90255621	4	101918130	BANK1	rs3733197	missense_variant	8.00E-13
GCST009871	4	101465477	BANK1	rs149496329	intron_variant	3.00E-11
GCST009871	4	101745628	BANK1	rs12511373	intron_variant	3.00E-11
GCST90255621	4	93515546	GRID2	rs6834210	intron_variant	5.00E-13
GCST009004	4	93490455	GRID2	rs2870710	intron_variant	9.00E-13
GCST90179150	4	93486690	GRID2	rs10014552	intron_variant	5.00E-12
GCST90255621	4	144412457	GYPA - KRT18P51	rs17019336	intron_variant	4.00E-14
GCST009004	4	144412457	GYPA - KRT18P51	rs17019336	intron_variant	2.00E-11
GCST90179150	4	144399854	GYPA - KRT18P51	rs7654571	intron_variant	2.00E-11
GCST009001	4	144399854	GYPA - KRT18P51	rs7654571	intron_variant	3.00E-11
GCST90271771	4	144737912	HHIP	rs11727676	synonymous_variant	1.00E-15
GCST90255621	4	144737912	HHIP	rs11727676	synonymous_variant	3.00E-11
GCST005951	4	144737912	HHIP	rs11727676	synonymous_variant	6.00E-11
GCST90255621	4	170711486	HSP90AA6P	rs1522569	intergenic_variant	4.00E-12
GCST007039	4	170714320	HSP90AA6P	rs111598585	intergenic_variant	2.00E-11
GCST009871	4	170714320	HSP90AA6P	rs111598585	intergenic_variant	8.00E-11
GCST009004	4	28513208	IGBP1P5	rs73213501	intron_variant	1.00E-14
GCST90179150	4	28513208	IGBP1P5	rs73213501	intron_variant	1.00E-14
GCST009003	4	28487717	IGBP1P5	rs73213484	intron_variant	2.00E-14
GCST007039	4	28513208	IGBP1P5	rs73213501	intron_variant	3.00E-14
GCST009871	4	28513208	IGBP1P5	rs73213501	intron_variant	6.00E-14
GCST90255621	4	44486179	KCTD8	rs11731229	intergenic_variant	2.00E-14
GCST90255622	4	44486694	KCTD8	rs57257292	intergenic_variant	2.00E-11
GCST009004	4	54639193	KIT	rs2192158	intergenic_variant	7.00E-16
GCST90179150	4	54633584	KIT	rs6554193	regulatory_region_variant	1.00E-15
GCST009003	4	54643581	KIT	rs11727273	intergenic_variant	4.00E-15
GCST009871	4	54635561	KIT	rs13106834	intergenic_variant	9.00E-15
GCST90255621	4	54639193	KIT	rs2192158	intergenic_variant	4.00E-14
GCST007039	4	54634059	KIT	rs6831020	intergenic_variant	1.00E-13
GCST90018947	4	54634059	KIT	rs6831020	intergenic_variant	7.00E-12
GCST90255621	4	38698036	KLF3	rs3209570	3_prime_UTR_variant	7.00E-20
GCST90179150	4	38698036	KLF3	rs3209570	3_prime_UTR_variant	1.00E-17
GCST009004	4	38691214	KLF3	rs1000096	intron_variant	2.00E-16
GCST009001	4	38691214	KLF3	rs1000096	intron_variant	2.00E-16
GCST006368	4	38653060	KLF3	rs4833079	intron_variant	8.00E-16
GCST007039	4	38697303	KLF3	rs36023504	3_prime_UTR_variant	2.00E-14
GCST009871	4	38691214	KLF3	rs1000096	intron_variant	3.00E-13
GCST009871	4	18510115	LCORL	rs1477890	intron_variant	5.00E-15
GCST007039	4	18510115	LCORL	rs1477890	intron_variant	6.00E-15
GCST90255621	4	18481782	LCORL	rs10939792	intron_variant	1.00E-14

GCST009004	4	18510115	LCORL	rs1477890	intron_variant	6.00E-14
GCST90179150	4	18510115	LCORL	rs1477890	intron_variant	8.00E-14
GCST009003	4	18513204	LCORL	rs1477887	intron_variant	9.00E-13
GCST90018947	4	18497219	LCORL	rs34430935	intron_variant	1.00E-12
GCST90255621	4	17884523	LCORL	rs17526944	missense_variant	7.00E-11
GCST90255621	4	129810129	LINC02465	rs4864201	intron_variant	9.00E-21
GCST90179150	4	129810129	LINC02465	rs4864201	intron_variant	3.00E-15
GCST009004	4	129805678	LINC02465	rs2391540	intron_variant	1.00E-14
GCST009001	4	129843450	LINC02465	rs2860199	intron_variant	2.00E-14
GCST007039	4	129803585	LINC02465	rs4398538	intron_variant	7.00E-13
GCST009871	4	129805678	LINC02465	rs2391540	intron_variant	4.00E-12
GCST90018947	4	129838492	LINC02465	rs2952863	intron_variant	1.00E-11
GCST009004	4	139942211	MAML3	rs57800857	intron_variant	3.00E-15
GCST009003	4	139942211	MAML3	rs57800857	intron_variant	3.00E-15
GCST90179150	4	139942211	MAML3	rs57800857	intron_variant	6.00E-15
GCST009871	4	139942211	MAML3	rs57800857	intron_variant	3.00E-13
GCST90255621	4	139965809	MAML3	rs13109980	intron_variant	3.00E-13
GCST007039	4	139942211	MAML3	rs57800857	intron_variant	1.00E-12
GCST90179150	4	139866541	MAML3	rs2271391	intron_variant	1.00E-11
GCST90018947	4	139941525	MAML3	rs141468630	intron_variant	3.00E-11
GCST009871	4	139872377	MAML3	rs2667360	intron_variant	4.00E-11
GCST009004	4	64786012	MTCO3P28	rs1346841	intergenic_variant	3.00E-13
GCST90179150	4	64786012	MTCO3P28	rs1346841	intergenic_variant	5.00E-13
GCST90255621	4	64874669	MTCO3P28	rs17085463	intron_variant	5.00E-12
GCST90255621	4	31010688	PCDH7	rs4596205	intron_variant	6.00E-17
GCST90255621	4	30701621	PCDH7	rs4270551	intergenic_variant	4.00E-13
GCST90179150	4	31021988	PCDH7	rs10007906	intron_variant	1.00E-12
GCST007039	4	30841158	PCDH7	rs4527444	intron_variant	1.00E-12
GCST90018947	4	30999679	PCDH7	rs4132132	intron_variant	1.00E-12
GCST90179150	4	30838171	PCDH7	rs7434610	intron_variant	2.00E-12
GCST009004	4	30701621	PCDH7	rs4270551	intergenic_variant	8.00E-12
GCST90255621	4	30838709	PCDH7	rs7656673	intron_variant	1.00E-11
GCST90179150	4	30701621	PCDH7	rs4270551	intergenic_variant	5.00E-11
GCST007039	4	101261042	PPP3CA	rs2583410	intron_variant	1.00E-12
GCST90179150	4	101283372	PPP3CA	rs2583401	intron_variant	9.00E-12
GCST90255621	4	45180510	PRDX4P1	rs10938397	intergenic_variant	2.00E-114
GCST90179150	4	45180510	PRDX4P1	rs10938397	intergenic_variant	1.00E-87
GCST009004	4	45180510	PRDX4P1	rs10938397	intergenic_variant	2.00E-86
GCST009003	4	45180510	PRDX4P1	rs10938397	intergenic_variant	2.00E-86
GCST009001	4	45173674	PRDX4P1	rs13130484	intergenic_variant	8.00E-86
GCST90103755	4	45180510	PRDX4P1	rs10938397	intergenic_variant	3.00E-53
GCST007039	4	45179317	PRDX4P1	rs12507026	intergenic_variant	3.00E-52
GCST009871	4	45179317	PRDX4P1	rs12507026	intergenic_variant	3.00E-51
GCST90018947	4	45180510	PRDX4P1	rs10938397	intergenic_variant	1.00E-49
GCST005951	4	45180510	PRDX4P1	rs10938397	intergenic_variant	1.00E-48
GCST004904	4	45180510	PRDX4P1	rs10938397	intergenic_variant	3.00E-48
GCST006368	4	45180510	PRDX4P1	rs10938397	intergenic_variant	1.00E-47
GCST004519	4	45180510	PRDX4P1	rs10938397	intergenic_variant	6.00E-46
GCST009121	4	45179317	PRDX4P1	rs12507026	intergenic_variant	7.00E-41
GCST002783	4	45180510	PRDX4P1	rs10938397	intergenic_variant	1.00E-40
GCST002783	4	45180510	PRDX4P1	rs10938397	intergenic_variant	3.00E-38

GCST000830	4	45180510	PRDX4P1	rs10938397	intergenic_variant	4.00E-31
GCST003177	4	45173674	PRDX4P1	rs13130484	intergenic_variant	2.00E-23
GCST004495	4	45180510	PRDX4P1	rs10938397	intergenic_variant	2.00E-23
GCST002783	4	45180510	PRDX4P1	rs10938397	intergenic_variant	3.00E-23
GCST004497	4	45180510	PRDX4P1	rs10938397	intergenic_variant	7.00E-23
GCST002783	4	45180510	PRDX4P1	rs10938397	intergenic_variant	2.00E-22
GCST004557	4	45180510	PRDX4P1	rs10938397	intergenic_variant	2.00E-21
GCST004558	4	45180510	PRDX4P1	rs10938397	intergenic_variant	2.00E-20
GCST004557	4	45180510	PRDX4P1	rs10938397	intergenic_variant	3.00E-20
GCST004558	4	45180510	PRDX4P1	rs10938397	intergenic_variant	2.00E-19
GCST90255621	4	45176064	PRDX4P1	rs74715904	intergenic_variant	2.00E-19
GCST004499	4	45180510	PRDX4P1	rs10938397	intergenic_variant	4.00E-18
GCST004904	4	45162620	PRDX4P1	rs1996023	intergenic_variant	1.00E-17
GCST90018727	4	45162620	PRDX4P1	rs1996023	intergenic_variant	2.00E-17
GCST90255621	4	45130272	PRDX4P1	rs4974508	intergenic_variant	5.00E-17
GCST90255622	4	45177866	PRDX4P1	rs12641981	intergenic_variant	2.00E-16
GCST000298	4	45180510	PRDX4P1	rs10938397	intergenic_variant	3.00E-16
GCST90239604	4	45173674	PRDX4P1	rs13130484	intergenic_variant	8.00E-16
GCST90179150	4	45130272	PRDX4P1	rs4974508	intergenic_variant	4.00E-15
GCST90255621	4	45028592	PRDX4P1	rs13140100	intron_variant	5.00E-15
GCST007240	4	45179317	PRDX4P1	rs12507026	intergenic_variant	6.00E-15
GCST004559	4	45180510	PRDX4P1	rs10938397	intergenic_variant	1.00E-14
GCST004559	4	45180510	PRDX4P1	rs10938397	intergenic_variant	3.00E-14
GCST004557	4	45177866	PRDX4P1	rs12641981	intergenic_variant	1.00E-13
GCST008025	4	45180510	PRDX4P1	rs10938397	intergenic_variant	1.00E-13
GCST001955	4	45180510	PRDX4P1	rs10938397	intergenic_variant	2.00E-13
GCST004497	4	45180510	PRDX4P1	rs10938397	intergenic_variant	2.00E-13
GCST004495	4	45180510	PRDX4P1	rs10938397	intergenic_variant	2.00E-13
GCST009871	4	45176064	PRDX4P1	rs74715904	intergenic_variant	2.00E-13
GCST008025	4	45173674	PRDX4P1	rs13130484	intergenic_variant	2.00E-13
GCST90131907	4	45173674	PRDX4P1	rs13130484	intergenic_variant	2.00E-13
GCST004495	4	45180510	PRDX4P1	rs10938397	intergenic_variant	4.00E-13
GCST009764	4	45184122	PRDX4P1	rs10938398	intergenic_variant	4.00E-13
GCST009871	4	45283052	PRDX4P1	rs35004261	intergenic_variant	4.00E-13
GCST006802	4	45180510	PRDX4P1	rs10938397	intergenic_variant	6.00E-13
GCST009871	4	45034429	PRDX4P1	rs12503491	intron_variant	7.00E-13
GCST004558	4	45177866	PRDX4P1	rs12641981	intergenic_variant	1.00E-12
GCST004499	4	45180510	PRDX4P1	rs10938397	intergenic_variant	2.00E-12
GCST008158	4	45163634	PRDX4P1	rs144582188	intergenic_variant	3.00E-12
GCST90255621	4	44953471	PRDX4P1	rs17573940	intergenic_variant	3.00E-12
GCST90255621	4	45253835	PRDX4P1	rs17637290	intergenic_variant	5.00E-12
GCST004557	4	45173674	PRDX4P1	rs13130484	intergenic_variant	6.00E-12
GCST009871	4	45074199	PRDX4P1	rs1159118	intergenic_variant	7.00E-12
GCST004557	4	45180510	PRDX4P1	rs10938397	intergenic_variant	9.00E-12
GCST007241	4	45182890	PRDX4P1	rs13104545	intergenic_variant	1.00E-11
GCST90179150	4	45176064	PRDX4P1	rs74715904	intergenic_variant	1.00E-11
GCST009107	4	45179317	PRDX4P1	rs12507026	intergenic_variant	2.00E-11
GCST004557	4	45180510	PRDX4P1	rs10938397	intergenic_variant	2.00E-11
GCST004497	4	45180510	PRDX4P1	rs10938397	intergenic_variant	3.00E-11
GCST004558	4	45180510	PRDX4P1	rs10938397	intergenic_variant	3.00E-11
GCST008025	4	45182425	PRDX4P1	rs348495	intergenic_variant	4.00E-11

GCST90267268	4	45066912	PRDX4P1	rs4488965	intergenic_variant	6.00E-11
GCST90018947	4	3279785	RGS12	rs199500642	intergenic_variant	7.00E-13
GCST007039	4	3297073	RGS12	rs2051559	intron_variant	1.00E-11
GCST009004	4	3297073	RGS12	rs2051559	intron_variant	4.00E-11
GCST90179150	4	3297073	RGS12	rs2051559	intron_variant	5.00E-11
GCST90255621	4	3297073	RGS12	rs2051559	intron_variant	5.00E-11
GCST009871	4	3297073	RGS12	rs2051559	intron_variant	6.00E-11
GCST009871	4	179246870	RNA5SP173 - NDUFB5P1	rs1037702	intergenic_variant	6.00E-12
GCST007039	4	179246870	RNA5SP173 - NDUFB5P1	rs1037702	intergenic_variant	6.00E-12
GCST90255621	4	111781479	RPL36AP23	rs1585471	intergenic_variant	2.00E-18
GCST90179150	4	111792280	RPL36AP23	rs326889	intergenic_variant	4.00E-12
GCST90179150	4	161208692	RPS14P7 - FSTL5	rs13110266	intergenic_variant	2.00E-14
GCST009004	4	161208692	RPS14P7 - FSTL5	rs13110266	intergenic_variant	4.00E-14
GCST009003	4	161208692	RPS14P7 - FSTL5	rs13110266	intergenic_variant	4.00E-14
GCST90255621	4	161208692	RPS14P7 - FSTL5	rs13110266	intergenic_variant	1.00E-13
GCST007039	4	161170487	RPS14P7 - FSTL5	rs6536575	intergenic_variant	6.00E-12
GCST009871	4	161208692	RPS14P7 - FSTL5	rs13110266	intergenic_variant	2.00E-11
GCST90255621	4	76174965	SCARB2	rs17001561	non_coding_transcript_exon_	1.00E-12
GCST005951	4	76208415	SCARB2	rs17001654	intron_variant	5.00E-12
GCST90255621	4	146455424	SLC10A7	rs7663212	intron_variant	1.00E-14
GCST009004	4	146516867	SLC10A7	rs3914628	intron_variant	7.00E-13
GCST90179150	4	146516867	SLC10A7	rs3914628	intron_variant	2.00E-12
GCST009871	4	146432937	SLC10A7	rs113079574	intron_variant	5.00E-12
GCST007039	4	146432937	SLC10A7	rs113079574	intron_variant	6.00E-12
GCST002830	4	9925343	SLC2A9	rs13129697	intron_variant	8.00E-150
GCST002830	4	9983972	SLC2A9	rs7680126	intron_variant	2.00E-134
GCST002829	4	9925343	SLC2A9	rs13129697	intron_variant	3.00E-111
GCST002829	4	9983972	SLC2A9	rs7680126	intron_variant	3.00E-92
GCST002828	4	9983972	SLC2A9	rs7680126	intron_variant	6.00E-62
GCST002828	4	9925343	SLC2A9	rs13129697	intron_variant	3.00E-59
GCST002829	4	9918723	SLC2A9	rs10805346	intron_variant	1.00E-31
GCST002830	4	9920543	SLC2A9	rs16890979	missense_variant	1.00E-29
GCST002828	4	9918723	SLC2A9	rs10805346	intron_variant	4.00E-14
GCST90255621	4	102267552	SLC39A8	rs13107325	missense_variant	2.00E-94
GCST90271771	4	102267552	SLC39A8	rs13107325	missense_variant	2.00E-52
GCST90271767	4	102267552	SLC39A8	rs13107325	missense_variant	2.00E-51
GCST90271770	4	102267552	SLC39A8	rs13107325	missense_variant	2.00E-48
GCST009004	4	102267552	SLC39A8	rs13107325	missense_variant	4.00E-47
GCST009001	4	102267552	SLC39A8	rs13107325	missense_variant	4.00E-47
GCST009003	4	102267552	SLC39A8	rs13107325	missense_variant	4.00E-47
GCST90179150	4	102267552	SLC39A8	rs13107325	missense_variant	4.00E-46
GCST008129	4	102267552	SLC39A8	rs13107325	missense_variant	5.00E-40
GCST009871	4	102267552	SLC39A8	rs13107325	missense_variant	8.00E-39
GCST007039	4	102267552	SLC39A8	rs13107325	missense_variant	1.00E-36
GCST90271769	4	102267552	SLC39A8	rs13107325	missense_variant	2.00E-31
GCST90018947	4	102267552	SLC39A8	rs13107325	missense_variant	1.00E-28
GCST90271768	4	102267552	SLC39A8	rs13107325	missense_variant	1.00E-25
GCST011331	4	102267552	SLC39A8	rs13107325	missense_variant	2.00E-22
GCST006368	4	102267552	SLC39A8	rs13107325	missense_variant	3.00E-21
GCST005951	4	102267552	SLC39A8	rs13107325	missense_variant	4.00E-16
GCST009871	4	102292205	SLC39A8	rs173048	intron_variant	2.00E-15

GCST90255621	4	102312050	SLC39A8	rs6822371	intron_variant	5.00E-14
GCST000830	4	102267552	SLC39A8	rs13107325	missense_variant	2.00E-13
GCST90255621	4	102421010	SLC39A8	rs146171187	intron_variant	2.00E-13
GCST002783	4	102267552	SLC39A8	rs13107325	missense_variant	1.00E-12
GCST002783	4	102267552	SLC39A8	rs13107325	missense_variant	2.00E-12
GCST90255621	4	102271086	SLC39A8	rs9992479	intron_variant	5.00E-12
GCST007039	4	20119240	SLIT2	rs1485554	intergenic_variant	3.00E-16
GCST90255621	4	20119240	SLIT2	rs1485554	intergenic_variant	3.00E-16
GCST009871	4	20119240	SLIT2	rs1485554	intergenic_variant	2.00E-15
GCST009004	4	20217972	SLIT2	rs73249175	intergenic_variant	2.00E-14
GCST90179150	4	20230101	SLIT2	rs55745630	intergenic_variant	5.00E-14
GCST009121	4	20212158	SLIT2	rs10016841	intergenic_variant	2.00E-13
GCST009003	4	20238040	SLIT2	rs28608644	intergenic_variant	1.00E-12
GCST90255621	4	20258405	SLIT2	rs2322459	intron_variant	2.00E-12
GCST90255621	4	136162038	TERF1P3	rs1296328	intron_variant	3.00E-30
GCST007039	4	136162038	TERF1P3	rs1296328	intron_variant	7.00E-23
GCST90179150	4	136162038	TERF1P3	rs1296328	intron_variant	4.00E-21
GCST006368	4	136127444	TERF1P3	rs10019997	intron_variant	7.00E-21
GCST009004	4	136150180	TERF1P3	rs1451109	intron_variant	1.00E-19
GCST009001	4	136150180	TERF1P3	rs1451109	intron_variant	1.00E-19
GCST009871	4	136150180	TERF1P3	rs1451109	intron_variant	4.00E-18
GCST90018947	4	136051386	TERF1P3	rs10018743	intergenic_variant	3.00E-14
GCST90267268	4	136051386	TERF1P3	rs10018743	intergenic_variant	2.00E-12
GCST009871	4	136285033	TERF1P3	rs1597598	intron_variant	2.00E-11
GCST90179150	4	95170373	UNC5C	rs2241743	intron_variant	6.00E-11
GCST009004	4	95170373	UNC5C	rs2241743	intron_variant	9.00E-11
GCST90255621	4	95232063	UNC5C	rs34656389	intron_variant	9.00E-11
GCST009871	4	25340984	ZCCHC4	rs56203712	intron_variant	5.00E-31
GCST90267268	4	25340984	ZCCHC4	rs56203712	intron_variant	3.00E-15
GCST009001	5	75671883	ANAPC4	rs34360	intron_variant	2.00E-56
GCST009871	5	75656076	ANKDD1B	rs7717355	synonymous_variant	3.00E-13
GCST90179150	5	75649551	ANKDD1B	rs115828485	intron_variant	5.00E-12
GCST009871	5	75279976	ANKRD26	rs1115091	intergenic_variant	3.00E-19
GCST90267268	5	75177114	ANKRD31	rs7707394	intron_variant	5.00E-14
GCST004046	5	75329662	ANKRD31	rs7703051	intron_variant	4.00E-12
GCST004045	5	75329662	ANKRD31	rs7703051	intron_variant	9.00E-12
GCST009871	5	75101621	ANKRD31	rs112386051	intron_variant	3.00E-11
GCST90255621	5	112841059	ANTXR2	rs459552	missense_variant	7.00E-12
GCST009004	5	112841059	APC	rs459552	missense_variant	8.00E-12
GCST009003	5	112841059	APC	rs459552	missense_variant	8.00E-12
GCST90255621	5	53976834	ARL14EP	rs4865796	intron_variant	2.00E-12
GCST011336	5	54002932	ARL15	rs3776717	intron_variant	7.00E-11
GCST009871	5	75895625	BDNF	rs4704262	intergenic_variant	1.00E-13
GCST90255621	5	75948297	BIN2P2	rs1404990	intergenic_variant	5.00E-12
GCST90179150	5	75888595	BIN2P2	rs1404989	intergenic_variant	3.00E-11
GCST90255621	5	75888595	BIN2P2	rs1404989	intergenic_variant	3.00E-11
GCST009871	5	75850950	BIN2P2	rs28433586	intergenic_variant	5.00E-11
GCST004904	5	96532204	CAST	rs10062657	intron_variant	4.00E-25
GCST90255621	5	96766240	CAST	rs27524	intron_variant	6.00E-21
GCST009001	5	96525308	CAST	rs11951673	non_coding_transcript_exon_	6.00E-14
GCST90255621	5	96579014	CAST	rs8180490	intron_variant	3.00E-13

GCST90131907	5	96526493	CAST	rs261969	intron_variant	5.00E-13
GCST90255621	5	123397623	CEP120	rs7711753	intron_variant	4.00E-18
GCST90018947	5	123316412	CEP120	rs4308481	intergenic_variant	7.00E-18
GCST90179150	5	123422024	CEP120	rs11953651	intron_variant	7.00E-14
GCST009004	5	123321505	CEP120	rs1582931	intergenic_variant	2.00E-13
GCST007039	5	123321505	CEP120	rs1582931	intergenic_variant	6.00E-13
GCST009871	5	123316412	CEP120	rs4308481	intergenic_variant	3.00E-12
GCST90255621	5	75460396	CERT1	rs4512110	intron_variant	2.00E-17
GCST004046	5	75463358	CERT1	rs4704221	intron_variant	5.00E-11
GCST90255621	5	173935455	CPEB4	rs6861681	intron_variant	8.00E-15
GCST90018947	5	173907398	CPEB4	rs4867732	intron_variant	4.00E-11
GCST90179150	5	173910850	CPEB4	rs17695092	intron_variant	7.00E-11
GCST009871	5	64780688	CWC27	rs9291822	intron_variant	6.00E-15
GCST009004	5	64791196	CWC27	rs12522567	intron_variant	2.00E-13
GCST007039	5	64780688	CWC27	rs9291822	intron_variant	2.00E-13
GCST009003	5	64778903	CWC27	rs7701842	intron_variant	3.00E-13
GCST90271767	5	64942904	CWC27	rs11738728	intron_variant	2.00E-12
GCST90255621	5	64791196	CWC27	rs12522567	intron_variant	4.00E-11
GCST009004	5	139701160	CXXC5	rs13174863	intron_variant	2.00E-17
GCST009003	5	139701160	CXXC5	rs13174863	intron_variant	2.00E-17
GCST90179150	5	139701160	CXXC5	rs13174863	intron_variant	3.00E-17
GCST90255621	5	139707066	CXXC5	rs2133561	intron_variant	1.00E-16
GCST90018947	5	139690813	CXXC5	rs71579590	intron_variant	8.00E-16
GCST009871	5	139701160	CXXC5	rs13174863	intron_variant	4.00E-15
GCST007039	5	139701160	CXXC5	rs13174863	intron_variant	9.00E-15
GCST90255621	5	153978666	FAM114A2	rs17115481	intergenic_variant	3.00E-17
GCST009871	5	153978666	FAM114A2	rs17115481	intergenic_variant	2.00E-11
GCST90179150	5	153978666	FAM114A2	rs17115481	intergenic_variant	2.00E-11
GCST90255621	5	108103311	FBXL17	rs40067	intron_variant	1.00E-31
GCST90179150	5	108093085	FBXL17	rs34415	intron_variant	8.00E-28
GCST009004	5	108086366	FBXL17	rs288230	intron_variant	5.00E-26
GCST009001	5	108086366	FBXL17	rs288230	intron_variant	5.00E-26
GCST007039	5	108102356	FBXL17	rs149457	intron_variant	2.00E-24
GCST009871	5	108140278	FBXL17	rs185361	intron_variant	3.00E-23
GCST009003	5	107981162	FBXL17	rs2916577	intron_variant	7.00E-22
GCST90018947	5	108142979	FBXL17	rs10623997	intron_variant	5.00E-19
GCST90179150	5	107988894	FBXL17	rs17438698	intron_variant	3.00E-14
GCST90255621	5	107988165	FBXL17	rs12514413	intron_variant	1.00E-13
GCST009871	5	108001645	FBXL17	rs12513616	intron_variant	5.00E-11
GCST90255621	5	177100576	FGFR4 - NSD1	rs6556301	intergenic_variant	5.00E-16
GCST90179150	5	177100576	FGFR4 - NSD1	rs6556301	intergenic_variant	3.00E-11
GCST009004	5	177100576	FGFR4 - NSD1	rs6556301	intergenic_variant	8.00E-11
GCST90255621	5	75044502	GCNT4	rs7714420	intron_variant	3.00E-22
GCST009871	5	75048667	GCNT4	rs73125247	intron_variant	2.00E-21
GCST90179150	5	75047944	GCNT4	rs7710706	5_prime_UTR_variant	8.00E-15
GCST90255621	5	140334979	HBEGF	rs2074613	non_coding_transcript_exon_	7.00E-13
GCST90179150	5	140332654	HBEGF	rs2282802	intergenic_variant	2.00E-11
GCST009871	5	140334979	HBEGF	rs2074613	non_coding_transcript_exon_	6.00E-11
GCST90255621	5	51618892	HMGB1P47	rs12189178	intron_variant	2.00E-18
GCST009004	5	51618892	HMGB1P47	rs12189178	intron_variant	4.00E-14
GCST90179150	5	51618892	HMGB1P47	rs12189178	intron_variant	2.00E-13

GCST90255621	5	51405916	HMGB1P47	rs6874700	intergenic_variant	1.00E-11
GCST007039	5	51661610	HMGB1P47	rs79125854	intron_variant	3.00E-11
GCST009871	5	51661610	HMGB1P47	rs79125854	intron_variant	4.00E-11
GCST90255621	5	63724879	HTR1A	rs1503526	intergenic_variant	5.00E-21
GCST009004	5	63730453	HTR1A	rs4700608	intergenic_variant	4.00E-20
GCST90179150	5	63724879	HTR1A	rs1503526	intergenic_variant	4.00E-20
GCST009003	5	63725811	HTR1A	rs1895407	intergenic_variant	5.00E-20
GCST009871	5	63738779	HTR1A	rs10805383	intergenic_variant	4.00E-14
GCST007039	5	63726237	HTR1A	rs1895408	intergenic_variant	8.00E-14
GCST90255621	5	64017101	HTR1A	rs6873465	intergenic_variant	7.00E-12
GCST90018947	5	134525973	JADE2	rs329118	5_prime_UTR_variant	5.00E-20
GCST90255621	5	134528909	JADE2	rs329122	intron_variant	3.00E-16
GCST009871	5	134529762	JADE2	rs329124	intron_variant	6.00E-16
GCST90179150	5	134528909	JADE2	rs329122	intron_variant	6.00E-16
GCST007039	5	134529762	JADE2	rs329124	intron_variant	2.00E-15
GCST009004	5	134529762	JADE2	rs329124	intron_variant	4.00E-15
GCST009003	5	134526066	JADE2	rs329120	5_prime_UTR_variant	5.00E-15
GCST90018947	5	88672529	LINC00461	rs35991856	intron_variant	5.00E-34
GCST90179150	5	88667943	LINC00461	rs1501672	intron_variant	1.00E-31
GCST009004	5	88667782	LINC00461	rs1501673	intron_variant	3.00E-31
GCST004904	5	88674109	LINC00461	rs1846974	intron_variant	2.00E-15
GCST90255621	5	88572347	LINC00461	rs34142155	intron_variant	5.00E-15
GCST90267268	5	88647802	LINC00461	rs13163173	intron_variant	4.00E-13
GCST90255621	5	87003253	LINC02059 - MIR4280HG	rs4920782	intron_variant	8.00E-12
GCST90255621	5	86791814	LINC02059 - MIR4280HG	rs2112457	intergenic_variant	1.00E-11
GCST90255621	5	124994829	LINC02240	rs6864049	intron_variant	5.00E-16
GCST009004	5	124994829	LINC02240	rs6864049	intron_variant	1.00E-13
GCST90179150	5	124994829	LINC02240	rs6864049	intron_variant	2.00E-13
GCST005951	5	124994829	LINC02240	rs6864049	intron_variant	3.00E-11
GCST009004	5	66904955	MAST4	rs249612	intron_variant	5.00E-13
GCST009003	5	66904955	MAST4	rs249612	intron_variant	5.00E-13
GCST90179150	5	66904955	MAST4	rs249612	intron_variant	6.00E-13
GCST90255621	5	66896143	MAST4	rs460799	intron_variant	1.00E-12
GCST009871	5	66904955	MAST4	rs249612	intron_variant	4.00E-12
GCST007039	5	66904955	MAST4	rs249612	intron_variant	4.00E-11
GCST90255621	5	88690467	MEF2C	rs7733438	intron_variant	8.00E-42
GCST007039	5	88693117	MEF2C	rs1477290	non_coding_transcript_exon_	9.00E-36
GCST009871	5	88693117	MEF2C	rs1477290	non_coding_transcript_exon_	1.00E-34
GCST009001	5	88690467	MEF2C	rs7733438	intron_variant	4.00E-31
GCST009003	5	88749212	MEF2C	rs10514303	intron_variant	8.00E-26
GCST90255621	5	89520899	MEF2C	rs12514792	intergenic_variant	9.00E-18
GCST009871	5	89502556	MEF2C	rs55814654	intergenic_variant	2.00E-16
GCST004904	5	88682435	MEF2C	rs16903285	non_coding_transcript_exon_	6.00E-16
GCST007039	5	89502556	MEF2C	rs55814654	intergenic_variant	7.00E-16
GCST90179150	5	89512777	MEF2C	rs12652212	intergenic_variant	2.00E-15
GCST90018727	5	88682435	MEF2C	rs16903285	non_coding_transcript_exon_	2.00E-15
GCST009871	5	88765439	MEF2C	rs76564347	intron_variant	2.00E-15
GCST90255621	5	88765439	MEF2C	rs76564347	intron_variant	2.00E-14
GCST90255621	5	154158333	MFAP3	rs7715256	non_coding_transcript_exon_	1.00E-30
GCST90179150	5	154158333	MFAP3	rs7715256	non_coding_transcript_exon_	4.00E-23
GCST009004	5	154167248	MFAP3	rs10044136	intron_variant	3.00E-21

GCST009001	5	154167935	MFAP3	rs1428121	intron_variant	3.00E-21
GCST009003	5	154167849	MFAP3	rs7701886	intron_variant	5.00E-21
GCST90255622	5	154160465	MFAP3	rs4569924	splice_polypyrimidine_tract_	1.00E-17
GCST007039	5	154164952	MFAP3	rs4958702	intron_variant	8.00E-17
GCST004519	5	154163906	MFAP3	rs7708584	intron_variant	4.00E-15
GCST004519	5	154163906	MFAP3	rs7708584	intron_variant	4.00E-14
GCST001967	5	154163906	MFAP3	rs7708584	intron_variant	5.00E-14
GCST006368	5	154137618	MFAP3	rs815610	intron_variant	2.00E-12
GCST005951	5	154137618	MFAP3	rs815610	intron_variant	4.00E-12
GCST90018947	5	154158333	MFAP3	rs7715256	non_coding_transcript_exon_	1.00E-11
GCST90255621	5	154167746	MFAP3	rs11957885	intron_variant	3.00E-11
GCST90255621	5	145104698	NAMPTP2 - ASS1P10	rs2190788	intron_variant	2.00E-14
GCST90179150	5	145104698	NAMPTP2 - ASS1P10	rs2190788	intron_variant	2.00E-13
GCST90054786	5	140735758	PCDHA1	rs801170	intergenic_variant	9.00E-13
GCST90054789	5	140735758	PCDHA1	rs801170	intergenic_variant	9.00E-13
GCST90018947	5	96520797	PCSK1	rs2611742	intron_variant	7.00E-30
GCST90255621	5	96520797	PCSK1	rs2611742	intron_variant	3.00E-29
GCST90018727	5	96520797	PCSK1	rs2611742	intron_variant	1.00E-27
GCST90255621	5	96393270	PCSK1	rs6234	missense_variant	2.00E-25
GCST004904	5	96522964	PCSK1	rs4869139	intron_variant	8.00E-24
GCST009004	5	96381018	PCSK1	rs7713317	intron_variant	2.00E-20
GCST009003	5	96381018	PCSK1	rs7713317	intron_variant	2.00E-20
GCST009001	5	96393194	PCSK1	rs6235	missense_variant	4.00E-20
GCST90179150	5	96381018	PCSK1	rs7713317	intron_variant	8.00E-20
GCST90179150	5	96523440	PCSK1	rs1837269	intron_variant	1.00E-18
GCST008129	5	96393270	PCSK1	rs6234	missense_variant	3.00E-17
GCST007039	5	96393194	PCSK1	rs6235	missense_variant	1.00E-15
GCST009871	5	96393194	PCSK1	rs6235	missense_variant	9.00E-15
GCST90255621	5	96458718	PCSK1	rs7716123	intron_variant	2.00E-14
GCST90255621	5	96058179	PCSK1	rs17383803	intron_variant	4.00E-14
GCST002461	5	96514546	PCSK1	rs261967	intron_variant	8.00E-13
GCST007039	5	96520797	PCSK1	rs2611742	intron_variant	2.00E-12
GCST009871	5	96523440	PCSK1	rs1837269	intron_variant	1.00E-11
GCST90104632	5	96315271	PCSK1	rs6899303	intron_variant	5.00E-11
GCST90179150	5	96416081	PCSK1	rs6232	missense_variant	5.00E-11
GCST90255621	5	75719417	POC5	rs2112347	intergenic_variant	4.00E-77
GCST009004	5	75719417	POC5	rs2112347	intergenic_variant	1.00E-61
GCST009003	5	75719417	POC5	rs2112347	intergenic_variant	1.00E-61
GCST90179150	5	75707853	POC5	rs2307111	missense_variant	8.00E-60
GCST007039	5	75707853	POC5	rs2307111	missense_variant	1.00E-46
GCST90018947	5	75707853	POC5	rs2307111	missense_variant	1.00E-44
GCST009871	5	75714177	POC5	rs6864091	intron_variant	9.00E-44
GCST008129	5	75707853	POC5	rs2307111	missense_variant	9.00E-32
GCST004904	5	75719417	POC5	rs2112347	intergenic_variant	8.00E-27
GCST006368	5	75719417	POC5	rs2112347	intergenic_variant	3.00E-26
GCST005951	5	75719417	POC5	rs2112347	intergenic_variant	8.00E-18
GCST002783	5	75719417	POC5	rs2112347	intergenic_variant	2.00E-17
GCST002783	5	75719417	POC5	rs2112347	intergenic_variant	6.00E-17
GCST004495	5	75719417	POC5	rs2112347	intergenic_variant	1.00E-13
GCST90018727	5	75719417	POC5	rs2112347	intergenic_variant	1.00E-13
GCST000830	5	75719417	POC5	rs2112347	intergenic_variant	2.00E-13

GCST002783	5	75719417	POC5	rs2112347	intergenic_variant	3.00E-13
GCST004904	5	75696024	POC5	rs6881648	intron_variant	2.00E-12
GCST004497	5	75719417	POC5	rs2112347	intergenic_variant	2.00E-12
GCST004499	5	75719417	POC5	rs2112347	intergenic_variant	6.00E-12
GCST90255621	5	93040754	POLD2P1	rs2453763	intron_variant	4.00E-13
GCST90179150	5	93052504	POLD2P1	rs1845840	intron_variant	1.00E-11
GCST006368	5	171032671	RANBP17	rs7730898	intron_variant	7.00E-22
GCST90179150	5	171172323	RANBP17	rs2053682	intron_variant	9.00E-21
GCST90255621	5	171032671	RANBP17	rs7730898	intron_variant	2.00E-20
GCST009004	5	171172323	RANBP17	rs2053682	intron_variant	3.00E-20
GCST009003	5	171172323	RANBP17	rs2053682	intron_variant	3.00E-20
GCST009001	5	171032671	RANBP17	rs7730898	intron_variant	7.00E-20
GCST009871	5	171068154	RANBP17	rs779655	intron_variant	8.00E-18
GCST007039	5	171102331	RANBP17	rs245769	intron_variant	1.00E-17
GCST90018947	5	171105101	RANBP17	rs245775	intron_variant	8.00E-16
GCST009871	5	64671495	RGS7BP	rs12518987	intergenic_variant	8.00E-14
GCST90255621	5	64671403	RGS7BP	rs12523594	intergenic_variant	1.00E-13
GCST90018947	5	64644435	RGS7BP	rs12697021	intergenic_variant	3.00E-13
GCST90179150	5	64646571	RGS7BP	rs10461497	regulatory_region_variant	3.00E-11
GCST007039	5	64636407	RGS7BP	rs6888159	intergenic_variant	4.00E-11
GCST90255622	5	64646368	RGS7BP	rs7726614	regulatory_region_variant	5.00E-11
GCST90179150	5	87431749	RNU6-727P - LINC02488	rs11951885	intron_variant	2.00E-12
GCST009871	5	87463896	RNU6-727P - LINC02488	rs323759	intron_variant	3.00E-11
GCST007039	5	87431749	RNU6-727P - LINC02488	rs11951885	intron_variant	3.00E-11
GCST90255621	5	87473198	RNU6-727P - LINC02488	rs323742	intron_variant	5.00E-11
GCST90255621	5	151666362	SPARC	rs41290587	missense_variant	1.00E-21
GCST90094398	5	151666362	SPARC	rs41290587	missense_variant	1.00E-14
GCST009004	5	151666362	SPARC	rs41290587	missense_variant	3.00E-11
GCST009004	5	81546095	SSBP2	rs10942267	intron_variant	7.00E-16
GCST009003	5	81546095	SSBP2	rs10942267	intron_variant	7.00E-16
GCST90179150	5	81546095	SSBP2	rs10942267	intron_variant	9.00E-16
GCST90255621	5	81522820	SSBP2	rs12514473	intron_variant	1.00E-15
GCST009871	5	81534969	SSBP2	rs59893724	intron_variant	3.00E-15
GCST007039	5	81534969	SSBP2	rs59893724	intron_variant	6.00E-15
GCST90018947	5	81534969	SSBP2	rs59893724	intron_variant	2.00E-14
GCST90255621	5	81835609	SSBP2	rs10514222	intron_variant	7.00E-11
GCST009871	5	87808076	TMEM161B	rs73167519	intergenic_variant	1.00E-11
GCST007039	5	88434210	TMEM161B-DT	rs7444298	non_coding_transcript_exon_	5.00E-18
GCST90255621	5	88401716	TMEM161B-DT	rs6870983	intron_variant	9.00E-34
GCST90179150	5	88401716	TMEM161B-DT	rs6870983	intron_variant	1.00E-26
GCST009871	5	88469841	TMEM161B-DT	rs9293499	intron_variant	8.00E-13
GCST006368	5	88401716	TMEM161B-DT	rs6870983	intron_variant	9.00E-12
GCST90255621	5	120036838	TUBAP15	rs6595205	intergenic_variant	5.00E-15
GCST009004	5	120052964	TUBAP15	rs4895231	intergenic_variant	2.00E-12
GCST009003	5	120052964	TUBAP15	rs4895231	intergenic_variant	2.00E-12
GCST90179150	5	120032971	TUBAP15	rs351114	intergenic_variant	3.00E-11
GCST007039	5	120053336	TUBAP15	rs347551	intergenic_variant	7.00E-11
GCST90255621	5	43143697	ZNF131	rs6892422	intron_variant	6.00E-19
GCST90255621	5	43161249	ZNF131	rs71627581	missense_variant	3.00E-17
GCST007039	5	43152114	ZNF131	rs13176429	intron_variant	1.00E-14
GCST009004	5	43110753	ZNF131	rs6451675	intron_variant	3.00E-14

GCST90179150	5	43190931	ZNF131	rs7730004	intron_variant	3.00E-14
GCST009871	5	43152114	ZNF131	rs13176429	intron_variant	5.00E-14
GCST009003	5	43124586	ZNF131	rs782971	non_coding_transcript_exon_	4.00E-13
GCST90018947	5	43190760	ZNF131	rs11444441	intron_variant	3.00E-12
GCST009871	5	43134408	ZNF131	rs512921	intron_variant	2.00E-11
GCST90255621	6	69546487	ADCY9	rs2757776	intergenic_variant	2.00E-11
GCST007039	6	69647476	ADGRB3	rs7761673	intergenic_variant	5.00E-11
GCST90267268	6	31617223	AIDAP3	rs2857597	intergenic_variant	3.00E-15
GCST90179150	6	35089554	ANKRD34C	rs3822921	3_prime_UTR_variant	4.00E-22
GCST009871	6	35082222	ANKS1A	rs56340485	intron_variant	8.00E-12
GCST009004	6	131577068	ARAP1	rs2246012	intron_variant	1.00E-13
GCST90179150	6	131576138	ARG1	rs2781668	intron_variant	2.00E-13
GCST90255621	6	100720563	ASB3	rs12194255	intron_variant	3.00E-14
GCST009004	6	100708930	ASCC3	rs12209887	intron_variant	2.00E-11
GCST007039	6	100679337	ASCC3	rs180963	intron_variant	2.00E-11
GCST90179150	6	100718933	ASCC3	rs7748644	intron_variant	3.00E-11
GCST009871	6	100679337	ASCC3	rs180963	intron_variant	5.00E-11
GCST009003	6	31645962	BAD, GPR137	rs3130048	intron_variant	2.00E-20
GCST009871	6	31645962	BAG6	rs3130048	intron_variant	1.00E-11
GCST90255621	6	34856859	BLC3	rs11755393	missense_variant	4.00E-55
GCST008129	6	34856859	BLTP3A	rs11755393	missense_variant	3.00E-26
GCST005951	6	34860776	BLTP3A	rs6457796	intron_variant	1.00E-14
GCST006368	6	34860776	BLTP3A	rs6457796	intron_variant	5.00E-13
GCST90018727	6	20693467	CDKAL1	rs138420022	intron_variant	1.00E-30
GCST90018947	6	20682553	CDKAL1	rs60229538	intron_variant	1.00E-29
GCST004904	6	20675561	CDKAL1	rs35261542	intron_variant	4.00E-29
GCST004904	6	20686765	CDKAL1	rs9368222	intron_variant	8.00E-22
GCST90255621	6	20694653	CDKAL1	rs2206734	intron_variant	2.00E-21
GCST002461	6	20685255	CDKAL1	rs9356744	intron_variant	5.00E-13
GCST001416	6	20694653	CDKAL1	rs2206734	intron_variant	1.00E-11
GCST001415	6	20685255	CDKAL1	rs9356744	intron_variant	2.00E-11
GCST90271770	6	20705359	CDKAL1	rs11753081	intron_variant	2.00E-11
GCST009871	6	50690562	DEFB112 - TFAP2D	rs72885809	intergenic_variant	3.00E-18
GCST90255621	6	50395504	DEFB112 - TFAP2D	rs1492627	intergenic_variant	8.00E-18
GCST90179150	6	50665668	DEFB112 - TFAP2D	rs280322	intergenic_variant	5.00E-17
GCST90179150	6	50393299	DEFB112 - TFAP2D	rs6900872	intergenic_variant	3.00E-16
GCST90179150	6	50634499	DEFB112 - TFAP2D	rs10948567	intron_variant	8.00E-16
GCST009871	6	50665668	DEFB112 - TFAP2D	rs280322	intergenic_variant	5.00E-15
GCST009871	6	50393299	DEFB112 - TFAP2D	rs6900872	intergenic_variant	5.00E-15
GCST90255621	6	50518248	DEFB112 - TFAP2D	rs75397901	intron_variant	5.00E-14
GCST90255621	6	50516777	DEFB112 - TFAP2D	rs1342228	intron_variant	6.00E-14
GCST009871	6	50634499	DEFB112 - TFAP2D	rs10948567	intron_variant	8.00E-14
GCST90255621	6	50558576	DEFB112 - TFAP2D	rs280346	intergenic_variant	1.00E-12
GCST90267268	6	50690562	DEFB112 - TFAP2D	rs72885809	intergenic_variant	6.00E-12
GCST90179150	6	50663913	DEFB112 - TFAP2D	rs75604722	intergenic_variant	1.00E-11
GCST90255621	6	50687601	DEFB112 - TFAP2D	rs280329	intergenic_variant	2.00E-11
GCST90255621	6	50522703	DEFB112 - TFAP2D	rs1342224	intergenic_variant	7.00E-11
GCST009871	6	20488666	E2F3	rs1322842	intron_variant	5.00E-12
GCST009004	6	20482104	E2F3	rs3806114	intron_variant	1.00E-11
GCST009003	6	20482104	E2F3	rs3806114	intron_variant	1.00E-11
GCST007039	6	20488666	E2F3	rs1322842	intron_variant	2.00E-11

GCST90179150	6	20482104	E2F3	rs3806114	intron_variant	5.00E-11
GCST006368	6	98091643	EIF4EBP2P3	rs901630	intron_variant	1.00E-25
GCST009003	6	98091643	EIF4EBP2P3	rs901630	intron_variant	2.00E-21
GCST90255621	6	98091643	EIF4EBP2P3	rs901630	intron_variant	6.00E-21
GCST009871	6	98109856	EIF4EBP2P3	rs56081191	intron_variant	2.00E-11
GCST90255621	6	108554799	FOXO3	rs768023	intron_variant	2.00E-24
GCST90179150	6	108554799	FOXO3	rs768023	intron_variant	8.00E-23
GCST007039	6	108567390	FOXO3	rs2253310	intron_variant	8.00E-20
GCST90018947	6	108605293	FOXO3	rs2802295	intron_variant	5.00E-18
GCST009004	6	108584997	FOXO3	rs12206094	intron_variant	2.00E-15
GCST009003	6	108676925	FOXO3	rs3800230	intron_variant	4.00E-15
GCST006368	6	108656460	FOXO3	rs9400239	5_prime_UTR_variant	5.00E-14
GCST004904	6	108675760	FOXO3	rs3800229	intron_variant	4.00E-13
GCST009871	6	108544460	FOXO3	rs6927268	intron_variant	6.00E-13
GCST009871	6	108584997	FOXO3	rs12206094	intron_variant	1.00E-12
GCST90255621	6	33565627	GGNBP1	rs210130	intron_variant	3.00E-16
GCST90255621	6	33513427	GGNBP1	rs210192	regulatory_region_variant	4.00E-13
GCST90275044	6	26092913	H2BC4	rs1800562	missense_variant	3.00E-16
GCST90255621	6	26099795	H2BC4	rs1150659	intron_variant	4.00E-16
GCST90255621	6	26119017	H2BC4	rs13206443	intron_variant	2.00E-11
GCST90255621	6	125769131	HEY2 - NCOA7	rs1159974	intergenic_variant	3.00E-16
GCST007039	6	125769131	HEY2 - NCOA7	rs1159974	intergenic_variant	2.00E-11
GCST90255621	6	12124622	HIVEP1	rs2228213	missense_variant	7.00E-24
GCST90179150	6	12124622	HIVEP1	rs2228213	missense_variant	4.00E-17
GCST009003	6	12124622	HIVEP1	rs2228213	missense_variant	5.00E-17
GCST009001	6	12124622	HIVEP1	rs2228213	missense_variant	5.00E-17
GCST009004	6	12124622	HIVEP1	rs2228213	missense_variant	6.00E-17
GCST008129	6	12124622	HIVEP1	rs2228213	missense_variant	3.00E-13
GCST007039	6	12142584	HIVEP1	rs10947793	intron_variant	1.00E-12
GCST90018947	6	12086593	HIVEP1	rs4467770	intron_variant	5.00E-12
GCST009871	6	12142584	HIVEP1	rs10947793	intron_variant	7.00E-12
GCST009004	6	142864546	HIVEP2	rs765875	intron_variant	1.00E-14
GCST90179150	6	142864754	HIVEP2	rs765876	intron_variant	2.00E-14
GCST90255621	6	142864546	HIVEP2	rs765875	intron_variant	3.00E-14
GCST009003	6	142833749	HIVEP2	rs198665	intron_variant	5.00E-13
GCST007039	6	142864546	HIVEP2	rs765875	intron_variant	9.00E-11
GCST90054786	6	32625831	HLA-DRB1	rs9271730	intergenic_variant	8.00E-15
GCST90054789	6	32625831	HLA-DRB1	rs9271730	intergenic_variant	2.00E-14
GCST90018727	6	32587454	HLA-DRB1	rs28724141	intron_variant	4.00E-12
GCST004904	6	34211613	HMGA1	rs6913361	intergenic_variant	3.00E-14
GCST004904	6	34221835	HMGA1	rs10947487	regulatory_region_variant	1.00E-13
GCST90255621	6	34240996	HMGA1	rs41269026	intron_variant	5.00E-12
GCST90018727	6	34217972	HMGA1	rs2797963	intergenic_variant	7.00E-12
GCST007039	6	34721169	ILRUN	rs9366863	intergenic_variant	2.00E-44
GCST009004	6	34709326	ILRUN	rs6932930	regulatory_region_variant	1.00E-40
GCST009001	6	34657434	ILRUN	rs2814998	intron_variant	3.00E-40
GCST009003	6	34617174	ILRUN	rs2744968	non_coding_transcript_exon_	6.00E-40
GCST009871	6	34667176	ILRUN	rs2744948	intron_variant	9.00E-36
GCST90018947	6	34693672	ILRUN	rs9368828	intron_variant	1.00E-34
GCST90271770	6	34740117	ILRUN	rs10947525	regulatory_region_variant	7.00E-19
GCST90271767	6	34740117	ILRUN	rs10947525	regulatory_region_variant	3.00E-17

GCST009871	6	34591820	ILRUN	rs74929281	intron_variant	4.00E-17
GCST005951	6	34595387	ILRUN	rs205262	intron_variant	2.00E-14
GCST006368	6	34595387	ILRUN	rs205262	intron_variant	8.00E-13
GCST006368	6	73028938	KCNQ5	rs947612	intron_variant	2.00E-13
GCST90255621	6	73032611	KCNQ5	rs6921533	intron_variant	9.00E-12
GCST90255621	6	130027974	L3MBTL3	rs6569648	intron_variant	1.00E-17
GCST90179150	6	130027974	L3MBTL3	rs6569648	intron_variant	4.00E-13
GCST006368	6	130027974	L3MBTL3	rs6569648	intron_variant	5.00E-12
GCST009871	6	40394284	LRFN2	rs9471333	intron_variant	2.00E-31
GCST007039	6	40394284	LRFN2	rs9471333	intron_variant	1.00E-30
GCST009003	6	40404179	LRFN2	rs1579557	intron_variant	2.00E-28
GCST009001	6	40401342	LRFN2	rs34045288	intron_variant	3.00E-28
GCST90179150	6	40394284	LRFN2	rs9471333	intron_variant	7.00E-28
GCST90018947	6	40441504	LRFN2	rs34298980	intron_variant	5.00E-26
GCST90255621	6	40393752	LRFN2	rs56084097	intron_variant	6.00E-17
GCST009871	6	40424630	LRFN2	rs13196792	intron_variant	3.00E-11
GCST90255621	6	40440241	LRFN2	rs6910503	intron_variant	6.00E-11
GCST90255621	6	89612518	LYRM2, ANKRD6	rs16882001	intron_variant	2.00E-12
GCST90255621	6	89586869	LYRM2, ANKRD6	rs9362662	intron_variant	6.00E-12
GCST009871	6	43636430	MAD2L1BP	rs35679149	missense_variant	2.00E-12
GCST007039	6	43636430	MAD2L1BP	rs35679149	missense_variant	2.00E-12
GCST009004	6	43636430	MAD2L1BP	rs35679149	missense_variant	7.00E-12
GCST009003	6	43636430	MAD2L1BP	rs35679149	missense_variant	7.00E-12
GCST90179150	6	43636430	MAD2L1BP	rs35679149	missense_variant	2.00E-11
GCST90255621	6	119892734	MAN1A1	rs2357760	intergenic_variant	3.00E-17
GCST009004	6	119892734	MAN1A1	rs2357760	intergenic_variant	2.00E-16
GCST009003	6	119892734	MAN1A1	rs2357760	intergenic_variant	2.00E-16
GCST90179150	6	119892734	MAN1A1	rs2357760	intergenic_variant	2.00E-16
GCST90255621	6	119187706	MAN1A1	rs9387640	intron_variant	3.00E-12
GCST90255621	6	119797302	MAN1A1	rs12208543	intergenic_variant	4.00E-11
GCST90255621	6	33806617	MLN	rs2894342	intergenic_variant	5.00E-23
GCST90255621	6	33829232	MLN	rs9461917	TF_binding_site_variant	3.00E-12
GCST90255621	6	33839314	MLN	rs6901216	intergenic_variant	2.00E-11
GCST007039	6	33803896	MLN	rs2281819	intron_variant	7.00E-11
GCST90179150	6	97963755	MMS22L	rs6905544	intron_variant	1.00E-21
GCST009004	6	97981461	MMS22L	rs9320823	intron_variant	2.00E-21
GCST007039	6	97973845	MMS22L	rs6938973	intron_variant	2.00E-20
GCST009871	6	97973845	MMS22L	rs6938973	intron_variant	3.00E-20
GCST009001	6	97951645	MMS22L	rs4548017	intron_variant	6.00E-20
GCST90255621	6	98018493	MMS22L	rs9385223	intron_variant	2.00E-18
GCST009004	6	97305347	MMS22L	rs13209872	intron_variant	2.00E-16
GCST90255621	6	97455798	MMS22L	rs200807	intron_variant	2.00E-16
GCST90179150	6	97306076	MMS22L	rs6909685	non_coding_transcript_exon_	3.00E-16
GCST90018947	6	97973980	MMS22L	rs397885265	intron_variant	3.00E-15
GCST009001	6	97344859	MMS22L	rs12663742	intron_variant	4.00E-15
GCST007039	6	97316423	MMS22L	rs9372414	intron_variant	5.00E-14
GCST90179150	6	97947329	MMS22L	rs9372649	intron_variant	7.00E-14
GCST009871	6	97947873	MMS22L	rs62420383	intron_variant	4.00E-13
GCST90255621	6	97731850	MMS22L	rs17057975	intron_variant	6.00E-13
GCST009871	6	97305347	MMS22L	rs13209872	intron_variant	2.00E-12
GCST009871	6	97731850	MMS22L	rs17057975	intron_variant	3.00E-12

GCST90255621	6	124603886	NKAIN2	rs2875762	intron_variant	5.00E-16
GCST009871	6	124603886	NKAIN2	rs2875762	intron_variant	4.00E-11
GCST007039	6	124603886	NKAIN2	rs2875762	intron_variant	6.00E-11
GCST009121	6	32209005	NOTCH4	rs3132947	intron_variant	9.00E-15
GCST009107	6	32209005	NOTCH4	rs3132947	intron_variant	2.00E-13
GCST90179150	6	104362208	NPM1P10 - HACE1	rs156126	intergenic_variant	3.00E-18
GCST009004	6	104369069	NPM1P10 - HACE1	rs1417665	intergenic_variant	5.00E-18
GCST009003	6	104369069	NPM1P10 - HACE1	rs1417665	intergenic_variant	5.00E-18
GCST009001	6	104369900	NPM1P10 - HACE1	rs17459015	intergenic_variant	6.00E-18
GCST90255621	6	104351132	NPM1P10 - HACE1	rs156151	intergenic_variant	8.00E-15
GCST009871	6	104342657	NPM1P10 - HACE1	rs270689	intergenic_variant	3.00E-14
GCST007039	6	104345319	NPM1P10 - HACE1	rs270694	intergenic_variant	4.00E-14
GCST90018947	6	104341917	NPM1P10 - HACE1	rs705638	intergenic_variant	4.00E-11
GCST009004	6	153988673	OPRM1	rs10499276	intergenic_variant	2.00E-13
GCST90179150	6	153988673	OPRM1	rs10499276	intergenic_variant	2.00E-13
GCST90255621	6	153988673	OPRM1	rs10499276	intergenic_variant	3.00E-13
GCST009871	6	154012048	OPRM1	rs9478496	intron_variant	3.00E-12
GCST007039	6	154012048	OPRM1	rs9478496	intron_variant	1.00E-11
GCST009871	6	34500990	PACSIN1	rs73405691	intron_variant	3.00E-20
GCST90255621	6	34530551	PACSIN1	rs41312309	missense_variant	1.00E-19
GCST007039	6	34448095	PACSIN1	rs77998125	regulatory_region_variant	2.00E-12
GCST009871	6	34443606	PACSIN1	rs80138699	intergenic_variant	6.00E-12
GCST009004	6	13180222	PHACTR1	rs11757278	intron_variant	7.00E-13
GCST009003	6	13189709	PHACTR1	rs9367369	intron_variant	2.00E-12
GCST009871	6	13178027	PHACTR1	rs9463511	intron_variant	2.00E-12
GCST007039	6	13183291	PHACTR1	rs9395520	non_coding_transcript_exon_	2.00E-12
GCST90179150	6	13191958	PHACTR1	rs9357634	intron_variant	6.00E-12
GCST90255621	6	13189043	PHACTR1	rs9367368	intron_variant	3.00E-11
GCST006368	6	50929538	PKHD1	rs4715210	regulatory_region_variant	2.00E-34
GCST90255621	6	51008732	PKHD1	rs9369992	intergenic_variant	3.00E-34
GCST90255621	6	51282222	PKHD1	rs4615388	intergenic_variant	9.00E-34
GCST009871	6	50988851	PKHD1	rs546979	intergenic_variant	8.00E-27
GCST90179150	6	51288190	PKHD1	rs2478879	intergenic_variant	8.00E-23
GCST009871	6	51280796	PKHD1	rs11751804	intergenic_variant	2.00E-21
GCST90255621	6	51322989	PKHD1	rs994270	intergenic_variant	6.00E-21
GCST90255621	6	51870815	PKHD1	rs2064871	intron_variant	3.00E-19
GCST004558	6	50929538	PKHD1	rs4715210	regulatory_region_variant	6.00E-17
GCST90179150	6	51603341	PKHD1	rs2784187	intron_variant	9.00E-17
GCST90255621	6	50980799	PKHD1	rs185848	intergenic_variant	7.00E-16
GCST007039	6	51322989	PKHD1	rs994270	intergenic_variant	2.00E-15
GCST009871	6	51322989	PKHD1	rs994270	intergenic_variant	3.00E-15
GCST90255621	6	51595356	PKHD1	rs2579989	intergenic_variant	4.00E-15
GCST90179150	6	51935104	PKHD1	rs9395747	intron_variant	1.00E-14
GCST90255622	6	50946635	PKHD1	rs72887147	intergenic_variant	4.00E-14
GCST90255621	6	51116988	PKHD1	rs72891110	intergenic_variant	5.00E-14
GCST009871	6	51622618	PKHD1	rs1414506	non_coding_transcript_exon_	9.00E-14
GCST009871	6	51933962	PKHD1	rs1884953	intron_variant	6.00E-13
GCST009871	6	51851917	PKHD1	rs9370068	intron_variant	8.00E-13
GCST007039	6	51872379	PKHD1	rs1321512	intron_variant	1.00E-12
GCST007039	6	51613025	PKHD1	rs2771011	intron_variant	3.00E-12
GCST90255621	6	51697987	PKHD1	rs62461297	intron_variant	1.00E-11

GCST009871	6	51550124	PKHD1	rs72904286	intergenic_variant	2.00E-11
GCST90255621	6	51619428	PKHD1	rs34548196	missense_variant	2.00E-11
GCST90255621	6	100162168	PRDX2P4 - SIM1	rs78677597	regulatory_region_variant	4.00E-15
GCST007039	6	100181202	PRDX2P4 - SIM1	rs57989773	intergenic_variant	2.00E-11
GCST90255621	6	162612318	PRKN	rs13191362	intron_variant	3.00E-23
GCST009004	6	162612318	PRKN	rs13191362	intron_variant	4.00E-21
GCST009001	6	162612318	PRKN	rs13191362	intron_variant	4.00E-21
GCST009003	6	162612318	PRKN	rs13191362	intron_variant	4.00E-21
GCST90179150	6	162612318	PRKN	rs13191362	intron_variant	5.00E-20
GCST007039	6	162588303	PRKN	rs36007635	intron_variant	7.00E-14
GCST009871	6	162588303	PRKN	rs36007635	intron_variant	1.00E-13
GCST004557	6	162612318	PRKN	rs13191362	intron_variant	2.00E-11
GCST005951	6	162612318	PRKN	rs13191362	intron_variant	7.00E-11
GCST90255621	6	42707245	PRPH2	rs9357402	intron_variant	8.00E-15
GCST009004	6	42710096	PRPH2	rs6931385	intron_variant	6.00E-11
GCST009004	6	46395820	RCAN2	rs10498767	intron_variant	5.00E-16
GCST009003	6	46407589	RCAN2	rs2153615	intron_variant	9.00E-16
GCST90179150	6	46393672	RCAN2	rs6458501	intron_variant	2.00E-15
GCST90255621	6	46430267	RCAN2	rs1554790	intron_variant	7.00E-13
GCST90018947	6	153043508	RGS17	rs7755574	intron_variant	2.00E-17
GCST90255621	6	153060487	RGS17	rs2185027	intron_variant	3.00E-16
GCST90179150	6	153060487	RGS17	rs2185027	intron_variant	1.00E-15
GCST009004	6	153106130	RGS17	rs9479509	intron_variant	3.00E-13
GCST007039	6	153054772	RGS17	rs7749708	intron_variant	3.00E-12
GCST004904	6	153060487	RGS17	rs2185027	intron_variant	3.00E-11
GCST009871	6	153106130	RGS17	rs9479509	intron_variant	8.00E-11
GCST90179150	6	50877777	RPS17P5 - FTH1P5	rs2207139	intergenic_variant	9.00E-83
GCST009003	6	50898107	RPS17P5 - FTH1P5	rs943005	non_coding_transcript_exon_	1.00E-81
GCST002783	6	50877777	RPS17P5 - FTH1P5	rs2207139	intergenic_variant	8.00E-31
GCST002783	6	50877777	RPS17P5 - FTH1P5	rs2207139	intergenic_variant	4.00E-29
GCST004497	6	50877777	RPS17P5 - FTH1P5	rs2207139	intergenic_variant	4.00E-21
GCST002783	6	50877777	RPS17P5 - FTH1P5	rs2207139	intergenic_variant	2.00E-19
GCST004495	6	50877777	RPS17P5 - FTH1P5	rs2207139	intergenic_variant	9.00E-19
GCST002783	6	50877777	RPS17P5 - FTH1P5	rs2207139	intergenic_variant	2.00E-16
GCST004499	6	50877777	RPS17P5 - FTH1P5	rs2207139	intergenic_variant	2.00E-13
GCST004497	6	50877777	RPS17P5 - FTH1P5	rs2207139	intergenic_variant	2.00E-13
GCST006802	6	50877777	RPS17P5 - FTH1P5	rs2207139	intergenic_variant	2.00E-12
GCST004495	6	50877777	RPS17P5 - FTH1P5	rs2207139	intergenic_variant	4.00E-12
GCST003177	6	50877777	RPS17P5 - FTH1P5	rs2207139	intergenic_variant	7.00E-11
GCST90179150	6	35238776	SCUBE3	rs732594	intron_variant	2.00E-23
GCST009871	6	35238776	SCUBE3	rs732594	intron_variant	3.00E-23
GCST90255621	6	35201931	SCUBE3	rs2104332	regulatory_region_variant	4.00E-23
GCST009871	6	35194364	SCUBE3	rs11755266	intergenic_variant	3.00E-21
GCST002829	6	25801091	SLC17A1	rs1165209	intron_variant	4.00E-15
GCST002830	6	25801091	SLC17A1	rs1165209	intron_variant	3.00E-13
GCST002830	6	25801091	SLC17A1	rs1165209	intron_variant	1.00E-11
GCST011334	6	160351810	SLC22A3	rs539958	intron_variant	1.00E-14
GCST006368	6	160353454	SLC22A3	rs487152	intron_variant	2.00E-12
GCST004904	6	160353454	SLC22A3	rs487152	intron_variant	2.00E-12
GCST90255621	6	160451119	SLC22A3	rs1810126	3_prime_UTR_variant	2.00E-12
GCST90179150	6	160354546	SLC22A3	rs518295	intron_variant	8.00E-12

GCST011335	6	160351810	SLC22A3	rs539958	intron_variant	2.00E-11
GCST009004	6	160353454	SLC22A3	rs487152	intron_variant	3.00E-11
GCST90255621	6	33338458	SMIM40 - MYL12BP3	rs9277988	intergenic_variant	3.00E-23
GCST009871	6	33338458	SMIM40 - MYL12BP3	rs9277988	intergenic_variant	3.00E-13
GCST90271771	6	44957556	SUPT3H	rs12210292	intron_variant	5.00E-19
GCST90271767	6	44801188	SUPT3H	rs12200892	intron_variant	5.00E-14
GCST90271767	6	45314374	SUPT3H	rs12191751	intron_variant	3.00E-12
GCST90255621	6	40380914	TDRG1	rs2033529	non_coding_transcript_exon_	4.00E-41
GCST009004	6	40380914	TDRG1	rs2033529	non_coding_transcript_exon_	2.00E-29
GCST009871	6	40380156	TDRG1	rs2033531	non_coding_transcript_exon_	2.00E-18
GCST90255621	6	40365072	TDRG1	rs11756704	intron_variant	1.00E-16
GCST009871	6	40377803	TDRG1	rs12663230	intron_variant	2.00E-13
GCST009871	6	40376720	TDRG1	rs4714342	intron_variant	8.00E-13
GCST90179150	6	40377959	TDRG1	rs12664614	intron_variant	4.00E-12
GCST006368	6	40380914	TDRG1	rs2033529	non_coding_transcript_exon_	2.00E-11
GCST90255621	6	40035763	TDRG1	rs4714290	intergenic_variant	2.00E-11
GCST90267268	6	40380156	TDRG1	rs2033531	non_coding_transcript_exon_	6.00E-11
GCST90255621	6	50835337	TFAP2B	rs987237	intron_variant	3.00E-109
GCST009004	6	50830813	TFAP2B	rs2206277	intron_variant	2.00E-83
GCST009001	6	50830813	TFAP2B	rs2206277	intron_variant	2.00E-83
GCST90255621	6	50853227	TFAP2B	rs2635727	intergenic_variant	8.00E-62
GCST007039	6	50849174	TFAP2B	rs72892910	regulatory_region_variant	3.00E-53
GCST009871	6	50849174	TFAP2B	rs72892910	regulatory_region_variant	2.00E-52
GCST90018947	6	50821065	TFAP2B	rs3798519	intron_variant	3.00E-52
GCST90179150	6	50845193	TFAP2B	rs2817419	3_prime_UTR_variant	4.00E-49
GCST009121	6	50835337	TFAP2B	rs987237	intron_variant	8.00E-43
GCST004904	6	50830813	TFAP2B	rs2206277	intron_variant	1.00E-38
GCST009871	6	50853227	TFAP2B	rs2635727	intergenic_variant	1.00E-35
GCST005951	6	50830813	TFAP2B	rs2206277	intron_variant	7.00E-32
GCST90103755	6	50845193	TFAP2B	rs2817419	3_prime_UTR_variant	6.00E-26
GCST000830	6	50835337	TFAP2B	rs987237	intron_variant	3.00E-20
GCST004904	6	50818295	TFAP2B	rs2206271	regulatory_region_variant	3.00E-18
GCST90018727	6	50818295	TFAP2B	rs2206271	regulatory_region_variant	4.00E-18
GCST009107	6	50835337	TFAP2B	rs987237	intron_variant	2.00E-16
GCST004557	6	50835337	TFAP2B	rs987237	intron_variant	2.00E-16
GCST004557	6	50835337	TFAP2B	rs987237	intron_variant	3.00E-15
GCST004558	6	50835337	TFAP2B	rs987237	intron_variant	4.00E-15
GCST009871	6	50715296	TFAP2B	rs78648104	missense_variant	1.00E-14
GCST004559	6	50835337	TFAP2B	rs987237	intron_variant	7.00E-14
GCST90131907	6	50818295	TFAP2B	rs2206271	regulatory_region_variant	9.00E-14
GCST90239604	6	50817167	TFAP2B	rs2744475	regulatory_region_variant	2.00E-13
GCST004559	6	50835337	TFAP2B	rs987237	intron_variant	6.00E-13
GCST003177	6	50835337	TFAP2B	rs987237	intron_variant	2.00E-12
GCST90002409	6	50823927	TFAP2B	rs2076308	intron_variant	2.00E-12
GCST004558	6	50835337	TFAP2B	rs987237	intron_variant	7.00E-12
GCST009764	6	50817167	TFAP2B	rs2744475	regulatory_region_variant	7.00E-12
GCST001955	6	50835337	TFAP2B	rs987237	intron_variant	2.00E-11
GCST009871	6	50795922	TFAP2B	rs141724389	intergenic_variant	2.00E-11
GCST004557	6	50835337	TFAP2B	rs987237	intron_variant	3.00E-11
GCST004559	6	50835337	TFAP2B	rs987237	intron_variant	3.00E-11
GCST009555	6	50845193	TFAP2B	rs2817419	3_prime_UTR_variant	4.00E-11

GCST90179150	6	82723511	TPBG - UBE3D	rs9294260	intergenic_variant	5.00E-18
GCST90255621	6	82723511	TPBG - UBE3D	rs9294260	intergenic_variant	8.00E-16
GCST007039	6	82723511	TPBG - UBE3D	rs9294260	intergenic_variant	4.00E-14
GCST90104632	6	82813965	TPBG - UBE3D	rs209421	intergenic_variant	5.00E-13
GCST009004	6	82738096	TPBG - UBE3D	rs7769594	regulatory_region_variant	2.00E-12
GCST90255621	6	82752604	TPBG - UBE3D	rs951537	regulatory_region_variant	5.00E-11
GCST90255621	6	41166068	TREM2	rs7748777	intron_variant	2.00E-16
GCST90179150	6	41166068	TREM2	rs7748777	intron_variant	4.00E-11
GCST90179150	6	35508665	TULP1	rs2064319	intron_variant	2.00E-12
GCST009871	6	35509255	TULP1	rs2064317	missense_variant	4.00E-12
GCST90255621	6	43790159	VEGFA	rs998584	regulatory_region_variant	5.00E-23
GCST90179150	6	43790159	VEGFA	rs998584	regulatory_region_variant	2.00E-17
GCST90271770	6	43790159	VEGFA	rs998584	regulatory_region_variant	2.00E-17
GCST011334	6	43790159	VEGFA	rs998584	regulatory_region_variant	3.00E-16
GCST011336	6	43790159	VEGFA	rs998584	regulatory_region_variant	6.00E-15
GCST90255621	7	70341037	ATXN7L3	rs6943555	intron_variant	2.00E-15
GCST90018947	7	70133342	AUTS2	rs1035010	intron_variant	3.00E-15
GCST90018727	7	69980355	AUTS2	rs11981973	intron_variant	6.00E-14
GCST90255621	7	70602329	AUTS2	rs38314	intron_variant	5.00E-13
GCST009004	7	70133342	AUTS2	rs1035010	intron_variant	2.00E-12
GCST004904	7	70303840	AUTS2	rs1949804	intron_variant	4.00E-12
GCST004904	7	69941675	AUTS2	rs6947395	intron_variant	5.00E-12
GCST009003	7	70580955	AUTS2	rs10237317	intron_variant	1.00E-11
GCST90179150	7	70182239	AUTS2	rs10268924	intron_variant	1.00E-11
GCST009871	7	70324581	AUTS2	rs6957740	intron_variant	2.00E-11
GCST90179150	7	70575572	AUTS2	rs4718966	intron_variant	2.00E-11
GCST009871	7	70641324	AUTS2	rs2866720	intron_variant	7.00E-11
GCST004046	7	73462836	BARX1	rs2074755	non_coding_transcript_exon_	3.00E-40
GCST004045	7	73462836	BAZ1B	rs2074755	non_coding_transcript_exon_	1.00E-17
GCST90255621	7	122320354	CADPS2	rs777907005	intron_variant	6.00E-17
GCST90271767	7	122320353	CADPS2	rs1348442	splice_polypyrimidine_tract_	2.00E-13
GCST009004	7	122324295	CADPS2	rs1899689	intron_variant	4.00E-13
GCST90179150	7	122320353	CADPS2	rs1348442	splice_polypyrimidine_tract_	3.00E-12
GCST90179150	7	93505743	CALCR	rs6962185	intron_variant	2.00E-15
GCST90255621	7	93532688	CALCR	rs12668072	intron_variant	4.00E-15
GCST009004	7	93459931	CALCR	rs7777084	intron_variant	2.00E-14
GCST004495	7	93568420	CALCR	rs9641123	intron_variant	1.00E-11
GCST004497	7	93568420	CALCR	rs9641123	intron_variant	2.00E-11
GCST007039	7	71972119	CALN1	rs12538435	intron_variant	4.00E-14
GCST90255621	7	72138707	CALN1	rs11772246	intron_variant	6.00E-14
GCST90179150	7	71974822	CALN1	rs12530737	intron_variant	6.00E-13
GCST009004	7	71964323	CALN1	rs10950289	intron_variant	1.00E-11
GCST009003	7	71964323	CALN1	rs10950289	intron_variant	1.00E-11
GCST009871	7	71963975	CALN1	rs3973896	intron_variant	3.00E-11
GCST90255621	7	77189360	CCDC146	rs11505821	intron_variant	9.00E-26
GCST009871	7	77184736	CCDC146	rs73371569	intron_variant	6.00E-15
GCST90255621	7	50496506	DDC	rs3807566	intron_variant	4.00E-16
GCST90179150	7	50546475	DDC	rs10499694	intron_variant	8.00E-16
GCST009004	7	50546475	DDC	rs10499694	intron_variant	1.00E-15
GCST009003	7	50546475	DDC	rs10499694	intron_variant	1.00E-15
GCST90255621	7	49576607	DDX43P2 - VWC2	rs10269783	intron_variant	3.00E-16

GCST009004	7	49576607	DDX43P2 - VWC2	rs10269783	intron_variant	2.00E-14
GCST009001	7	49576607	DDX43P2 - VWC2	rs10269783	intron_variant	2.00E-14
GCST90179150	7	49576607	DDX43P2 - VWC2	rs10269783	intron_variant	8.00E-13
GCST007039	7	49576607	DDX43P2 - VWC2	rs10269783	intron_variant	4.00E-11
GCST90255621	7	137739763	DGKI	rs3800649	intron_variant	9.00E-14
GCST009004	7	137751179	DGKI	rs7802342	intron_variant	6.00E-11
GCST90255621	7	76978826	DTX2P1	rs2245368	non_coding_transcript_exon_	1.00E-37
GCST007039	7	77010522	DTX2P1	rs6951489	intron_variant	4.00E-25
GCST90179150	7	77005146	DTX2P1	rs17149254	intron_variant	2.00E-24
GCST90018947	7	77008941	DTX2P1	rs7792906	intron_variant	2.00E-23
GCST009004	7	77002781	DTX2P1	rs6954694	intron_variant	1.00E-20
GCST009001	7	77002781	DTX2P1	rs6954694	intron_variant	1.00E-20
GCST005951	7	77005146	DTX2P1	rs17149254	intron_variant	3.00E-11
GCST90255621	7	114710047	FOXP2 - MDFIC	rs2045293	regulatory_region_variant	2.00E-18
GCST90179150	7	114711212	FOXP2 - MDFIC	rs10500039	regulatory_region_variant	3.00E-16
GCST007039	7	114712560	FOXP2 - MDFIC	rs1840660	intergenic_variant	3.00E-16
GCST90255621	7	114795292	FOXP2 - MDFIC	rs1448349	intergenic_variant	9.00E-13
GCST001527	7	44196069	GCK	rs4607517	intron_variant	8.00E-56
GCST90104629	7	44184259	GCK	rs78412508	intron_variant	4.00E-15
GCST90255621	7	93612328	GNGT1	rs7780752	intron_variant	2.00E-19
GCST009003	7	93607198	GNGT1	rs2528531	intron_variant	9.00E-14
GCST90179150	7	77426568	GSAP - GCNT1P5	rs740157	regulatory_region_variant	3.00E-13
GCST009004	7	77426568	GSAP - GCNT1P5	rs740157	regulatory_region_variant	5.00E-13
GCST90255621	7	77418465	GSAP - GCNT1P5	rs7808180	intron_variant	2.00E-12
GCST90255621	7	74687575	GTF2I	rs6964833	intron_variant	3.00E-18
GCST90179150	7	74687575	GTF2I	rs6964833	intron_variant	2.00E-17
GCST90255621	7	74683288	GTF2I	rs56383938	intron_variant	1.00E-15
GCST009871	7	74685413	GTF2I	rs138745417	intron_variant	5.00E-14
GCST90179150	7	74683288	GTF2I	rs56383938	intron_variant	3.00E-13
GCST009871	7	74708526	GTF2I	rs7795282	intron_variant	5.00E-12
GCST90255621	7	75533848	HIP1	rs1167827	3_prime_UTR_variant	1.00E-53
GCST005951	7	75533848	HIP1	rs1167827	3_prime_UTR_variant	1.00E-12
GCST006368	7	75533848	HIP1	rs1167827	3_prime_UTR_variant	3.00E-12
GCST90255621	7	28149792	JAZF1	rs1635852	intron_variant	1.00E-18
GCST90271771	7	28149792	JAZF1	rs1635852	intron_variant	4.00E-17
GCST90179150	7	28156794	JAZF1	rs849135	intron_variant	8.00E-12
GCST011329	7	28140937	JAZF1	rs864745	intron_variant	9.00E-12
GCST90255621	7	150948446	KCNH2	rs1805123	missense_variant	4.00E-21
GCST008129	7	150948446	KCNH2	rs1805123	missense_variant	5.00E-15
GCST009004	7	150941396	KCNH2	rs2907948	intergenic_variant	2.00E-14
GCST009003	7	150925074	KCNH2	rs2968864	intergenic_variant	4.00E-14
GCST90179150	7	150941396	KCNH2	rs2907948	intergenic_variant	2.00E-13
GCST009871	7	150948446	KCNH2	rs1805123	missense_variant	3.00E-13
GCST007039	7	150948446	KCNH2	rs1805123	missense_variant	3.00E-13
GCST90255621	7	130732987	KLF14	rs3909553	3_prime_UTR_variant	1.00E-11
GCST90255621	7	130782095	KLF14	rs972283	intergenic_variant	3.00E-11
GCST90018727	7	127478352	LINC03012	rs72607741	intron_variant	5.00E-12
GCST90018947	7	127478352	LINC03012	rs72607741	intron_variant	5.00E-12
GCST90255621	7	2064033	MAD1L1	rs6461115	intron_variant	1.00E-16
GCST90018947	7	2040109	MAD1L1	rs2056477	intron_variant	2.00E-14
GCST007039	7	1833285	MAD1L1	rs4721089	intron_variant	5.00E-14

GCST009871	7	1833285	MAD1L1	rs4721089	intron_variant	7.00E-14
GCST90179150	7	1833285	MAD1L1	rs4721089	intron_variant	5.00E-13
GCST009004	7	1833285	MAD1L1	rs4721089	intron_variant	6.00E-13
GCST90255621	7	78200451	MAGI2	rs1852006	intron_variant	7.00E-26
GCST009004	7	78200451	MAGI2	rs1852006	intron_variant	7.00E-18
GCST009001	7	78200451	MAGI2	rs1852006	intron_variant	7.00E-18
GCST90179150	7	78200451	MAGI2	rs1852006	intron_variant	8.00E-17
GCST009871	7	78200451	MAGI2	rs1852006	intron_variant	2.00E-13
GCST007039	7	78200451	MAGI2	rs1852006	intron_variant	3.00E-13
GCST90255621	7	78173777	MAGI2	rs2691528	intron_variant	2.00E-11
GCST90179150	7	78492141	MAGI2	rs7805441	intron_variant	3.00E-11
GCST90179150	7	78173777	MAGI2	rs2691528	intron_variant	6.00E-11
GCST004046	7	73637727	MLXIPL	rs799165	intergenic_variant	8.00E-19
GCST004045	7	73637727	MLXIPL	rs799165	intergenic_variant	9.00E-15
GCST90179150	7	73612048	MLXIPL	rs17145750	intron_variant	1.00E-12
GCST009004	7	24314681	NPY	rs4307239	intergenic_variant	1.00E-11
GCST009003	7	24314681	NPY	rs4307239	intergenic_variant	1.00E-11
GCST90255621	7	24314681	NPY	rs4307239	intergenic_variant	1.00E-11
GCST90179150	7	24314681	NPY	rs4307239	intergenic_variant	9.00E-11
GCST90255621	7	44745098	OGDH	rs799449	intergenic_variant	8.00E-16
GCST007039	7	44745098	OGDH	rs799449	intergenic_variant	2.00E-14
GCST90267268	7	44728822	OGDH	rs799442	intergenic_variant	8.00E-11
GCST90179150	7	32329536	PDE1C	rs215634	intron_variant	8.00E-19
GCST90255621	7	32298725	PDE1C	rs215607	missense_variant	1.00E-17
GCST009004	7	32307723	PDE1C	rs215614	intron_variant	3.00E-16
GCST007039	7	32329536	PDE1C	rs215634	intron_variant	3.00E-16
GCST009001	7	32298725	PDE1C	rs215607	missense_variant	6.00E-15
GCST009871	7	32307723	PDE1C	rs215614	intron_variant	8.00E-14
GCST90018947	7	32339367	PDE1C	rs215669	intron_variant	6.00E-13
GCST009004	7	75520983	PMS2P3	rs1167821	intron_variant	9.00E-26
GCST009001	7	75520983	PMS2P3	rs1167821	intron_variant	9.00E-26
GCST90267268	7	75520983	PMS2P3	rs1167821	intron_variant	4.00E-15
GCST90179150	7	75471799	POM121C	rs17207196	intron_variant	2.00E-37
GCST007039	7	75452145	POM121C	rs58862095	intron_variant	5.00E-31
GCST90018947	7	75420803	POM121C	rs236660	non_coding_transcript_exon_	1.00E-26
GCST009003	7	75432493	POM121C	rs6944634	intron_variant	8.00E-25
GCST009871	7	75429830	POM121C	rs6979377	intron_variant	3.00E-20
GCST90255621	7	75450397	POM121C	rs58030979	intron_variant	9.00E-18
GCST90179150	7	75420076	POM121C	rs60932781	non_coding_transcript_exon_	9.00E-15
GCST90255621	7	39409337	POU6F2	rs2237403	intron_variant	4.00E-13
GCST009004	7	39409337	POU6F2	rs2237403	intron_variant	1.00E-12
GCST90179150	7	39409337	POU6F2	rs2237403	intron_variant	8.00E-12
GCST009871	7	39409337	POU6F2	rs2237403	intron_variant	5.00E-11
GCST90255621	7	39018977	POU6F2	rs702820	intron_variant	5.00E-11
GCST90091177	7	141851391	PRSS37 - OR9A3P	rs77586724	intergenic_variant	4.00E-22
GCST009057	7	141849517	PRSS37 - OR9A3P	rs7798566	intergenic_variant	3.00E-15
GCST90255621	7	103778399	RELN	rs2299383	intron_variant	2.00E-27
GCST90179150	7	103778399	RELN	rs2299383	intron_variant	7.00E-24
GCST009004	7	103778399	RELN	rs2299383	intron_variant	7.00E-23
GCST009003	7	103778399	RELN	rs2299383	intron_variant	7.00E-23
GCST009001	7	103777110	RELN	rs11496125	intron_variant	9.00E-23

GCST009871	7	103776094	RELN	rs12375196	intron_variant	1.00E-21
GCST007039	7	103776094	RELN	rs12375196	intron_variant	2.00E-20
GCST90018947	7	103809108	RELN	rs39330	intron_variant	1.00E-15
GCST90267268	7	103775848	RELN	rs17155888	intron_variant	4.00E-12
GCST90255621	7	113388579	SMIM30 - PPP1R3A	rs2396625	intergenic_variant	5.00E-25
GCST009004	7	113388579	SMIM30 - PPP1R3A	rs2396625	intergenic_variant	3.00E-24
GCST90179150	7	113347595	SMIM30 - PPP1R3A	rs4476935	intergenic_variant	2.00E-22
GCST009003	7	113395778	SMIM30 - PPP1R3A	rs1524445	regulatory_region_variant	3.00E-22
GCST007039	7	113388579	SMIM30 - PPP1R3A	rs2396625	intergenic_variant	4.00E-21
GCST009871	7	113346729	SMIM30 - PPP1R3A	rs10953733	intergenic_variant	9.00E-20
GCST90255621	7	113753935	SMIM30 - PPP1R3A	rs13222241	intergenic_variant	3.00E-19
GCST009004	7	113852779	SMIM30 - PPP1R3A	rs12705916	intergenic_variant	1.00E-18
GCST009003	7	113852779	SMIM30 - PPP1R3A	rs12705916	intergenic_variant	1.00E-18
GCST007039	7	113843514	SMIM30 - PPP1R3A	rs6944092	intergenic_variant	2.00E-18
GCST90179150	7	113722744	SMIM30 - PPP1R3A	rs13245051	intergenic_variant	5.00E-18
GCST009871	7	113843514	SMIM30 - PPP1R3A	rs6944092	intergenic_variant	6.00E-18
GCST009001	7	113393371	SMIM30 - PPP1R3A	rs1701829	regulatory_region_variant	2.00E-17
GCST90267268	7	113332428	SMIM30 - PPP1R3A	rs7788008	intergenic_variant	1.00E-13
GCST90018947	7	113338667	SMIM30 - PPP1R3A	rs397703606	intergenic_variant	5.00E-12
GCST009871	7	77053418	SPDYE18	rs9649187	intron_variant	2.00E-21
GCST90179150	7	77083484	SPDYE18	rs17722341	intron_variant	2.00E-12
GCST90255621	7	139189670	TTC26	rs13233916	3_prime_UTR_variant	1.00E-20
GCST009004	7	139132447	TTC26	rs11525873	intergenic_variant	3.00E-13
GCST007039	7	139132447	TTC26	rs11525873	intergenic_variant	3.00E-13
GCST009871	7	139132447	TTC26	rs11525873	intergenic_variant	4.00E-13
GCST90179150	7	139132447	TTC26	rs11525873	intergenic_variant	5.00E-13
GCST90018947	7	139132447	TTC26	rs11525873	intergenic_variant	8.00E-11
GCST007039	7	99510104	ZKSCAN5	rs3901286	intron_variant	2.00E-19
GCST90179150	7	99510104	ZKSCAN5	rs3901286	intron_variant	3.00E-16
GCST009871	7	99505288	ZKSCAN5	rs11973801	5_prime_UTR_variant	2.00E-14
GCST009004	7	99521178	ZKSCAN5	rs11761528	intron_variant	6.00E-14
GCST90018947	7	44764626	ZMIZ2	rs2289379	intron_variant	4.00E-15
GCST90179150	7	44751389	ZMIZ2	rs799451	intron_variant	4.00E-14
GCST009871	7	44749058	ZMIZ2	rs6962280	intron_variant	1.00E-13
GCST009004	7	44749058	ZMIZ2	rs6962280	intron_variant	3.00E-13
GCST009001	7	44749058	ZMIZ2	rs6962280	intron_variant	3.00E-13
GCST90018947	8	141606996	C8orf90	rs11782074	intron_variant	4.00E-15
GCST009004	8	141609134	C8orf90	rs1106761	intron_variant	2.00E-11
GCST90179150	8	141606996	C8orf90	rs11782074	intron_variant	6.00E-11
GCST007039	8	141606996	C8orf90	rs11782074	intron_variant	6.00E-11
GCST90255621	8	61205414	CLVS1	rs6471941	intron_variant	1.00E-15
GCST006368	8	61166345	CLVS1	rs6471932	intron_variant	1.00E-14
GCST90179150	8	61141904	CLVS1	rs12681792	intron_variant	2.00E-12
GCST009004	8	61141904	CLVS1	rs12681792	intron_variant	3.00E-12
GCST009003	8	61141904	CLVS1	rs12681792	intron_variant	3.00E-12
GCST009004	8	4431055	CSMD1	rs1658820	intron_variant	4.00E-11
GCST007039	8	4431055	CSMD1	rs1658820	intron_variant	6.00E-11
GCST90179150	8	4431055	CSMD1	rs1658820	intron_variant	7.00E-11
GCST90255621	8	28164252	ELP3	rs1982441	intron_variant	3.00E-14
GCST90255621	8	28260613	ELP3	rs2100814	intron_variant	6.00E-14
GCST009004	8	28164252	ELP3	rs1982441	intron_variant	2.00E-11

GCST90018947	8	28285742	ELP3	rs7009996	intron_variant	2.00E-11
GCST90179150	8	28164252	ELP3	rs1982441	intron_variant	6.00E-11
GCST007039	8	117872140	EXT1	rs72673947	intron_variant	6.00E-14
GCST009004	8	117872140	EXT1	rs72673947	intron_variant	4.00E-13
GCST009001	8	117872140	EXT1	rs72673947	intron_variant	4.00E-13
GCST009871	8	117872140	EXT1	rs72673947	intron_variant	4.00E-13
GCST90179150	8	117872140	EXT1	rs72673947	intron_variant	4.00E-12
GCST90255621	8	117850822	EXT1	rs11781699	intron_variant	8.00E-11
GCST009004	8	75894349	HNF4G	rs17405819	intergenic_variant	6.00E-33
GCST009003	8	75894349	HNF4G	rs17405819	intergenic_variant	6.00E-33
GCST90179150	8	75894349	HNF4G	rs17405819	intergenic_variant	1.00E-30
GCST009001	8	75723956	HNF4G	rs2588785	intergenic_variant	2.00E-28
GCST009871	8	75874706	HNF4G	rs12678759	intergenic_variant	8.00E-26
GCST90018947	8	75809375	HNF4G	rs2977321	intergenic_variant	2.00E-25
GCST90255621	8	75738099	HNF4G	rs2060604	intergenic_variant	1.00E-24
GCST90179150	8	76250459	HNF4G	rs10112085	intron_variant	4.00E-21
GCST006368	8	75894349	HNF4G	rs17405819	intergenic_variant	9.00E-20
GCST004904	8	75894349	HNF4G	rs17405819	intergenic_variant	8.00E-19
GCST006368	8	75703428	HNF4G	rs4735692	intergenic_variant	7.00E-17
GCST005951	8	75894349	HNF4G	rs17405819	intergenic_variant	6.00E-16
GCST90255621	8	75856503	HNF4G	rs1462441	intergenic_variant	2.00E-15
GCST009871	8	75498433	HNF4G	rs1096389	intron_variant	5.00E-15
GCST002783	8	75894349	HNF4G	rs17405819	intergenic_variant	1.00E-11
GCST002783	8	75894349	HNF4G	rs17405819	intergenic_variant	2.00E-11
GCST007039	8	75613351	HNF4G	rs2941428	intergenic_variant	3.00E-11
GCST009871	8	75784741	HNF4G	rs2977329	intergenic_variant	7.00E-11
GCST90255621	8	72526835	KCNB2	rs1431659	intergenic_variant	1.00E-25
GCST007039	8	72523729	KCNB2	rs1808629	intergenic_variant	3.00E-24
GCST009871	8	72530963	KCNB2	rs12679106	intergenic_variant	7.00E-24
GCST009004	8	72526835	KCNB2	rs1431659	intergenic_variant	2.00E-23
GCST009001	8	72526835	KCNB2	rs1431659	intergenic_variant	2.00E-23
GCST009003	8	72537705	KCNB2	rs7845090	5_prime_UTR_variant	5.00E-23
GCST90179150	8	72523729	KCNB2	rs1808629	intergenic_variant	7.00E-23
GCST90018947	8	72528136	KCNB2	rs35957544	intergenic_variant	3.00E-16
GCST90255621	8	94570378	KIAA1429	rs12680842	non_coding_transcript_exon_	4.00E-20
GCST90018947	8	94556299	KIAA1429	rs11986009	intron_variant	9.00E-18
GCST90179150	8	94570378	KIAA1429	rs12680842	non_coding_transcript_exon_	2.00E-16
GCST009004	8	94570378	KIAA1429	rs12680842	non_coding_transcript_exon_	3.00E-16
GCST009001	8	94559374	KIAA1429	rs10104041	intron_variant	1.00E-11
GCST004904	8	94570378	KIAA1429	rs12680842	non_coding_transcript_exon_	2.00E-11
GCST009871	8	94611879	KIAA1429	rs12678226	intergenic_variant	2.00E-11
GCST007039	8	94570378	KIAA1429	rs12680842	non_coding_transcript_exon_	3.00E-11
GCST004046	8	20008763	LPL	rs765547	intergenic_variant	3.00E-51
GCST004045	8	20008763	LPL	rs765547	intergenic_variant	2.00E-44
GCST90179150	8	20811113	LZTS1	rs2616192	intergenic_variant	4.00E-12
GCST90255621	8	20811113	LZTS1	rs2616192	intergenic_variant	4.00E-12
GCST007039	8	20774511	LZTS1	rs2616143	intergenic_variant	7.00E-12
GCST009871	8	20782300	LZTS1	rs4366093	intergenic_variant	2.00E-11
GCST009004	8	20777377	LZTS1	rs10101364	intergenic_variant	6.00E-11
GCST90255621	8	9938811	MSRA	rs615632	intergenic_variant	4.00E-25
GCST90179150	8	10036930	MSRA	rs77092199	intron_variant	1.00E-14

GCST90255621	8	10036930	MSRA	rs77092199	intron_variant	2.00E-14
GCST90179150	8	10125701	MSRA	rs17151145	intron_variant	3.00E-13
GCST90255621	8	10217128	MSRA	rs4275226	intron_variant	1.00E-12
GCST90255621	8	10330369	MSRA	rs62490332	intron_variant	1.00E-12
GCST90179150	8	10207280	MSRA	rs7844551	intron_variant	2.00E-12
GCST90179150	8	10337135	MSRA	rs55878365	non_coding_transcript_exon_	3.00E-12
GCST009121	8	9888288	MSRA	rs17150703	intergenic_variant	2.00E-11
GCST90255621	8	32542597	NRG1	rs7826312	intron_variant	3.00E-15
GCST90255621	8	32554786	NRG1	rs2466103	intron_variant	2.00E-13
GCST009004	8	32542597	NRG1	rs7826312	intron_variant	3.00E-11
GCST90179150	8	32542597	NRG1	rs7826312	intron_variant	4.00E-11
GCST90179150	8	32554786	NRG1	rs2466103	intron_variant	4.00E-11
GCST90179150	8	9221187	PPP1R3B	rs2929459	intron_variant	4.00E-15
GCST90271769	8	9227785	PPP1R3B	rs2929451	intron_variant	6.00E-14
GCST90179150	8	9363817	PPP1R3B	rs13279515	intron_variant	6.00E-13
GCST011334	8	9338128	PPP1R3B	rs17149279	intron_variant	3.00E-12
GCST90255621	8	9348559	PPP1R3B	rs10102352	intron_variant	4.00E-12
GCST011336	8	9338128	PPP1R3B	rs17149279	intron_variant	1.00E-11
GCST90255621	8	9277687	PPP1R3B	rs330029	intron_variant	9.00E-11
GCST007039	8	27403621	PTK2B	rs117176448	intron_variant	6.00E-12
GCST009871	8	27403621	PTK2B	rs117176448	intron_variant	8.00E-12
GCST009004	8	27403621	PTK2B	rs117176448	intron_variant	7.00E-11
GCST90255621	8	30998948	PURG	rs1362910	intron_variant	2.00E-16
GCST90018947	8	31006422	PURG	rs10954772	intron_variant	2.00E-15
GCST90179150	8	31008217	PURG	rs1421334	intron_variant	2.00E-14
GCST009004	8	31006422	PURG	rs10954772	intron_variant	5.00E-14
GCST009871	8	30996517	PURG	rs2725371	3_prime_UTR_variant	2.00E-13
GCST007039	8	31006422	PURG	rs10954772	intron_variant	2.00E-13
GCST009001	8	31000538	PURG	rs1125841	intron_variant	1.00E-12
GCST90255621	8	84177202	RALYL	rs2196618	intergenic_variant	3.00E-16
GCST90255621	8	84784102	RALYL	rs2634047	intron_variant	2.00E-14
GCST009004	8	84177202	RALYL	rs2196618	intergenic_variant	1.00E-12
GCST90179150	8	84177202	RALYL	rs2196618	intergenic_variant	5.00E-12
GCST009871	8	84611521	RALYL	rs28465008	intron_variant	4.00E-11
GCST90179150	8	84614369	RALYL	rs7842614	intron_variant	4.00E-11
GCST007039	8	84784102	RALYL	rs2634047	intron_variant	4.00E-11
GCST90255621	8	86507313	RMDN1	rs7006629	intron_variant	1.00E-11
GCST90018947	8	86490903	RMDN1	rs7817738	intron_variant	2.00E-11
GCST90018947	8	63654964	RN7SKP135	rs10091265	intron_variant	2.00E-15
GCST004904	8	63640221	RN7SKP135	rs77636220	intergenic_variant	3.00E-11
GCST90018727	8	63653399	RN7SKP135	rs2060859	intron_variant	5.00E-11
GCST90255621	8	76322861	RNU2-54P - LINC01109	rs10808810	intergenic_variant	3.00E-36
GCST009871	8	76326157	RNU2-54P - LINC01109	rs10102172	intergenic_variant	6.00E-28
GCST007039	8	76326157	RNU2-54P - LINC01109	rs10102172	intergenic_variant	2.00E-27
GCST90255621	8	137202985	RNU6-144P - ZYXP1	rs16906845	intergenic_variant	8.00E-13
GCST009004	8	137202985	RNU6-144P - ZYXP1	rs16906845	intergenic_variant	1.00E-11
GCST90179150	8	137202985	RNU6-144P - ZYXP1	rs16906845	intergenic_variant	2.00E-11
GCST90255621	8	34646258	RPL10AP3 - LINC01288	rs7844647	regulatory_region_variant	2.00E-12
GCST90018947	8	34323941	RPL10AP3 - LINC01288	rs1458913	intron_variant	6.00E-11
GCST90255621	8	14238391	SGCZ	rs13263601	intron_variant	2.00E-18
GCST90179150	8	14210380	SGCZ	rs7008726	intron_variant	4.00E-16

GCST009004	8	14233516	SGCZ	rs4123853	intron_variant	1.00E-15
GCST009001	8	14233516	SGCZ	rs4123853	intron_variant	1.00E-15
GCST007039	8	14238391	SGCZ	rs13263601	intron_variant	2.00E-14
GCST009871	8	14236584	SGCZ	rs13250197	intron_variant	8.00E-13
GCST90018947	8	14238254	SGCZ	rs6530737	intron_variant	2.00E-12
GCST90255621	8	14476215	SGCZ	rs2126320	intron_variant	4.00E-12
GCST009871	8	14416489	SGCZ	rs13264759	intron_variant	5.00E-12
GCST90255621	8	23532058	SLC25A37	rs11781222	intron_variant	3.00E-15
GCST009004	8	23532058	SLC25A37	rs11781222	intron_variant	3.00E-12
GCST90179150	8	23532058	SLC25A37	rs11781222	intron_variant	1.00E-11
GCST004046	8	125466108	TRIB1	rs2980853	regulatory_region_variant	2.00E-22
GCST004045	8	125466108	TRIB1	rs2980853	regulatory_region_variant	3.00E-18
GCST90179150	8	125467120	TRIB1	rs6982502	intron_variant	3.00E-16
GCST90255621	8	125469835	TRIB1	rs2954021	intron_variant	1.00E-14
GCST90255621	8	115738322	TRPS1	rs2694047	intron_variant	2.00E-22
GCST009004	8	115658120	TRPS1	rs3808477	intron_variant	9.00E-22
GCST009871	8	115659621	TRPS1	rs4876611	intron_variant	1.00E-21
GCST007039	8	115738322	TRPS1	rs2694047	intron_variant	1.00E-21
GCST009003	8	115649811	TRPS1	rs2721965	intron_variant	4.00E-21
GCST90179150	8	115659621	TRPS1	rs4876611	intron_variant	7.00E-21
GCST009001	8	115706393	TRPS1	rs2960151	intron_variant	2.00E-20
GCST90018947	8	115658120	TRPS1	rs3808477	intron_variant	3.00E-16
GCST009871	8	115509798	TRPS1	rs2293888	intron_variant	3.00E-15
GCST90255621	8	115586972	TRPS1	rs2293889	intron_variant	1.00E-13
GCST90179150	8	115547208	TRPS1	rs3808434	intron_variant	1.00E-12
GCST009871	8	115417770	TRPS1	rs808994	intron_variant	4.00E-11
GCST90255621	8	115892450	TRPS1	rs2205258	intergenic_variant	5.00E-11
GCST90255621	8	142266561	TSNARE1	rs4601464	intron_variant	1.00E-13
GCST009004	8	142302333	TSNARE1	rs10099330	intron_variant	3.00E-12
GCST90179150	8	142302333	TSNARE1	rs10099330	intron_variant	1.00E-11
GCST007039	8	15708655	TUSC3	rs12679528	intron_variant	4.00E-11
GCST90179150	8	15707748	TUSC3	rs13265882	intron_variant	5.00E-11
GCST90255621	8	15707748	TUSC3	rs13265882	intron_variant	5.00E-11
GCST90271771	8	10930102	XKR6	rs4240673	intron_variant	8.00E-32
GCST90255621	8	11187652	XKR6	rs7819412	intron_variant	1.00E-30
GCST90271767	8	10930102	XKR6	rs4240673	intron_variant	5.00E-29
GCST90179150	8	10930102	XKR6	rs4240673	intron_variant	1.00E-28
GCST90179150	8	100949682	YWHAZ	rs983583	intron_variant	1.00E-12
GCST009004	8	100946205	YWHAZ	rs3134358	intron_variant	4.00E-12
GCST90255621	8	100935225	YWHAZ	rs3134353	intron_variant	2.00E-11
GCST90179150	9	11831420	AKAP6	rs17820822	intron_variant	6.00E-14
GCST90255621	9	11831420	AKAP8P1 - JKAMPP1	rs17820822	intron_variant	1.00E-13
GCST007039	9	11813799	AKAP8P1 - JKAMPP1	rs12375985	intron_variant	2.00E-13
GCST009004	9	11619764	AKAP8P1 - JKAMPP1	rs12336441	intron_variant	5.00E-13
GCST009003	9	11425445	AKAP8P1 - JKAMPP1	rs2105054	intron_variant	3.00E-12
GCST009871	9	11618414	AKAP8P1 - JKAMPP1	rs34640360	regulatory_region_variant	8.00E-12
GCST009871	9	11847175	AKAP8P1 - JKAMPP1	rs1486826	intron_variant	1.00E-11
GCST90255621	9	117637809	ASS1P14 - SYT10	rs7032255	intergenic_variant	9.00E-24
GCST90179150	9	117616205	ASTN2	rs1928295	intergenic_variant	1.00E-16
GCST009004	9	117616205	ASTN2	rs1928295	intergenic_variant	2.00E-16
GCST009003	9	117616205	ASTN2	rs1928295	intergenic_variant	2.00E-16

GCST004904	9	117616205	ASTN2	rs1928295	intergenic_variant	2.00E-13
GCST005951	9	117616205	ASTN2	rs1928295	intergenic_variant	2.00E-12
GCST90018947	9	117614900	ASTN2	rs7038943	intergenic_variant	7.00E-12
GCST009871	9	117614900	ASTN2	rs7038943	intergenic_variant	2.00E-11
GCST002783	9	117616205	ASTN2	rs1928295	intergenic_variant	3.00E-11
GCST007039	9	117614900	ASTN2	rs7038943	intergenic_variant	4.00E-11
GCST90255621	9	93820153	BANK1	rs117560393	intron_variant	2.00E-12
GCST009871	9	93820494	BARX1	rs117440602	intron_variant	1.00E-11
GCST90018947	9	16721923	BMAL1	rs146494938	intron_variant	1.00E-21
GCST90271770	9	16712249	BNC2	rs10962547	intron_variant	3.00E-20
GCST007039	9	16712249	BNC2	rs10962547	intron_variant	3.00E-19
GCST009871	9	16712249	BNC2	rs10962547	intron_variant	4.00E-19
GCST009004	9	16728723	BNC2	rs1411431	intron_variant	1.00E-18
GCST009003	9	16724057	BNC2	rs73646205	intron_variant	2.00E-18
GCST90179150	9	16728723	BNC2	rs1411431	intron_variant	2.00E-18
GCST90255621	9	16719447	BNC2	rs10962549	intron_variant	2.00E-17
GCST009001	9	16728534	BNC2	rs1411432	intron_variant	3.00E-17
GCST90255621	9	16763142	BNC2	rs10810619	intron_variant	1.00E-13
GCST90255621	9	15634328	CCDC171	rs4740619	intron_variant	2.00E-33
GCST90179150	9	15634328	CCDC171	rs4740619	intron_variant	1.00E-31
GCST009004	9	15634328	CCDC171	rs4740619	intron_variant	3.00E-31
GCST009003	9	15634328	CCDC171	rs4740619	intron_variant	3.00E-31
GCST009001	9	15634328	CCDC171	rs4740619	intron_variant	3.00E-31
GCST009871	9	15647350	CCDC171	rs9776097	intron_variant	9.00E-26
GCST007039	9	15910046	CCDC171	rs13292699	intron_variant	2.00E-25
GCST009871	9	16016880	CCDC171	rs3008713	intron_variant	5.00E-11
GCST90255621	9	123365932	CRB2	rs1105223	missense_variant	2.00E-14
GCST90179150	9	123334243	CRB2	rs10818810	intergenic_variant	5.00E-14
GCST009004	9	123331877	CRB2	rs10985968	intergenic_variant	8.00E-14
GCST009871	9	123331498	CRB2	rs10760276	intergenic_variant	2.00E-11
GCST007039	9	123331720	CRB2	rs10760277	intergenic_variant	3.00E-11
GCST90271767	9	34124862	DCAF12	rs2275003	intron_variant	8.00E-14
GCST90271769	9	34074478	DCAF12	rs12001437	TF_binding_site_variant	3.00E-11
GCST90271771	9	115094633	DELEC1	rs1929395	intron_variant	1.00E-17
GCST90255621	9	115103547	DELEC1	rs2989518	intron_variant	3.00E-12
GCST90255621	9	115628708	DELEC1	rs2418393	intron_variant	9.00E-11
GCST90018727	9	22134095	DMRTA1	rs10811661	intergenic_variant	3.00E-25
GCST90018947	9	22130516	DMRTA1	rs10965245	regulatory_region_variant	1.00E-19
GCST004904	9	22129580	DMRTA1	rs7020996	regulatory_region_variant	6.00E-18
GCST004904	9	22134095	DMRTA1	rs10811661	intergenic_variant	5.00E-13
GCST90255621	9	137367972	EXD3	rs11507683	synonymous_variant	7.00E-17
GCST90255621	9	137383374	EXD3	rs13291830	missense_variant	1.00E-11
GCST90255621	9	130911838	FIBCD1	rs6597650	missense_variant	4.00E-17
GCST90179150	9	130908179	FIBCD1	rs4740383	intron_variant	9.00E-13
GCST007039	9	130908179	FIBCD1	rs4740383	intron_variant	3.00E-11
GCST009871	9	130907638	FIBCD1	rs3739514	intron_variant	5.00E-11
GCST009004	9	130907638	FIBCD1	rs3739514	intron_variant	7.00E-11
GCST009871	9	14777397	FREM1	rs7046483	intron_variant	7.00E-11
GCST007039	9	14775861	FREM1	rs10733289	synonymous_variant	9.00E-11
GCST90255621	9	109170062	FRRS1L - EPB41L4B	rs6477694	intergenic_variant	5.00E-17
GCST009004	9	109170062	FRRS1L - EPB41L4B	rs6477694	intergenic_variant	7.00E-14

GCST90179150	9	109170062	FRRS1L - EPB41L4B	rs6477694	intergenic_variant	4.00E-13
GCST004904	9	109170062	FRRS1L - EPB41L4B	rs6477694	intergenic_variant	2.00E-12
GCST005951	9	109170062	FRRS1L - EPB41L4B	rs6477694	intergenic_variant	5.00E-12
GCST90018947	9	109170506	FRRS1L - EPB41L4B	rs34468594	intergenic_variant	6.00E-12
GCST90255621	9	98715218	GABPB2	rs420158	regulatory_region_variant	1.00E-11
GCST90255621	9	128265703	GOLGA2	rs7871866	splice_polypyrimidine_tract_	2.00E-18
GCST009004	9	128265703	GOLGA2	rs7871866	splice_polypyrimidine_tract_	8.00E-14
GCST009001	9	128256360	GOLGA2	rs4734	3_prime_UTR_variant	2.00E-13
GCST90179150	9	128256360	GOLGA2	rs4734	3_prime_UTR_variant	9.00E-13
GCST007039	9	128263829	GOLGA2	rs113132247	intron_variant	3.00E-12
GCST009871	9	128263829	GOLGA2	rs113132247	intron_variant	5.00E-12
GCST90255621	9	6959840	KDM4C	rs7042372	intron_variant	1.00E-13
GCST006368	9	6956850	KDM4C	rs12352785	intron_variant	6.00E-13
GCST90255621	9	106309794	LINC01505	rs7024334	intron_variant	1.00E-13
GCST90018947	9	106309794	LINC01505	rs7024334	intron_variant	4.00E-13
GCST009003	9	106309794	LINC01505	rs7024334	intron_variant	5.00E-12
GCST009004	9	106309794	LINC01505	rs7024334	intron_variant	5.00E-12
GCST90179150	9	106305280	LINC01505	rs1484375	intron_variant	6.00E-11
GCST90255621	9	28414341	LINGO2	rs10968576	intron_variant	2.00E-75
GCST90179150	9	28414341	LINGO2	rs10968576	intron_variant	1.00E-42
GCST009004	9	28412080	LINGO2	rs2183824	intron_variant	2.00E-41
GCST009003	9	28413463	LINGO2	rs16912921	intron_variant	4.00E-41
GCST009001	9	28412377	LINGO2	rs2183825	intron_variant	4.00E-41
GCST007039	9	28425517	LINGO2	rs1412239	intron_variant	2.00E-32
GCST009871	9	28412277	LINGO2	rs7873025	intron_variant	2.00E-30
GCST90255621	9	27760948	LINGO2	rs1330199	intergenic_variant	2.00E-22
GCST90018947	9	28414627	LINGO2	rs17770336	intron_variant	3.00E-21
GCST006368	9	28414341	LINGO2	rs10968576	intron_variant	7.00E-21
GCST005951	9	28415514	LINGO2	rs10968577	intron_variant	1.00E-16
GCST90255621	9	28446610	LINGO2	rs10812830	intron_variant	3.00E-16
GCST009371	9	28414341	LINGO2	rs10968576	intron_variant	3.00E-15
GCST90255621	9	29672407	LINGO2	rs11792311	intergenic_variant	6.00E-15
GCST002783	9	28414341	LINGO2	rs10968576	intron_variant	2.00E-14
GCST90267268	9	28410685	LINGO2	rs1412234	intron_variant	2.00E-14
GCST90255621	9	28942540	LINGO2	rs13285474	intron_variant	2.00E-14
GCST002783	9	28414341	LINGO2	rs10968576	intron_variant	7.00E-14
GCST009004	9	27803739	LINGO2	rs3922980	intergenic_variant	8.00E-14
GCST009001	9	27803739	LINGO2	rs3922980	intergenic_variant	8.00E-14
GCST90179150	9	27803739	LINGO2	rs3922980	intergenic_variant	1.00E-13
GCST004495	9	28414341	LINGO2	rs10968576	intron_variant	3.00E-13
GCST000830	9	28414341	LINGO2	rs10968576	intron_variant	3.00E-13
GCST004557	9	28415514	LINGO2	rs10968577	intron_variant	5.00E-13
GCST009871	9	27780655	LINGO2	rs1854671	intergenic_variant	1.00E-12
GCST90255622	9	28418513	LINGO2	rs13288841	intron_variant	1.00E-12
GCST007039	9	27777014	LINGO2	rs7874154	intergenic_variant	3.00E-12
GCST004557	9	28415514	LINGO2	rs10968577	intron_variant	4.00E-12
GCST004558	9	28415514	LINGO2	rs10968577	intron_variant	4.00E-12
GCST009004	9	29716657	LINGO2	rs12238336	intergenic_variant	4.00E-12
GCST90179150	9	29716657	LINGO2	rs12238336	intergenic_variant	4.00E-12
GCST009003	9	29750169	LINGO2	rs1932133	regulatory_region_variant	7.00E-12
GCST004558	9	28415514	LINGO2	rs10968577	intron_variant	8.00E-12

GCST004497	9	28414341	LINGO2	rs10968576	intron_variant	1.00E-11
GCST004559	9	28415514	LINGO2	rs10968577	intron_variant	1.00E-11
GCST002783	9	28414341	LINGO2	rs10968576	intron_variant	1.00E-11
GCST009871	9	29708931	LINGO2	rs60131931	intergenic_variant	1.00E-11
GCST007039	9	29708931	LINGO2	rs60131931	intergenic_variant	1.00E-11
GCST90179150	9	28446610	LINGO2	rs10812830	intron_variant	2.00E-11
GCST004559	9	28415514	LINGO2	rs10968577	intron_variant	3.00E-11
GCST90255621	9	126646011	LMX1B	rs7027304	intron_variant	7.00E-23
GCST90179150	9	126698635	LMX1B	rs10733682	3_prime_UTR_variant	6.00E-20
GCST90271771	9	126628521	LMX1B	rs3829849	intron_variant	9.00E-18
GCST90255621	9	126673993	LMX1B	rs3850585	intron_variant	4.00E-17
GCST90255621	9	126705061	LMX1B	rs13292976	regulatory_region_variant	1.00E-16
GCST009004	9	126702954	LMX1B	rs867560	non_coding_transcript_exon_	1.00E-15
GCST009003	9	126702954	LMX1B	rs867560	non_coding_transcript_exon_	1.00E-15
GCST006368	9	126698635	LMX1B	rs10733682	3_prime_UTR_variant	8.00E-13
GCST009001	9	126652650	LMX1B	rs3814120	intron_variant	1.00E-12
GCST007039	9	126698635	LMX1B	rs10733682	3_prime_UTR_variant	2.00E-12
GCST90018947	9	126646011	LMX1B	rs7027304	intron_variant	6.00E-12
GCST005951	9	126698635	LMX1B	rs10733682	3_prime_UTR_variant	3.00E-11
GCST009871	9	126666398	LMX1B	rs4322101	intron_variant	4.00E-11
GCST90239604	9	126657746	LMX1B	rs16929203	intron_variant	4.00E-11
GCST90255621	9	124286958	NEK6	rs10818938	intron_variant	4.00E-12
GCST009004	9	124298152	NEK6	rs944340	intron_variant	6.00E-11
GCST90179150	9	124290188	NEK6	rs4838152	intron_variant	8.00E-11
GCST90255621	9	91418345	NFIL3	rs7869771	intron_variant	2.00E-14
GCST009004	9	91424965	NFIL3	rs3811125	TF_binding_site_variant	9.00E-13
GCST007039	9	91424691	NFIL3	rs10820852	regulatory_region_variant	9.00E-13
GCST90179150	9	91424965	NFIL3	rs3811125	TF_binding_site_variant	1.00E-12
GCST009871	9	91424965	NFIL3	rs3811125	TF_binding_site_variant	8.00E-12
GCST90018947	9	91424691	NFIL3	rs10820852	regulatory_region_variant	5.00E-11
GCST90255621	9	84678542	NTRK2	rs1187352	intron_variant	3.00E-13
GCST009004	9	84678542	NTRK2	rs1187352	intron_variant	3.00E-11
GCST90179150	9	93698731	PHF2	rs10992867	intergenic_variant	2.00E-17
GCST90018947	9	93686011	PHF2	rs398011514	intergenic_variant	1.00E-15
GCST007039	9	93668465	PHF2	rs2398861	intron_variant	1.00E-14
GCST90255621	9	93713024	PHF2	rs7045925	intergenic_variant	7.00E-14
GCST90255621	9	93587025	PHF2	rs7041943	intron_variant	2.00E-13
GCST009004	9	93641085	PHF2	rs10761247	intron_variant	5.00E-11
GCST007039	9	78734692	PSAT1	rs725959	intergenic_variant	2.00E-12
GCST009871	9	78719768	PSAT1	rs1634350	intergenic_variant	8.00E-12
GCST009004	9	78719768	PSAT1	rs1634350	intergenic_variant	3.00E-11
GCST90179150	9	78719768	PSAT1	rs1634350	intergenic_variant	4.00E-11
GCST90255621	9	15509726	PSIP1	rs62571016	intron_variant	1.00E-14
GCST90179150	9	15511801	PSIP1	rs62571018	regulatory_region_variant	2.00E-14
GCST009871	9	15511801	PSIP1	rs62571018	regulatory_region_variant	5.00E-14
GCST90255621	9	10119157	PTPRD	rs10118866	intron_variant	3.00E-12
GCST90255621	9	8845911	PTPRD	rs1865341	intron_variant	4.00E-11
GCST90255621	9	89576341	SEMA4D - GADD45G	rs10797115	intron_variant	4.00E-16
GCST009004	9	89592393	SEMA4D - GADD45G	rs7357754	intron_variant	2.00E-12
GCST009003	9	89593312	SEMA4D - GADD45G	rs1329733	intron_variant	3.00E-12
GCST90018947	9	89591869	SEMA4D - GADD45G	rs769359210	intron_variant	6.00E-12

GCST90179150	9	89538473	SEMA4D - GADD45G	rs17440913	intergenic_variant	1.00E-11
GCST009871	9	89584356	SEMA4D - GADD45G	rs10908900	intron_variant	2.00E-11
GCST90179150	9	89593312	SEMA4D - GADD45G	rs1329733	intron_variant	2.00E-11
GCST007039	9	89563557	SEMA4D - GADD45G	rs3949781	non_coding_transcript_exon_	4.00E-11
GCST90255621	9	89504664	SEMA4D - GADD45G	rs13287131	intergenic_variant	8.00E-11
GCST90255621	9	100349683	TEX10	rs10989064	intron_variant	6.00E-25
GCST90179150	9	100345896	TEX10	rs9299338	intron_variant	1.00E-18
GCST007039	9	100345896	TEX10	rs9299338	intron_variant	2.00E-16
GCST90018947	9	100351370	TEX10	rs3931548	intron_variant	1.00E-12
GCST90255621	9	81580274	TLE1	rs4877217	intergenic_variant	1.00E-12
GCST90255621	9	81694033	TLE1	rs2796441	intron_variant	9.00E-11
GCST90255621	9	71169348	TRPM3	rs4745062	intron_variant	5.00E-16
GCST007039	9	71254839	TRPM3	rs10868998	intron_variant	4.00E-12
GCST009004	9	71222239	TRPM3	rs2134858	intron_variant	6.00E-12
GCST009871	9	71162861	TRPM3	rs7038966	intron_variant	7.00E-12
GCST90179150	9	71221315	TRPM3	rs1504398	intron_variant	9.00E-12
GCST009001	9	71165996	TRPM3	rs10746862	intron_variant	3.00E-11
GCST90255621	9	33827696	UBE2R2	rs10971721	intron_variant	8.00E-20
GCST009004	9	33827696	UBE2R2	rs10971721	intron_variant	3.00E-13
GCST90179150	9	33820940	UBE2R2	rs10971712	intron_variant	4.00E-13
GCST90255621	9	33804815	UBE2R2	rs10971709	intron_variant	1.00E-11
GCST90255621	9	23362313	UMO2P2	rs11793831	intron_variant	7.00E-17
GCST90255621	9	23199447	UMO2P2	rs10811867	intron_variant	3.00E-14
GCST009871	9	23227374	UMO2P2	rs1339139	intron_variant	3.00E-11
GCST007039	9	23228277	UMO2P2	rs1934102	intron_variant	3.00E-11
GCST90179150	9	37200106	ZCCHC7	rs6476617	intron_variant	5.00E-18
GCST90255621	9	37209399	ZCCHC7	rs1472169	intron_variant	7.00E-18
GCST009004	9	37200106	ZCCHC7	rs6476617	intron_variant	2.00E-17
GCST009003	9	37348062	ZCCHC7	rs308499	intron_variant	1.00E-16
GCST007039	9	37127681	ZCCHC7	rs13293465	intron_variant	5.00E-13
GCST90255621	10	74663981	ADH1B	rs946185	intron_variant	1.00E-14
GCST009004	10	74287706	ADK	rs12098284	intron_variant	1.00E-12
GCST009003	10	74287706	ADK	rs12098284	intron_variant	1.00E-12
GCST90255621	10	74420577	ADK	rs11001034	intron_variant	2.00E-12
GCST007039	10	74603349	ADK	rs10824211	intron_variant	3.00E-12
GCST90179150	10	74287706	ADK	rs12098284	intron_variant	4.00E-12
GCST009871	10	74295203	ADK	rs112921972	intron_variant	1.00E-11
GCST009871	10	74663981	ADK	rs946185	intron_variant	4.00E-11
GCST90255621	10	27028911	ANKDD1B	rs10829163	missense_variant	2.00E-15
GCST008129	10	27028911	ANKRD26	rs10829163	missense_variant	2.00E-12
GCST007039	10	102875591	ARPP21	rs12765002	intron_variant	1.00E-13
GCST009871	10	102875591	AS3MT	rs12765002	intron_variant	9.00E-13
GCST004904	10	102856906	ASIC2	rs4409766	intron_variant	6.00E-17
GCST006368	10	102856906	ASMT	rs4409766	intron_variant	2.00E-12
GCST90018727	10	102856906	ASMT	rs4409766	intron_variant	6.00E-12
GCST009001	10	102876519	ASMT	rs77335224	intron_variant	1.00E-11
GCST004904	10	102856906	ASMT	rs4409766	intron_variant	5.00E-11
GCST90018947	10	18284725	CACNB2	rs72784229	intron_variant	5.00E-13
GCST90255621	10	18265659	CACNB2	rs1857423	intron_variant	6.00E-11
GCST90255621	10	102959339	CNNM2	rs12413409	intron_variant	2.00E-44
GCST90179150	10	102925542	CNNM2	rs12411886	intron_variant	8.00E-20

GCST90018947	10	102988252	CNNM2	rs77860422	intron_variant	2.00E-17
GCST90255621	10	102961968	CNNM2	rs11191478	intron_variant	5.00E-13
GCST005951	10	103013607	CNNM2	rs11191514	intron_variant	3.00E-11
GCST009871	10	98013128	CRTAC1	rs522110	intron_variant	6.00E-23
GCST007039	10	98018469	CRTAC1	rs2439823	intron_variant	1.00E-22
GCST009004	10	98018469	CRTAC1	rs2439823	intron_variant	7.00E-22
GCST009001	10	98038477	CRTAC1	rs10883027	TF_binding_site_variant	7.00E-22
GCST009003	10	98012647	CRTAC1	rs563296	intron_variant	9.00E-22
GCST90179150	10	98018469	CRTAC1	rs2439823	intron_variant	5.00E-21
GCST90255621	10	98009631	CRTAC1	rs577525	intron_variant	7.00E-20
GCST90018947	10	98024796	CRTAC1	rs112213274	intron_variant	5.00E-19
GCST009871	10	98101555	CRTAC1	rs10786387	regulatory_region_variant	4.00E-17
GCST90267268	10	97935213	CRTAC1	rs472576	intron_variant	2.00E-12
GCST90179150	10	98128586	CRTAC1	rs928579	intergenic_variant	3.00E-12
GCST009871	10	97939041	CRTAC1	rs61875762	intron_variant	7.00E-12
GCST009003	10	125007104	CTBP2	rs1561589	intron_variant	1.00E-15
GCST009871	10	124995197	CTBP2	rs11245453	intron_variant	4.00E-13
GCST009871	10	125041645	CTBP2	rs4962424	intron_variant	3.00E-11
GCST90255621	10	116861485	ENO4	rs2170862	intron_variant	4.00E-15
GCST90179150	10	116857026	ENO4	rs7087685	intron_variant	1.00E-14
GCST009871	10	116891485	ENO4	rs10510025	intron_variant	1.00E-13
GCST007039	10	116891485	ENO4	rs10510025	intron_variant	7.00E-13
GCST90255621	10	85651147	GRID1	rs7899106	intron_variant	3.00E-24
GCST009004	10	85651147	GRID1	rs7899106	intron_variant	2.00E-18
GCST009003	10	85651147	GRID1	rs7899106	intron_variant	2.00E-18
GCST90179150	10	85651147	GRID1	rs7899106	intron_variant	5.00E-18
GCST90179150	10	86359258	GRID1	rs2114824	intron_variant	3.00E-14
GCST90255621	10	86336290	GRID1	rs10887578	intron_variant	2.00E-13
GCST005951	10	85651147	GRID1	rs7899106	intron_variant	3.00E-13
GCST009871	10	85731093	GRID1	rs17399739	intron_variant	7.00E-13
GCST007039	10	85731093	GRID1	rs17399739	intron_variant	1.00E-12
GCST007039	10	86336290	GRID1	rs10887578	intron_variant	2.00E-11
GCST006368	10	85651147	GRID1	rs7899106	intron_variant	3.00E-11
GCST009004	10	86352566	GRID1	rs10887584	intron_variant	5.00E-11
GCST90255621	10	100635683	HIF1AN	rs17094222	intergenic_variant	7.00E-27
GCST90179150	10	100635683	HIF1AN	rs17094222	intergenic_variant	3.00E-18
GCST009001	10	100635683	HIF1AN	rs17094222	intergenic_variant	4.00E-18
GCST004904	10	100635683	HIF1AN	rs17094222	intergenic_variant	2.00E-15
GCST006368	10	100635683	HIF1AN	rs17094222	intergenic_variant	6.00E-15
GCST90255621	10	100652186	HIF1AN	rs4075098	intergenic_variant	5.00E-14
GCST009871	10	100660414	HIF1AN	rs72838588	intergenic_variant	2.00E-12
GCST002783	10	100635683	HIF1AN	rs17094222	intergenic_variant	2.00E-11
GCST002783	10	100635683	HIF1AN	rs17094222	intergenic_variant	6.00E-11
GCST90255621	10	132179185	JAKMIP3	rs4880341	intron_variant	1.00E-17
GCST009004	10	132179185	JAKMIP3	rs4880341	intron_variant	3.00E-14
GCST009003	10	132179185	JAKMIP3	rs4880341	intron_variant	3.00E-14
GCST90179150	10	132179185	JAKMIP3	rs4880341	intron_variant	9.00E-14
GCST009871	10	132165458	JAKMIP3	rs2172131	intron_variant	6.00E-13
GCST007039	10	132165458	JAKMIP3	rs2172131	intron_variant	6.00E-13
GCST009871	10	63431885	JMJD1C	rs7924036	intron_variant	4.00E-13
GCST007039	10	63431885	JMJD1C	rs7924036	intron_variant	6.00E-12

GCST90255621	10	77001201	KCNMA1	rs11001963	intron_variant	8.00E-12
GCST009004	10	77001201	KCNMA1	rs11001963	intron_variant	9.00E-11
GCST90179150	10	77001201	KCNMA1	rs11001963	intron_variant	9.00E-11
GCST90255621	10	33677392	LINC02628 - LINC00838	rs12782894	intergenic_variant	5.00E-16
GCST009871	10	33681003	LINC02628 - LINC00838	rs12762034	regulatory_region_variant	4.00E-15
GCST007039	10	33681003	LINC02628 - LINC00838	rs12762034	regulatory_region_variant	1.00E-14
GCST009004	10	33724579	LINC02628 - LINC00838	rs12765914	intergenic_variant	2.00E-13
GCST009003	10	33698401	LINC02628 - LINC00838	rs34930419	intron_variant	6.00E-13
GCST90179150	10	33725725	LINC02628 - LINC00838	rs34413031	intergenic_variant	1.00E-12
GCST90018947	10	33725507	LINC02628 - LINC00838	rs71495049	intergenic_variant	8.00E-11
GCST90018947	10	123458659	LINC02641	rs705139	intron_variant	5.00E-17
GCST90255621	10	123458380	LINC02641	rs845085	intron_variant	4.00E-16
GCST007039	10	123460520	LINC02641	rs845084	intron_variant	9.00E-14
GCST009871	10	123460520	LINC02641	rs845084	intron_variant	1.00E-13
GCST90255621	10	123437356	LINC02641	rs705128	intron_variant	2.00E-13
GCST004904	10	123492235	LINC02641	rs1568079	intron_variant	2.00E-12
GCST009004	10	123460520	LINC02641	rs845084	intron_variant	3.00E-12
GCST90179150	10	123460520	LINC02641	rs845084	intron_variant	5.00E-12
GCST90018727	10	123484138	LINC02641	rs7070259	intron_variant	5.00E-11
GCST90255621	10	98257696	LOXL4	rs1983864	missense_variant	3.00E-29
GCST008129	10	98257696	LOXL4	rs1983864	missense_variant	8.00E-16
GCST90255621	10	98275126	LOXL4	rs12784535	intergenic_variant	5.00E-11
GCST90255621	10	75774242	LRMDA	rs4745794	intron_variant	8.00E-15
GCST009004	10	75896751	LRMDA	rs4746339	intron_variant	1.00E-11
GCST009003	10	75885471	LRMDA	rs10824347	intron_variant	5.00E-11
GCST90179150	10	75899485	LRMDA	rs10762686	intron_variant	5.00E-11
GCST90255621	10	21532345	MLLT10	rs7084454	intron_variant	5.00E-33
GCST009003	10	21532345	MLLT10	rs7084454	intron_variant	5.00E-27
GCST009004	10	21532345	MLLT10	rs7084454	intron_variant	5.00E-27
GCST90179150	10	21532345	MLLT10	rs7084454	intron_variant	1.00E-26
GCST009001	10	21532989	MLLT10	rs12251016	intron_variant	1.00E-25
GCST007039	10	21541175	MLLT10	rs11012732	intron_variant	2.00E-25
GCST009871	10	21541175	MLLT10	rs11012732	intron_variant	5.00E-25
GCST90018947	10	21541175	MLLT10	rs11012732	intron_variant	4.00E-20
GCST009003	10	102224303	NOLC1 - ELOVL3	rs7083450	intergenic_variant	2.00E-13
GCST90255621	10	102224303	NOLC1 - ELOVL3	rs7083450	intergenic_variant	2.00E-13
GCST90179150	10	102224303	NOLC1 - ELOVL3	rs7083450	intergenic_variant	3.00E-13
GCST90255621	10	102207159	NOLC1 - ELOVL3	rs147506742	intergenic_variant	7.00E-11
GCST90255621	10	124905509	NPM1P31 - ZRANB1	rs17636031	intergenic_variant	8.00E-21
GCST009004	10	124905509	NPM1P31 - ZRANB1	rs17636031	intergenic_variant	4.00E-17
GCST90179150	10	124905509	NPM1P31 - ZRANB1	rs17636031	intergenic_variant	8.00E-17
GCST007039	10	124898919	NPM1P31 - ZRANB1	rs72828935	regulatory_region_variant	6.00E-16
GCST011331	10	103111522	NT5C2	rs10883832	intron_variant	3.00E-24
GCST011330	10	103111522	NT5C2	rs10883832	intron_variant	9.00E-16
GCST006368	10	103109281	NT5C2	rs11191560	intron_variant	8.00E-15
GCST90255621	10	103192742	NT5C2	rs79780963	intron_variant	1.00E-12
GCST90179150	10	103192742	NT5C2	rs79780963	intron_variant	1.00E-11
GCST005951	10	103146454	NT5C2	rs11191580	intron_variant	2.00E-11
GCST009871	10	103183291	NT5C2	rs11191595	intron_variant	6.00E-11
GCST90255621	10	100687890	PAX2	rs41310284	intergenic_variant	3.00E-26
GCST007039	10	100687890	PAX2	rs41310284	intergenic_variant	5.00E-21

GCST009871	10	100687890	PAX2	rs41310284	intergenic_variant	3.00E-20
GCST90179150	10	100687890	PAX2	rs41310284	intergenic_variant	8.00E-20
GCST009004	10	100687890	PAX2	rs41310284	intergenic_variant	4.00E-19
GCST009871	10	100866753	PAX2	rs61873510	regulatory_region_variant	3.00E-17
GCST009871	10	100710675	PAX2	rs7916385	intergenic_variant	8.00E-16
GCST90179150	10	100875718	PAX2	rs10883553	regulatory_region_variant	1.00E-15
GCST90255621	10	100875718	PAX2	rs10883553	regulatory_region_variant	1.00E-14
GCST90018947	10	100687890	PAX2	rs41310284	intergenic_variant	2.00E-14
GCST009004	10	100875096	PAX2	rs34277166	regulatory_region_variant	8.00E-14
GCST009001	10	100875096	PAX2	rs34277166	regulatory_region_variant	8.00E-14
GCST90179150	10	100710675	PAX2	rs7916385	intergenic_variant	6.00E-13
GCST90255621	10	100860798	PAX2	rs17113518	intergenic_variant	6.00E-13
GCST90255621	10	100811803	PAX2	rs876670	intron_variant	4.00E-11
GCST009004	10	63559006	REEP3	rs10761785	intron_variant	3.00E-16
GCST009003	10	63559006	REEP3	rs10761785	intron_variant	3.00E-16
GCST90179150	10	63559006	REEP3	rs10761785	intron_variant	2.00E-14
GCST90255621	10	63559006	REEP3	rs10761785	intron_variant	1.00E-13
GCST90018947	10	63554059	REEP3	rs9919429	intron_variant	7.00E-12
GCST90018947	10	121138445	RPL19P16 - LINC01153	rs1907240	regulatory_region_variant	8.00E-15
GCST90018727	10	121138470	RPL19P16 - LINC01153	rs1907241	regulatory_region_variant	3.00E-13
GCST004904	10	121138445	RPL19P16 - LINC01153	rs1907240	regulatory_region_variant	3.00E-11
GCST004904	10	121139183	RPL19P16 - LINC01153	rs2257129	regulatory_region_variant	4.00E-11
GCST90255621	10	16708130	RSU1	rs7893571	intron_variant	2.00E-12
GCST009004	10	16708130	RSU1	rs7893571	intron_variant	6.00E-12
GCST007039	10	16708130	RSU1	rs7893571	intron_variant	8.00E-12
GCST90179150	10	16708130	RSU1	rs7893571	intron_variant	1.00E-11
GCST009871	10	16717313	RSU1	rs10795422	intron_variant	5.00E-11
GCST90018947	10	16732125	RSU1	rs10630240	intron_variant	6.00E-11
GCST009004	10	116913020	SHTN1	rs10886017	intron_variant	4.00E-15
GCST009003	10	116913020	SHTN1	rs10886017	intron_variant	4.00E-15
GCST011329	10	112998590	TCF7L2	rs7903146	intron_variant	2.00E-77
GCST90255621	10	112998590	TCF7L2	rs7903146	intron_variant	2.00E-66
GCST009004	10	112998590	TCF7L2	rs7903146	intron_variant	2.00E-23
GCST009001	10	112998590	TCF7L2	rs7903146	intron_variant	2.00E-23
GCST90179150	10	112998590	TCF7L2	rs7903146	intron_variant	5.00E-23
GCST009003	10	112996282	TCF7L2	rs4506565	intron_variant	2.00E-22
GCST011332	10	112998590	TCF7L2	rs7903146	intron_variant	4.00E-19
GCST004904	10	112998590	TCF7L2	rs7903146	intron_variant	1.00E-18
GCST004045	10	112996282	TCF7L2	rs4506565	intron_variant	8.00E-17
GCST004046	10	112996282	TCF7L2	rs4506565	intron_variant	4.00E-16
GCST011331	10	112998590	TCF7L2	rs7903146	intron_variant	2.00E-15
GCST009121	10	113008012	TCF7L2	rs4132670	intron_variant	3.00E-15
GCST007039	10	112995025	TCF7L2	rs35198068	intron_variant	1.00E-14
GCST001527	10	112998590	TCF7L2	rs7903146	intron_variant	2.00E-14
GCST90018947	10	112995025	TCF7L2	rs35198068	intron_variant	2.00E-14
GCST009871	10	112995025	TCF7L2	rs35198068	intron_variant	3.00E-14
GCST006368	10	112998590	TCF7L2	rs7903146	intron_variant	3.00E-14
GCST005951	10	112998590	TCF7L2	rs7903146	intron_variant	3.00E-13
GCST009764	10	112998590	TCF7L2	rs7903146	intron_variant	3.00E-13
GCST005952	10	112992744	TCF7L2	rs17747324	intron_variant	3.00E-13
GCST001527	10	113029056	TCF7L2	rs12243326	intron_variant	7.00E-13

GCST002783	10	112998590	TCF7L2	rs7903146	intron_variant	1.00E-12
GCST90255621	10	113060432	TCF7L2	rs12245680	intron_variant	7.00E-12
GCST005950	10	112992744	TCF7L2	rs17747324	intron_variant	8.00E-12
GCST004904	10	112998590	TCF7L2	rs7903146	intron_variant	9.00E-12
GCST002783	10	112998590	TCF7L2	rs7903146	intron_variant	1.00E-11
GCST009107	10	113008012	TCF7L2	rs4132670	intron_variant	4.00E-11
GCST002783	10	112998590	TCF7L2	rs7903146	intron_variant	4.00E-11
GCST009871	11	43857990	ALKAL2	rs11037685	intron_variant	2.00E-14
GCST90255621	11	43878051	ALKBH3	rs2434467	non_coding_transcript_exon_	4.00E-14
GCST009004	11	43878051	ALKBH3	rs2434467	non_coding_transcript_exon_	2.00E-11
GCST007039	11	43913042	ALKBH3	rs7115013	intron_variant	5.00E-11
GCST90179150	11	43915395	ALKBH3	rs10768994	intron_variant	6.00E-11
GCST009871	11	43878051	ALKBH3	rs2434467	non_coding_transcript_exon_	7.00E-11
GCST90255621	11	72722053	APOE	rs1552224	5_prime_UTR_variant	2.00E-15
GCST001527	11	72721940	ARAP1	rs11603334	5_prime_UTR_variant	2.00E-14
GCST011329	11	72721940	ARAP1	rs11603334	5_prime_UTR_variant	2.00E-11
GCST009001	11	30332178	ARHGAP15	rs1765139	intron_variant	5.00E-14
GCST90179150	11	30332178	ARL14EP	rs1765139	intron_variant	7.00E-14
GCST90018947	11	134648974	AXIN1	rs12806052	intergenic_variant	1.00E-11
GCST009871	11	134652703	B3GAT1	rs35025195	intergenic_variant	3.00E-11
GCST90255621	11	371265	B3GAT1	rs11246136	intron_variant	5.00E-13
GCST90271770	11	370252	B4GALNT4	rs76560824	intron_variant	9.00E-12
GCST009871	11	64281549	BACE2	rs34292685	intron_variant	1.00E-13
GCST007039	11	64281549	BAD, GPR137	rs34292685	intron_variant	1.00E-13
GCST009003	11	64281549	BAD, GPR137	rs34292685	intron_variant	7.00E-13
GCST90255621	11	27658369	BCL7A	rs6265	missense_variant	1.00E-122
GCST009004	11	27658369	BDNF	rs6265	missense_variant	7.00E-89
GCST009003	11	27658369	BDNF	rs6265	missense_variant	7.00E-89
GCST009001	11	27658369	BDNF	rs6265	missense_variant	7.00E-89
GCST90179150	11	27658369	BDNF	rs6265	missense_variant	3.00E-83
GCST008129	11	27658369	BDNF	rs6265	missense_variant	3.00E-68
GCST90018947	11	27682662	BDNF	rs34379767	intron_variant	7.00E-63
GCST007039	11	27658369	BDNF	rs6265	missense_variant	8.00E-61
GCST009871	11	27658369	BDNF	rs6265	missense_variant	3.00E-58
GCST90255621	11	27561582	BDNF	rs7481311	intron_variant	9.00E-57
GCST004904	11	27658369	BDNF	rs6265	missense_variant	2.00E-51
GCST009871	11	27706555	BDNF	rs11030119	intron_variant	6.00E-51
GCST90179150	11	27585996	BDNF	rs10835197	intron_variant	1.00E-44
GCST009121	11	27672694	BDNF	rs2049045	intron_variant	8.00E-42
GCST006368	11	27658369	BDNF	rs6265	missense_variant	2.00E-39
GCST005951	11	27680836	BDNF	rs16917237	intron_variant	1.00E-34
GCST002783	11	27662970	BDNF	rs11030104	intron_variant	7.00E-30
GCST004904	11	27656039	BDNF	rs11030100	3_prime_UTR_variant	1.00E-28
GCST002783	11	27662970	BDNF	rs11030104	intron_variant	6.00E-28
GCST90018727	11	27646808	BDNF	rs16917204	intron_variant	2.00E-27
GCST000830	11	27704439	BDNF	rs10767664	intron_variant	5.00E-26
GCST004497	11	27662970	BDNF	rs11030104	intron_variant	7.00E-24
GCST004557	11	27678578	BDNF	rs7103411	intron_variant	2.00E-22
GCST004495	11	27662970	BDNF	rs11030104	intron_variant	5.00E-22
GCST004558	11	27678578	BDNF	rs7103411	intron_variant	2.00E-21
GCST002461	11	27662970	BDNF	rs11030104	intron_variant	2.00E-20

GCST004558	11	27706992	BDNF	rs2030323	intron_variant	4.00E-20
GCST004557	11	27678578	BDNF	rs7103411	intron_variant	2.00E-19
GCST002783	11	27662970	BDNF	rs11030104	intron_variant	5.00E-19
GCST001416	11	27706992	BDNF	rs2030323	intron_variant	4.00E-16
GCST004557	11	27706992	BDNF	rs2030323	intron_variant	9.00E-16
GCST004499	11	27662970	BDNF	rs11030104	intron_variant	1.00E-15
GCST002783	11	27662970	BDNF	rs11030104	intron_variant	2.00E-15
GCST004558	11	27706992	BDNF	rs2030323	intron_variant	3.00E-15
GCST004559	11	27678578	BDNF	rs7103411	intron_variant	3.00E-15
GCST006802	11	27662970	BDNF	rs11030104	intron_variant	7.00E-15
GCST90255621	11	27769631	BDNF	rs10835219	intron_variant	1.00E-14
GCST009871	11	27697875	BDNF	rs76324918	non_coding_transcript_exon_	3.00E-14
GCST004497	11	27662970	BDNF	rs11030104	intron_variant	3.00E-14
GCST009871	11	27620761	BDNF	rs12276130	non_coding_transcript_exon_	6.00E-14
GCST004495	11	27662970	BDNF	rs11030104	intron_variant	1.00E-13
GCST90255621	11	27724217	BDNF	rs908867	intron_variant	2.00E-13
GCST004557	11	27706992	BDNF	rs2030323	intron_variant	3.00E-13
GCST004559	11	27672694	BDNF	rs2049045	intron_variant	1.00E-12
GCST004558	11	27706992	BDNF	rs2030323	intron_variant	2.00E-12
GCST90179150	11	27724217	BDNF	rs908867	intron_variant	2.00E-12
GCST008158	11	27618676	BDNF	rs10767654	intron_variant	2.00E-12
GCST90255621	11	27686279	BDNF	rs145596917	intron_variant	4.00E-12
GCST004559	11	27678578	BDNF	rs7103411	intron_variant	1.00E-11
GCST009871	11	27709325	BDNF	rs144089176	intron_variant	2.00E-11
GCST009764	11	27654165	BDNF	rs1519480	intron_variant	2.00E-11
GCST90255622	11	27648561	BDNF	rs10501087	intron_variant	4.00E-11
GCST004557	11	27635242	BDNF	rs4923460	intron_variant	5.00E-11
GCST004495	11	27662970	BDNF	rs11030104	intron_variant	5.00E-11
GCST004497	11	27662970	BDNF	rs11030104	intron_variant	5.00E-11
GCST004498	11	27662970	BDNF	rs11030104	intron_variant	6.00E-11
GCST90255621	11	13328584	BLTP3A	rs1982350	intron_variant	4.00E-26
GCST009004	11	13328584	BMAL1	rs1982350	intron_variant	2.00E-18
GCST007039	11	13272721	BMAL1	rs900144	intergenic_variant	3.00E-18
GCST90179150	11	13304148	BMAL1	rs4757142	intron_variant	4.00E-18
GCST009001	11	13267151	BMAL1	rs7942486	intergenic_variant	4.00E-18
GCST009003	11	13324747	BMAL1	rs11824092	intron_variant	5.00E-18
GCST009871	11	13328012	BMAL1	rs28711392	intron_variant	4.00E-17
GCST006368	11	13298986	BMAL1	rs7938308	intron_variant	8.00E-15
GCST90018947	11	13304148	BMAL1	rs4757142	intron_variant	8.00E-13
GCST009871	11	13209377	BMAL1	rs10500775	intergenic_variant	5.00E-11
GCST90179150	11	115174130	CADM1	rs1048932	3_prime_UTR_variant	2.00E-27
GCST009004	11	115151684	CADM1	rs12286929	intergenic_variant	2.00E-27
GCST009001	11	115151684	CADM1	rs12286929	intergenic_variant	2.00E-27
GCST009003	11	115174130	CADM1	rs1048932	3_prime_UTR_variant	4.00E-27
GCST90255621	11	115174130	CADM1	rs1048932	3_prime_UTR_variant	7.00E-25
GCST90018947	11	115174130	CADM1	rs1048932	3_prime_UTR_variant	5.00E-18
GCST009871	11	115174130	CADM1	rs1048932	3_prime_UTR_variant	9.00E-17
GCST007039	11	115174130	CADM1	rs1048932	3_prime_UTR_variant	1.00E-16
GCST006368	11	115151684	CADM1	rs12286929	intergenic_variant	6.00E-16
GCST004904	11	115174130	CADM1	rs1048932	3_prime_UTR_variant	1.00E-15
GCST002783	11	115151684	CADM1	rs12286929	intergenic_variant	5.00E-13

GCST002783	11	115151684	CADM1	rs12286929	intergenic_variant	1.00E-12
GCST005951	11	115151684	CADM1	rs12286929	intergenic_variant	1.00E-11
GCST90255621	11	69490649	CCND1	rs10796821	non_coding_transcript_exon_	3.00E-20
GCST009004	11	69630405	CCND1	rs592483	regulatory_region_variant	2.00E-16
GCST009003	11	69630405	CCND1	rs592483	regulatory_region_variant	2.00E-16
GCST90179150	11	69630405	CCND1	rs592483	regulatory_region_variant	1.00E-15
GCST007039	11	69628195	CCND1	rs1982774	intergenic_variant	2.00E-13
GCST009871	11	69628195	CCND1	rs1982774	intergenic_variant	4.00E-13
GCST009871	11	69492243	CCND1	rs4980662	non_coding_transcript_exon_	1.00E-11
GCST90255621	11	69600013	CCND1	rs7122472	regulatory_region_variant	1.00E-11
GCST90179150	11	69485003	CCND1	rs587230	intron_variant	3.00E-11
GCST009004	11	47508395	CELF1	rs7124681	intron_variant	4.00E-55
GCST009001	11	47508395	CELF1	rs7124681	intron_variant	4.00E-55
GCST009003	11	47508395	CELF1	rs7124681	intron_variant	4.00E-55
GCST90179150	11	47508395	CELF1	rs7124681	intron_variant	4.00E-53
GCST007039	11	47508395	CELF1	rs7124681	intron_variant	1.00E-39
GCST90018947	11	47544800	CELF1	rs7928842	intron_variant	1.00E-35
GCST006368	11	47508395	CELF1	rs7124681	intron_variant	1.00E-19
GCST005951	11	47508395	CELF1	rs7124681	intron_variant	3.00E-19
GCST004557	11	47508395	CELF1	rs7124681	intron_variant	5.00E-12
GCST004557	11	47508395	CELF1	rs7124681	intron_variant	1.00E-11
GCST004558	11	47508395	CELF1	rs7124681	intron_variant	2.00E-11
GCST004558	11	47508395	CELF1	rs7124681	intron_variant	6.00E-11
GCST90255621	11	892089	CHID1	rs7952102	intron_variant	2.00E-13
GCST90179150	11	891338	CHID1	rs12421848	intron_variant	3.00E-13
GCST007039	11	892089	CHID1	rs7952102	intron_variant	6.00E-13
GCST006368	11	45416824	CHST1	rs10742752	intron_variant	6.00E-16
GCST90179150	11	45404591	CHST1	rs10838465	intron_variant	6.00E-14
GCST90255621	11	45416824	CHST1	rs10742752	intron_variant	8.00E-14
GCST009871	11	45398683	CHST1	rs34042421	non_coding_transcript_exon_	9.00E-12
GCST007039	11	45398683	CHST1	rs34042421	non_coding_transcript_exon_	2.00E-11
GCST90255621	11	85103349	DLG2	rs349088	intron_variant	9.00E-15
GCST009004	11	85103349	DLG2	rs349088	intron_variant	4.00E-14
GCST90179150	11	85103349	DLG2	rs349088	intron_variant	1.00E-13
GCST009001	11	85065805	DLG2	rs349071	intron_variant	4.00E-13
GCST007039	11	85065805	DLG2	rs349071	intron_variant	9.00E-13
GCST009871	11	85065805	DLG2	rs349071	intron_variant	7.00E-12
GCST90255621	11	28741774	EEF1A1P47	rs7948120	intron_variant	3.00E-18
GCST90255621	11	29229363	EEF1A1P47	rs4923597	intron_variant	2.00E-17
GCST90179150	11	28720673	EEF1A1P47	rs491711	intron_variant	2.00E-11
GCST90179150	11	29229363	EEF1A1P47	rs4923597	intron_variant	2.00E-11
GCST009003	11	29209187	EEF1A1P47	rs2452141	intron_variant	7.00E-11
GCST90255621	11	65871903	EFEMP2	rs2234458	non_coding_transcript_exon_	1.00E-21
GCST007039	11	65871903	EFEMP2	rs2234458	non_coding_transcript_exon_	2.00E-21
GCST90018947	11	65874460	EFEMP2	rs625652	intergenic_variant	3.00E-18
GCST004046	11	61803311	FADS1, FADS2	rs174547	intron_variant	1.00E-29
GCST004045	11	61803311	FADS1, FADS2	rs174547	intron_variant	7.00E-25
GCST90255621	11	64056147	FLRT1	rs562664	intron_variant	4.00E-12
GCST011336	11	64101590	FLRT1	rs2845885	intron_variant	8.00E-11
GCST90271770	11	76754228	GUCY2EP - TSKU	rs7942337	intergenic_variant	2.00E-15
GCST90271767	11	76781151	GUCY2EP - TSKU	rs7107409	TF_binding_site_variant	2.00E-13

GCST009004	11	76764986	GUCY2EP - TSKU	rs12282785	intron_variant	1.00E-11
GCST90179150	11	76756303	GUCY2EP - TSKU	rs11236924	regulatory_region_variant	6.00E-11
GCST90255621	11	43632283	HSD17B12	rs2862996	intron_variant	3.00E-35
GCST009004	11	43632283	HSD17B12	rs2862996	intron_variant	4.00E-35
GCST009001	11	43632283	HSD17B12	rs2862996	intron_variant	4.00E-35
GCST90179150	11	43632283	HSD17B12	rs2862996	intron_variant	8.00E-35
GCST009003	11	43634985	HSD17B12	rs2862961	intron_variant	6.00E-34
GCST009871	11	43670873	HSD17B12	rs59227842	intron_variant	1.00E-28
GCST007039	11	43670873	HSD17B12	rs59227842	intron_variant	4.00E-28
GCST90018947	11	43828363	HSD17B12	rs9651614	non_coding_transcript_exon_	1.00E-21
GCST008129	11	43855148	HSD17B12	rs11555762	missense_variant	5.00E-14
GCST009871	11	43843391	HSD17B12	rs117242440	intron_variant	8.00E-12
GCST90255621	11	133897727	IGSF9B	rs329651	3_prime_UTR_variant	1.00E-14
GCST90179150	11	133897727	IGSF9B	rs329651	3_prime_UTR_variant	4.00E-14
GCST007039	11	133897727	IGSF9B	rs329651	3_prime_UTR_variant	1.00E-11
GCST90255621	11	17368303	KCNJ11	rs2051772	intron_variant	5.00E-18
GCST90179150	11	17382092	KCNJ11	rs1557765	non_coding_transcript_exon_	1.00E-12
GCST90018947	11	17384070	KCNJ11	rs1002226	intron_variant	4.00E-12
GCST009004	11	17387083	KCNJ11	rs5215	missense_variant	1.00E-11
GCST004904	11	17387083	KCNJ11	rs5215	missense_variant	3.00E-11
GCST004904	11	2836003	KCNQ1	rs60808706	intron_variant	1.00E-38
GCST90018727	11	2837316	KCNQ1	rs2237897	intron_variant	9.00E-37
GCST90018947	11	2837316	KCNQ1	rs2237897	intron_variant	9.00E-37
GCST004904	11	2837316	KCNQ1	rs2237897	intron_variant	1.00E-30
GCST002461	11	2818521	KCNQ1	rs2237892	intron_variant	9.00E-13
GCST009871	11	27434512	LGR4	rs4514364	intron_variant	4.00E-18
GCST90255621	11	27401539	LGR4	rs7952482	intron_variant	5.00E-14
GCST90267268	11	27434035	LGR4	rs11030003	intron_variant	2.00E-13
GCST90179150	11	27408439	LGR4	rs11825412	intron_variant	5.00E-11
GCST009004	11	134731118	LINC02706 - LINC02714	rs12364470	intergenic_variant	2.00E-17
GCST009003	11	134731118	LINC02706 - LINC02714	rs12364470	intergenic_variant	2.00E-17
GCST90179150	11	134731118	LINC02706 - LINC02714	rs12364470	intergenic_variant	5.00E-17
GCST90255621	11	134731118	LINC02706 - LINC02714	rs12364470	intergenic_variant	8.00E-17
GCST009871	11	134731118	LINC02706 - LINC02714	rs12364470	intergenic_variant	1.00E-14
GCST007039	11	134731118	LINC02706 - LINC02714	rs12364470	intergenic_variant	3.00E-14
GCST90255621	11	69667201	LTO1	rs1789165	3_prime_UTR_variant	8.00E-21
GCST90179150	11	69694575	LTO1	rs1789170	intergenic_variant	3.00E-15
GCST009871	11	69685595	LTO1	rs4441044	regulatory_region_variant	3.00E-11
GCST90255621	11	47271906	MADD	rs7114704	intron_variant	2.00E-14
GCST001527	11	47314769	MADD	rs7944584	intron_variant	4.00E-12
GCST90271771	11	65510990	MALAT1	rs10896012	TF_binding_site_variant	5.00E-20
GCST009004	11	65510990	MALAT1	rs10896012	TF_binding_site_variant	1.00E-14
GCST90179150	11	65510990	MALAT1	rs10896012	TF_binding_site_variant	5.00E-14
GCST009871	11	65510990	MALAT1	rs10896012	TF_binding_site_variant	1.00E-12
GCST90255621	11	122051879	MIR100HG	rs11218510	intron_variant	6.00E-16
GCST009004	11	122051879	MIR100HG	rs11218510	intron_variant	7.00E-13
GCST009003	11	122051879	MIR100HG	rs11218510	intron_variant	7.00E-13
GCST009871	11	122051879	MIR100HG	rs11218510	intron_variant	2.00E-12
GCST007039	11	122051879	MIR100HG	rs11218510	intron_variant	9.00E-12
GCST90179150	11	122051879	MIR100HG	rs11218510	intron_variant	1.00E-11
GCST90255621	11	30400521	MPPED2	rs2065418	intron_variant	6.00E-27

GCST007039	11	30408785	MPPED2	rs7942037	intron_variant	3.00E-18
GCST009871	11	30408785	MPPED2	rs7942037	intron_variant	1.00E-17
GCST009004	11	30400521	MPPED2	rs2065418	intron_variant	6.00E-15
GCST90018947	11	30394229	MPPED2	rs1717776	intron_variant	2.00E-13
GCST90255621	11	47629441	MTCH2	rs3817334	intron_variant	1.00E-78
GCST008129	11	47618877	MTCH2	rs1064608	missense_variant	1.00E-31
GCST009871	11	47602338	MTCH2	rs12363232	intergenic_variant	1.00E-29
GCST004904	11	47629441	MTCH2	rs3817334	intron_variant	1.00E-24
GCST006368	11	47629441	MTCH2	rs3817334	intron_variant	6.00E-20
GCST005951	11	47629441	MTCH2	rs3817334	intron_variant	1.00E-18
GCST002783	11	47629441	MTCH2	rs3817334	intron_variant	1.00E-17
GCST002783	11	47629441	MTCH2	rs3817334	intron_variant	5.00E-17
GCST006802	11	47629441	MTCH2	rs3817334	intron_variant	2.00E-13
GCST000830	11	47629441	MTCH2	rs3817334	intron_variant	2.00E-12
GCST004557	11	47629441	MTCH2	rs3817334	intron_variant	5.00E-12
GCST004497	11	47629441	MTCH2	rs3817334	intron_variant	7.00E-12
GCST004557	11	47629441	MTCH2	rs3817334	intron_variant	1.00E-11
GCST004495	11	47629441	MTCH2	rs3817334	intron_variant	1.00E-11
GCST004558	11	47629441	MTCH2	rs3817334	intron_variant	1.00E-11
GCST002783	11	47629441	MTCH2	rs3817334	intron_variant	2.00E-11
GCST004558	11	47629441	MTCH2	rs3817334	intron_variant	5.00E-11
GCST009871	11	47599417	MTCH2	rs78339356	regulatory_region_variant	9.00E-11
GCST009871	11	90189249	NAALAD2	rs61903695	intron_variant	1.00E-11
GCST007039	11	90189249	NAALAD2	rs61903695	intron_variant	1.00E-11
GCST90018947	11	90189249	NAALAD2	rs61903695	intron_variant	3.00E-11
GCST009004	11	90189249	NAALAD2	rs61903695	intron_variant	5.00E-11
GCST90179150	11	90189249	NAALAD2	rs61903695	intron_variant	7.00E-11
GCST90255621	11	131597900	NTM	rs2512885	intron_variant	2.00E-16
GCST90255621	11	132072923	NTM	rs1793631	intron_variant	7.00E-15
GCST90179150	11	131583018	NTM	rs12788343	intron_variant	4.00E-14
GCST007039	11	131583018	NTM	rs12788343	intron_variant	1.00E-13
GCST009871	11	131609374	NTM	rs4936142	intron_variant	8.00E-13
GCST90018947	11	131007583	NTM	rs7949071	intergenic_variant	9.00E-13
GCST009004	11	132114436	NTM	rs12222235	intron_variant	2.00E-12
GCST90179150	11	132114436	NTM	rs12222235	intron_variant	2.00E-12
GCST009004	11	131607353	NTM	rs1506662	intron_variant	3.00E-12
GCST009871	11	131584054	NTM	rs1040102	intron_variant	3.00E-11
GCST006368	11	47835701	NUP160	rs3816605	missense_variant	4.00E-16
GCST009871	11	47893406	NUP160	rs78239375	intergenic_variant	8.00E-11
GCST90179150	11	132772064	OPCML	rs4936175	intron_variant	3.00E-14
GCST90255621	11	132769711	OPCML	rs2007518	intron_variant	4.00E-12
GCST009004	11	132770106	OPCML	rs2105808	intron_variant	3.00E-11
GCST007039	11	132772064	OPCML	rs4936175	intron_variant	5.00E-11
GCST009871	11	66168031	PACS1	rs801732	intron_variant	5.00E-12
GCST90255621	11	66117329	PACS1	rs527737	intron_variant	3.00E-11
GCST009871	11	45999358	PHF21A	rs79686965	intron_variant	2.00E-11
GCST007039	11	45999358	PHF21A	rs79686965	intron_variant	2.00E-11
GCST90179150	11	64322950	PRDX5 - CCDC88B	rs7947143	intergenic_variant	4.00E-14
GCST009004	11	64322950	PRDX5 - CCDC88B	rs7947143	intergenic_variant	8.00E-14
GCST90255621	11	64322950	PRDX5 - CCDC88B	rs7947143	intergenic_variant	1.00E-13
GCST90179150	11	47425795	PSMC3	rs10838709	non_coding_transcript_exon_	5.00E-16

GCST90018727	11	47419784	PSMC3	rs7120413	intron_variant	4.00E-11
GCST90179150	11	117102531	SIK3	rs76942203	intron_variant	1.00E-11
GCST009004	11	117102531	SIK3	rs76942203	intron_variant	9.00E-11
GCST90255621	11	64589600	SLC22A12	rs505802	intergenic_variant	6.00E-23
GCST90179150	11	64598324	SLC22A12	rs893006	intron_variant	4.00E-16
GCST002829	11	64593233	SLC22A12	rs10897518	intron_variant	9.00E-14
GCST009871	11	64593151	SLC22A12	rs576076	intron_variant	2.00E-12
GCST90255621	11	47385888	SLC39A13	rs11606287	intron_variant	7.00E-18
GCST009871	11	47387500	SLC39A13	rs11500477	intron_variant	2.00E-13
GCST90255621	11	130925803	SNX19	rs7944782	intergenic_variant	4.00E-19
GCST009004	11	130925803	SNX19	rs7944782	intergenic_variant	4.00E-17
GCST90179150	11	130925803	SNX19	rs7944782	intergenic_variant	1.00E-16
GCST007039	11	130925803	SNX19	rs7944782	intergenic_variant	1.00E-15
GCST009001	11	130926353	SNX19	rs7933085	intergenic_variant	2.00E-15
GCST009871	11	130925803	SNX19	rs7944782	intergenic_variant	8.00E-15
GCST009003	11	130886281	SNX19	rs4459316	intron_variant	3.00E-11
GCST009004	11	133842787	SPATA19	rs2187449	intron_variant	4.00E-14
GCST009003	11	133842787	SPATA19	rs2187449	intron_variant	4.00E-14
GCST009871	11	133842787	SPATA19	rs2187449	intron_variant	3.00E-11
GCST005953	11	8478498	STK33	rs10840060	intron_variant	4.00E-11
GCST004904	11	8382954	STK33	rs16937956	regulatory_region_variant	5.00E-11
GCST005950	11	8478498	STK33	rs10840060	intron_variant	6.00E-11
GCST005951	11	8478498	STK33	rs10840060	intron_variant	8.00E-11
GCST90255621	11	8617653	TRIM66	rs4929923	3_prime_UTR_variant	9.00E-32
GCST009004	11	8652392	TRIM66	rs4256980	intron_variant	9.00E-29
GCST009003	11	8617653	TRIM66	rs4929923	3_prime_UTR_variant	3.00E-28
GCST009001	11	8617653	TRIM66	rs4929923	3_prime_UTR_variant	3.00E-28
GCST90179150	11	8657469	TRIM66	rs2316901	intron_variant	2.00E-26
GCST90018947	11	8614558	TRIM66	rs10769931	3_prime_UTR_variant	7.00E-19
GCST009871	11	8617653	TRIM66	rs4929923	3_prime_UTR_variant	9.00E-19
GCST007039	11	8617653	TRIM66	rs4929923	3_prime_UTR_variant	2.00E-18
GCST004904	11	8646133	TRIM66	rs10840099	intron_variant	1.00E-17
GCST008129	11	8640969	TRIM66	rs11042023	missense_variant	1.00E-16
GCST005951	11	8647890	TRIM66	rs10840100	intron_variant	2.00E-15
GCST009871	11	8659338	TRIM66	rs11042026	intron_variant	5.00E-13
GCST006368	11	8647890	TRIM66	rs10840100	intron_variant	5.00E-12
GCST002783	11	8652392	TRIM66	rs4256980	intron_variant	8.00E-12
GCST002783	11	8652392	TRIM66	rs4256980	intron_variant	3.00E-11
GCST004557	11	8617653	TRIM66	rs4929923	3_prime_UTR_variant	9.00E-11
GCST90179150	11	43510651	TTC17 - CTBP2P6	rs4755713	intergenic_variant	6.00E-13
GCST007039	11	43510651	TTC17 - CTBP2P6	rs4755713	intergenic_variant	2.00E-12
GCST90255621	11	43510651	TTC17 - CTBP2P6	rs4755713	intergenic_variant	7.00E-12
GCST009871	11	118525616	TTC36	rs1064939	3_prime_UTR_variant	1.00E-12
GCST007039	11	118525616	TTC36	rs1064939	3_prime_UTR_variant	2.00E-12
GCST90255621	11	118525616	TTC36	rs1064939	3_prime_UTR_variant	4.00E-11
GCST90179150	11	118528385	TTC36	rs148118632	intron_variant	8.00E-11
GCST009004	11	122664625	UBASH3B	rs10892873	intron_variant	2.00E-11
GCST009003	11	122664625	UBASH3B	rs10892873	intron_variant	2.00E-11
GCST90255621	11	122655793	UBASH3B	rs7127978	5_prime_UTR_variant	2.00E-11
GCST90179150	11	122647817	UBASH3B	rs6589939	regulatory_region_variant	5.00E-11
GCST90255621	11	119081463	VPS11	rs15818	missense_variant	6.00E-17

GCST009004	11	119073965	VPS11	rs3825061	intron_variant	6.00E-16
GCST90179150	11	119073965	VPS11	rs3825061	intron_variant	2.00E-15
GCST007039	11	119070886	VPS11	rs7925100	intron_variant	3.00E-12
GCST008129	11	119081463	VPS11	rs15818	missense_variant	9.00E-12
GCST009871	11	119070886	VPS11	rs7925100	intron_variant	1.00E-11
GCST004046	11	116778201	ZPR1	rs964184	3_prime_UTR_variant	3.00E-116
GCST004045	11	116778201	ZPR1	rs964184	3_prime_UTR_variant	2.00E-108
GCST002461	12	111803962	AKT3	rs671	missense_variant	3.00E-11
GCST009004	12	99166405	ANKS1A	rs651548	intron_variant	2.00E-16
GCST009003	12	99246152	ANKS1B	rs923724	intron_variant	2.00E-15
GCST90179150	12	99166405	ANKS1B	rs651548	intron_variant	2.00E-15
GCST006368	12	99179648	ANKS1B	rs2372716	intron_variant	3.00E-15
GCST90255621	12	99191065	ANKS1B	rs2263049	intron_variant	1.00E-14
GCST007039	12	99160037	ANKS1B	rs660879	intron_variant	7.00E-13
GCST009871	12	99160037	ANKS1B	rs660879	intron_variant	1.00E-12
GCST90018947	12	99068704	ANKS1B	rs10554812	intron_variant	6.00E-11
GCST009004	12	108000068	ASCC3	rs12316047	intergenic_variant	1.00E-17
GCST90179150	12	108000068	ASCL4	rs12316047	intergenic_variant	4.00E-17
GCST90255621	12	108000068	ASCL4	rs12316047	intergenic_variant	2.00E-16
GCST009003	12	107939217	ASCL4	rs1895941	intergenic_variant	2.00E-15
GCST009871	12	108019073	ASCL4	rs74710200	intergenic_variant	4.00E-14
GCST007039	12	108042619	ASCL4	rs10861861	intergenic_variant	7.00E-14
GCST90018947	12	107900763	ASCL4	rs35046541	intron_variant	2.00E-11
GCST90255621	12	33226505	ASMT	rs10772055	intergenic_variant	7.00E-20
GCST90018947	12	33228959	ASS1P14 - SYT10	rs28851196	intergenic_variant	4.00E-11
GCST90255621	12	111634620	ATP2A1	rs11065987	intergenic_variant	6.00E-23
GCST011331	12	111472415	ATXN2	rs10774625	intron_variant	4.00E-22
GCST011336	12	111472415	ATXN2	rs10774625	intron_variant	1.00E-13
GCST011330	12	111472415	ATXN2	rs10774625	intron_variant	2.00E-13
GCST005951	12	111634620	ATXN2	rs11065987	intergenic_variant	2.00E-11
GCST90255621	12	49853685	BBS4	rs7138803	intergenic_variant	4.00E-90
GCST009004	12	49853685	BCDIN3D	rs7138803	intergenic_variant	3.00E-71
GCST009003	12	49853685	BCDIN3D	rs7138803	intergenic_variant	3.00E-71
GCST90179150	12	49853685	BCDIN3D	rs7138803	intergenic_variant	1.00E-65
GCST005951	12	49853685	BCDIN3D	rs7138803	intergenic_variant	2.00E-31
GCST006368	12	49853685	BCDIN3D	rs7138803	intergenic_variant	6.00E-27
GCST002783	12	49853685	BCDIN3D	rs7138803	intergenic_variant	5.00E-26
GCST002783	12	49853685	BCDIN3D	rs7138803	intergenic_variant	8.00E-24
GCST004904	12	49853685	BCDIN3D	rs7138803	intergenic_variant	2.00E-21
GCST90002409	12	49853685	BCDIN3D	rs7138803	intergenic_variant	7.00E-20
GCST003177	12	49853685	BCDIN3D	rs7138803	intergenic_variant	5.00E-18
GCST002783	12	49853685	BCDIN3D	rs7138803	intergenic_variant	2.00E-17
GCST000830	12	49853685	BCDIN3D	rs7138803	intergenic_variant	2.00E-17
GCST004495	12	49853685	BCDIN3D	rs7138803	intergenic_variant	4.00E-16
GCST004557	12	49853685	BCDIN3D	rs7138803	intergenic_variant	5.00E-16
GCST004558	12	49853685	BCDIN3D	rs7138803	intergenic_variant	1.00E-15
GCST006802	12	49853685	BCDIN3D	rs7138803	intergenic_variant	2.00E-15
GCST004497	12	49853685	BCDIN3D	rs7138803	intergenic_variant	3.00E-15
GCST004557	12	49853685	BCDIN3D	rs7138803	intergenic_variant	9.00E-15
GCST004558	12	49853685	BCDIN3D	rs7138803	intergenic_variant	6.00E-14
GCST004557	12	49853685	BCDIN3D	rs7138803	intergenic_variant	6.00E-12

GCST004557	12	49853685	BCDIN3D	rs7138803	intergenic_variant	2.00E-11
GCST004558	12	49853685	BCDIN3D	rs7138803	intergenic_variant	2.00E-11
GCST004559	12	49853685	BCDIN3D	rs7138803	intergenic_variant	2.00E-11
GCST004558	12	49853685	BCDIN3D	rs7138803	intergenic_variant	4.00E-11
GCST004499	12	49853685	BCDIN3D	rs7138803	intergenic_variant	4.00E-11
GCST002783	12	49853685	BCDIN3D	rs7138803	intergenic_variant	6.00E-11
GCST90255621	12	122056903	BCL2	rs11835818	intron_variant	2.00E-17
GCST009871	12	122047483	BCL7A	rs144713920	intron_variant	1.00E-13
GCST90255621	12	103264318	BTBD7	rs4764949	intron_variant	5.00E-32
GCST009004	12	103312976	C12orf42	rs6539064	intron_variant	1.00E-23
GCST009003	12	103312976	C12orf42	rs6539064	intron_variant	1.00E-23
GCST009001	12	103312976	C12orf42	rs6539064	intron_variant	1.00E-23
GCST90179150	12	103330312	C12orf42	rs7138383	intron_variant	3.00E-23
GCST007039	12	103264318	C12orf42	rs4764949	intron_variant	7.00E-21
GCST009871	12	103312976	C12orf42	rs6539064	intron_variant	3.00E-19
GCST90018947	12	103312976	C12orf42	rs6539064	intron_variant	5.00E-14
GCST009871	12	103247174	C12orf42	rs1520194	intron_variant	4.00E-13
GCST007039	12	2050390	CACNA1C	rs2108635	intron_variant	3.00E-13
GCST90018947	12	2050390	CACNA1C	rs2108635	intron_variant	2.00E-12
GCST90255621	12	2050390	CACNA1C	rs2108635	intron_variant	1.00E-11
GCST90179150	12	19055014	CAPZA3	rs7976757	intergenic_variant	1.00E-11
GCST009004	12	19055014	CAPZA3	rs7976757	intergenic_variant	3.00E-11
GCST009003	12	19055014	CAPZA3	rs7976757	intergenic_variant	3.00E-11
GCST90255621	12	54273501	CBX5, SCAT2	rs4759075	non_coding_transcript_exon_	1.00E-13
GCST90179150	12	54276271	CBX5, SCAT2	rs1920046	intron_variant	5.00E-13
GCST007039	12	54277096	CBX5, SCAT2	rs4237798	non_coding_transcript_exon_	1.00E-11
GCST009004	12	82072018	CCDC59	rs11115176	intergenic_variant	7.00E-12
GCST90179150	12	82072018	CCDC59	rs11115176	intergenic_variant	1.00E-11
GCST90255621	12	82072018	CCDC59	rs11115176	intergenic_variant	6.00E-11
GCST90179150	12	123924955	CCDC92, DNAH10	rs7133378	intron_variant	4.00E-15
GCST011336	12	123924955	CCDC92, DNAH10	rs7133378	intron_variant	6.00E-15
GCST90255621	12	123924955	CCDC92, DNAH10	rs7133378	intron_variant	5.00E-14
GCST009004	12	123924955	CCDC92, DNAH10	rs7133378	intron_variant	3.00E-13
GCST009003	12	123924955	CCDC92, DNAH10	rs7133378	intron_variant	3.00E-13
GCST011334	12	123924955	CCDC92, DNAH10	rs7133378	intron_variant	3.00E-12
GCST90179150	12	122297350	CLIP1	rs11057405	intron_variant	2.00E-28
GCST90271772	12	122458829	CLIP1	rs10846921	intergenic_variant	2.00E-28
GCST011336	12	122297350	CLIP1	rs11057405	intron_variant	1.00E-18
GCST009871	12	122302757	CLIP1	rs12578953	intron_variant	3.00E-11
GCST90255621	12	39036246	CPNE8-AS1 - LINC02406	rs11170468	intergenic_variant	3.00E-15
GCST006368	12	39036246	CPNE8-AS1 - LINC02406	rs11170468	intergenic_variant	8.00E-13
GCST009004	12	39036246	CPNE8-AS1 - LINC02406	rs11170468	intergenic_variant	1.00E-11
GCST90179150	12	39036246	CPNE8-AS1 - LINC02406	rs11170468	intergenic_variant	1.00E-11
GCST90271769	12	69280815	CPSF6	rs650198	intergenic_variant	6.00E-14
GCST90271767	12	69280815	CPSF6	rs650198	intergenic_variant	3.00E-13
GCST009004	12	69280815	CPSF6	rs650198	intergenic_variant	6.00E-13
GCST90179150	12	69280815	CPSF6	rs650198	intergenic_variant	3.00E-12
GCST90255621	12	69248535	CPSF6	rs10878946	intron_variant	8.00E-11
GCST90255621	12	89351700	DUSP6	rs2279574	missense_variant	1.00E-17
GCST90179150	12	89351700	DUSP6	rs2279574	missense_variant	4.00E-17
GCST009003	12	89378126	DUSP6	rs704061	intron_variant	8.00E-17

GCST009004	12	89378126	DUSP6	rs704061	intron_variant	8.00E-17
GCST009871	12	89378126	DUSP6	rs704061	intron_variant	2.00E-16
GCST007039	12	89378126	DUSP6	rs704061	intron_variant	2.00E-15
GCST90255621	12	89364269	DUSP6	rs1689437	intron_variant	5.00E-14
GCST009001	12	89364269	DUSP6	rs1689437	intron_variant	5.00E-13
GCST90179150	12	89364269	DUSP6	rs1689437	intron_variant	2.00E-12
GCST90018947	12	89383076	DUSP6	rs397944660	intron_variant	2.00E-11
GCST009004	12	60570327	DUX4L52	rs7975187	intergenic_variant	4.00E-11
GCST009003	12	60570327	DUX4L52	rs7975187	intergenic_variant	4.00E-11
GCST90255621	12	17059947	EEF1A1P11	rs10744146	intergenic_variant	3.00E-14
GCST90018947	12	17043898	EEF1A1P11	rs7313220	intergenic_variant	5.00E-12
GCST009004	12	17059947	EEF1A1P11	rs10744146	intergenic_variant	3.00E-11
GCST90179150	12	17059947	EEF1A1P11	rs10744146	intergenic_variant	6.00E-11
GCST009868	12	56080696	ERBB3	rs4759229	intron_variant	2.00E-23
GCST009866	12	56080696	ERBB3	rs4759229	intron_variant	2.00E-21
GCST009871	12	56104119	ERBB3	rs3759094	intron_variant	9.00E-16
GCST90179150	12	56100038	ERBB3	rs2292238	intron_variant	2.00E-15
GCST009004	12	56098096	ERBB3	rs10783779	non_coding_transcript_exon_	5.00E-15
GCST009003	12	56098096	ERBB3	rs10783779	non_coding_transcript_exon_	5.00E-15
GCST009001	12	49869365	FAIM2	rs7132908	3_prime_UTR_variant	5.00E-64
GCST007039	12	49869365	FAIM2	rs7132908	3_prime_UTR_variant	5.00E-50
GCST009871	12	49869365	FAIM2	rs7132908	3_prime_UTR_variant	3.00E-49
GCST90018947	12	49869365	FAIM2	rs7132908	3_prime_UTR_variant	1.00E-39
GCST009121	12	49869365	FAIM2	rs7132908	3_prime_UTR_variant	4.00E-32
GCST007241	12	49869365	FAIM2	rs7132908	3_prime_UTR_variant	2.00E-31
GCST003177	12	49869365	FAIM2	rs7132908	3_prime_UTR_variant	2.00E-18
GCST007240	12	49869365	FAIM2	rs7132908	3_prime_UTR_variant	2.00E-16
GCST009871	12	49877262	FAIM2	rs1248264	intron_variant	7.00E-16
GCST009871	12	49897952	FAIM2	rs297937	intron_variant	1.00E-14
GCST90255621	12	49883643	FAIM2	rs417644	intron_variant	5.00E-14
GCST008158	12	49876581	FAIM2	rs12146733	intron_variant	2.00E-12
GCST009107	12	49869365	FAIM2	rs7132908	3_prime_UTR_variant	4.00E-11
GCST90255621	12	49896945	FAIM2	rs297935	intron_variant	4.00E-11
GCST90255621	12	14260997	GNAI2P1	rs12422552	regulatory_region_variant	9.00E-12
GCST009004	12	14260997	GNAI2P1	rs12422552	regulatory_region_variant	2.00E-11
GCST009004	12	110465575	GPN3	rs6606686	intron_variant	4.00E-19
GCST009003	12	110465575	GPN3	rs6606686	intron_variant	4.00E-19
GCST90255621	12	110465575	GPN3	rs6606686	intron_variant	5.00E-18
GCST009871	12	110465575	GPN3	rs6606686	intron_variant	4.00E-12
GCST007039	12	110465575	GPN3	rs6606686	intron_variant	4.00E-12
GCST90179150	12	122705247	HCAR2 - HCAR3	rs7133768	intron_variant	5.00E-15
GCST009871	12	122708052	HCAR2 - HCAR3	rs11059476	intron_variant	8.00E-13
GCST90179150	12	112172910	HECTD4	rs11066188	intron_variant	5.00E-13
GCST90054786	12	112189546	HECTD4	rs144504271	intron_variant	2.00E-12
GCST005951	12	112153882	HECTD4	rs17630235	intergenic_variant	4.00E-12
GCST008128	12	122860962	HIP1R	rs34149579	missense_variant	8.00E-15
GCST008129	12	122860962	HIP1R	rs34149579	missense_variant	2.00E-14
GCST90179150	12	122844554	HIP1R	rs11060344	intron_variant	6.00E-11
GCST90054786	12	102442081	IGF1	rs5742653	intron_variant	2.00E-11
GCST011336	12	57450266	INHBC	rs3741414	3_prime_UTR_variant	2.00E-13
GCST011334	12	57450266	INHBC	rs3741414	3_prime_UTR_variant	1.00E-12

GCST007039	12	122539929	KNTC1	rs147730268	intron_variant	2.00E-26
GCST009871	12	122539929	KNTC1	rs147730268	intron_variant	1.00E-25
GCST009003	12	122615850	KNTC1	rs6489156	intron_variant	2.00E-24
GCST90018947	12	122539929	KNTC1	rs147730268	intron_variant	2.00E-17
GCST009871	12	122640231	KNTC1	rs10773394	regulatory_region_variant	2.00E-15
GCST90179150	12	122625818	KNTC1	rs6489158	intron_variant	1.00E-14
GCST90255621	12	122628870	KNTC1	rs10773378	intergenic_variant	1.00E-11
GCST90255621	12	90234453	LINC02399 - LINC02392	rs2579106	regulatory_region_variant	2.00E-14
GCST90179150	12	90227881	LINC02399 - LINC02392	rs2731251	intergenic_variant	2.00E-11
GCST006368	12	67811824	LINC02421 - LINC01479	rs1819844	TF_binding_site_variant	2.00E-14
GCST90255621	12	67811824	LINC02421 - LINC01479	rs1819844	TF_binding_site_variant	4.00E-13
GCST009004	12	67811824	LINC02421 - LINC01479	rs1819844	TF_binding_site_variant	2.00E-11
GCST90179150	12	67811824	LINC02421 - LINC01479	rs1819844	TF_binding_site_variant	3.00E-11
GCST90255621	12	90844143	LINC02822	rs11105839	intron_variant	8.00E-12
GCST009004	12	90844143	LINC02822	rs11105839	intron_variant	1.00E-11
GCST009871	12	69287321	LYZ	rs317656	intergenic_variant	8.00E-13
GCST007039	12	69287321	LYZ	rs317656	intergenic_variant	2.00E-12
GCST90271771	12	122128993	MLXIP	rs6489244	5_prime_UTR_variant	2.00E-18
GCST90179150	12	122128993	MLXIP	rs6489244	5_prime_UTR_variant	3.00E-14
GCST009871	12	122117758	MLXIP	rs28642975	intron_variant	2.00E-13
GCST90255621	12	57217502	NXPH4	rs113397893	intron_variant	8.00E-17
GCST007039	12	57217502	NXPH4	rs113397893	intron_variant	5.00E-12
GCST009871	12	57217502	NXPH4	rs113397893	intron_variant	1.00E-11
GCST90255621	12	121232923	P2RX4	rs11608486	non_coding_transcript_exon_	3.00E-12
GCST009004	12	121233458	P2RX4	rs7961979	non_coding_transcript_exon_	7.00E-12
GCST009003	12	121233458	P2RX4	rs7961979	non_coding_transcript_exon_	7.00E-12
GCST90179150	12	121233458	P2RX4	rs7961979	non_coding_transcript_exon_	6.00E-11
GCST007039	12	56114625	PA2G4	rs4759228	intergenic_variant	1.00E-16
GCST90018947	12	56114625	PA2G4	rs4759228	intergenic_variant	2.00E-15
GCST90255621	12	41437372	PDZRN4	rs2730829	intron_variant	1.00E-34
GCST009004	12	41554394	PDZRN4	rs11181001	intron_variant	1.00E-16
GCST009001	12	41554394	PDZRN4	rs11181001	intron_variant	1.00E-16
GCST009003	12	41554394	PDZRN4	rs11181001	intron_variant	1.00E-16
GCST90179150	12	41554394	PDZRN4	rs11181001	intron_variant	1.00E-16
GCST007039	12	41494138	PDZRN4	rs1458156	intron_variant	3.00E-15
GCST009871	12	41452470	PDZRN4	rs1841025	intron_variant	4.00E-14
GCST90018947	12	41444433	PDZRN4	rs2733289	intron_variant	2.00E-11
GCST007039	12	19250055	PLEKHA5	rs11044463	intron_variant	2.00E-11
GCST90255621	12	19161981	PLEKHA5	rs954976	intron_variant	7.00E-11
GCST009871	12	19134482	PLEKHA5	rs11044430	intron_variant	8.00E-11
GCST90255621	12	49005349	PRKAG1	rs1126930	missense_variant	3.00E-23
GCST008129	12	49005349	PRKAG1	rs1126930	missense_variant	4.00E-12
GCST90255621	12	71785666	RAB21	rs61754230	missense_variant	9.00E-19
GCST007039	12	71785666	RAB21	rs61754230	missense_variant	7.00E-11
GCST009871	12	924017	RAD52	rs57307148	intron_variant	2.00E-25
GCST90018947	12	924017	RAD52	rs57307148	intron_variant	9.00E-17
GCST90255621	12	47749532	RAPGEF3	rs145878042	missense_variant	9.00E-16
GCST008129	12	47749532	RAPGEF3	rs145878042	missense_variant	2.00E-15
GCST009871	12	114096867	RBM19	rs61930810	intron_variant	2.00E-11
GCST007039	12	114008651	RBM19	rs76406609	intergenic_variant	2.00E-11
GCST90255621	12	114096867	RBM19	rs61930810	intron_variant	3.00E-11

GCST90179150	12	114008651	RBM19	rs76406609	intergenic_variant	5.00E-11
GCST90255621	12	124022084	RFLNA	rs10773049	intron_variant	2.00E-16
GCST011336	12	124020897	RFLNA	rs863750	intron_variant	5.00E-16
GCST90179150	12	124022084	RFLNA	rs10773049	intron_variant	2.00E-12
GCST90255621	12	89819293	RNU6-148P - BRWD1P2	rs12427047	intron_variant	4.00E-14
GCST009871	12	89819293	RNU6-148P - BRWD1P2	rs12427047	intron_variant	2.00E-13
GCST90179150	12	89821287	RNU6-148P - BRWD1P2	rs11105423	intron_variant	2.00E-13
GCST90018727	12	31288245	SINHCAF	rs80234489	intron_variant	4.00E-13
GCST90018947	12	31313679	SINHCAF	rs147538848	intron_variant	6.00E-13
GCST004904	12	31288245	SINHCAF	rs80234489	intron_variant	1.00E-11
GCST90255621	12	23907141	SOX5	rs10842240	intron_variant	1.00E-17
GCST009004	12	23855501	SOX5	rs11047132	intron_variant	2.00E-13
GCST009003	12	23849191	SOX5	rs16926778	intron_variant	2.00E-13
GCST007039	12	23907141	SOX5	rs10842240	intron_variant	2.00E-13
GCST90179150	12	23848971	SOX5	rs16926776	intron_variant	2.00E-12
GCST009871	12	23855501	SOX5	rs11047132	intron_variant	1.00E-11
GCST90018947	12	23905201	SOX5	rs10771041	intron_variant	3.00E-11
GCST90255621	12	830314	WNK1	rs11611246	intron_variant	3.00E-39
GCST009004	12	830314	WNK1	rs11611246	intron_variant	2.00E-28
GCST009001	12	830314	WNK1	rs11611246	intron_variant	2.00E-28
GCST009003	12	830314	WNK1	rs11611246	intron_variant	2.00E-28
GCST90179150	12	830314	WNK1	rs11611246	intron_variant	2.00E-28
GCST007039	12	882140	WNK1	rs55726687	intron_variant	3.00E-26
GCST008129	12	889199	WNK1	rs12828016	missense_variant	5.00E-15
GCST009871	12	886440	WNK1	rs10744727	intron_variant	2.00E-13
GCST90255621	12	841915	WNK1	rs10849568	intron_variant	3.00E-13
GCST006368	12	830314	WNK1	rs11611246	intron_variant	2.00E-12
GCST005951	12	830314	WNK1	rs11611246	intron_variant	1.00E-11
GCST90179150	12	841915	WNK1	rs10849568	intron_variant	5.00E-11
GCST90271770	12	108236003	WSCD2	rs1426371	intron_variant	2.00E-14
GCST90271769	12	108224853	WSCD2	rs3764002	missense_variant	6.00E-12
GCST90271770	12	108179913	WSCD2	rs35739367	intron_variant	8.00E-11
GCST90255621	12	122479003	ZCCHC8	rs12369179	intron_variant	1.00E-39
GCST009004	12	122479003	ZCCHC8	rs12369179	intron_variant	2.00E-28
GCST009001	12	122479003	ZCCHC8	rs12369179	intron_variant	2.00E-28
GCST90179150	13	79006784	CCT5P2 - NIPA2P5	rs1441264	intergenic_variant	8.00E-24
GCST007039	13	79006784	CCT5P2 - NIPA2P5	rs1441264	intergenic_variant	3.00E-21
GCST009871	13	79006784	CCT5P2 - NIPA2P5	rs1441264	intergenic_variant	2.00E-20
GCST90255621	13	78989614	CCT5P2 - NIPA2P5	rs9530843	intergenic_variant	1.00E-14
GCST90267268	13	79006784	CCT5P2 - NIPA2P5	rs1441264	intergenic_variant	4.00E-13
GCST006368	13	79006784	CCT5P2 - NIPA2P5	rs1441264	intergenic_variant	2.00E-12
GCST90018947	13	79013706	CCT5P2 - NIPA2P5	rs1576655	intergenic_variant	3.00E-12
GCST90255621	13	58601593	DNAJA1P1	rs9317002	intergenic_variant	4.00E-31
GCST90255621	13	58820185	DNAJA1P1	rs6561987	intergenic_variant	1.00E-21
GCST007039	13	58691919	DNAJA1P1	rs4055791	intergenic_variant	1.00E-19
GCST90179150	13	58604124	DNAJA1P1	rs9538141	intergenic_variant	2.00E-19
GCST009004	13	58601593	DNAJA1P1	rs9317002	intergenic_variant	5.00E-19
GCST009871	13	58601593	DNAJA1P1	rs9317002	intergenic_variant	6.00E-19
GCST009001	13	58623402	DNAJA1P1	rs4432163	intergenic_variant	6.00E-18
GCST90179150	13	58852765	DNAJA1P1	rs7328849	intergenic_variant	3.00E-16
GCST009871	13	58824900	DNAJA1P1	rs722122	intergenic_variant	3.00E-14

GCST009871	13	58706571	DNAJA1P1	rs11839227	regulatory_region_variant	8.00E-13
GCST90255621	13	58598516	DNAJA1P1	rs9538136	regulatory_region_variant	5.00E-12
GCST006368	13	58877855	DNAJA1P1	rs2321882	intergenic_variant	8.00E-12
GCST90255621	13	28050157	FLT3	rs1933437	missense_variant	9.00E-25
GCST008129	13	28050157	FLT3	rs1933437	missense_variant	1.00E-18
GCST90179150	13	28050157	FLT3	rs1933437	missense_variant	7.00E-17
GCST009004	13	28043571	FLT3	rs7318817	intron_variant	4.00E-16
GCST009003	13	28043571	FLT3	rs7318817	intron_variant	4.00E-16
GCST009001	13	28043571	FLT3	rs7318817	intron_variant	4.00E-16
GCST009871	13	28038749	FLT3	rs2504235	intron_variant	3.00E-12
GCST007039	13	28066426	FLT3	rs9513018	intron_variant	4.00E-12
GCST90255621	13	27434894	GTF3A	rs7323	missense_variant	2.00E-29
GCST009001	13	27424463	GTF3A	rs11619722	regulatory_region_variant	2.00E-15
GCST90255621	13	30459095	HMGB1	rs1045411	3_prime_UTR_variant	4.00E-17
GCST009004	13	30459095	HMGB1	rs1045411	3_prime_UTR_variant	7.00E-14
GCST007039	13	30463766	HMGB1	rs2249825	5_prime_UTR_variant	1.00E-13
GCST009871	13	30463766	HMGB1	rs2249825	5_prime_UTR_variant	2.00E-13
GCST90179150	13	30459095	HMGB1	rs1045411	3_prime_UTR_variant	2.00E-12
GCST90255621	13	55891463	HNF4GP1	rs9527455	intergenic_variant	3.00E-12
GCST90255621	13	56198562	HNF4GP1	rs1512651	intergenic_variant	1.00E-11
GCST90255621	13	96269937	HS6ST3	rs1927790	intron_variant	9.00E-25
GCST90179150	13	96269937	HS6ST3	rs1927790	intron_variant	4.00E-18
GCST009004	13	96269937	HS6ST3	rs1927790	intron_variant	2.00E-17
GCST009001	13	96368451	HS6ST3	rs912690	intron_variant	2.00E-17
GCST009003	13	96269937	HS6ST3	rs1927790	intron_variant	2.00E-17
GCST009871	13	96366836	HS6ST3	rs7996639	intron_variant	2.00E-14
GCST007039	13	96366836	HS6ST3	rs7996639	intron_variant	3.00E-14
GCST90018947	13	96330287	HS6ST3	rs7990098	intron_variant	2.00E-13
GCST006368	13	96396750	HS6ST3	rs9634489	intron_variant	7.00E-11
GCST90255621	13	54254826	LINC00458	rs7995015	intron_variant	4.00E-12
GCST009871	13	54117349	LINC00458	rs6561766	non_coding_transcript_exon_	2.00E-11
GCST007039	13	54254826	LINC00458	rs7995015	intron_variant	2.00E-11
GCST90179150	13	54254826	LINC00458	rs7995015	intron_variant	5.00E-11
GCST009004	13	54117349	LINC00458	rs6561766	non_coding_transcript_exon_	7.00E-11
GCST009004	13	27461925	MTIF3	rs1967772	intergenic_variant	9.00E-20
GCST009003	13	27461925	MTIF3	rs1967772	intergenic_variant	9.00E-20
GCST90179150	13	27461925	MTIF3	rs1967772	intergenic_variant	4.00E-19
GCST007039	13	27437826	MTIF3	rs1218822	intron_variant	4.00E-17
GCST009871	13	27461925	MTIF3	rs1967772	intergenic_variant	5.00E-16
GCST90255621	13	27443133	MTIF3	rs9579083	intron_variant	1.00E-15
GCST90018947	13	27448777	MTIF3	rs4771123	intron_variant	4.00E-15
GCST005951	13	27443645	MTIF3	rs12016871	intron_variant	1.00E-13
GCST006368	13	27443645	MTIF3	rs9581854	intron_variant	3.00E-11
GCST90179150	13	27473132	MTIF3	rs1006353	regulatory_region_variant	5.00E-11
GCST002783	13	27443645	MTIF3	rs12016871	intron_variant	9.00E-11
GCST90255621	13	32573411	N4BP2L2	rs7332115	intergenic_variant	3.00E-22
GCST009871	13	32569111	N4BP2L2	rs12323184	intergenic_variant	6.00E-17
GCST90018947	13	32500537	N4BP2L2	rs34916533	intron_variant	4.00E-15
GCST90255621	13	53528071	OLFM4	rs12429545	intron_variant	4.00E-45
GCST90179150	13	53528071	OLFM4	rs12429545	intron_variant	3.00E-38
GCST009004	13	53528071	OLFM4	rs12429545	intron_variant	1.00E-37

GCST009001	13	53528071	OLFM4	rs12429545	intron_variant	1.00E-37
GCST009003	13	53528071	OLFM4	rs12429545	intron_variant	1.00E-37
GCST90018947	13	53530833	OLFM4	rs4477562	intron_variant	3.00E-27
GCST004904	13	53528071	OLFM4	rs12429545	intron_variant	1.00E-24
GCST007039	13	53530833	OLFM4	rs4477562	intron_variant	1.00E-24
GCST009871	13	53530833	OLFM4	rs4477562	intron_variant	4.00E-24
GCST006368	13	53528071	OLFM4	rs12429545	intron_variant	5.00E-18
GCST005951	13	53528071	OLFM4	rs12429545	intron_variant	7.00E-17
GCST004904	13	53533217	OLFM4	rs9568867	intron_variant	1.00E-14
GCST003177	13	53528071	OLFM4	rs12429545	intron_variant	2.00E-14
GCST90018727	13	53533448	OLFM4	rs9568868	intron_variant	2.00E-14
GCST009871	13	53482418	OLFM4	rs9596810	intron_variant	7.00E-14
GCST002783	13	53528071	OLFM4	rs12429545	intron_variant	3.00E-13
GCST009871	13	53524263	OLFM4	rs1927855	intron_variant	4.00E-13
GCST90002409	13	53530833	OLFM4	rs4477562	intron_variant	8.00E-13
GCST002783	13	53528071	OLFM4	rs12429545	intron_variant	1.00E-12
GCST90179150	13	53482418	OLFM4	rs9596810	intron_variant	5.00E-12
GCST90255621	13	53077715	OLFM4	rs1112613	intergenic_variant	7.00E-11
GCST009871	13	57678667	PCDH17	rs7319102	intron_variant	2.00E-11
GCST90179150	13	57828345	PCDH17	rs9527706	intergenic_variant	2.00E-11
GCST90255621	13	66898581	PCDH9	rs9571687	intron_variant	3.00E-15
GCST009004	13	66898581	PCDH9	rs9571687	intron_variant	2.00E-11
GCST009003	13	66898581	PCDH9	rs9571687	intron_variant	2.00E-11
GCST006368	13	32610151	PDS5B	rs9595908	intron_variant	5.00E-23
GCST009004	13	32610151	PDS5B	rs9595908	intron_variant	4.00E-20
GCST009003	13	32610151	PDS5B	rs9595908	intron_variant	4.00E-20
GCST009001	13	32601656	PDS5B	rs9595893	intron_variant	4.00E-20
GCST90179150	13	32610151	PDS5B	rs9595908	intron_variant	4.00E-19
GCST007039	13	32807583	PDS5B	rs56399737	intergenic_variant	1.00E-17
GCST006368	13	58096013	RNA5SP30 - LINC02338	rs9563576	intergenic_variant	6.00E-28
GCST90255621	13	58057391	RNA5SP30 - LINC02338	rs9569808	intergenic_variant	2.00E-25
GCST009003	13	58056517	RNA5SP30 - LINC02338	rs12431244	intergenic_variant	2.00E-22
GCST90179150	13	58057391	RNA5SP30 - LINC02338	rs9569808	intergenic_variant	2.00E-21
GCST009871	13	58052054	RNA5SP30 - LINC02338	rs11148421	regulatory_region_variant	3.00E-20
GCST90018947	13	58049649	RNA5SP30 - LINC02338	rs12866691	intergenic_variant	3.00E-17
GCST009004	13	85920682	SLITRK6 - MOB1AP1	rs77432547	intergenic_variant	1.00E-15
GCST009003	13	85920682	SLITRK6 - MOB1AP1	rs77432547	intergenic_variant	1.00E-15
GCST90179150	13	85908431	SLITRK6 - MOB1AP1	rs72632780	intergenic_variant	2.00E-15
GCST90255621	13	85966654	SLITRK6 - MOB1AP1	rs7331467	intergenic_variant	2.00E-14
GCST007039	13	85909890	SLITRK6 - MOB1AP1	rs61969510	intergenic_variant	3.00E-13
GCST009871	13	85909890	SLITRK6 - MOB1AP1	rs61969510	intergenic_variant	4.00E-13
GCST90018947	13	85902937	SLITRK6 - MOB1AP1	rs116394958	intergenic_variant	6.00E-11
GCST90255621	13	65631572	STARP1	rs9540493	intergenic_variant	3.00E-21
GCST009004	13	65631572	STARP1	rs9540493	intergenic_variant	8.00E-15
GCST009003	13	65631572	STARP1	rs9540493	intergenic_variant	8.00E-15
GCST90179150	13	65631572	STARP1	rs9540493	intergenic_variant	6.00E-14
GCST90018947	13	65233151	STARP1	rs778433	intergenic_variant	1.00E-11
GCST009004	13	98464130	STK24	rs17574378	intron_variant	7.00E-14
GCST90179150	13	98464130	STK24	rs17574378	intron_variant	4.00E-13
GCST90255621	13	98474049	STK24	rs9517320	intron_variant	2.00E-11
GCST90255621	13	111331897	TEX29	rs2479958	intron_variant	7.00E-21

GCST90255621	13	111535889	TEX29	rs2528787	intergenic_variant	1.00E-18
GCST009004	13	111566577	TEX29	rs12868881	intergenic_variant	1.00E-15
GCST009001	13	111566577	TEX29	rs12868881	intergenic_variant	1.00E-15
GCST90179150	13	111568949	TEX29	rs9522279	intergenic_variant	1.00E-15
GCST007039	13	111535889	TEX29	rs2528787	intergenic_variant	2.00E-15
GCST009871	13	111535889	TEX29	rs2528787	intergenic_variant	5.00E-15
GCST90018947	13	111535889	TEX29	rs2528787	intergenic_variant	5.00E-13
GCST009871	13	111331897	TEX29	rs2479958	intron_variant	8.00E-13
GCST90179150	13	111324933	TEX29	rs9522183	intron_variant	1.00E-12
GCST90267268	13	111552234	TEX29	rs9515445	intergenic_variant	7.00E-11
GCST009004	13	111329944	TEX29	rs1536053	intron_variant	8.00E-11
GCST009871	13	20042417	ZMYM2	rs9579775	intron_variant	6.00E-13
GCST007039	13	20042417	ZMYM2	rs9579775	intron_variant	2.00E-12
GCST90179150	13	20042417	ZMYM2	rs9579775	intron_variant	7.00E-12
GCST90018947	13	20042417	ZMYM2	rs9579775	intron_variant	3.00E-11
GCST90255621	14	32823916	AK5	rs1051695	missense_variant	2.00E-36
GCST009004	14	32833676	AKAP6	rs17522122	3_prime_UTR_variant	8.00E-24
GCST009003	14	32833676	AKAP6	rs17522122	3_prime_UTR_variant	8.00E-24
GCST90179150	14	32823537	AKAP6	rs2239647	synonymous_variant	2.00E-22
GCST007039	14	32823537	AKAP6	rs2239647	synonymous_variant	1.00E-21
GCST009001	14	32828192	AKAP6	rs2143975	intron_variant	8.00E-21
GCST90018947	14	32829526	AKAP6	rs34179565	intron_variant	4.00E-20
GCST009871	14	32834334	AKAP6	rs12883788	3_prime_UTR_variant	5.00E-20
GCST008129	14	32823916	AKAP6	rs1051695	missense_variant	1.00E-18
GCST006368	14	32833676	AKAP6	rs17522122	3_prime_UTR_variant	8.00E-12
GCST90255621	14	99233743	BCDIN3D	rs4082793	intron_variant	1.00E-16
GCST007039	14	99233743	BCL11B	rs4082793	intron_variant	7.00E-13
GCST90179150	14	99205451	BCL11B	rs3850422	intron_variant	7.00E-12
GCST009004	14	99206460	BCL11B	rs2693686	intron_variant	6.00E-11
GCST004904	14	93323930	BRWD1	rs3783890	intron_variant	5.00E-11
GCST90267268	14	93232657	BTBD7	rs740791	intergenic_variant	8.00E-11
GCST009871	14	103521843	CKB	rs1136165	synonymous_variant	1.00E-16
GCST007039	14	103521843	CKB	rs1136165	synonymous_variant	1.00E-15
GCST009004	14	100678259	DLK1	rs12147845	intron_variant	5.00E-12
GCST009871	14	100678259	DLK1	rs12147845	intron_variant	5.00E-12
GCST90179150	14	100678259	DLK1	rs12147845	intron_variant	5.00E-12
GCST007039	14	100678259	DLK1	rs12147845	intron_variant	1.00E-11
GCST90255621	14	100678259	DLK1	rs12147845	intron_variant	1.00E-11
GCST009871	14	100720304	DLK1	rs11160601	intergenic_variant	4.00E-11
GCST90255621	14	72856974	DPF3	rs6574100	intron_variant	4.00E-12
GCST009004	14	72856974	DPF3	rs6574100	intron_variant	1.00E-11
GCST90018947	14	72874947	DPF3	rs35020117	intron_variant	3.00E-11
GCST90255621	14	40283447	FBXO33	rs10131761	intron_variant	5.00E-12
GCST009004	14	40364973	FBXO33	rs872281	intron_variant	2.00E-11
GCST009001	14	40332547	FBXO33	rs1955540	intron_variant	2.00E-11
GCST90179150	14	40406178	FBXO33	rs2150527	intergenic_variant	2.00E-11
GCST007039	14	40375921	FBXO33	rs67272968	intron_variant	8.00E-11
GCST90255621	14	87841700	GALC	rs12888545	intron_variant	3.00E-14
GCST009004	14	87908696	GALC	rs7141307	intron_variant	6.00E-13
GCST90179150	14	87908696	GALC	rs7141307	intron_variant	5.00E-12
GCST009001	14	87855540	GALC	rs8008772	intron_variant	2.00E-11

GCST90179150	14	69323038	GALNT16	rs3902951	intron_variant	7.00E-12
GCST90255621	14	69323038	GALNT16	rs3902951	intron_variant	2.00E-11
GCST90255621	14	69328482	GALNT16	rs12879377	missense_variant	2.00E-11
GCST009004	14	103683138	KLC1	rs709400	non_coding_transcript_exon_	6.00E-19
GCST009003	14	103683138	KLC1	rs709400	non_coding_transcript_exon_	6.00E-19
GCST009871	14	103688541	KLC1	rs4900591	intron_variant	2.00E-12
GCST90255621	14	77063440	LINC02288	rs17105272	intron_variant	2.00E-11
GCST90179150	14	77063440	LINC02288	rs17105272	intron_variant	8.00E-11
GCST009004	14	42416133	LRFN5 - YWHAQP1	rs12889085	intron_variant	8.00E-12
GCST90179150	14	42416133	LRFN5 - YWHAQP1	rs12889085	intron_variant	2.00E-11
GCST007039	14	42470268	LRFN5 - YWHAQP1	rs8011566	intron_variant	2.00E-11
GCST009871	14	42470268	LRFN5 - YWHAQP1	rs8011566	intron_variant	9.00E-11
GCST90255621	14	103395985	MARK3	rs2010281	intron_variant	1.00E-21
GCST90179150	14	103395985	MARK3	rs2010281	intron_variant	2.00E-19
GCST90255621	14	46840293	MDGA2	rs1235	3_prime_UTR_variant	3.00E-25
GCST007039	14	46833911	MDGA2	rs12885458	intergenic_variant	9.00E-19
GCST009004	14	46827083	MDGA2	rs1491905	intergenic_variant	4.00E-18
GCST009001	14	46837215	MDGA2	rs4900715	intergenic_variant	5.00E-18
GCST90179150	14	46833016	MDGA2	rs4900714	intergenic_variant	5.00E-18
GCST009871	14	46829302	MDGA2	rs35697587	intergenic_variant	6.00E-18
GCST90018947	14	46889271	MDGA2	rs2933223	intron_variant	1.00E-14
GCST006368	14	101062668	MEG9	rs7161194	intron_variant	1.00E-22
GCST90179150	14	101062668	MEG9	rs7161194	intron_variant	1.00E-22
GCST007039	14	101062668	MEG9	rs7161194	intron_variant	3.00E-21
GCST009871	14	101062668	MEG9	rs7161194	intron_variant	3.00E-20
GCST90255621	14	101073662	MEG9	rs12431682	non_coding_transcript_exon_	7.00E-13
GCST009871	14	101073047	MEG9	rs7147503	intron_variant	2.00E-11
GCST90018947	14	101062668	MEG9	rs7161194	intron_variant	6.00E-11
GCST90255621	14	102315772	MOK	rs10145749	regulatory_region_variant	1.00E-12
GCST90179150	14	102273938	MOK	rs8022504	intron_variant	4.00E-11
GCST90018727	14	52045193	NID2	rs75766425	intron_variant	5.00E-11
GCST90018947	14	52045193	NID2	rs75766425	intron_variant	5.00E-11
GCST90255621	14	79478819	NRXN3	rs10146997	intron_variant	2.00E-68
GCST009004	14	79474040	NRXN3	rs7144011	intron_variant	2.00E-40
GCST009001	14	79473650	NRXN3	rs17109256	intron_variant	1.00E-39
GCST90179150	14	79433111	NRXN3	rs7141420	intron_variant	5.00E-38
GCST009003	14	79433111	NRXN3	rs7141420	intron_variant	2.00E-37
GCST007039	14	79464301	NRXN3	rs72690737	intron_variant	1.00E-32
GCST009871	14	79473182	NRXN3	rs10145154	intron_variant	2.00E-32
GCST005951	14	79433111	NRXN3	rs7141420	intron_variant	1.00E-24
GCST90255621	14	79412984	NRXN3	rs2574750	intron_variant	4.00E-21
GCST90255621	14	79033507	NRXN3	rs10146527	intron_variant	5.00E-21
GCST006368	14	79474040	NRXN3	rs7144011	intron_variant	3.00E-20
GCST009871	14	79477584	NRXN3	rs4899746	intron_variant	8.00E-20
GCST90018947	14	79433111	NRXN3	rs7141420	intron_variant	4.00E-19
GCST90179150	14	79434940	NRXN3	rs11625620	intron_variant	8.00E-17
GCST004904	14	79433111	NRXN3	rs7141420	intron_variant	7.00E-15
GCST002783	14	79433111	NRXN3	rs7141420	intron_variant	9.00E-15
GCST002783	14	79433111	NRXN3	rs7141420	intron_variant	1.00E-14
GCST90179150	14	79033507	NRXN3	rs10146527	intron_variant	1.00E-13
GCST004557	14	79433111	NRXN3	rs7141420	intron_variant	2.00E-12

GCST009871	14	79375343	NRXN3	rs35993073	intron_variant	4.00E-12
GCST004495	14	79433111	NRXN3	rs7141420	intron_variant	5.00E-12
GCST004557	14	79433111	NRXN3	rs7141420	intron_variant	1.00E-11
GCST004497	14	79433111	NRXN3	rs7141420	intron_variant	1.00E-11
GCST002783	14	79433111	NRXN3	rs7141420	intron_variant	1.00E-11
GCST90255621	14	79228625	NRXN3	rs117116987	intron_variant	1.00E-11
GCST004558	14	79433111	NRXN3	rs7141420	intron_variant	2.00E-11
GCST000830	14	79470621	NRXN3	rs10150332	intron_variant	3.00E-11
GCST004495	14	79433111	NRXN3	rs7141420	intron_variant	3.00E-11
GCST004497	14	79433111	NRXN3	rs7141420	intron_variant	3.00E-11
GCST004499	14	79433111	NRXN3	rs7141420	intron_variant	4.00E-11
GCST004558	14	79433111	NRXN3	rs7141420	intron_variant	5.00E-11
GCST90255621	14	25458973	OR7K1P	rs10132280	intergenic_variant	5.00E-45
GCST009004	14	25458973	OR7K1P	rs10132280	intergenic_variant	2.00E-33
GCST009001	14	25458973	OR7K1P	rs10132280	intergenic_variant	2.00E-33
GCST90179150	14	25458973	OR7K1P	rs10132280	intergenic_variant	3.00E-32
GCST007039	14	25458626	OR7K1P	rs12879423	intergenic_variant	6.00E-26
GCST009871	14	25465955	OR7K1P	rs7154982	intergenic_variant	2.00E-24
GCST90018947	14	25463379	OR7K1P	rs1950436	intergenic_variant	1.00E-22
GCST90255621	14	25420133	OR7K1P	rs2754087	intergenic_variant	2.00E-21
GCST009003	14	25419471	OR7K1P	rs2754084	intergenic_variant	2.00E-20
GCST90179150	14	25419471	OR7K1P	rs2754084	intergenic_variant	4.00E-20
GCST90255621	14	25523406	OR7K1P	rs17110049	intergenic_variant	5.00E-16
GCST006368	14	25458973	OR7K1P	rs10132280	intergenic_variant	6.00E-15
GCST90267268	14	25461782	OR7K1P	rs8015400	intergenic_variant	2.00E-14
GCST009871	14	25420133	OR7K1P	rs2754087	intergenic_variant	4.00E-14
GCST005951	14	25458973	OR7K1P	rs10132280	intergenic_variant	5.00E-13
GCST004904	14	25458973	OR7K1P	rs10132280	intergenic_variant	6.00E-13
GCST002783	14	25458973	OR7K1P	rs10132280	intergenic_variant	1.00E-11
GCST002783	14	25458973	OR7K1P	rs10132280	intergenic_variant	1.00E-11
GCST002783	14	25458973	OR7K1P	rs10132280	intergenic_variant	5.00E-11
GCST90255621	14	29267632	PRKD1	rs12885454	non_coding_transcript_exon_	1.00E-33
GCST009004	14	29267632	PRKD1	rs12885454	non_coding_transcript_exon_	5.00E-26
GCST009001	14	29267632	PRKD1	rs12885454	non_coding_transcript_exon_	5.00E-26
GCST90179150	14	29267632	PRKD1	rs12885454	non_coding_transcript_exon_	2.00E-25
GCST009003	14	29212088	PRKD1	rs1569979	intron_variant	3.00E-24
GCST007039	14	29211932	PRKD1	rs9788550	intron_variant	2.00E-20
GCST009871	14	29211932	PRKD1	rs9788550	intron_variant	5.00E-20
GCST90255621	14	30026513	PRKD1	rs10483389	intron_variant	1.00E-19
GCST009871	14	29268260	PRKD1	rs11624548	intron_variant	1.00E-18
GCST90255621	14	29186112	PRKD1	rs10138733	intron_variant	4.00E-16
GCST009004	14	30015516	PRKD1	rs8016859	intron_variant	7.00E-16
GCST009003	14	30015516	PRKD1	rs8016859	intron_variant	7.00E-16
GCST90179150	14	30026513	PRKD1	rs10483389	intron_variant	1.00E-15
GCST006368	14	29267632	PRKD1	rs12885454	non_coding_transcript_exon_	4.00E-14
GCST005951	14	29267632	PRKD1	rs12885454	non_coding_transcript_exon_	5.00E-14
GCST90018947	14	29213968	PRKD1	rs144355275	intron_variant	8.00E-14
GCST009871	14	29338389	PRKD1	rs75639464	intron_variant	3.00E-13
GCST007039	14	29961477	PRKD1	rs61980001	intron_variant	1.00E-11
GCST009871	14	29961477	PRKD1	rs61980001	intron_variant	2.00E-11
GCST000830	14	30045906	PRKD1	rs11847697	intron_variant	6.00E-11

GCST002783	14	29267632	PRKD1	rs12885454	non_coding_transcript_exon_	9.00E-11
GCST006368	14	30045906	PRKD1	rs11847697	intron_variant	9.00E-11
GCST90239604	14	35679545	RALGAPA1	rs75308477	intron_variant	6.00E-13
GCST90131907	14	35687824	RALGAPA1	rs76115093	intron_variant	3.00E-12
GCST90255621	14	90985439	RPS6KA5	rs1286065	intron_variant	9.00E-18
GCST90179150	14	91045995	RPS6KA5	rs1951455	intron_variant	4.00E-15
GCST009004	14	91045995	RPS6KA5	rs1951455	intron_variant	6.00E-15
GCST009003	14	91046685	RPS6KA5	rs1285997	intron_variant	7.00E-15
GCST90018947	14	91032788	RPS6KA5	rs34722618	intron_variant	4.00E-13
GCST009871	14	91046685	RPS6KA5	rs1285997	intron_variant	5.00E-13
GCST007039	14	91046685	RPS6KA5	rs1285997	intron_variant	1.00E-12
GCST007039	14	61894303	SYT16	rs217672	intron_variant	3.00E-16
GCST90179150	14	61893357	SYT16	rs217669	intron_variant	4.00E-16
GCST009004	14	61893357	SYT16	rs217669	intron_variant	6.00E-16
GCST009001	14	61894303	SYT16	rs217672	intron_variant	2.00E-15
GCST009871	14	61893357	SYT16	rs217669	intron_variant	3.00E-14
GCST90255621	14	61893357	SYT16	rs217669	intron_variant	2.00E-12
GCST90255621	14	102780133	TRAF3	rs3803286	intron_variant	2.00E-24
GCST009004	14	102782790	TRAF3	rs4906263	intron_variant	8.00E-23
GCST009003	14	102782790	TRAF3	rs4906263	intron_variant	8.00E-23
GCST009001	14	102782790	TRAF3	rs4906263	intron_variant	8.00E-23
GCST90179150	14	102780133	TRAF3	rs3803286	intron_variant	4.00E-22
GCST007039	14	102780133	TRAF3	rs3803286	intron_variant	3.00E-21
GCST009871	14	102782790	TRAF3	rs4906263	intron_variant	7.00E-20
GCST008129	14	102875712	TRAF3	rs1131877	missense_variant	2.00E-16
GCST90018947	14	102790540	TRAF3	rs12888955	intron_variant	2.00E-16
GCST009871	14	102820265	TRAF3	rs149154820	intron_variant	2.00E-11
GCST006368	14	102838088	TRAF3	rs7143963	intron_variant	8.00E-11
GCST90255621	14	93565568	UNC79	rs942066	intron_variant	1.00E-28
GCST009871	14	93557626	UNC79	rs6575340	intron_variant	1.00E-25
GCST007039	14	93557626	UNC79	rs6575340	intron_variant	5.00E-25
GCST009004	14	93565568	UNC79	rs942066	intron_variant	2.00E-24
GCST009003	14	93565568	UNC79	rs942066	intron_variant	2.00E-24
GCST90179150	14	93565568	UNC79	rs942066	intron_variant	3.00E-23
GCST009001	14	93465912	UNC79	rs11160119	intron_variant	9.00E-22
GCST90018947	14	93610632	UNC79	rs11851122	intron_variant	5.00E-21
GCST006811	14	93716037	UNC79	rs35914833	regulatory_region_variant	5.00E-14
GCST90267268	14	93418852	UNC79	rs10144067	intron_variant	2.00E-13
GCST009871	14	93718153	UNC79	rs10137191	intergenic_variant	9.00E-13
GCST90255621	14	93352870	UNC79	rs11622939	intron_variant	2.00E-11
GCST90255621	15	80701229	ABCG2	rs12914623	intron_variant	1.00E-17
GCST009004	15	80701229	ABHD17C	rs12914623	intron_variant	2.00E-16
GCST009003	15	80766311	ABHD17C	rs12593036	intergenic_variant	2.00E-15
GCST90179150	15	80766311	ABHD17C	rs12593036	intergenic_variant	3.00E-14
GCST007039	15	80696831	ABHD17C	rs34769775	intron_variant	7.00E-14
GCST009871	15	80696831	ABHD17C	rs34769775	intron_variant	9.00E-14
GCST90018947	15	80696259	ABHD17C	rs7165759	intron_variant	6.00E-12
GCST006368	15	80717305	ABHD17C	rs2759315	intron_variant	5.00E-11
GCST90255621	15	72801650	ADK	rs7164727	intergenic_variant	2.00E-29
GCST90179150	15	72801650	ADPGK	rs7164727	intergenic_variant	2.00E-22
GCST007039	15	72798942	ADPGK	rs3850986	regulatory_region_variant	3.00E-18

GCST009871	15	72797284	ADPGK	rs6495017	intron_variant	4.00E-16
GCST009001	15	72794390	ADPGK	rs12439200	non_coding_transcript_exon_	5.00E-16
GCST90255621	15	79140017	ANKRD31	rs12595749	intron_variant	6.00E-15
GCST009871	15	79148685	ANKRD34C	rs1915724	intron_variant	3.00E-14
GCST90179150	15	79140017	ANKRD34C	rs12595749	intron_variant	4.00E-14
GCST009004	15	79170694	ANKRD34C	rs8038464	intron_variant	2.00E-13
GCST009001	15	79173207	ANKRD34C	rs8041222	intron_variant	1.00E-11
GCST009004	15	72715552	BAZ1B	rs730180	intron_variant	2.00E-18
GCST008129	15	72735137	BBS4	rs2277598	missense_variant	2.00E-15
GCST009871	15	72708973	BBS4	rs11072384	intron_variant	2.00E-12
GCST004496	15	78675059	CHRNB4	rs12902602	intron_variant	6.00E-13
GCST004498	15	78675059	CHRNB4	rs12902602	intron_variant	7.00E-13
GCST004497	15	78675059	CHRNB4	rs12902602	intron_variant	2.00E-12
GCST90244062	15	78662037	CHRNB4	rs12899135	intron_variant	3.00E-12
GCST90244064	15	78662037	CHRNB4	rs12899135	intron_variant	3.00E-12
GCST90255621	15	51663859	DMXL2	rs7170980	intergenic_variant	9.00E-19
GCST009004	15	51462254	DMXL2	rs6493498	intron_variant	5.00E-17
GCST009003	15	51462254	DMXL2	rs6493498	intron_variant	5.00E-17
GCST90179150	15	51462254	DMXL2	rs6493498	intron_variant	1.00E-16
GCST007039	15	51605861	DMXL2	rs9944241	intron_variant	3.00E-12
GCST009871	15	51667233	DMXL2	rs979259	intergenic_variant	5.00E-12
GCST90018947	15	26756340	GABRB3	rs7170907	intron_variant	6.00E-12
GCST90255621	15	26789841	GABRB3	rs12594043	splice_region_variant	7.00E-11
GCST009871	15	73339360	HCN4	rs8032565	intron_variant	2.00E-14
GCST90255621	15	73391277	HCN4	rs74958060	intergenic_variant	6.00E-13
GCST90179150	15	73342241	HCN4	rs77688257	intron_variant	2.00E-12
GCST009871	15	73325897	HCN4	rs3826043	intron_variant	9.00E-12
GCST90255621	15	98690564	IGF1R	rs58553392	intron_variant	8.00E-13
GCST90018947	15	98679280	IGF1R	rs56803094	intron_variant	2.00E-12
GCST90255621	15	98961614	IGF1R	rs2654980	3_prime_UTR_variant	2.00E-12
GCST007039	15	98679280	IGF1R	rs56803094	intron_variant	1.00E-11
GCST009871	15	98679280	IGF1R	rs56803094	intron_variant	2.00E-11
GCST90267268	15	67387871	IQCH	rs12903168	intron_variant	2.00E-14
GCST009871	15	67395630	IQCH	rs4275804	intron_variant	5.00E-11
GCST90255621	15	51967910	LEO1, MAPK6	rs117632017	intron_variant	8.00E-13
GCST009871	15	51967910	LEO1, MAPK6	rs117632017	intron_variant	9.00E-13
GCST90179150	15	51967910	LEO1, MAPK6	rs117632017	intron_variant	2.00E-12
GCST90255621	15	94727238	LETR1	rs7173947	intergenic_variant	1.00E-20
GCST009004	15	94728149	LETR1	rs11633626	intergenic_variant	7.00E-19
GCST009001	15	94723581	LETR1	rs8036171	intergenic_variant	8.00E-19
GCST90179150	15	94723581	LETR1	rs8036171	intergenic_variant	8.00E-18
GCST009003	15	94731120	LETR1	rs11073383	intergenic_variant	3.00E-16
GCST007039	15	94728175	LETR1	rs7181498	intergenic_variant	2.00E-14
GCST009871	15	94728643	LETR1	rs8025516	intergenic_variant	3.00E-14
GCST009004	15	77720346	LINGO1	rs11856579	intron_variant	1.00E-16
GCST009003	15	77720346	LINGO1	rs11856579	intron_variant	1.00E-16
GCST90179150	15	77720346	LINGO1	rs11856579	intron_variant	2.00E-16
GCST007039	15	77720276	LINGO1	rs11855853	intron_variant	5.00E-14
GCST90018947	15	77720276	LINGO1	rs11855853	intron_variant	4.00E-13
GCST90255621	15	77720276	LINGO1	rs11855853	intron_variant	8.00E-12
GCST90267268	15	77708669	LINGO1	rs62009152	intron_variant	9.00E-11

GCST004046	15	58435126	LIPC	rs261332	non_coding_transcript_exon_	4.00E-42
GCST004045	15	58435126	LIPC	rs261332	non_coding_transcript_exon_	2.00E-35
GCST90255621	15	58587167	LIPC	rs12438487	intergenic_variant	3.00E-15
GCST009871	15	51742928	LYSMD2	rs11632106	intron_variant	2.00E-11
GCST90179150	15	51747879	LYSMD2	rs2622765	intron_variant	7.00E-11
GCST90179150	15	66437969	MAP2K1	rs17200912	intron_variant	2.00E-13
GCST009004	15	66449049	MAP2K1	rs11629783	intron_variant	1.00E-12
GCST90255621	15	67794500	MAP2K5	rs2241423	intron_variant	4.00E-65
GCST90179150	15	67810953	MAP2K5	rs4776976	intergenic_variant	1.00E-54
GCST007039	15	67780120	MAP2K5	rs3784710	intron_variant	2.00E-38
GCST009003	15	67600428	MAP2K5	rs3865018	intron_variant	2.00E-29
GCST90018947	15	67780120	MAP2K5	rs3784710	intron_variant	1.00E-27
GCST006368	15	67788548	MAP2K5	rs4776970	intron_variant	4.00E-24
GCST005951	15	67794500	MAP2K5	rs2241423	intron_variant	5.00E-23
GCST004904	15	67788548	MAP2K5	rs4776970	intron_variant	2.00E-19
GCST000830	15	67794500	MAP2K5	rs2241423	intron_variant	1.00E-18
GCST002783	15	67784830	MAP2K5	rs16951275	intron_variant	2.00E-18
GCST006368	15	67645417	MAP2K5	rs11637027	intron_variant	1.00E-17
GCST002783	15	67784830	MAP2K5	rs16951275	intron_variant	2.00E-17
GCST004495	15	67784830	MAP2K5	rs16951275	intron_variant	5.00E-14
GCST004497	15	67784830	MAP2K5	rs16951275	intron_variant	4.00E-13
GCST004557	15	67784061	MAP2K5	rs8025790	intron_variant	7.00E-13
GCST004557	15	67794500	MAP2K5	rs2241423	intron_variant	8.00E-13
GCST004499	15	67784830	MAP2K5	rs16951275	intron_variant	1.00E-12
GCST004558	15	67794500	MAP2K5	rs2241423	intron_variant	2.00E-12
GCST004559	15	67785292	MAP2K5	rs7175517	intron_variant	5.00E-12
GCST004558	15	67785292	MAP2K5	rs7175517	intron_variant	6.00E-12
GCST004495	15	67784830	MAP2K5	rs16951275	intron_variant	2.00E-11
GCST009871	15	67736484	MAP2K5	rs117049462	intron_variant	4.00E-11
GCST009371	15	67798486	MAP2K5	rs2127162	intron_variant	5.00E-11
GCST006802	15	67784830	MAP2K5	rs16951275	intron_variant	6.00E-11
GCST004497	15	67784830	MAP2K5	rs16951275	intron_variant	9.00E-11
GCST002783	15	67784830	MAP2K5	rs16951275	intron_variant	9.00E-11
GCST90271770	15	52061301	MAPK3	rs35697691	missense_variant	5.00E-16
GCST90255621	15	89384958	MIR9-3HG	rs150353	intron_variant	9.00E-13
GCST90255621	15	89417055	MIR9-3HG	rs62020775	intron_variant	2.00E-11
GCST90255621	15	59181770	MYO1E	rs8041615	intron_variant	6.00E-12
GCST90179150	15	59178167	MYO1E	rs2899644	intron_variant	3.00E-11
GCST007039	15	62074814	NPM1P47	rs72749772	intergenic_variant	4.00E-12
GCST001527	15	62104743	NPM1P47	rs7173964	regulatory_region_variant	6.00E-12
GCST90255621	15	52850973	ONECUT1 - RPSAP55	rs16965225	intergenic_variant	2.00E-14
GCST90255621	15	52873941	ONECUT1 - RPSAP55	rs1899730	intergenic_variant	4.00E-11
GCST90179150	15	52850973	ONECUT1 - RPSAP55	rs16965225	intergenic_variant	6.00E-11
GCST90255621	15	31585487	OTUD7A	rs7172627	intron_variant	2.00E-12
GCST90179150	15	31585487	OTUD7A	rs7172627	intron_variant	5.00E-11
GCST009004	15	67889196	PIAS1	rs1996120	intergenic_variant	4.00E-24
GCST009001	15	67889196	PIAS1	rs1996120	intergenic_variant	4.00E-24
GCST009871	15	67881583	PIAS1	rs338364	intergenic_variant	3.00E-23
GCST90103755	15	67845026	PIAS1	rs4776990	intron_variant	1.00E-21
GCST007039	15	79120057	RASGRF1	rs1521624	intergenic_variant	6.00E-16
GCST90018947	15	79111243	RASGRF1	rs2870111	intergenic_variant	4.00E-12

GCST90255621	15	73490581	REC114	rs142592509	intron_variant	8.00E-13
GCST009871	15	73490581	REC114	rs142592509	intron_variant	3.00E-11
GCST90255621	15	60602766	RORA	rs340029	intron_variant	1.00E-19
GCST009004	15	60616108	RORA	rs340025	intron_variant	1.00E-14
GCST009003	15	60616108	RORA	rs340025	intron_variant	1.00E-14
GCST90179150	15	60616108	RORA	rs340025	intron_variant	5.00E-14
GCST90255621	15	47371673	SEMA6D	rs12913596	intron_variant	2.00E-16
GCST90255621	15	47622494	SEMA6D	rs1912631	intron_variant	6.00E-15
GCST90018947	15	47457237	SEMA6D	rs11857221	intron_variant	9.00E-14
GCST90255621	15	47445866	SEMA6D	rs1559677	intron_variant	2.00E-12
GCST90179150	15	47445866	SEMA6D	rs1559677	intron_variant	3.00E-12
GCST90104633	15	83615800	SH3GL3	rs2585058	intron_variant	9.00E-12
GCST90104632	15	83615800	SH3GL3	rs2585058	intron_variant	2.00E-11
GCST90255621	15	67834337	SKOR1	rs3803522	3_prime_UTR_variant	1.00E-18
GCST90179150	15	67834337	SKOR1	rs3803522	3_prime_UTR_variant	3.00E-14
GCST90131907	15	67829090	SKOR1	rs28539889	intron_variant	1.00E-11
GCST009871	15	67834337	SKOR1	rs3803522	3_prime_UTR_variant	3.00E-11
GCST90255621	15	92030832	SLCO3A1	rs58431213	intron_variant	3.00E-14
GCST009004	15	92030409	SLCO3A1	rs7498044	intron_variant	5.00E-12
GCST90179150	15	92029532	SLCO3A1	rs72757415	intron_variant	1.00E-11
GCST90255621	15	46292589	SQOR - MTND5P40	rs12439798	intergenic_variant	4.00E-18
GCST009871	15	46293890	SQOR - MTND5P40	rs7183417	intergenic_variant	2.00E-14
GCST007039	15	46293897	SQOR - MTND5P40	rs7162533	intergenic_variant	7.00E-14
GCST009004	15	46292589	SQOR - MTND5P40	rs12439798	intergenic_variant	1.00E-13
GCST90179150	15	46292589	SQOR - MTND5P40	rs12439798	intergenic_variant	5.00E-13
GCST90018947	15	46292589	SQOR - MTND5P40	rs12439798	intergenic_variant	3.00E-12
GCST009871	15	73985785	STOML1	rs35364449	non_coding_transcript_exon_	2.00E-13
GCST007039	15	73985785	STOML1	rs35364449	non_coding_transcript_exon_	2.00E-13
GCST90255621	15	73985785	STOML1	rs35364449	non_coding_transcript_exon_	2.00E-12
GCST90018947	15	73985785	STOML1	rs35364449	non_coding_transcript_exon_	4.00E-12
GCST90179150	15	73985785	STOML1	rs35364449	non_coding_transcript_exon_	5.00E-12
GCST90179150	15	63501039	USP3	rs11635675	non_coding_transcript_exon_	9.00E-13
GCST90255621	15	63501039	USP3	rs11635675	non_coding_transcript_exon_	9.00E-13
GCST009004	15	61830340	VPS13C	rs12438629	intergenic_variant	2.00E-11
GCST90255621	15	62023836	VPS13C	rs12595158	missense_variant	9.00E-16
GCST90018947	15	62051133	VPS13C	rs35057371	intron_variant	2.00E-13
GCST90179150	15	62023836	VPS13C	rs12595158	missense_variant	7.00E-13
GCST90255621	15	53150733	WDR72	rs11070956	intergenic_variant	6.00E-16
GCST009004	15	53181793	WDR72	rs8024806	intergenic_variant	7.00E-13
GCST90179150	15	53165329	WDR72	rs1877139	intergenic_variant	3.00E-12
GCST90179150	15	53129207	WDR72	rs17659514	intron_variant	2.00E-11
GCST90271770	16	70282008	AARS1	rs775208	intron_variant	2.00E-22
GCST90179150	16	70282008	AARS1	rs775208	intron_variant	3.00E-19
GCST009871	16	70269677	AARS1	rs2070203	missense_variant	9.00E-19
GCST007039	16	70275334	AARS1	rs12149660	intron_variant	7.00E-14
GCST009871	16	70275334	AARS1	rs12149660	intron_variant	2.00E-13
GCST90255621	16	3965728	ADCY5	rs879620	3_prime_UTR_variant	9.00E-57
GCST90179150	16	3965728	ADCY9	rs879620	3_prime_UTR_variant	3.00E-39
GCST90018947	16	3965728	ADCY9	rs879620	3_prime_UTR_variant	6.00E-35
GCST007039	16	3964281	ADCY9	rs2238435	3_prime_UTR_variant	2.00E-31
GCST004904	16	3965728	ADCY9	rs879620	3_prime_UTR_variant	9.00E-19

GCST009004	16	3985067	ADCY9	rs2601777	intron_variant	3.00E-17
GCST009001	16	3985067	ADCY9	rs2601777	intron_variant	3.00E-17
GCST009871	16	3993174	ADCY9	rs2601791	intron_variant	3.00E-17
GCST009003	16	3972702	ADCY9	rs7185966	intron_variant	2.00E-16
GCST005951	16	3963466	ADCY9	rs2531995	3_prime_UTR_variant	9.00E-15
GCST006368	16	3965728	ADCY9	rs879620	3_prime_UTR_variant	4.00E-14
GCST90179150	16	3992448	ADCY9	rs2239307	intron_variant	9.00E-14
GCST011332	16	3965728	ADCY9	rs879620	3_prime_UTR_variant	6.00E-13
GCST004904	16	3972693	ADCY9	rs2540034	intron_variant	3.00E-12
GCST90018727	16	3965728	ADCY9	rs879620	3_prime_UTR_variant	6.00E-12
GCST004557	16	3963466	ADCY9	rs2531995	3_prime_UTR_variant	1.00E-11
GCST004557	16	3963466	ADCY9	rs2531995	3_prime_UTR_variant	2.00E-11
GCST90255621	16	3987393	ADCY9	rs2239304	intron_variant	2.00E-11
GCST004558	16	3963466	ADCY9	rs2531995	3_prime_UTR_variant	3.00E-11
GCST004558	16	3963466	ADCY9	rs2531995	3_prime_UTR_variant	5.00E-11
GCST90239604	16	3964281	ADCY9	rs2238435	3_prime_UTR_variant	5.00E-11
GCST90255621	16	70251998	AIF1	rs4985407	3_prime_UTR_variant	7.00E-26
GCST90179150	16	28878165	ASXL3	rs3888190	non_coding_transcript_exon_	1.00E-68
GCST009001	16	28883809	ATP2A1	rs8061590	intron_variant	3.00E-66
GCST90018947	16	28881816	ATP2A1	rs72793818	intron_variant	2.00E-37
GCST004904	16	28878165	ATP2A1	rs3888190	non_coding_transcript_exon_	2.00E-31
GCST005951	16	28878165	ATP2A1	rs3888190	non_coding_transcript_exon_	1.00E-29
GCST002783	16	28878165	ATP2A1	rs3888190	non_coding_transcript_exon_	3.00E-25
GCST009371	16	28878165	ATP2A1	rs3888190	non_coding_transcript_exon_	1.00E-24
GCST002783	16	28878165	ATP2A1	rs3888190	non_coding_transcript_exon_	3.00E-23
GCST004495	16	28878165	ATP2A1	rs3888190	non_coding_transcript_exon_	2.00E-18
GCST004497	16	28878165	ATP2A1	rs3888190	non_coding_transcript_exon_	6.00E-18
GCST002783	16	28878165	ATP2A1	rs3888190	non_coding_transcript_exon_	1.00E-17
GCST004499	16	28878165	ATP2A1	rs3888190	non_coding_transcript_exon_	4.00E-15
GCST002783	16	28878165	ATP2A1	rs3888190	non_coding_transcript_exon_	4.00E-12
GCST004497	16	28878165	ATP2A1	rs3888190	non_coding_transcript_exon_	2.00E-11
GCST004495	16	28878165	ATP2A1	rs3888190	non_coding_transcript_exon_	6.00E-11
GCST006368	16	28837347	ATXN2	rs12325113	intergenic_variant	4.00E-29
GCST004557	16	28837347	ATXN2L	rs12325113	intergenic_variant	2.00E-15
GCST004558	16	28837347	ATXN2L	rs12325113	intergenic_variant	9.00E-15
GCST004559	16	28837347	ATXN2L	rs12325113	intergenic_variant	2.00E-13
GCST90255621	16	337867	AUTS2	rs11866815	intron_variant	1.00E-17
GCST009004	16	337867	AXIN1	rs11866815	intron_variant	2.00E-16
GCST90179150	16	298687	AXIN1	rs214249	intron_variant	1.00E-15
GCST009871	16	357800	AXIN1	rs7206329	intergenic_variant	5.00E-14
GCST90018947	16	299331	AXIN1	rs7200589	intron_variant	7.00E-14
GCST007039	16	357723	AXIN1	rs7201895	intergenic_variant	1.00E-13
GCST009003	16	299331	AXIN1	rs7200589	intron_variant	4.00E-13
GCST005951	16	337867	AXIN1	rs11866815	intron_variant	5.00E-11
GCST009871	16	28940136	CD19	rs11645302	intergenic_variant	1.00E-11
GCST90179150	16	28940136	CD19	rs11645302	intergenic_variant	3.00E-11
GCST90255621	16	82839023	CDH13	rs7206608	intron_variant	1.00E-18
GCST90255621	16	82404732	CDH13	rs12922346	intergenic_variant	2.00E-16
GCST009004	16	82839023	CDH13	rs7206608	intron_variant	1.00E-12
GCST007039	16	82414590	CDH13	rs11150461	regulatory_region_variant	3.00E-12
GCST009004	16	82404732	CDH13	rs12922346	intergenic_variant	1.00E-11

GCST90179150	16	82422346	CDH13	rs60449588	intergenic_variant	3.00E-11
GCST90255621	16	82617112	CDH13	rs3865188	intergenic_variant	9.00E-11
GCST90179150	16	3522694	CLUAP1	rs8054140	intron_variant	2.00E-15
GCST007039	16	3533173	CLUAP1	rs3751837	3_prime_UTR_variant	4.00E-11
GCST009871	16	3533173	CLUAP1	rs3751837	3_prime_UTR_variant	7.00E-11
GCST90018947	16	81701407	CMIP	rs3751859	intron_variant	3.00E-13
GCST009004	16	81694476	CMIP	rs2012502	intron_variant	2.00E-12
GCST009003	16	81694476	CMIP	rs2012502	intron_variant	2.00E-12
GCST90255621	16	81701407	CMIP	rs3751859	intron_variant	3.00E-12
GCST90179150	16	81694476	CMIP	rs2012502	intron_variant	1.00E-11
GCST90255621	16	62804400	DPPA3P11	rs1896767	intergenic_variant	1.00E-14
GCST009004	16	62769937	DPPA3P11	rs11075489	intergenic_variant	1.00E-11
GCST90255621	16	63040537	DPPA3P11	rs153322	intergenic_variant	2.00E-11
GCST90179150	16	62769937	DPPA3P11	rs11075489	intergenic_variant	2.00E-11
GCST009871	16	53776634	FTO	rs7187250	intron_variant	2.00E-306
GCST007039	16	53767042	FTO	rs1421085	intron_variant	1.00E-300
GCST90018947	16	53768582	FTO	rs11642015	intron_variant	9.00E-290
GCST009001	16	53776774	FTO	rs7193144	intron_variant	3.00E-242
GCST004904	16	53769662	FTO	rs1558902	intron_variant	2.00E-223
GCST90103755	16	53769662	FTO	rs1558902	intron_variant	6.00E-222
GCST009121	16	53768582	FTO	rs11642015	intron_variant	7.00E-217
GCST006368	16	53769662	FTO	rs1558902	intron_variant	2.00E-210
GCST009003	16	53775335	FTO	rs1121980	intron_variant	1.00E-206
GCST005951	16	53769662	FTO	rs1558902	intron_variant	9.00E-199
GCST005951	16	53785257	FTO	rs9936385	intron_variant	4.00E-188
GCST005950	16	53785257	FTO	rs9936385	intron_variant	2.00E-187
GCST90090977	16	53787213	FTO	rs17817712	intron_variant	2.00E-177
GCST002783	16	53769662	FTO	rs1558902	intron_variant	1.00E-156
GCST002783	16	53769662	FTO	rs1558902	intron_variant	8.00E-153
GCST004519	16	53794154	FTO	rs17817964	intron_variant	1.00E-146
GCST90090978	16	53787213	FTO	rs17817712	intron_variant	1.00E-121
GCST000830	16	53769662	FTO	rs1558902	intron_variant	5.00E-120
GCST004497	16	53769662	FTO	rs1558902	intron_variant	2.00E-115
GCST004495	16	53769662	FTO	rs1558902	intron_variant	3.00E-110
GCST004558	16	53784548	FTO	rs3751812	intron_variant	3.00E-109
GCST004558	16	53784548	FTO	rs3751812	intron_variant	2.00E-108
GCST004557	16	53784548	FTO	rs3751812	intron_variant	6.00E-108
GCST004557	16	53766065	FTO	rs9930333	intron_variant	1.00E-103
GCST007241	16	53765993	FTO	rs9928094	intron_variant	6.00E-101
GCST005952	16	53785257	FTO	rs9936385	intron_variant	1.00E-97
GCST005953	16	53785257	FTO	rs9936385	intron_variant	5.00E-95
GCST002783	16	53769662	FTO	rs1558902	intron_variant	1.00E-93
GCST90018727	16	53768582	FTO	rs11642015	intron_variant	2.00E-85
GCST002783	16	53769662	FTO	rs1558902	intron_variant	4.00E-84
GCST004904	16	53768582	FTO	rs11642015	intron_variant	2.00E-81
GCST009871	16	53763653	FTO	rs4784323	intron_variant	8.00E-80
GCST004499	16	53769662	FTO	rs1558902	intron_variant	1.00E-79
GCST007240	16	53766065	FTO	rs9930333	intron_variant	9.00E-76
GCST006802	16	53767042	FTO	rs1421085	intron_variant	2.00E-75
GCST90271560	16	53767042	FTO	rs1421085	intron_variant	1.00E-73
GCST009107	16	53768582	FTO	rs11642015	intron_variant	2.00E-73

GCST004559	16	53766065	FTO	rs9930333	intron_variant	5.00E-72
GCST004559	16	53766065	FTO	rs9930333	intron_variant	2.00E-71
GCST009871	16	53783406	FTO	rs113191842	intron_variant	2.00E-69
GCST004558	16	53766065	FTO	rs9930333	intron_variant	1.00E-68
GCST004558	16	53766065	FTO	rs9930333	intron_variant	2.00E-68
GCST004495	16	53769662	FTO	rs1558902	intron_variant	7.00E-65
GCST004557	16	53766065	FTO	rs9930333	intron_variant	2.00E-64
GCST004557	16	53766065	FTO	rs9930333	intron_variant	2.00E-64
GCST004497	16	53769662	FTO	rs1558902	intron_variant	2.00E-63
GCST004497	16	53769662	FTO	rs1558902	intron_variant	2.00E-62
GCST004495	16	53769662	FTO	rs1558902	intron_variant	5.00E-59
GCST004557	16	53782363	FTO	rs8050136	intron_variant	2.00E-58
GCST90255621	16	53843680	FTO	rs6499653	intron_variant	1.00E-56
GCST004558	16	53782363	FTO	rs8050136	intron_variant	4.00E-54
GCST90179150	16	53735381	FTO	rs7203521	intron_variant	6.00E-54
GCST004557	16	53782363	FTO	rs8050136	intron_variant	1.00E-52
GCST001955	16	53785981	FTO	rs11075990	intron_variant	2.00E-51
GCST000298	16	53786615	FTO	rs9939609	intron_variant	4.00E-51
GCST90239604	16	53767042	FTO	rs1421085	intron_variant	5.00E-49
GCST000296	16	53782363	FTO	rs8050136	intron_variant	1.00E-47
GCST004499	16	53769662	FTO	rs1558902	intron_variant	3.00E-45
GCST004499	16	53769662	FTO	rs1558902	intron_variant	4.00E-44
GCST009764	16	53767042	FTO	rs1421085	intron_variant	9.00E-44
GCST004559	16	53766065	FTO	rs9930333	intron_variant	9.00E-44
GCST004559	16	53766065	FTO	rs9930333	intron_variant	2.00E-43
GCST009871	16	53735381	FTO	rs7203521	intron_variant	3.00E-42
GCST004560	16	53784548	FTO	rs3751812	intron_variant	5.00E-42
GCST004560	16	53784548	FTO	rs3751812	intron_variant	5.00E-41
GCST90255621	16	53773586	FTO	rs16952522	intron_variant	5.00E-41
GCST90179150	16	53832063	FTO	rs9302652	intron_variant	7.00E-41
GCST008158	16	53769662	FTO	rs1558902	intron_variant	4.00E-40
GCST009003	16	53832063	FTO	rs9302652	intron_variant	9.00E-40
GCST004559	16	53782363	FTO	rs8050136	intron_variant	4.00E-39
GCST004498	16	53769662	FTO	rs1558902	intron_variant	8.00E-39
GCST90131907	16	53767042	FTO	rs1421085	intron_variant	9.00E-39
GCST009871	16	53760557	FTO	rs17525605	intron_variant	1.00E-37
GCST000022	16	53786615	FTO	rs9939609	intron_variant	3.00E-35
GCST90255621	16	53800772	FTO	rs12596054	intron_variant	8.00E-35
GCST004558	16	53784548	FTO	rs3751812	intron_variant	3.00E-32
GCST90095034	16	53769662	FTO	rs1558902	intron_variant	3.00E-32
GCST009871	16	53832063	FTO	rs9302652	intron_variant	2.00E-31
GCST009555	16	53767042	FTO	rs1421085	intron_variant	3.00E-30
GCST004560	16	53791576	FTO	rs9941349	intron_variant	2.00E-29
GCST90255621	16	53831789	FTO	rs10521307	intron_variant	3.00E-29
GCST008025	16	53769662	FTO	rs1558902	intron_variant	4.00E-29
GCST90095034	16	53797234	FTO	rs9922708	intron_variant	6.00E-29
GCST008025	16	53767042	FTO	rs1421085	intron_variant	7.00E-29
GCST008025	16	53777876	FTO	rs62033400	intron_variant	3.00E-28
GCST008025	16	53794154	FTO	rs17817964	intron_variant	3.00E-28
GCST004560	16	53784548	FTO	rs3751812	intron_variant	7.00E-28
GCST90255621	16	53758552	FTO	rs74498370	intron_variant	2.00E-27

GCST90255621	16	53739135	FTO	rs4396532	intron_variant	4.00E-27
GCST002461	16	53769662	FTO	rs1558902	intron_variant	7.00E-27
GCST002893	16	53769662	FTO	rs1558902	intron_variant	1.00E-26
GCST002894	16	53769662	FTO	rs1558902	intron_variant	1.00E-26
GCST008025	16	53808996	FTO	rs12149832	intron_variant	1.00E-25
GCST90255621	16	53787217	FTO	rs112232322	intron_variant	3.00E-25
GCST90255621	16	53779586	FTO	rs8063946	intron_variant	5.00E-25
GCST002021	16	53766842	FTO	rs9940128	intron_variant	4.00E-23
GCST004498	16	53769662	FTO	rs1558902	intron_variant	4.00E-22
GCST90179150	16	53778612	FTO	rs16945088	intron_variant	4.00E-22
GCST001416	16	53808996	FTO	rs12149832	intron_variant	5.00E-22
GCST009871	16	53773586	FTO	rs16952522	intron_variant	8.00E-22
GCST008025	16	53791576	FTO	rs9941349	intron_variant	4.00E-21
GCST004208	16	53775211	FTO	rs55872725	intron_variant	6.00E-21
GCST90255621	16	53808800	FTO	rs111357538	intron_variant	7.00E-21
GCST90095034	16	53784796	FTO	rs3751813	intron_variant	8.00E-21
GCST004498	16	53769662	FTO	rs1558902	intron_variant	9.00E-21
GCST90255621	16	53728765	FTO	rs368270837	intron_variant	1.00E-20
GCST009871	16	53758552	FTO	rs74498370	intron_variant	5.00E-20
GCST90255622	16	53767042	FTO	rs1421085	intron_variant	7.00E-20
GCST004559	16	53784548	FTO	rs3751812	intron_variant	2.00E-19
GCST90267268	16	53756994	FTO	rs1075440	intron_variant	2.00E-19
GCST90255621	16	53794987	FTO	rs79226694	intron_variant	2.00E-19
GCST90179150	16	53758552	FTO	rs74498370	intron_variant	4.00E-19
GCST008025	16	53779455	FTO	rs17817449	intron_variant	5.00E-19
GCST90179150	16	53794987	FTO	rs79226694	intron_variant	7.00E-19
GCST90179150	16	53872187	FTO	rs8060649	intron_variant	1.00E-18
GCST004560	16	53782363	FTO	rs8050136	intron_variant	2.00E-18
GCST009871	16	53767204	FTO	rs111240785	intron_variant	3.00E-18
GCST90255621	16	53717471	FTO	rs16952465	intron_variant	3.00E-18
GCST008025	16	53796553	FTO	rs9930506	intron_variant	4.00E-18
GCST008025	16	53779538	FTO	rs8043757	intron_variant	8.00E-18
GCST90002409	16	53779455	FTO	rs17817449	intron_variant	2.00E-17
GCST009871	16	53780558	FTO	rs62033401	intron_variant	2.00E-17
GCST004046	16	53786615	FTO	rs9939609	intron_variant	2.00E-17
GCST008025	16	53782363	FTO	rs8050136	intron_variant	7.00E-17
GCST002894	16	53769662	FTO	rs1558902	intron_variant	2.00E-16
GCST90179150	16	53787217	FTO	rs112232322	intron_variant	3.00E-16
GCST009871	16	53820852	FTO	rs8054908	intron_variant	4.00E-16
GCST003177	16	53767042	FTO	rs1421085	intron_variant	5.00E-16
GCST009871	16	53795056	FTO	rs73607075	intron_variant	5.00E-16
GCST008025	16	53785981	FTO	rs11075990	intron_variant	7.00E-16
GCST008025	16	53786615	FTO	rs9939609	intron_variant	9.00E-16
GCST008025	16	53766842	FTO	rs9940128	intron_variant	9.00E-16
GCST004560	16	53784548	FTO	rs3751812	intron_variant	1.00E-15
GCST008025	16	53788739	FTO	rs7185735	intron_variant	1.00E-15
GCST008025	16	53787703	FTO	rs7202116	intron_variant	1.00E-15
GCST008025	16	53775335	FTO	rs1121980	intron_variant	1.00E-15
GCST90179150	16	53809936	FTO	rs75582195	intron_variant	2.00E-15
GCST004045	16	53786615	FTO	rs9939609	intron_variant	1.00E-14
GCST002227	16	53777876	FTO	rs62033400	intron_variant	2.00E-14

GCST009871	16	53872940	FTO	rs7188378	intron_variant	4.00E-14
GCST003177	16	53769662	FTO	rs1558902	intron_variant	4.00E-14
GCST90103751	16	53767042	FTO	rs1421085	intron_variant	5.00E-14
GCST90179150	16	53839516	FTO	rs17819063	intron_variant	1.00E-13
GCST000296	16	53735765	FTO	rs6499640	intron_variant	4.00E-13
GCST90104640	16	53773852	FTO	rs17817288	intron_variant	1.00E-12
GCST90095034	16	53764611	FTO	rs8047395	intron_variant	1.00E-12
GCST009299	16	53797234	FTO	rs9922708	intron_variant	3.00E-12
GCST90179150	16	54117066	FTO	rs11076022	3_prime_UTR_variant	1.00E-11
GCST90255621	16	53780915	FTO	rs149264883	intron_variant	1.00E-11
GCST009871	16	53771614	FTO	rs147870440	intron_variant	2.00E-11
GCST009871	16	53783051	FTO	rs181337907	intron_variant	2.00E-11
GCST90179150	16	53771614	FTO	rs147870440	intron_variant	2.00E-11
GCST90255621	16	53750466	FTO	rs149715977	intron_variant	2.00E-11
GCST004519	16	53794154	FTO	rs17817964	intron_variant	3.00E-11
GCST90255621	16	53815869	FTO	rs111370419	intron_variant	3.00E-11
GCST009004	16	54118818	FTO	rs2072518	3_prime_UTR_variant	7.00E-11
GCST90255621	16	20243801	GP2	rs11074446	regulatory_region_variant	3.00E-23
GCST90018947	16	20248613	GP2	rs57508503	intergenic_variant	2.00E-21
GCST009004	16	20243801	GP2	rs11074446	regulatory_region_variant	4.00E-19
GCST009003	16	20243801	GP2	rs11074446	regulatory_region_variant	4.00E-19
GCST90179150	16	20243801	GP2	rs11074446	regulatory_region_variant	2.00E-18
GCST004904	16	20243775	GP2	rs4238585	regulatory_region_variant	1.00E-17
GCST90131907	16	20244860	GP2	rs28516382	intergenic_variant	3.00E-14
GCST90018727	16	20247110	GP2	rs12597682	intergenic_variant	2.00E-13
GCST006368	16	20243801	GP2	rs11074446	regulatory_region_variant	6.00E-12
GCST004904	16	20247110	GP2	rs12597682	intergenic_variant	8.00E-12
GCST90255621	16	20043049	GPR139	rs889201	intron_variant	3.00E-26
GCST90179150	16	20043049	GPR139	rs889201	intron_variant	2.00E-19
GCST009001	16	20237144	GPR139	rs6497451	intergenic_variant	4.00E-18
GCST009871	16	20039144	GPR139	rs868554	intron_variant	4.00E-17
GCST002783	16	19924067	GPR139	rs12446632	intergenic_variant	1.00E-13
GCST90239604	16	20237087	GPR139	rs12599169	intergenic_variant	2.00E-13
GCST007039	16	20233961	GPR139	rs4432271	intergenic_variant	7.00E-13
GCST004497	16	19924067	GPR139	rs12446632	intergenic_variant	3.00E-11
GCST004557	16	19924067	GPR139	rs12446632	intergenic_variant	6.00E-11
GCST008158	16	19964085	GPR139	rs8054079	intergenic_variant	7.00E-11
GCST90255621	16	20131422	GPR139	rs116235638	intergenic_variant	8.00E-11
GCST90255621	16	19922278	GPRC5B	rs12444979	intergenic_variant	3.00E-59
GCST90179150	16	19933041	GPRC5B	rs11639988	intergenic_variant	2.00E-51
GCST009004	16	19924067	GPRC5B	rs12446632	intergenic_variant	3.00E-50
GCST009001	16	19924067	GPRC5B	rs12446632	intergenic_variant	3.00E-50
GCST009003	16	19924067	GPRC5B	rs12446632	intergenic_variant	3.00E-50
GCST90271769	16	19924067	GPRC5B	rs12446632	intergenic_variant	6.00E-33
GCST009871	16	19922189	GPRC5B	rs72771049	intergenic_variant	4.00E-32
GCST007039	16	19931605	GPRC5B	rs7204864	intergenic_variant	3.00E-31
GCST005951	16	19924067	GPRC5B	rs12446632	intergenic_variant	1.00E-26
GCST000830	16	19922278	GPRC5B	rs12444979	intergenic_variant	3.00E-21
GCST006368	16	19924067	GPRC5B	rs12446632	intergenic_variant	9.00E-20
GCST002783	16	19924067	GPRC5B	rs12446632	intergenic_variant	2.00E-19
GCST002783	16	19924067	GPRC5B	rs12446632	intergenic_variant	1.00E-18

GCST90255621	16	9642000	GRIN2A	rs316371	intergenic_variant	7.00E-16
GCST009004	16	9619831	GRIN2A	rs1990573	intergenic_variant	2.00E-13
GCST009001	16	9619831	GRIN2A	rs1990573	intergenic_variant	2.00E-13
GCST90179150	16	9619831	GRIN2A	rs1990573	intergenic_variant	2.00E-12
GCST009871	16	9626273	GRIN2A	rs8062638	intergenic_variant	1.00E-11
GCST007039	16	9626273	GRIN2A	rs8062638	intergenic_variant	2.00E-11
GCST009871	16	9639874	GRIN2A	rs35014268	intergenic_variant	9.00E-11
GCST004046	16	56956804	HERPUD1 - CETP	rs247617	regulatory_region_variant	4.00E-165
GCST004045	16	56956804	HERPUD1 - CETP	rs247617	regulatory_region_variant	1.00E-149
GCST90271770	16	70657405	IL34	rs12935589	intron_variant	4.00E-16
GCST90271767	16	70657405	IL34	rs12935589	intron_variant	3.00E-12
GCST90179150	16	70657405	IL34	rs12935589	intron_variant	5.00E-11
GCST90179150	16	30503402	ITGAL	rs4243232	intron_variant	3.00E-17
GCST90255621	16	30503402	ITGAL	rs4243232	intron_variant	3.00E-15
GCST009001	16	31356857	ITGAX	rs7190997	intron_variant	3.00E-18
GCST009871	16	31356857	ITGAX	rs7190997	intron_variant	1.00E-13
GCST90255621	16	31130672	KAT8	rs1549293	3_prime_UTR_variant	2.00E-34
GCST90179150	16	31130672	KAT8	rs1549293	3_prime_UTR_variant	1.00E-33
GCST006368	16	31118574	KAT8	rs9925964	splice_region_variant	2.00E-13
GCST005951	16	31118574	KAT8	rs9925964	splice_region_variant	7.00E-13
GCST002783	16	31118574	KAT8	rs9925964	splice_region_variant	9.00E-11
GCST009001	16	30130700	MAPK3	rs57149692	regulatory_region_variant	1.00E-31
GCST90267268	16	30123358	MAPK3	rs61764202	intron_variant	9.00E-12
GCST90255621	16	69554669	NFAT5	rs1364063	TF_binding_site_variant	2.00E-43
GCST90271770	16	69522812	NFAT5	rs889398	regulatory_region_variant	2.00E-35
GCST009004	16	69522812	NFAT5	rs889398	regulatory_region_variant	3.00E-32
GCST009001	16	69522812	NFAT5	rs889398	regulatory_region_variant	3.00E-32
GCST009871	16	69617963	NFAT5	rs862320	intron_variant	3.00E-32
GCST90179150	16	69522812	NFAT5	rs889398	regulatory_region_variant	5.00E-32
GCST007039	16	69617963	NFAT5	rs862320	intron_variant	7.00E-32
GCST009003	16	69515846	NFAT5	rs11866219	regulatory_region_variant	5.00E-28
GCST90018947	16	69559452	NFAT5	rs12599637	intergenic_variant	9.00E-23
GCST009871	16	69541335	NFAT5	rs62052820	intergenic_variant	2.00E-16
GCST90267268	16	69513838	NFAT5	rs4783718	regulatory_region_variant	5.00E-16
GCST90255621	16	69693577	NFAT5	rs145602190	missense_variant	9.00E-14
GCST005951	16	69522812	NFAT5	rs889398	regulatory_region_variant	5.00E-11
GCST90255621	16	3549655	NLRC3	rs12448257	intron_variant	4.00E-24
GCST009004	16	3549655	NLRC3	rs12448257	intron_variant	9.00E-16
GCST009001	16	3549655	NLRC3	rs12448257	intron_variant	9.00E-16
GCST90255621	16	2047157	NTHL1	rs2516739	non_coding_transcript_exon_	2.00E-17
GCST90179150	16	2047157	NTHL1	rs2516739	non_coding_transcript_exon_	1.00E-14
GCST007039	16	2047157	NTHL1	rs2516739	non_coding_transcript_exon_	2.00E-11
GCST90255621	16	56437498	NUDT21	rs7189122	intron_variant	6.00E-16
GCST009004	16	56437498	NUDT21	rs7189122	intron_variant	4.00E-11
GCST90179150	16	56437498	NUDT21	rs7189122	intron_variant	6.00E-11
GCST90255621	16	20359494	PDILT	rs9652589	missense_variant	2.00E-27
GCST009004	16	20359753	PDILT	rs9929792	intron_variant	2.00E-19
GCST009003	16	20359753	PDILT	rs9929792	intron_variant	2.00E-19
GCST90179150	16	20364029	PDILT	rs9931967	intron_variant	3.00E-19
GCST009871	16	20359753	PDILT	rs9929792	intron_variant	7.00E-17
GCST007039	16	20368682	PDILT	rs4780885	intron_variant	1.00E-16

GCST008129	16	20359494	PDILT	rs9652589	missense_variant	4.00E-13
GCST90255621	16	15036113	PDXDC1	rs7200543	synonymous_variant	8.00E-17
GCST011334	16	15035602	PDXDC1	rs4985155	intron_variant	1.00E-12
GCST90255621	16	71961033	PKD1L3	rs12926804	intron_variant	1.00E-16
GCST90271770	16	71954829	PKD1L3	rs2303220	intron_variant	1.00E-13
GCST009871	16	72000754	PKD1L3	rs12599952	intergenic_variant	3.00E-13
GCST009871	16	71961033	PKD1L3	rs12926804	intron_variant	4.00E-13
GCST90179150	16	71961033	PKD1L3	rs12926804	intron_variant	4.00E-11
GCST90255621	16	72217233	PMFBP1	rs811054	intergenic_variant	2.00E-20
GCST009003	16	72217233	PMFBP1	rs811054	intergenic_variant	2.00E-16
GCST90179150	16	72217233	PMFBP1	rs811054	intergenic_variant	2.00E-16
GCST009871	16	72217233	PMFBP1	rs811054	intergenic_variant	7.00E-14
GCST007039	16	72217233	PMFBP1	rs811054	intergenic_variant	8.00E-14
GCST009871	16	72181075	PMFBP1	rs217179	intergenic_variant	4.00E-12
GCST90255621	16	4892098	PPL	rs1049205	missense_variant	1.00E-20
GCST007039	16	4892098	PPL	rs1049205	missense_variant	9.00E-13
GCST009871	16	4890993	PPL	rs760118	intron_variant	3.00E-12
GCST009871	16	4891046	PPL	rs760117	intron_variant	4.00E-11
GCST90255621	16	68348075	PRMT7	rs2307022	intron_variant	5.00E-18
GCST009004	16	68348075	PRMT7	rs2307022	intron_variant	8.00E-15
GCST009001	16	68348075	PRMT7	rs2307022	intron_variant	8.00E-15
GCST90179150	16	68348075	PRMT7	rs2307022	intron_variant	2.00E-14
GCST007039	16	68348075	PRMT7	rs2307022	intron_variant	8.00E-11
GCST008129	16	28933075	RABEP2	rs2904880	missense_variant	1.00E-35
GCST009121	16	28925938	RABEP2	rs4072402	intron_variant	3.00E-28
GCST009107	16	28925938	RABEP2	rs4072402	intron_variant	6.00E-12
GCST90018947	16	24567137	RBBP6	rs7195386	splice_region_variant	2.00E-14
GCST90179150	16	24567137	RBBP6	rs7195386	splice_region_variant	7.00E-14
GCST009004	16	24529485	RBBP6	rs2342892	intergenic_variant	1.00E-13
GCST007039	16	24567137	RBBP6	rs7195386	splice_region_variant	4.00E-13
GCST009871	16	24529485	RBBP6	rs2342892	intergenic_variant	6.00E-12
GCST90255621	16	24567137	RBBP6	rs7195386	splice_region_variant	8.00E-12
GCST90255621	16	6651399	RBFOX1	rs10083803	intron_variant	1.00E-13
GCST90255621	16	7014701	RBFOX1	rs9926160	intron_variant	9.00E-11
GCST90255621	16	49028679	RNU6-257P - MTND4LP25	rs2080454	intergenic_variant	4.00E-19
GCST009004	16	48977338	RNU6-257P - MTND4LP25	rs6500208	intergenic_variant	3.00E-13
GCST90179150	16	48977338	RNU6-257P - MTND4LP25	rs6500208	intergenic_variant	4.00E-13
GCST005951	16	49028679	RNU6-257P - MTND4LP25	rs2080454	intergenic_variant	3.00E-12
GCST009001	16	49023277	RNU6-257P - MTND4LP25	rs2908889	regulatory_region_variant	7.00E-12
GCST004904	16	49028679	RNU6-257P - MTND4LP25	rs2080454	intergenic_variant	8.00E-11
GCST90255621	16	53678223	RPGRIP1L	rs1477199	intron_variant	1.00E-28
GCST90179150	16	53678223	RPGRIP1L	rs1477199	intron_variant	3.00E-21
GCST009871	16	53603976	RPGRIP1L	rs16952362	intron_variant	7.00E-17
GCST007039	16	53603976	RPGRIP1L	rs16952362	intron_variant	2.00E-16
GCST90179150	16	53580834	RPGRIP1L	rs12928335	intergenic_variant	9.00E-15
GCST009871	16	53565725	RPGRIP1L	rs12931642	intergenic_variant	3.00E-14
GCST008129	16	53605526	RPGRIP1L	rs3213758	missense_variant	5.00E-11
GCST90255621	16	9318365	RPL21P119 - LINC02177	rs249293	intergenic_variant	1.00E-11
GCST009004	16	9318365	RPL21P119 - LINC02177	rs249293	intergenic_variant	3.00E-11
GCST009871	16	28335819	SBK1	rs2726036	intergenic_variant	2.00E-38
GCST90255621	16	28290166	SBK1	rs9939450	intron_variant	6.00E-22

GCST005951	16	28322090	SBK1	rs2650492	3_prime_UTR_variant	7.00E-15
GCST006368	16	28322090	SBK1	rs2650492	3_prime_UTR_variant	2.00E-12
GCST90255621	16	28874338	SH2B1	rs7359397	regulatory_region_variant	8.00E-88
GCST009004	16	28871920	SH2B1	rs7498665	missense_variant	1.00E-66
GCST009003	16	28871920	SH2B1	rs7498665	missense_variant	1.00E-66
GCST007039	16	28871920	SH2B1	rs7498665	missense_variant	1.00E-40
GCST009871	16	28871920	SH2B1	rs7498665	missense_variant	4.00E-40
GCST000830	16	28874338	SH2B1	rs7359397	regulatory_region_variant	2.00E-20
GCST004558	16	28862077	SH2B1	rs4788102	intron_variant	1.00E-16
GCST004559	16	28862077	SH2B1	rs4788102	intron_variant	8.00E-15
GCST000298	16	28871920	SH2B1	rs7498665	missense_variant	5.00E-11
GCST009871	16	69251607	SNTB2	rs192042440	intron_variant	5.00E-13
GCST007039	16	69251607	SNTB2	rs192042440	intron_variant	1.00E-12
GCST90179150	16	69251607	SNTB2	rs192042440	intron_variant	4.00E-12
GCST007039	16	2747160	SRRM2	rs117549504	intron_variant	4.00E-12
GCST009871	16	2747160	SRRM2	rs117549504	intron_variant	5.00E-12
GCST90255621	16	2766626	SRRM2	rs117133016	missense_variant	3.00E-11
GCST90094398	16	2766626	SRRM2	rs117133016	missense_variant	3.00E-11
GCST007039	16	30999862	STX1B	rs4889606	intron_variant	4.00E-26
GCST009121	16	31000500	STX1B	rs12716979	intron_variant	7.00E-24
GCST009107	16	31000500	STX1B	rs12716979	intron_variant	1.00E-16
GCST90255621	16	29983601	TAOK2	rs3814883	synonymous_variant	5.00E-50
GCST90271770	16	29983601	TAOK2	rs3814883	synonymous_variant	1.00E-40
GCST90179150	16	29983601	TAOK2	rs3814883	synonymous_variant	2.00E-38
GCST007039	16	29983601	TAOK2	rs3814883	synonymous_variant	7.00E-35
GCST008129	16	29986879	TAOK2	rs4077410	synonymous_variant	2.00E-21
GCST90179150	16	30039284	TLCD3B	rs147553474	intron_variant	1.00E-12
GCST009871	16	30039284	TLCD3B	rs147553474	intron_variant	6.00E-11
GCST90018947	16	29943333	TMEM219	rs4402589	intron_variant	6.00E-34
GCST009871	16	29943333	TMEM219	rs4402589	intron_variant	2.00E-33
GCST006368	16	29956694	TMEM219	rs7204797	intron_variant	6.00E-11
GCST009871	16	29950023	TMEM219	rs143504748	intron_variant	7.00E-11
GCST90255621	16	24777324	TNRC6A	rs11639856	missense_variant	3.00E-18
GCST009004	16	24795099	TNRC6A	rs7186893	intron_variant	1.00E-13
GCST009003	16	24795099	TNRC6A	rs7186893	intron_variant	1.00E-13
GCST90179150	16	24795099	TNRC6A	rs7186893	intron_variant	2.00E-13
GCST009871	16	24773154	TNRC6A	rs7204281	intron_variant	5.00E-12
GCST007039	16	24773154	TNRC6A	rs7204281	intron_variant	5.00E-12
GCST009004	16	4880099	UBN1	rs1876359	missense_variant	3.00E-14
GCST90179150	16	4880099	UBN1	rs1876359	missense_variant	3.00E-13
GCST90255621	16	72962263	ZFHX3	rs756717	intron_variant	2.00E-24
GCST90255621	16	73063130	ZFHX3	rs7498798	intron_variant	1.00E-17
GCST90018947	16	72962263	ZFHX3	rs756717	intron_variant	4.00E-15
GCST90179150	16	72962263	ZFHX3	rs756717	intron_variant	1.00E-14
GCST009871	16	72962263	ZFHX3	rs756717	intron_variant	5.00E-13
GCST007039	16	72962263	ZFHX3	rs756717	intron_variant	5.00E-13
GCST90255621	16	72959807	ZFHX3	rs62053191	synonymous_variant	4.00E-12
GCST009871	16	72959807	ZFHX3	rs62053191	synonymous_variant	3.00E-11
GCST004045	16	72963084	ZFHX3	rs7190256	intron_variant	3.00E-11
GCST007039	17	33146437	ASH1L	rs73982435	intron_variant	4.00E-12
GCST90255621	17	33133881	ASIC2	rs7211567	intron_variant	4.00E-12

GCST90179150	17	33148527	ASIC2	rs3930349	intron_variant	5.00E-12
GCST009004	17	33148527	ASIC2	rs3930349	intron_variant	8.00E-12
GCST009871	17	33146437	ASIC2	rs73982435	intron_variant	3.00E-11
GCST90255621	17	44203914	ATXN2L	rs4473241	intron_variant	1.00E-17
GCST90018947	17	44203914	ATXN7L3	rs4473241	intron_variant	9.00E-11
GCST009871	17	81110567	BAG6	rs3935190	3_prime_UTR_variant	4.00E-17
GCST007039	17	81110567	BAIAP2	rs3935190	3_prime_UTR_variant	5.00E-17
GCST009004	17	81110567	BAIAP2	rs3935190	3_prime_UTR_variant	9.00E-16
GCST90179150	17	81110567	BAIAP2	rs3935190	3_prime_UTR_variant	1.00E-15
GCST009003	17	81113737	BAIAP2	rs4076427	3_prime_UTR_variant	4.00E-15
GCST90255621	17	81076907	BAIAP2	rs9908480	intron_variant	3.00E-14
GCST90255621	17	67873957	BNIPL	rs12602912	intron_variant	2.00E-23
GCST90018947	17	67986827	BPTF	rs62086046	intergenic_variant	4.00E-18
GCST90179150	17	67873957	BPTF	rs12602912	intron_variant	2.00E-15
GCST007039	17	67835900	BPTF	rs7218014	intron_variant	7.00E-15
GCST009871	17	67981016	BPTF	rs4790941	intron_variant	1.00E-13
GCST009004	17	67951524	BPTF	rs12449442	intron_variant	2.00E-13
GCST009003	17	67951524	BPTF	rs12449442	intron_variant	2.00E-13
GCST004904	17	67873957	BPTF	rs12602912	intron_variant	6.00E-12
GCST008129	17	67991933	C12orf42	rs9891146	missense_variant	1.00E-11
GCST90255621	17	70347398	CALM2P1	rs312750	intron_variant	4.00E-12
GCST90255621	17	70221719	CALM2P1	rs236531	intergenic_variant	4.00E-11
GCST90255621	17	4898592	CHRNE	rs12936083	3_prime_UTR_variant	4.00E-17
GCST90179150	17	4903803	CHRNE	rs3966782	intron_variant	7.00E-11
GCST90255621	17	48043405	COPZ2	rs10491182	regulatory_region_variant	3.00E-13
GCST90255621	17	48004549	COPZ2	rs2905855	intergenic_variant	4.00E-11
GCST90255621	17	63589325	DCAF7	rs72845886	3_prime_UTR_variant	5.00E-19
GCST009871	17	63589646	DCAF7	rs72845888	3_prime_UTR_variant	7.00E-12
GCST005951	17	63568639	DCAF7	rs17631394	intron_variant	3.00E-11
GCST007039	17	63589646	DCAF7	rs72845888	3_prime_UTR_variant	5.00E-11
GCST90104629	17	7219058	DLG4	rs739669	5_prime_UTR_variant	5.00E-17
GCST90255621	17	7197973	DLG4	rs3826408	intron_variant	7.00E-11
GCST90179150	17	36558947	GGNBP2	rs12150665	intron_variant	2.00E-25
GCST005951	17	36558947	GGNBP2	rs12150665	intron_variant	3.00E-13
GCST006368	17	36558525	GGNBP2	rs28573110	intron_variant	3.00E-12
GCST004904	17	36558947	GGNBP2	rs12150665	intron_variant	5.00E-11
GCST90255621	17	48592068	HOXB-AS3, HOXB5, HOXB3	rs9299	3_prime_UTR_variant	2.00E-18
GCST90179150	17	48592068	HOXB-AS3, HOXB5, HOXB3	rs9299	3_prime_UTR_variant	9.00E-11
GCST90255621	17	49013423	IGF2BP1	rs11079849	intron_variant	5.00E-30
GCST009004	17	49063432	IGF2BP1	rs11655587	intergenic_variant	7.00E-26
GCST90179150	17	49063432	IGF2BP1	rs11655587	intergenic_variant	3.00E-25
GCST007039	17	49013423	IGF2BP1	rs11079849	intron_variant	1.00E-24
GCST009871	17	49013423	IGF2BP1	rs11079849	intron_variant	3.00E-24
GCST009003	17	49014058	IGF2BP1	rs9906944	intron_variant	9.00E-23
GCST90018947	17	49063432	IGF2BP1	rs11655587	intergenic_variant	3.00E-18
GCST90255621	17	48992888	IGF2BP1	rs67645835	intergenic_variant	9.00E-13
GCST90179150	17	49067202	IGF2BP1	rs12939237	regulatory_region_variant	9.00E-11
GCST90255621	17	21358248	MAP2K3	rs4986044	regulatory_region_variant	5.00E-28
GCST90179150	17	21358248	MAP2K3	rs4986044	regulatory_region_variant	2.00E-27
GCST009004	17	21361084	MAP2K3	rs1320251	regulatory_region_variant	6.00E-25
GCST009003	17	21361084	MAP2K3	rs1320251	regulatory_region_variant	6.00E-25

GCST009001	17	21360381	MAP2K3	rs4986045	regulatory_region_variant	9.00E-25
GCST007039	17	21361084	MAP2K3	rs1320251	regulatory_region_variant	6.00E-21
GCST009871	17	21361084	MAP2K3	rs1320251	regulatory_region_variant	3.00E-20
GCST90018947	17	21347781	MAP2K3	rs55678940	regulatory_region_variant	2.00E-19
GCST009004	17	63651521	MAP3K3	rs8075273	intron_variant	5.00E-14
GCST009003	17	63651521	MAP3K3	rs8075273	intron_variant	5.00E-14
GCST90179150	17	63651521	MAP3K3	rs8075273	intron_variant	1.00E-13
GCST009001	17	36603030	MRM1	rs12936319	intron_variant	1.00E-22
GCST009004	17	36700930	MRM1	rs6607337	intergenic_variant	3.00E-11
GCST90255621	17	36498436	MYO19	rs2306590	missense_variant	1.00E-35
GCST009003	17	36510707	MYO19	rs2306593	intron_variant	2.00E-22
GCST008129	17	36498436	MYO19	rs2306590	missense_variant	2.00E-18
GCST009871	17	36510707	MYO19	rs2306593	intron_variant	3.00E-17
GCST007039	17	36510707	MYO19	rs2306593	intron_variant	4.00E-17
GCST009871	17	80539301	NPTX1	rs7222801	regulatory_region_variant	1.00E-14
GCST90255621	17	80474840	NPTX1	rs12600720	non_coding_transcript_exon_	2.00E-12
GCST009866	17	39994239	PSMD3	rs3826331	non_coding_transcript_exon_	1.00E-12
GCST90255621	17	40004501	PSMD3	rs8070454	intergenic_variant	3.00E-12
GCST90255621	17	5379957	RABEP1	rs1000940	intron_variant	7.00E-20
GCST009004	17	5379957	RABEP1	rs1000940	intron_variant	8.00E-18
GCST009001	17	5379957	RABEP1	rs1000940	intron_variant	8.00E-18
GCST009003	17	5377145	RABEP1	rs3026101	missense_variant	1.00E-17
GCST90179150	17	5379957	RABEP1	rs1000940	intron_variant	3.00E-17
GCST006368	17	5377145	RABEP1	rs3026101	missense_variant	3.00E-13
GCST007039	17	5384859	RABEP1	rs10792	3_prime_UTR_variant	2.00E-12
GCST009871	17	5384859	RABEP1	rs10792	3_prime_UTR_variant	3.00E-12
GCST90255621	17	80641771	RPTOR	rs12940622	intron_variant	2.00E-30
GCST009004	17	80637924	RPTOR	rs12939549	intron_variant	4.00E-28
GCST009001	17	80637924	RPTOR	rs12939549	intron_variant	4.00E-28
GCST90179150	17	80637924	RPTOR	rs12939549	intron_variant	5.00E-28
GCST009003	17	80636743	RPTOR	rs9910745	intron_variant	3.00E-26
GCST007039	17	80783826	RPTOR	rs11150745	intron_variant	3.00E-24
GCST90255621	17	80666710	RPTOR	rs4889782	intron_variant	4.00E-22
GCST009871	17	80627018	RPTOR	rs8079537	intron_variant	2.00E-19
GCST90018947	17	80783826	RPTOR	rs11150745	intron_variant	1.00E-15
GCST90179150	17	80666710	RPTOR	rs4889782	intron_variant	3.00E-13
GCST009871	17	80654176	RPTOR	rs35675302	intron_variant	1.00E-11
GCST006368	17	80637924	RPTOR	rs12939549	intron_variant	3.00E-11
GCST009871	17	80793475	RPTOR	rs12601434	intron_variant	5.00E-11
GCST009871	17	80666756	RPTOR	rs62069691	intron_variant	5.00E-11
GCST005951	17	80637924	RPTOR	rs12939549	intron_variant	7.00E-11
GCST90255621	17	1956248	RTN4RL1	rs4790849	intron_variant	9.00E-24
GCST009004	17	1939895	RTN4RL1	rs3923783	intron_variant	4.00E-23
GCST009001	17	1939895	RTN4RL1	rs3923783	intron_variant	4.00E-23
GCST90179150	17	1939895	RTN4RL1	rs3923783	intron_variant	5.00E-23
GCST009871	17	1921011	RTN4RL1	rs4790292	intergenic_variant	4.00E-22
GCST007039	17	1943537	RTN4RL1	rs4516268	intron_variant	4.00E-22
GCST009003	17	1921011	RTN4RL1	rs4790292	intergenic_variant	7.00E-20
GCST90018947	17	1933875	RTN4RL1	rs111279065	intergenic_variant	5.00E-19
GCST90267268	17	1916786	RTN4RL1	rs12602230	intergenic_variant	2.00E-13
GCST009871	17	1946368	RTN4RL1	rs75848890	intron_variant	2.00E-12

GCST90179150	17	48174984	SKAP1	rs208015	intron_variant	1.00E-26
GCST009001	17	48174984	SKAP1	rs208015	intron_variant	7.00E-26
GCST90255621	17	48174984	SKAP1	rs208015	intron_variant	1.00E-25
GCST007039	17	48193244	SKAP1	rs113866544	intron_variant	1.00E-20
GCST009871	17	48193244	SKAP1	rs113866544	intron_variant	2.00E-20
GCST004904	17	48215561	SKAP1	rs6504108	intron_variant	2.00E-11
GCST009871	17	30219896	SLC6A4	rs2020942	intron_variant	4.00E-11
GCST007039	17	30219896	SLC6A4	rs2020942	intron_variant	4.00E-11
GCST90271770	17	2232771	SMG6	rs7217226	intron_variant	2.00E-22
GCST90255621	17	2240166	SMG6	rs10852932	5_prime_UTR_variant	3.00E-20
GCST009004	17	2232771	SMG6	rs7217226	intron_variant	9.00E-16
GCST009001	17	2232771	SMG6	rs7217226	intron_variant	9.00E-16
GCST90179150	17	2232771	SMG6	rs7217226	intron_variant	9.00E-16
GCST90255621	17	2230277	SMG6	rs28611065	intron_variant	1.00E-14
GCST009871	17	2230277	SMG6	rs28611065	intron_variant	1.00E-13
GCST007039	17	2229911	SMG6	rs9896535	intron_variant	8.00E-12
GCST009871	17	2229911	SMG6	rs9896535	intron_variant	1.00E-11
GCST004904	17	2214651	SMG6	rs2281727	intron_variant	6.00E-11
GCST90255621	17	29747545	SSH2	rs1038088	intron_variant	7.00E-14
GCST90018947	17	29743413	SSH2	rs11395406	intron_variant	2.00E-13
GCST009004	17	29747545	SSH2	rs1038088	intron_variant	1.00E-12
GCST90179150	17	29747545	SSH2	rs1038088	intron_variant	1.00E-12
GCST90255621	17	61419916	TBX4	rs757608	regulatory_region_variant	5.00E-13
GCST90271770	17	61419916	TBX4	rs757608	regulatory_region_variant	2.00E-11
GCST90255621	17	16040596	TTC19, NCOR1	rs1075901	intron_variant	4.00E-14
GCST009004	17	16040596	TTC19, NCOR1	rs1075901	intron_variant	4.00E-13
GCST009003	17	16040596	TTC19, NCOR1	rs1075901	intron_variant	4.00E-13
GCST90179150	17	16040596	TTC19, NCOR1	rs1075901	intron_variant	7.00E-13
GCST007039	17	16040596	TTC19, NCOR1	rs1075901	intron_variant	4.00E-12
GCST009871	17	16039830	TTC19, NCOR1	rs4792716	intron_variant	2.00E-11
GCST009868	17	75829382	UNC13D	rs111365807	intron_variant	7.00E-12
GCST009866	17	75829382	UNC13D	rs111365807	intron_variant	1.00E-11
GCST90255621	18	1839600	AGBL4	rs8097672	intron_variant	6.00E-23
GCST90179150	18	1839338	AIDAP3	rs9955276	intron_variant	3.00E-18
GCST009004	18	1840657	AIDAP3	rs7238896	intron_variant	2.00E-15
GCST009003	18	1840657	AIDAP3	rs7238896	intron_variant	2.00E-15
GCST009871	18	1840657	AIDAP3	rs7238896	intron_variant	7.00E-15
GCST007039	18	1839338	AIDAP3	rs9955276	intron_variant	5.00E-14
GCST90018947	18	1840657	AIDAP3	rs7238896	intron_variant	7.00E-13
GCST006368	18	33671124	ASTN2	rs11081818	intron_variant	1.00E-15
GCST90255621	18	33671312	ASXL3	rs1941697	intron_variant	7.00E-15
GCST009004	18	33671312	ASXL3	rs1941697	intron_variant	8.00E-13
GCST90179150	18	33671312	ASXL3	rs1941697	intron_variant	3.00E-12
GCST007039	18	33668359	ASXL3	rs6507054	intron_variant	8.00E-12
GCST90018947	18	33671312	ASXL3	rs1941697	intron_variant	3.00E-11
GCST009871	18	33643812	ASXL3	rs1941706	intron_variant	5.00E-11
GCST90255621	18	63178651	BCL11B	rs12454712	intron_variant	8.00E-22
GCST90179150	18	63178651	BCL2	rs12454712	intron_variant	8.00E-15
GCST90018947	18	63178651	BCL2	rs12454712	intron_variant	9.00E-15
GCST009004	18	63178651	BCL2	rs12454712	intron_variant	1.00E-14
GCST009001	18	63178651	BCL2	rs12454712	intron_variant	1.00E-14

GCST007039	18	63178651	BCL2	rs12454712	intron_variant	2.00E-12
GCST90255621	18	63095879	BCL2	rs11152368	intergenic_variant	2.00E-12
GCST009871	18	63178651	BCL2	rs12454712	intron_variant	1.00E-11
GCST90179150	18	63056554	BCL2	rs1031670	intergenic_variant	2.00E-11
GCST004904	18	63178651	BCL2	rs12454712	intron_variant	3.00E-11
GCST90255621	18	65706526	CDH7	rs7235205	intergenic_variant	1.00E-17
GCST90179150	18	65630436	CDH7	rs2012927	intron_variant	7.00E-17
GCST009004	18	65618547	CDH7	rs10871589	intron_variant	1.00E-15
GCST009003	18	65618547	CDH7	rs10871589	intron_variant	1.00E-15
GCST009871	18	65615756	CDH7	rs1373349	intron_variant	6.00E-14
GCST007039	18	65630353	CDH7	rs1344374	intron_variant	8.00E-14
GCST009001	18	65794402	CDH7	rs17783165	intron_variant	3.00E-13
GCST90018947	18	65615756	CDH7	rs1373349	intron_variant	6.00E-13
GCST90018947	18	53076643	DCC	rs11463289	intron_variant	1.00E-11
GCST90255621	18	52915447	DCC	rs9964756	intron_variant	9.00E-11
GCST009871	18	60972314	HMGN1P31	rs193239997	intergenic_variant	9.00E-25
GCST90255621	18	60787403	HMGN1P31	rs77627291	intron_variant	2.00E-17
GCST90179150	18	61071705	HMGN1P31	rs1426284	intergenic_variant	3.00E-14
GCST90255621	18	71557242	LINC01541	rs8089514	intron_variant	4.00E-17
GCST007039	18	71557242	LINC01541	rs8089514	intron_variant	6.00E-11
GCST90255621	18	75786573	LINC01898 - LINC01893	rs11150911	intergenic_variant	7.00E-15
GCST90179150	18	75786573	LINC01898 - LINC01893	rs11150911	intergenic_variant	7.00E-11
GCST90255621	18	24722019	LINC01915	rs1680339	intergenic_variant	3.00E-17
GCST90255621	18	24557355	LINC01915	rs16940823	intron_variant	9.00E-14
GCST90179150	18	24557355	LINC01915	rs16940823	intron_variant	1.00E-12
GCST009004	18	24557355	LINC01915	rs16940823	intron_variant	2.00E-12
GCST009003	18	24574271	LINC01915	rs11660335	intron_variant	6.00E-12
GCST90255621	18	60172536	MC4R	rs571312	intergenic_variant	2.00E-213
GCST009004	18	60161902	MC4R	rs6567160	intergenic_variant	8.00E-184
GCST009003	18	60161902	MC4R	rs6567160	intergenic_variant	8.00E-184
GCST009001	18	60161902	MC4R	rs6567160	intergenic_variant	8.00E-184
GCST90179150	18	60161902	MC4R	rs6567160	intergenic_variant	5.00E-180
GCST90090978	18	60184530	MC4R	rs10871777	intergenic_variant	2.00E-152
GCST009871	18	60161902	MC4R	rs6567160	intergenic_variant	7.00E-126
GCST007039	18	60161902	MC4R	rs6567160	intergenic_variant	1.00E-125
GCST90018947	18	60161902	MC4R	rs6567160	intergenic_variant	3.00E-118
GCST90255621	18	60275566	MC4R	rs9956279	intergenic_variant	8.00E-100
GCST90090977	18	60184530	MC4R	rs10871777	intergenic_variant	3.00E-94
GCST004904	18	60161902	MC4R	rs6567160	intergenic_variant	2.00E-84
GCST009121	18	60184530	MC4R	rs10871777	intergenic_variant	3.00E-81
GCST90179150	18	60275566	MC4R	rs9956279	intergenic_variant	4.00E-81
GCST90255621	18	60372043	MC4R	rs2229616	missense_variant	2.00E-78
GCST90103755	18	60161902	MC4R	rs6567160	intergenic_variant	3.00E-75
GCST006368	18	60161902	MC4R	rs6567160	intergenic_variant	7.00E-70
GCST90179150	18	60372043	MC4R	rs2229616	missense_variant	6.00E-69
GCST005951	18	60161902	MC4R	rs6567160	intergenic_variant	9.00E-66
GCST004519	18	60161902	MC4R	rs6567160	intergenic_variant	2.00E-64
GCST009871	18	60278047	MC4R	rs7227810	regulatory_region_variant	3.00E-62
GCST005950	18	60205756	MC4R	rs12955983	intergenic_variant	9.00E-60
GCST002783	18	60161902	MC4R	rs6567160	intergenic_variant	7.00E-59
GCST005951	18	60205756	MC4R	rs12955983	intergenic_variant	2.00E-58

GCST007039	18	60373556	MC4R	rs34974495	intron_variant	5.00E-56
GCST004497	18	60161902	MC4R	rs6567160	intergenic_variant	4.00E-55
GCST009871	18	60373556	MC4R	rs34974495	intron_variant	7.00E-55
GCST004495	18	60161902	MC4R	rs6567160	intergenic_variant	3.00E-54
GCST002783	18	60161902	MC4R	rs6567160	intergenic_variant	4.00E-53
GCST004558	18	60208994	MC4R	rs11663816	intergenic_variant	2.00E-47
GCST004557	18	60192330	MC4R	rs12969709	intergenic_variant	1.00E-45
GCST90255621	18	60382423	MC4R	rs17066856	intron_variant	6.00E-44
GCST007241	18	60165624	MC4R	rs35614134	intergenic_variant	2.00E-43
GCST004499	18	60161902	MC4R	rs6567160	intergenic_variant	9.00E-43
GCST000830	18	60172536	MC4R	rs571312	intergenic_variant	6.00E-42
GCST004558	18	60192330	MC4R	rs12969709	intergenic_variant	1.00E-41
GCST005953	18	60205756	MC4R	rs12955983	intergenic_variant	2.00E-41
GCST004557	18	60192330	MC4R	rs12969709	intergenic_variant	5.00E-41
GCST90179150	18	60382423	MC4R	rs17066856	intron_variant	2.00E-37
GCST004559	18	60208994	MC4R	rs11663816	intergenic_variant	4.00E-37
GCST004904	18	60161902	MC4R	rs6567160	intergenic_variant	8.00E-37
GCST90018727	18	60161902	MC4R	rs6567160	intergenic_variant	2.00E-36
GCST004559	18	60192330	MC4R	rs12969709	intergenic_variant	7.00E-35
GCST002783	18	60161902	MC4R	rs6567160	intergenic_variant	5.00E-34
GCST004497	18	60161902	MC4R	rs6567160	intergenic_variant	1.00E-31
GCST004495	18	60161902	MC4R	rs6567160	intergenic_variant	1.00E-31
GCST006368	18	60291011	MC4R	rs9944545	intergenic_variant	9.00E-31
GCST002783	18	60161902	MC4R	rs6567160	intergenic_variant	5.00E-30
GCST90255621	18	60381644	MC4R	rs78138914	intron_variant	6.00E-29
GCST006802	18	60161902	MC4R	rs6567160	intergenic_variant	1.00E-28
GCST004495	18	60161902	MC4R	rs6567160	intergenic_variant	1.00E-28
GCST004497	18	60161902	MC4R	rs6567160	intergenic_variant	4.00E-28
GCST004558	18	60192330	MC4R	rs12969709	intergenic_variant	7.00E-28
GCST004557	18	60192330	MC4R	rs12969709	intergenic_variant	2.00E-27
GCST009871	18	60367971	MC4R	rs1943226	intron_variant	1.00E-26
GCST90179150	18	60202357	MC4R	rs9947301	intergenic_variant	1.00E-25
GCST004557	18	60172536	MC4R	rs571312	intergenic_variant	1.00E-25
GCST004557	18	60192330	MC4R	rs12969709	intergenic_variant	1.00E-25
GCST004558	18	60192330	MC4R	rs12969709	intergenic_variant	2.00E-25
GCST004499	18	60161902	MC4R	rs6567160	intergenic_variant	4.00E-25
GCST009871	18	60300422	MC4R	rs75941710	intron_variant	1.00E-24
GCST90255621	18	60187234	MC4R	rs8082946	intergenic_variant	1.00E-24
GCST90255621	18	60343663	MC4R	rs79131845	intron_variant	3.00E-24
GCST90179150	18	60281167	MC4R	rs80116344	intergenic_variant	5.00E-24
GCST004558	18	60172536	MC4R	rs571312	intergenic_variant	5.00E-24
GCST005952	18	60205756	MC4R	rs12955983	intergenic_variant	2.00E-23
GCST004499	18	60161902	MC4R	rs6567160	intergenic_variant	2.00E-23
GCST004559	18	60192330	MC4R	rs12969709	intergenic_variant	5.00E-22
GCST004557	18	60172536	MC4R	rs571312	intergenic_variant	7.00E-22
GCST90255621	18	60474274	MC4R	rs599148	intron_variant	2.00E-21
GCST007240	18	60181298	MC4R	rs2168711	intergenic_variant	2.00E-21
GCST009871	18	60343663	MC4R	rs79131845	intron_variant	2.00E-21
GCST001955	18	60191596	MC4R	rs8089364	intergenic_variant	4.00E-21
GCST90179150	18	60186911	MC4R	rs11152214	intergenic_variant	5.00E-21
GCST004559	18	60192330	MC4R	rs12969709	intergenic_variant	5.00E-21

GCST004559	18	60208994	MC4R	rs11663816	intergenic_variant	3.00E-20
GCST009107	18	60184530	MC4R	rs10871777	intergenic_variant	2.00E-19
GCST90255621	18	60196554	MC4R	rs35087079	regulatory_region_variant	5.00E-19
GCST009871	18	60282622	MC4R	rs12962523	intergenic_variant	6.00E-19
GCST90255622	18	60161902	MC4R	rs6567160	intergenic_variant	6.00E-19
GCST002227	18	60161902	MC4R	rs6567160	intergenic_variant	8.00E-19
GCST90267268	18	60414949	MC4R	rs9946862	intron_variant	1.00E-18
GCST009764	18	60161902	MC4R	rs6567160	intergenic_variant	2.00E-18
GCST000298	18	60183864	MC4R	rs17782313	intergenic_variant	5.00E-18
GCST90239604	18	60161902	MC4R	rs6567160	intergenic_variant	5.00E-18
GCST90131907	18	60160805	MC4R	rs536783	intron_variant	9.00E-18
GCST90179150	18	60276192	MC4R	rs1563602	intergenic_variant	1.00E-17
GCST009871	18	60187234	MC4R	rs8082946	intergenic_variant	1.00E-17
GCST008158	18	60181790	MC4R	rs12967135	intergenic_variant	1.00E-16
GCST009871	18	60246470	MC4R	rs12963186	intergenic_variant	9.00E-16
GCST90179150	18	60196908	MC4R	rs9319962	non_coding_transcript_exon_	2.00E-15
GCST000185	18	60183864	MC4R	rs17782313	intergenic_variant	3.00E-15
GCST90179150	18	60246470	MC4R	rs12963186	intergenic_variant	7.00E-15
GCST90255621	18	60462418	MC4R	rs4940940	intron_variant	1.00E-14
GCST004498	18	60161902	MC4R	rs6567160	intergenic_variant	2.00E-14
GCST002461	18	60174356	MC4R	rs591166	intergenic_variant	7.00E-14
GCST90179150	18	60433167	MC4R	rs8088123	intron_variant	2.00E-13
GCST90094398	18	60372169	MC4R	rs370479598	missense_variant	2.00E-13
GCST009871	18	60433167	MC4R	rs8088123	intron_variant	3.00E-13
GCST004558	18	60183864	MC4R	rs17782313	intergenic_variant	3.00E-13
GCST009871	18	60513563	MC4R	rs67611440	intron_variant	6.00E-13
GCST000296	18	60217517	MC4R	rs12970134	intergenic_variant	1.00E-12
GCST90255621	18	60513563	MC4R	rs67611440	intron_variant	2.00E-12
GCST004519	18	60161902	MC4R	rs6567160	intergenic_variant	2.00E-12
GCST004560	18	60172536	MC4R	rs571312	intergenic_variant	4.00E-12
GCST009871	18	60438196	MC4R	rs948810	intron_variant	6.00E-12
GCST004560	18	60183864	MC4R	rs17782313	intergenic_variant	2.00E-11
GCST001416	18	60161404	MC4R	rs2331841	intergenic_variant	2.00E-11
GCST008025	18	60161902	MC4R	rs6567160	intergenic_variant	2.00E-11
GCST009871	18	60208134	MC4R	rs76752804	intergenic_variant	4.00E-11
GCST90095034	18	60161902	MC4R	rs6567160	intergenic_variant	4.00E-11
GCST90179150	18	60559879	MRPS5P4 - GAD3P	rs1430387	intergenic_variant	2.00E-12
GCST007039	18	60553938	MRPS5P4 - GAD3P	rs11152236	intergenic_variant	7.00E-12
GCST90255621	18	23523945	NPC1	rs891387	intron_variant	1.00E-41
GCST009004	18	23523945	NPC1	rs891387	intron_variant	9.00E-35
GCST009001	18	23523945	NPC1	rs891387	intron_variant	9.00E-35
GCST009003	18	23524924	NPC1	rs1808579	intron_variant	2.00E-33
GCST90179150	18	23520276	NPC1	rs1367083	synonymous_variant	9.00E-33
GCST007039	18	23510059	NPC1	rs1788808	intron_variant	2.00E-26
GCST009871	18	23539327	NPC1	rs6507720	intron_variant	1.00E-25
GCST90018947	18	23510059	NPC1	rs1788808	intron_variant	2.00E-19
GCST008129	18	23544981	NPC1	rs1788799	missense_variant	1.00E-17
GCST006368	18	23521980	NPC1	rs1788820	intron_variant	4.00E-12
GCST005951	18	23535096	NPC1	rs6507716	intron_variant	4.00E-11
GCST90255621	18	42064283	PIK3C3	rs559231	intron_variant	9.00E-16
GCST009866	18	41911693	PIK3C3	rs8089834	intergenic_variant	1.00E-13

GCST90179150	18	42064283	PIK3C3	rs559231	intron_variant	2.00E-13
GCST007039	18	42064283	PIK3C3	rs559231	intron_variant	1.00E-12
GCST009871	18	42025102	PIK3C3	rs7237783	intron_variant	4.00E-11
GCST90255621	18	54812256	RAB27B	rs8092503	intron_variant	1.00E-20
GCST009004	18	54812256	RAB27B	rs8092503	intron_variant	4.00E-17
GCST009001	18	54832780	RAB27B	rs12454204	intron_variant	5.00E-17
GCST90179150	18	54812256	RAB27B	rs8092503	intron_variant	2.00E-16
GCST90018947	18	54842602	RAB27B	rs58243949	intron_variant	8.00E-14
GCST009871	18	54842602	RAB27B	rs58243949	intron_variant	2.00E-13
GCST007039	18	54842602	RAB27B	rs58243949	intron_variant	2.00E-13
GCST90255621	18	43156625	RIT2	rs1158805	intergenic_variant	1.00E-16
GCST009004	18	43128073	RIT2	rs1356506	non_coding_transcript_exon_	8.00E-15
GCST90179150	18	43158686	RIT2	rs1424394	intergenic_variant	1.00E-14
GCST90018947	18	43121293	RIT2	rs144012557	intron_variant	3.00E-14
GCST009871	18	43164825	RIT2	rs1834144	intergenic_variant	2.00E-13
GCST007039	18	43164825	RIT2	rs1834144	intergenic_variant	8.00E-13
GCST009003	18	43180181	RIT2	rs1518159	intergenic_variant	2.00E-12
GCST90255621	18	60108452	RNU6-567P - RPS3AP49	rs17700028	intergenic_variant	4.00E-27
GCST90255621	18	60055359	RNU6-567P - RPS3AP49	rs4395179	intergenic_variant	1.00E-23
GCST90255621	18	60142512	RNU6-567P - RPS3AP49	rs117758180	intron_variant	1.00E-22
GCST90179150	18	60053105	RNU6-567P - RPS3AP49	rs1573399	regulatory_region_variant	1.00E-21
GCST90255621	18	60020341	RNU6-567P - RPS3AP49	rs1893514	regulatory_region_variant	3.00E-21
GCST90179150	18	60020341	RNU6-567P - RPS3AP49	rs1893514	regulatory_region_variant	1.00E-20
GCST009871	18	60142512	RNU6-567P - RPS3AP49	rs117758180	intron_variant	2.00E-20
GCST90179150	18	60142512	RNU6-567P - RPS3AP49	rs117758180	intron_variant	8.00E-19
GCST009871	18	60097229	RNU6-567P - RPS3AP49	rs9963612	intergenic_variant	1.00E-17
GCST009871	18	60108452	RNU6-567P - RPS3AP49	rs17700028	intergenic_variant	2.00E-17
GCST002021	18	60067625	RNU6-567P - RPS3AP49	rs7234864	regulatory_region_variant	4.00E-17
GCST90267268	18	60053966	RNU6-567P - RPS3AP49	rs72989246	TF_binding_site_variant	8.00E-15
GCST009871	18	60055359	RNU6-567P - RPS3AP49	rs4395179	intergenic_variant	1.00E-13
GCST90255621	18	38584285	RPL12P40 - RN7SKP182	rs11874286	intergenic_variant	3.00E-15
GCST90179150	18	38602476	RPL12P40 - RN7SKP182	rs1365466	intergenic_variant	4.00E-11
GCST90255621	18	59216087	SEC11C - GRP	rs7243357	intergenic_variant	2.00E-25
GCST009004	18	59216087	SEC11C - GRP	rs7243357	intergenic_variant	1.00E-20
GCST009001	18	59216087	SEC11C - GRP	rs7243357	intergenic_variant	1.00E-20
GCST90179150	18	59216087	SEC11C - GRP	rs7243357	intergenic_variant	2.00E-20
GCST009871	18	59211130	SEC11C - GRP	rs1517036	intergenic_variant	7.00E-13
GCST007039	18	59211130	SEC11C - GRP	rs1517036	intergenic_variant	1.00E-12
GCST005951	18	59216087	SEC11C - GRP	rs7243357	intergenic_variant	5.00E-11
GCST90255621	18	45018498	SETBP1	rs954018	intron_variant	1.00E-15
GCST90179150	18	45018498	SETBP1	rs954018	intron_variant	5.00E-13
GCST009004	18	45016031	SETBP1	rs11874040	intron_variant	2.00E-11
GCST007039	18	45015111	SETBP1	rs7233512	intron_variant	4.00E-11
GCST90255621	18	45360534	SLC14A2	rs16978350	intron_variant	1.00E-13
GCST009004	18	45360534	SLC14A2	rs16978350	intron_variant	5.00E-11
GCST90179150	18	45360534	SLC14A2	rs16978350	intron_variant	9.00E-11
GCST90255621	18	43412733	SYT4	rs555267	intergenic_variant	5.00E-15
GCST90179150	18	43412733	SYT4	rs555267	intergenic_variant	3.00E-14
GCST90255621	18	43367426	SYT4	rs624565	intergenic_variant	1.00E-12
GCST009871	18	55668281	TCF4	rs11659764	intron_variant	3.00E-13
GCST007039	18	55668281	TCF4	rs11659764	intron_variant	6.00E-13

GCST90255621	18	48394843	ZBTB7C	rs7239114	intron_variant	2.00E-13
GCST009004	18	48394843	ZBTB7C	rs7239114	intron_variant	6.00E-13
GCST009003	18	48394843	ZBTB7C	rs7239114	intron_variant	6.00E-13
GCST90179150	18	48394843	ZBTB7C	rs7239114	intron_variant	3.00E-12
GCST009871	18	48394843	ZBTB7C	rs7239114	intron_variant	2.00E-11
GCST007039	18	48394843	ZBTB7C	rs7239114	intron_variant	7.00E-11
GCST004046	19	44919689	APOB	rs4420638	regulatory_region_variant	2.00E-25
GCST004045	19	44919689	APOC1	rs4420638	regulatory_region_variant	1.00E-22
GCST005952	19	44919689	APOC1	rs4420638	regulatory_region_variant	9.00E-12
GCST90255621	19	44917947	APOC1	rs150966173	intron_variant	5.00E-11
GCST90255621	19	44906745	APOC1	rs769449	non_coding_transcript_exon_	2.00E-49
GCST90179150	19	44908684	APOE	rs429358	missense_variant	3.00E-36
GCST90255621	19	44908822	APOE	rs7412	missense_variant	2.00E-17
GCST90244565	19	44908684	APOE	rs429358	missense_variant	2.00E-14
GCST90275047	19	44908684	APOE	rs429358	missense_variant	1.00E-11
GCST004046	19	44744370	BIN2P2	rs4803750	intergenic_variant	4.00E-16
GCST004045	19	44744370	BLC3	rs4803750	intergenic_variant	1.00E-13
GCST90255621	19	29805946	CCNE1	rs8102137	regulatory_region_variant	9.00E-34
GCST90179150	19	29781295	CCNE1	rs12462975	intergenic_variant	2.00E-26
GCST009004	19	29781295	CCNE1	rs12462975	intergenic_variant	1.00E-25
GCST009003	19	29781295	CCNE1	rs12462975	intergenic_variant	1.00E-25
GCST009001	19	29795915	CCNE1	rs17513613	regulatory_region_variant	6.00E-25
GCST007039	19	29786822	CCNE1	rs113701136	intergenic_variant	2.00E-24
GCST009871	19	29786822	CCNE1	rs113701136	intergenic_variant	1.00E-23
GCST90018947	19	29809110	CCNE1	rs62104483	intergenic_variant	6.00E-19
GCST90267268	19	29774328	CCNE1	rs12461902	regulatory_region_variant	5.00E-12
GCST90255621	19	18698105	CRTC1	rs7258722	intron_variant	1.00E-40
GCST90179150	19	18701214	CRTC1	rs4808845	intron_variant	1.00E-27
GCST007039	19	18701975	CRTC1	rs8112818	intron_variant	1.00E-24
GCST009004	19	18725864	CRTC1	rs11668500	intron_variant	2.00E-23
GCST009003	19	18725864	CRTC1	rs11668500	intron_variant	2.00E-23
GCST009001	19	18716165	CRTC1	rs10221489	intron_variant	1.00E-22
GCST009871	19	18725864	CRTC1	rs11668500	intron_variant	4.00E-22
GCST90018947	19	18723704	CRTC1	rs10404726	intron_variant	5.00E-18
GCST90267268	19	18695858	CRTC1	rs2003476	intron_variant	4.00E-13
GCST005951	19	18709498	CRTC1	rs757318	intron_variant	5.00E-13
GCST009871	19	18777340	CRTC1	rs78830644	synonymous_variant	6.00E-12
GCST90179150	19	18777340	CRTC1	rs78830644	synonymous_variant	2.00E-11
GCST90255621	19	18777340	CRTC1	rs78830644	synonymous_variant	2.00E-11
GCST009871	19	18698901	CRTC1	rs55935482	intron_variant	3.00E-11
GCST90255621	19	1956036	CSNK1G2	rs4807179	intron_variant	4.00E-13
GCST009004	19	1970022	CSNK1G2	rs2074881	intron_variant	2.00E-12
GCST90255621	19	18389912	GDF15	rs16982345	intergenic_variant	9.00E-12
GCST009871	19	18378272	GDF15	rs10424912	intron_variant	6.00E-11
GCST90255621	19	45678134	GIPR	rs1800437	missense_variant	5.00E-90
GCST90018947	19	45671788	GIPR	rs35560038	intron_variant	4.00E-61
GCST009004	19	45676926	GIPR	rs11672660	intron_variant	7.00E-60
GCST009003	19	45676926	GIPR	rs11672660	intron_variant	7.00E-60
GCST009001	19	45678134	GIPR	rs1800437	missense_variant	3.00E-59
GCST90179150	19	45676926	GIPR	rs11672660	intron_variant	1.00E-58
GCST004904	19	45671788	GIPR	rs35560038	intron_variant	3.00E-52

GCST90018727	19	45671788	GIPR	rs35560038	intron_variant	6.00E-52
GCST008129	19	45678134	GIPR	rs1800437	missense_variant	6.00E-45
GCST007039	19	45679046	GIPR	rs10423928	intron_variant	1.00E-44
GCST009871	19	45679046	GIPR	rs10423928	intron_variant	4.00E-43
GCST004904	19	45669020	GIPR	rs11671664	intron_variant	3.00E-42
GCST009121	19	45675785	GIPR	rs2238691	intron_variant	2.00E-32
GCST90255621	19	45677718	GIPR	rs143430880	missense_variant	2.00E-18
GCST005951	19	45676926	GIPR	rs11672660	intron_variant	3.00E-16
GCST90255621	19	45665409	GIPR	rs12611237	intergenic_variant	3.00E-16
GCST009107	19	45675785	GIPR	rs2238691	intron_variant	3.00E-15
GCST001415	19	45669020	GIPR	rs11671664	intron_variant	6.00E-14
GCST001416	19	45669020	GIPR	rs11671664	intron_variant	7.00E-14
GCST90267268	19	45680328	GIPR	rs61373376	intron_variant	1.00E-13
GCST002461	19	45669020	GIPR	rs11671664	intron_variant	3.00E-12
GCST90179150	19	45659141	GIPR	rs12976464	intergenic_variant	2.00E-11
GCST90255621	19	45655215	GIPR	rs12972158	intergenic_variant	8.00E-11
GCST90179150	19	19260108	HAPLN4	rs4808934	intron_variant	4.00E-15
GCST007039	19	19262159	HAPLN4	rs10403273	intron_variant	2.00E-13
GCST90255621	19	33818627	KCTD15	rs29941	intergenic_variant	2.00E-21
GCST90179150	19	33820576	KCTD15	rs29938	intergenic_variant	9.00E-21
GCST009004	19	33820576	KCTD15	rs29938	intergenic_variant	4.00E-20
GCST009001	19	33820576	KCTD15	rs29938	intergenic_variant	4.00E-20
GCST009003	19	33819895	KCTD15	rs29939	intergenic_variant	1.00E-18
GCST005951	19	33820576	KCTD15	rs29938	intergenic_variant	4.00E-14
GCST007039	19	33825800	KCTD15	rs7256653	intergenic_variant	6.00E-14
GCST009871	19	33825800	KCTD15	rs7256653	intergenic_variant	9.00E-13
GCST000296	19	33818627	KCTD15	rs29941	intergenic_variant	7.00E-12
GCST90018947	19	33819129	KCTD15	rs29940	intergenic_variant	1.00E-11
GCST90255621	19	12885926	KLF1	rs2072597	missense_variant	3.00E-13
GCST007039	19	12885926	KLF1	rs2072597	missense_variant	4.00E-11
GCST009871	19	12883326	KLF1	rs12609744	intron_variant	5.00E-11
GCST90179150	19	1865902	KLF16	rs12981256	intergenic_variant	2.00E-18
GCST009004	19	1852495	KLF16	rs3746038	3_prime_UTR_variant	3.00E-16
GCST009003	19	1852495	KLF16	rs3746038	3_prime_UTR_variant	3.00E-16
GCST007039	19	1866116	KLF16	rs12974458	intergenic_variant	2.00E-14
GCST009871	19	1854936	KLF16	rs4806812	intron_variant	7.00E-12
GCST90255621	19	51278201	LINC01872	rs148108087	intron_variant	4.00E-15
GCST009004	19	51278201	LINC01872	rs148108087	intron_variant	2.00E-11
GCST007039	19	51278201	LINC01872	rs148108087	intron_variant	2.00E-11
GCST009871	19	51278201	LINC01872	rs148108087	intron_variant	3.00E-11
GCST90179150	19	51278201	LINC01872	rs148108087	intron_variant	7.00E-11
GCST90255621	19	18144549	MAST3	rs8108738	missense_variant	2.00E-22
GCST90179150	19	18104437	MAST3	rs273504	intron_variant	2.00E-19
GCST009004	19	18106337	MAST3	rs273505	intron_variant	3.00E-19
GCST009001	19	18106337	MAST3	rs273505	intron_variant	3.00E-19
GCST009003	19	18104437	MAST3	rs273504	intron_variant	4.00E-19
GCST009871	19	18106337	MAST3	rs273505	intron_variant	1.00E-18
GCST007039	19	18106337	MAST3	rs273505	intron_variant	1.00E-18
GCST009004	19	44888997	NECTIN2	rs6857	3_prime_UTR_variant	6.00E-26
GCST009003	19	44888997	NECTIN2	rs6857	3_prime_UTR_variant	6.00E-26
GCST007039	19	44888997	NECTIN2	rs6857	3_prime_UTR_variant	3.00E-23

GCST009871	19	44888997	NECTIN2	rs6857	3_prime_UTR_variant	7.00E-23
GCST009871	19	47042461	NPAS1	rs4577206	intron_variant	2.00E-16
GCST90267268	19	47041770	NPAS1	rs12609885	intron_variant	3.00E-12
GCST90255621	19	18344015	PGPEP1	rs17724992	intron_variant	4.00E-28
GCST90179150	19	18344015	PGPEP1	rs17724992	intron_variant	2.00E-21
GCST009871	19	18350146	PGPEP1	rs113230003	intron_variant	3.00E-20
GCST007039	19	18350146	PGPEP1	rs113230003	intron_variant	4.00E-20
GCST009003	19	18356588	PGPEP1	rs12984770	intron_variant	6.00E-17
GCST004904	19	18344015	PGPEP1	rs17724992	intron_variant	4.00E-12
GCST006368	19	45698914	QPCTL	rs2287019	intron_variant	1.00E-19
GCST002783	19	45698914	QPCTL	rs2287019	intron_variant	2.00E-18
GCST002783	19	45698914	QPCTL	rs2287019	intron_variant	5.00E-18
GCST000830	19	45698914	QPCTL	rs2287019	intron_variant	2.00E-16
GCST009871	19	45704328	QPCTL	rs78784145	intergenic_variant	1.00E-15
GCST009871	19	45701620	QPCTL	rs11083779	intron_variant	3.00E-12
GCST002783	19	45698914	QPCTL	rs2287019	intron_variant	3.00E-12
GCST006802	19	45698914	QPCTL	rs2287019	intron_variant	8.00E-11
GCST90018947	19	1831868	REXO1	rs144020965	intron_variant	4.00E-16
GCST008129	19	1819126	REXO1	rs2396359	missense_variant	2.00E-11
GCST90255621	19	1891993	SCAMP4	rs3957285	regulatory_region_variant	4.00E-20
GCST90179150	19	1937194	SCAMP4	rs11672550	regulatory_region_variant	6.00E-15
GCST90255621	19	2245623	SF3A2	rs45521740	non_coding_transcript_exon_	1.00E-19
GCST009004	19	2245623	SF3A2	rs45521740	non_coding_transcript_exon_	3.00E-14
GCST90179150	19	2245623	SF3A2	rs45521740	non_coding_transcript_exon_	3.00E-13
GCST009001	19	2244850	SF3A2	rs45486197	non_coding_transcript_exon_	2.00E-12
GCST009871	19	2245623	SF3A2	rs45521740	non_coding_transcript_exon_	3.00E-11
GCST007039	19	2245623	SF3A2	rs45521740	non_coding_transcript_exon_	4.00E-11
GCST009004	19	19281042	SUGP1	rs6511027	non_coding_transcript_exon_	1.00E-14
GCST009871	19	19314336	SUGP1	rs112253053	intron_variant	4.00E-13
GCST004046	19	19296909	SUGP1	rs10401969	intron_variant	2.00E-11
GCST004045	19	19296909	SUGP1	rs10401969	intron_variant	4.00E-11
GCST90275047	19	19268740	TM6SF2	rs58542926	missense_variant	2.00E-28
GCST90275050	19	19268740	TM6SF2	rs58542926	missense_variant	5.00E-15
GCST90275044	19	19268740	TM6SF2	rs58542926	missense_variant	5.00E-14
GCST011335	19	44892362	TOMM40	rs2075650	intron_variant	2.00E-237
GCST011336	19	44892362	TOMM40	rs2075650	intron_variant	2.00E-29
GCST009001	19	44892362	TOMM40	rs2075650	intron_variant	7.00E-26
GCST011334	19	44892362	TOMM40	rs2075650	intron_variant	1.00E-23
GCST011330	19	44892362	TOMM40	rs2075650	intron_variant	1.00E-14
GCST006368	19	44892362	TOMM40	rs2075650	intron_variant	1.00E-13
GCST90179150	19	4064059	ZBTB7A	rs56356382	intron_variant	6.00E-20
GCST009004	19	4064059	ZBTB7A	rs56356382	intron_variant	3.00E-19
GCST007039	19	4050426	ZBTB7A	rs72976986	intron_variant	5.00E-19
GCST90255621	19	4060709	ZBTB7A	rs895330	intron_variant	6.00E-19
GCST009871	19	4064059	ZBTB7A	rs56356382	intron_variant	1.00E-18
GCST009003	19	4086809	ZBTB7A	rs350818	intergenic_variant	4.00E-16
GCST90018947	19	4067316	ZBTB7A	rs188955288	regulatory_region_variant	2.00E-15
GCST90255621	19	47065746	ZC3H4	rs3810291	3_prime_UTR_variant	1.00E-68
GCST90179150	19	47065746	ZC3H4	rs3810291	3_prime_UTR_variant	3.00E-51
GCST007039	19	47065746	ZC3H4	rs3810291	3_prime_UTR_variant	3.00E-43
GCST009871	19	47060275	ZC3H4	rs12151152	intergenic_variant	2.00E-29

GCST009004	19	47060275	ZC3H4	rs12151152	intergenic_variant	7.00E-27
GCST009003	19	47059252	ZC3H4	rs7259070	regulatory_region_variant	2.00E-26
GCST009001	19	47054713	ZC3H4	rs112551143	intergenic_variant	7.00E-26
GCST005951	19	47065746	ZC3H4	rs3810291	3_prime_UTR_variant	1.00E-19
GCST006368	19	47065746	ZC3H4	rs3810291	3_prime_UTR_variant	8.00E-19
GCST002783	19	47065746	ZC3H4	rs3810291	3_prime_UTR_variant	6.00E-16
GCST002783	19	47065746	ZC3H4	rs3810291	3_prime_UTR_variant	5.00E-15
GCST004497	19	47065746	ZC3H4	rs3810291	3_prime_UTR_variant	1.00E-13
GCST004495	19	47065746	ZC3H4	rs3810291	3_prime_UTR_variant	1.00E-12
GCST000830	19	47065746	ZC3H4	rs3810291	3_prime_UTR_variant	2.00E-12
GCST004497	19	47065746	ZC3H4	rs3810291	3_prime_UTR_variant	4.00E-11
GCST90255621	19	30525289	ZNF536	rs11668301	intron_variant	2.00E-23
GCST007039	19	30528873	ZNF536	rs11084553	intron_variant	1.00E-18
GCST90179150	19	30528873	ZNF536	rs11084553	intron_variant	3.00E-16
GCST009871	19	30537759	ZNF536	rs10518269	intron_variant	8.00E-16
GCST90255621	19	30448453	ZNF536	rs33429	intron_variant	3.00E-15
GCST009004	19	30537759	ZNF536	rs10518269	intron_variant	5.00E-14
GCST009001	19	30537759	ZNF536	rs10518269	intron_variant	5.00E-14
GCST90179150	19	30448536	ZNF536	rs33430	intron_variant	2.00E-12
GCST90255621	19	30192972	ZNF536	rs2866816	intergenic_variant	6.00E-12
GCST90255621	20	6632185	CASC20	rs1884897	regulatory_region_variant	6.00E-34
GCST009004	20	6632185	CASC20	rs1884897	regulatory_region_variant	3.00E-28
GCST009003	20	6632185	CASC20	rs1884897	regulatory_region_variant	3.00E-28
GCST90179150	20	6642727	CASC20	rs979012	intergenic_variant	2.00E-27
GCST009001	20	6641038	CASC20	rs2145270	regulatory_region_variant	1.00E-26
GCST007039	20	6632185	CASC20	rs1884897	regulatory_region_variant	2.00E-25
GCST009871	20	6632185	CASC20	rs1884897	regulatory_region_variant	4.00E-25
GCST90018947	20	6614999	CASC20	rs4813800	intergenic_variant	9.00E-18
GCST90271771	20	6481955	CASC20	rs964220	intron_variant	5.00E-12
GCST006368	20	6645571	CASC20	rs2145272	regulatory_region_variant	6.00E-11
GCST90255621	20	55867410	CBLN4	rs2870687	intergenic_variant	1.00E-12
GCST90179150	20	55819919	CBLN4	rs6014523	intergenic_variant	7.00E-11
GCST90255621	20	46276199	CDH22	rs2425840	intron_variant	2.00E-14
GCST90179150	20	46276199	CDH22	rs2425840	intron_variant	5.00E-12
GCST009004	20	46285495	CDH22	rs2425857	intron_variant	2.00E-11
GCST007039	20	46276199	CDH22	rs2425840	intron_variant	5.00E-11
GCST90255621	20	62908967	DIDO1	rs6010784	intron_variant	7.00E-16
GCST90179150	20	62908967	DIDO1	rs6010784	intron_variant	5.00E-11
GCST90255621	20	25206577	ENTPD6	rs2076559	missense_variant	5.00E-21
GCST90255621	20	25214873	ENTPD6	rs6050446	stop_gained	9.00E-20
GCST008129	20	25206577	ENTPD6	rs2076559	missense_variant	2.00E-16
GCST009004	20	25211413	ENTPD6	rs8122855	intron_variant	4.00E-14
GCST90179150	20	25211413	ENTPD6	rs8122855	intron_variant	2.00E-13
GCST007039	20	25210141	ENTPD6	rs4456769	intron_variant	3.00E-13
GCST009871	20	25209400	ENTPD6	rs55927253	intron_variant	4.00E-13
GCST90179150	20	25214873	ENTPD6	rs6050446	stop_gained	7.00E-13
GCST90255621	20	15838850	MACROD2	rs8123881	intron_variant	4.00E-20
GCST90179150	20	15838850	MACROD2	rs8123881	intron_variant	8.00E-13
GCST009004	20	15832830	MACROD2	rs16996644	intron_variant	2.00E-12
GCST90255621	20	15104719	MACROD2	rs17272434	intron_variant	2.00E-11
GCST009004	20	40663144	MAFB	rs16989232	intergenic_variant	6.00E-12

GCST90179150	20	40663144	MAFB	rs16989232	intergenic_variant	9.00E-12
GCST90271770	20	40603657	MAFB	rs6029202	intergenic_variant	4.00E-11
GCST009004	20	21400483	NKX2-4 - RN7SKP140	rs1409818	intergenic_variant	3.00E-12
GCST009003	20	21400483	NKX2-4 - RN7SKP140	rs1409818	intergenic_variant	3.00E-12
GCST90255621	20	21400483	NKX2-4 - RN7SKP140	rs1409818	intergenic_variant	3.00E-12
GCST90179150	20	21400483	NKX2-4 - RN7SKP140	rs1409818	intergenic_variant	8.00E-12
GCST90255621	20	33965289	PIGPP3	rs4911382	non_coding_transcript_exon_	8.00E-20
GCST90179150	20	33965241	PIGPP3	rs4012234	non_coding_transcript_exon_	3.00E-13
GCST009004	20	33968766	PIGPP3	rs6142067	intergenic_variant	4.00E-13
GCST007039	20	33965289	PIGPP3	rs4911382	non_coding_transcript_exon_	8.00E-13
GCST009871	20	33968766	PIGPP3	rs6142067	intergenic_variant	1.00E-12
GCST90255621	20	43358752	PPIAP21	rs2143253	intergenic_variant	3.00E-16
GCST007039	20	43362121	PPIAP21	rs6103254	intergenic_variant	6.00E-12
GCST009004	20	43382356	PPIAP21	rs6130360	intergenic_variant	7.00E-12
GCST90179150	20	43382356	PPIAP21	rs6130360	intergenic_variant	4.00E-11
GCST90018947	20	43357867	PPIAP21	rs6030803	intergenic_variant	9.00E-11
GCST007039	20	48881580	PREX1 - ARFGEF2	rs112852122	regulatory_region_variant	2.00E-15
GCST009871	20	48881580	PREX1 - ARFGEF2	rs112852122	regulatory_region_variant	2.00E-14
GCST90179150	20	48877468	PREX1 - ARFGEF2	rs13037473	regulatory_region_variant	2.00E-14
GCST009004	20	48879119	PREX1 - ARFGEF2	rs6019483	regulatory_region_variant	4.00E-14
GCST90255621	20	48840522	PREX1 - ARFGEF2	rs11905956	intron_variant	5.00E-13
GCST009001	20	48833526	PREX1 - ARFGEF2	rs34417222	intron_variant	7.00E-11
GCST007039	20	54854044	RNU4ATAC7P	rs6023649	intergenic_variant	3.00E-11
GCST009871	20	54836787	RNU4ATAC7P	rs1512065	intergenic_variant	9.00E-11
GCST90255621	20	54861530	RNU4ATAC7P	rs912507	intergenic_variant	9.00E-11
GCST90179150	20	17190728	RNU6-27P - RNU1-131P	rs947088	intergenic_variant	3.00E-11
GCST009004	20	17190728	RNU6-27P - RNU1-131P	rs947088	intergenic_variant	4.00E-11
GCST009003	20	17190728	RNU6-27P - RNU1-131P	rs947088	intergenic_variant	4.00E-11
GCST90255621	20	24796095	SYNDIG1	rs226688	intergenic_variant	9.00E-13
GCST90179150	20	24796095	SYNDIG1	rs226688	intergenic_variant	4.00E-11
GCST90255621	20	64060197	TCEA2	rs6512302	intron_variant	9.00E-14
GCST009004	20	64060197	TCEA2	rs6512302	intron_variant	2.00E-11
GCST90255621	20	52490751	ZFP64	rs17806379	intron_variant	3.00E-39
GCST009004	20	52449315	ZFP64	rs17806224	intron_variant	8.00E-32
GCST009003	20	52449315	ZFP64	rs17806224	intron_variant	8.00E-32
GCST90179150	20	52490751	ZFP64	rs17806379	intron_variant	8.00E-31
GCST007039	20	52578848	ZFP64	rs66460909	intron_variant	5.00E-27
GCST009001	20	52343951	ZFP64	rs35388084	intron_variant	6.00E-26
GCST009871	20	52579393	ZFP64	rs73142879	intron_variant	7.00E-26
GCST90018947	20	52532117	ZFP64	rs11907932	intron_variant	2.00E-22
GCST006368	20	52471323	ZFP64	rs6091540	intron_variant	4.00E-16
GCST009871	20	52230379	ZFP64	rs6021798	intron_variant	1.00E-13
GCST004904	20	52471323	ZFP64	rs6091540	intron_variant	5.00E-13
GCST006368	20	52365406	ZFP64	rs16996700	intron_variant	2.00E-12
GCST90255621	20	52255271	ZFP64	rs7271567	intron_variant	1.00E-11
GCST002783	20	52471323	ZFP64	rs6091540	intron_variant	2.00E-11
GCST90179150	20	52259063	ZFP64	rs2426424	intron_variant	3.00E-11
GCST009871	20	52135557	ZFP64	rs34248912	intron_variant	3.00E-11
GCST009004	21	45150981	ADAMTS9	rs427943	intron_variant	4.00E-25
GCST009003	21	45150981	ADARB1	rs427943	intron_variant	4.00E-25
GCST009001	21	45150981	ADARB1	rs427943	intron_variant	4.00E-25

GCST90179150	21	45150981	ADARB1	rs427943	intron_variant	2.00E-24
GCST90255621	21	45150981	ADARB1	rs427943	intron_variant	2.00E-22
GCST009871	21	45161883	ADARB1	rs394608	intron_variant	4.00E-21
GCST007039	21	45161883	ADARB1	rs394608	intron_variant	1.00E-20
GCST90018947	21	45161883	ADARB1	rs394608	intron_variant	5.00E-14
GCST90255621	21	41281362	B4GALNT4	rs1041448	3_prime_UTR_variant	4.00E-15
GCST90179150	21	41281640	BACE2	rs2838006	3_prime_UTR_variant	7.00E-13
GCST009004	21	41261138	BACE2	rs4818226	intron_variant	2.00E-11
GCST90271767	21	39239516	BRINP3	rs4818008	intron_variant	1.00E-12
GCST009004	21	39272244	BRWD1	rs8134638	intron_variant	2.00E-11
GCST009871	21	39272244	BRWD1	rs8134638	intron_variant	4.00E-11
GCST007039	21	39272244	BRWD1	rs8134638	intron_variant	4.00E-11
GCST009871	21	45069044	PICSAR	rs76040172	intergenic_variant	9.00E-16
GCST90179150	21	45067815	PICSAR	rs79585412	regulatory_region_variant	2.00E-15
GCST90255621	21	45069044	PICSAR	rs76040172	intergenic_variant	3.00E-15
GCST90267268	21	45067815	PICSAR	rs79585412	regulatory_region_variant	1.00E-11
GCST90179150	21	45047012	PICSAR	rs4819021	intergenic_variant	7.00E-11
GCST90255621	21	38919816	RPSAP64	rs2836754	intron_variant	4.00E-27
GCST90179150	21	38919816	RPSAP64	rs2836754	intron_variant	1.00E-15
GCST90255621	21	38931504	RPSAP64	rs2222994	intron_variant	8.00E-13
GCST90018947	21	38937512	RPSAP64	rs13047416	intron_variant	9.00E-13
GCST004904	21	38939569	RPSAP64	rs2836768	intron_variant	6.00E-12
GCST009871	21	38916653	RPSAP64	rs8132491	intron_variant	8.00E-12
GCST007039	21	38937512	RPSAP64	rs13047416	intron_variant	1.00E-11
GCST006368	21	38919816	RPSAP64	rs2836754	intron_variant	7.00E-11
GCST90275047	22	43928847	PNPLA3	rs738409	missense_variant	3.00E-41
GCST90275050	22	43928847	PNPLA3	rs738409	missense_variant	1.00E-17
GCST90275044	22	43928847	PNPLA3	rs738409	missense_variant	9.00E-13
GCST90255621	22	48475812	TAFA5	rs13053342	non_coding_transcript_exon_	5.00E-13
GCST009004	22	48475207	TAFA5	rs713763	non_coding_transcript_exon_	4.00E-12
GCST90179150	22	48475271	TAFA5	rs737720	non_coding_transcript_exon_	9.00E-12
GCST007039	22	48475207	TAFA5	rs713763	non_coding_transcript_exon_	3.00E-11
GCST009871	22	48475207	TAFA5	rs713763	non_coding_transcript_exon_	5.00E-11
GCST90179150	22	41408712	TEF - TOB2	rs28489620	regulatory_region_variant	4.00E-12
GCST007039	22	41408712	TEF - TOB2	rs28489620	regulatory_region_variant	4.00E-12
GCST90255621	22	40208941	TNRC6B	rs4820408	intron_variant	2.00E-28
GCST90018947	22	40316633	TNRC6B	rs6001877	intron_variant	1.00E-22
GCST009004	22	40258272	TNRC6B	rs12628051	intron_variant	3.00E-19
GCST009003	22	40162060	TNRC6B	rs12484438	intron_variant	5.00E-19
GCST90179150	22	40301373	TNRC6B	rs5995843	non_coding_transcript_exon_	2.00E-18
GCST007039	22	40294381	TNRC6B	rs4820410	intron_variant	6.00E-17
GCST004904	22	40208941	TNRC6B	rs4820408	intron_variant	2.00E-16
GCST009871	22	40294381	TNRC6B	rs4820410	intron_variant	6.00E-16
GCST009001	22	40273644	TNRC6B	rs733381	intron_variant	8.00E-16
GCST004904	22	40317857	TNRC6B	rs139913	intron_variant	2.00E-15
GCST90018727	22	40317857	TNRC6B	rs139913	intron_variant	4.00E-12
GCST90018947	X	118797395	IL13RA1	rs2489879	intergenic_variant	8.00E-27
GCST004904	X	118782407	IL13RA1	rs3121672	intron_variant	3.00E-17
GCST90018727	X	118797395	IL13RA1	rs2489879	intergenic_variant	3.00E-14
