# Supplementary material for: A Complex Network of Obesity-Risk Genes Revealed by Systematic Bioinformatics and Single-Cell Transcriptomic Analyses
Source: J Obes. 2025 Mar 31;2025:7821115. doi: 10.1155/jobe/7821115 (PMC11976034; doi:10.1155/jobe/7821115)
Supplement: Supporting Information — Additional supporting information can be found online in the Supporting Information section. [file 7821115.f1.zip › Supplementary Table 2.pdf]

Gene	Class
AARS1	Protein-coding
ABCF2P1	Protein-coding
ABCG2	Protein-coding
ABHD17C	Protein-coding
ABHD5	Protein-coding
ACTG1P22	Protein-coding
ADAMTS9	Protein-coding
ADARB1	Protein-coding
ADCY3	Protein-coding
ADCY5	Protein-coding
ADCY9	Protein-coding
ADGRB3	Protein-coding
ADGRL2	Protein-coding
ADH1B	Protein-coding
ADK	Protein-coding
ADPGK	Protein-coding
AFF3	Protein-coding
AGAP1	Protein-coding
AGBL4	Protein-coding
AIDAP3	Protein-coding
AIF1	Protein-coding
AK5	Protein-coding
AKAP6	Protein-coding
AKAP8P1 - JKAMPP1	Read-Through
AKT3	Protein-coding
ALDH2	Protein-coding
ALKAL2	Protein-coding
ALKBH3	Protein-coding
ANAPC4	Protein-coding
ANKDD1B	Protein-coding
ANKRD26	Protein-coding
ANKRD31	Protein-coding
ANKRD34C	Protein-coding
ANKS1A	Protein-coding
ANKS1B	Protein-coding
ANTXR2	Protein-coding
APC	Protein-coding
APOB	Protein-coding
APOC1	Protein-coding
APOE	Protein-coding
ARAP1	Protein-coding
ARG1	Protein-coding
ARHGAP15	Protein-coding
ARL14EP	Protein-coding
ARL15	Protein-coding
ARPP21	Protein-coding
AS3MT	Protein-coding
ASB3	Protein-coding
ASCC3	Protein-coding
ASCL4	Protein-coding

ASH1L	Protein-coding
ASIC2	Protein-coding
ASMT	Protein-coding
ASS1P14 - SYT10	Read-Through
ASTN2	Protein-coding
ASXL3	Protein-coding
ATP2A1	Protein-coding
ATXN2	Protein-coding
ATXN2L	Protein-coding
ATXN7L3	Protein-coding
AUTS2	Protein-coding
AXIN1	Protein-coding
B3GAT1	Protein-coding
B4GALNT4	Protein-coding
BACE2	Protein-coding
BAD, GPR137	Protein-coding
BAG6	Protein-coding
BAIAP2	Protein-coding
BANK1	Protein-coding
BARX1	Protein-coding
BAZ1B	Protein-coding
BBS4	Protein-coding
BCDIN3D	Protein-coding
BCL11B	Protein-coding
BCL2	Protein-coding
BCL7A	Protein-coding
BDNF	Protein-coding
BIN2P2	Protein-coding
BLC3	Protein-coding
BLTP3A	Protein-coding
BMAL1	Protein-coding
BNC2	Protein-coding
BNIPL	Protein-coding
BPTF	Protein-coding
BRINP3	Protein-coding
BRWD1	Protein-coding
BTBD7	Protein-coding
C12orf42	Protein-coding
C8orf90	Protein-coding
CACNA1C	Protein-coding
CACNA1D	Protein-coding
CACNA2D2	Protein-coding
CACNB2	Protein-coding
CADM1	Protein-coding
CADM2	Protein-coding
CADPS	Protein-coding
CADPS2	Protein-coding
CALCR	Protein-coding
CALM2P1	Protein-coding
CALN1	Protein-coding
CAMKMT	Protein-coding

CAMKV	Protein-coding
CAMTA1	Protein-coding
CAPZA3	Protein-coding
CAPZBP1	Protein-coding
CASC20	Protein-coding
CAST	Protein-coding
CBLN4	Protein-coding
CBX5, SCAT2	Protein-coding
CCDC146	Protein-coding
CCDC171	Protein-coding
CCDC59	Protein-coding
CCDC92, DNAH10	Protein-coding
CCK	Protein-coding
CCND1	Protein-coding
CCNE1	Protein-coding
CCNL1	Protein-coding
CCT5P2 - NIPA2P5	Protein-coding
CD19	Protein-coding
CD47	Protein-coding
CDH13	Protein-coding
CDH22	Protein-coding
CDH7	Protein-coding
CDKAL1	Protein-coding
CELF1	Protein-coding
CELSR2	Protein-coding
CEP120	Protein-coding
CERT1	Protein-coding
CFAP74	Protein-coding
CGGBP1	Protein-coding
CHID1	Protein-coding
CHMP3	Protein-coding
CHRNB4	Protein-coding
CHRNE	Protein-coding
CHST1	Protein-coding
CKB	Protein-coding
CLIP1	Protein-coding
CLUAP1	Protein-coding
CLVS1	Protein-coding
CMIP	Protein-coding
CNNM2	Protein-coding
COBLL1	Protein-coding
COL16A1	Protein-coding
COPZ2	Protein-coding
CPEB4	Protein-coding
CPNE4	Protein-coding
CPNE8-AS1 - LINC02406	Read-Through
CPS1	Protein-coding
CPSF6	Protein-coding
CRB1	Protein-coding
CRB2	Protein-coding
CREB1	Protein-coding

CRIPT	Protein-coding
CRTAC1	Protein-coding
CRTC1	Protein-coding
CSMD1	Protein-coding
CSNK1G2	Protein-coding
CTBP2	Protein-coding
CTDSP1 - VIL1	Read-Through
CTNNA2	Protein-coding
CWC27	Protein-coding
CXXC5	Protein-coding
CYP51A1P1	Protein-coding
DCAF12	Protein-coding
DCAF7	Protein-coding
DCC	Protein-coding
DDC	Protein-coding
DDX43P2 - VWC2	Read-Through
DEFB112 - TFAP2D	Read-Through
DELEC1	Protein-coding
DGKG	Protein-coding
DGKI	Protein-coding
DHX36	Protein-coding
DIDO1	Protein-coding
DIS3L2	Protein-coding
DLG2	Protein-coding
DLG4	Protein-coding
DLK1	Protein-coding
DMRTA1	Protein-coding
DMXL2	Protein-coding
DNAJA1P1	Protein-coding
DNAJC11	Protein-coding
DNAJC27	Protein-coding
DNER	Protein-coding
DNMT3A	Protein-coding
DOCK3	Protein-coding
DPF3	Protein-coding
DPPA3P11	Protein-coding
DPYD	Protein-coding
DSTNP5 - PARD3B	Read-Through
DTX2P1	Protein-coding
DUSP6	Protein-coding
DUX4L52	Protein-coding
DYNC1I2	Protein-coding
E2F3	Protein-coding
EEF1A1P11	Protein-coding
EEF1A1P47	Protein-coding
EEF1AKMT4	Protein-coding
EFEMP2	Protein-coding
EFR3B	Protein-coding
EHBP1	Protein-coding
EIF4BP4	Protein-coding
EIF4EBP2P3	Protein-coding

EIF5A2	Protein-coding
ELAVL4	Protein-coding
ELP3	Protein-coding
ENO4	Protein-coding
ENTPD6	Protein-coding
ERBB3	Protein-coding
ERBB4	Protein-coding
ETV5	Protein-coding
EVI5	Protein-coding
EXD3	Protein-coding
EXT1	Protein-coding
EZRP1 - ALCAM	Read-Through
FADS1, FADS2	Protein-coding
FAIM2	Protein-coding
FAM114A2	Protein-coding
FAM150B	Protein-coding
FANCL	Protein-coding
FBXL17	Protein-coding
FBXO33	Protein-coding
FECHP1 - KRT8P18	Read-Through
FGFR4 - NSD1	Read-Through
FHIT	Protein-coding
FIBCD1	Protein-coding
FIGN	Protein-coding
FLRT1	Protein-coding
FLT3	Protein-coding
FOXO3	Protein-coding
FOXP2 - MDFIC	Read-Through
FREM1	Protein-coding
FRRS1L - EPB41L4B	Read-Through
FTO	Protein-coding
GABPB2	Protein-coding
GABRB3	Protein-coding
GALC	Protein-coding
GALNT16	Protein-coding
GBE1	Protein-coding
GCK	Protein-coding
GCKR	Protein-coding
GCNT4	Protein-coding
GDF15	Protein-coding
GGNBP1	Protein-coding
GGNBP2	Protein-coding
GIPC2	Protein-coding
GIPR	Protein-coding
GNAI2P1	Protein-coding
GNAT2	Protein-coding
GNB1	Protein-coding
GNGT1	Protein-coding
GOLGA2	Protein-coding
GON4L	Protein-coding
GP2	Protein-coding

GPN3	Protein-coding
GPR139	Protein-coding
GPR61	Protein-coding
GPRC5B	Protein-coding
GRB14	Protein-coding
GRID1	Protein-coding
GRID2	Protein-coding
GRIN2A	Protein-coding
GSAP - GCNT1P5	Read-Through
GTF2A1L,LHCGR	Protein-coding
GTF2I	Protein-coding
GTF3A	Protein-coding
GUCY2EP - TSKU	Read-Through
GYPA - KRT18P51	Read-Through
H2BC4	Protein-coding
HACD2	Protein-coding
HAPLN4	Protein-coding
HBEGF	Protein-coding
HCAR2 - HCAR3	Read-Through
HCN4	Protein-coding
HECTD4	Protein-coding
HERPUD1 - CETP	Read-Through
HEY2 - NCOA7	Read-Through
HHIP	Protein-coding
HHLA2	Protein-coding
HIF1AN	Protein-coding
HIP1	Protein-coding
HIP1R	Protein-coding
HIVEP1	Protein-coding
HIVEP2	Protein-coding
HLA-DRB1	Read-Through
HMGA1	Protein-coding
HMGB1	Protein-coding
HMGB1P18 - HNRNPA1P64	Read-Through
HMGB1P47	Protein-coding
HMGN1P31	Protein-coding
HNF4G	Protein-coding
HNF4GP1	Protein-coding
HNRNPA1P57 - LDHAP3	Read-Through
HOXB-AS3, HOXB5, HOXB3	Protein-coding, non-coding RNA
HS6ST3	Protein-coding
HSD17B12	Protein-coding
HSP90AA6P	Protein-coding
HSPE1P19	Protein-coding
HTR1A	Protein-coding
HYAL3	Protein-coding
IGBP1P5	Protein-coding
IGF1	Protein-coding
IGF1R	Protein-coding
IGF2BP1	Protein-coding
IGF2BP2	Protein-coding

IGSF9B	Protein-coding
IL13RA1	Protein-coding
IL34	Protein-coding
ILRUN	Protein-coding
INHBC	Protein-coding
INKA2	Protein-coding
IPO9	Protein-coding
IPP	Protein-coding
IQCH	Protein-coding
ITGAL	Protein-coding
ITGAX	Protein-coding
ITIH1	Protein-coding
ITIH3	Protein-coding
JADE2	Protein-coding
JAKMIP3	Protein-coding
JAZF1	Protein-coding
JMJD1C	Protein-coding
KAT8	Protein-coding
KCNB2	Protein-coding
KCND3	Protein-coding
KCNH2	Protein-coding
KCNJ11	Protein-coding
KCNK3	Protein-coding
KCNMA1	Protein-coding
KCNQ1	Protein-coding
KCNQ5	Protein-coding
KCTD15	Protein-coding
KCTD8	Protein-coding
KDM4C	Protein-coding
KIAA1429	Protein-coding
KIAA1522	Protein-coding
KIT	Protein-coding
KLC1	Protein-coding
KLF1	Protein-coding
KLF14	Protein-coding
KLF16	Protein-coding
KLF3	Protein-coding
KLF7	Protein-coding
KNTC1	Protein-coding
L3MBTL3	Protein-coding
LARS2	Protein-coding
LCORL	Protein-coding
LEO1, MAPK6	Protein-coding
LEPR	Protein-coding
LETR1	Protein-coding
LGR4	Protein-coding
LINC00458	lncRNA
LINC00461	lncRNA
LINC01505	lncRNA
LINC01541	lncRNA
LINC01698	lncRNA

LINC01872	lncRNA
LINC01898 - LINC01893	lncRNA
LINC01911 - RNU6-692P	lncRNA
LINC01915	lncRNA
LINC02006	lncRNA
LINC02059 - MIR4280HG	lncRNA
LINC02240	lncRNA
LINC02288	lncRNA
LINC02399 - LINC02392	lncRNA
LINC02421 - LINC01479	lncRNA
LINC02465	lncRNA
LINC02607	lncRNA
LINC02628 - LINC00838	lncRNA
LINC02641	lncRNA
LINC02706 - LINC02714	lncRNA
LINC02790 - RNU1-130P	lncRNA
LINC02822	lncRNA
LINC02831	lncRNA
LINC03012	lncRNA
LINGO1	Protein-coding
LINGO2	Protein-coding
LIPC	Protein-coding
LMOD1	Protein-coding
LMX1B	Protein-coding
LONRF2	Protein-coding
LOXL4	Protein-coding
LPL	Protein-coding
LRFN2	Protein-coding
LRFN5 - YWHAQP1	Read-Through
LRMDA	Protein-coding
LRP1B	Protein-coding
LRRC2P1 - HNRNPA1P39	Read-Through
LTBP1	Protein-coding
LTO1	Protein-coding
LYPLAL1	Protein-coding
LYRM2, ANKRD6	Protein-coding
LYSMD2	Protein-coding
LYZ	Protein-coding
LYZL4	Protein-coding
LZTS1	Protein-coding
MACF1	Protein-coding
MACROD2	Protein-coding
MAD1L1	Protein-coding
MAD2L1BP	Protein-coding
MADD	Protein-coding
MAFB	Protein-coding
MAGI2	Protein-coding
MALAT1	Protein-coding
MAML3	Protein-coding
MAN1A1	Protein-coding
MAP2K1	Protein-coding

MAP2K3	Protein-coding
MAP2K5	Protein-coding
MAP3K3	Protein-coding
MAPK3	Protein-coding
MARK3	Protein-coding
MAST2	Protein-coding
MAST3	Protein-coding
MAST4	Protein-coding
MC4R	Protein-coding
MCM6	Protein-coding
MDGA2	Protein-coding
MEF2C	Protein-coding
MEG9	Protein-coding
METAP1D	Protein-coding
MEX3A	Protein-coding
MFAP3	Protein-coding
MIGA1	Protein-coding
MIR100HG	microRNA host gene
MIR4432HG	microRNA host gene
MIR9-1HG	microRNA host gene
MIR9-3HG	microRNA host gene
MLLT10	Protein-coding
MLN	Protein-coding
MLXIP	Protein-coding
MLXIPL	Protein-coding
MMS22L	Protein-coding
MOK	Protein-coding
MON1A	Protein-coding
MPPED2	Protein-coding
MRAS	Protein-coding
MRM1	Protein-coding
MRPS14 - ENTR1P2	Read-Through
MRPS5P4 - GAD3P	Read-Through
MRPS9-AS2	Read-Through
MSRA	Protein-coding
MST1R	Protein-coding
MTCH2	Protein-coding
MTCO3P28	Protein-coding
MTIF3	Protein-coding
MTOR	Protein-coding
MYH15	Protein-coding
MYO19	Protein-coding
MYO1E	Protein-coding
N4BP2L2	Protein-coding
NAALAD2	Protein-coding
NAMPTP2 - ASS1P10	Read-Through
NBEAL1	Protein-coding
NCKAP5	Protein-coding
NCOA1	Protein-coding
NDUFA5P5 - ICE2P2	Read-Through
NDUFS5P2	Protein-coding

NECTIN2	Protein-coding
NEGR1	Protein-coding
NEK6	Protein-coding
NFAT5	Protein-coding
NFIL3	Protein-coding
NID2	Protein-coding
NKAIN2	Protein-coding
NKX2-4 - RN7SKP140	Read-Through
NLGN1	Protein-coding
NLRC3	Protein-coding
NOLC1 - ELOVL3	Read-Through
NOTCH4	Protein-coding
NPAS1	Protein-coding
NPC1	Protein-coding
NPM1P10 - HACE1	Read-Through
NPM1P31 - ZRANB1	Read-Through
NPM1P47	Protein-coding
NPTX1	Protein-coding
NPY	Protein-coding
NRG1	Protein-coding
NRXN1	Protein-coding
NRXN3	Protein-coding
NSUN3	Protein-coding
NT5C2	Protein-coding
NTHL1	Protein-coding
NTM	Protein-coding
NTNG1	Protein-coding
NTRK2	Protein-coding
NUDT21	Protein-coding
NUP160	Protein-coding
NXPH4	Protein-coding
NYAP2	Protein-coding
OGDH	Protein-coding
OLA1	Protein-coding
OLFM4	Protein-coding
ONECUT1 - RPSAP55	Read-Through
OPCML	Protein-coding
OPRM1	Protein-coding
OR7K1P	Protein-coding
OTUD7A	Protein-coding
P2RX4	Protein-coding
P4HTM	Protein-coding
PA2G4	Protein-coding
PABPC4	Protein-coding
PACS1	Protein-coding
PACSIN1	Protein-coding
PANK4	Protein-coding
PANTR1	Protein-coding
PATJ	Protein-coding
PAX2	Protein-coding
PCCB	Protein-coding

PCDH17	Protein-coding
PCDH7	Protein-coding
PCDH9	Protein-coding
PCDHA1	Protein-coding
PCSK1	Protein-coding
PDE1C	Protein-coding
PDE4B	Protein-coding
PDILT	Protein-coding
PDS5B	Protein-coding
PDXDC1	Protein-coding
PDZRN4	Protein-coding
PGPEP1	Protein-coding
PHACTR1	Protein-coding
PHF2	Protein-coding
PHF21A	Protein-coding
PIAS1	Protein-coding
PICSAR	Protein-coding
PIGPP3	Protein-coding
PIK3C3	Protein-coding
PKD1L3	Protein-coding
PKHD1	Protein-coding
PLCL1	Protein-coding
PLEKHA5	Protein-coding
PMFBP1	Protein-coding
PMS2P3	Protein-coding
PNPLA3	Protein-coding
POC5	Protein-coding
POLD2P1	Protein-coding
POM121C	Protein-coding
POU6F2	Protein-coding
PPARG	Protein-coding
PPIAP21	Protein-coding
PPL	Protein-coding
PPM1G	Protein-coding
PPP1CB	Protein-coding
PPP1R3B	Protein-coding
PPP2R3A	Protein-coding
PPP3CA	Protein-coding
PRDX2P4 - SIM1	Read-Through
PRDX4P1	Protein-coding
PRDX5 - CCDC88B	Read-Through
PREX1 - ARFGEF2	Read-Through
PRKAG1	Protein-coding
PRKD1	Protein-coding
PRKN	Protein-coding
PRMT6	Protein-coding
PRMT7	Protein-coding
PRPH2	Protein-coding
PRRC2C	Protein-coding
PRSS37 - OR9A3P	Read-Through
PRUNE1	Protein-coding

PSAT1	Protein-coding
PSIP1	Protein-coding
PSMC3	Protein-coding
PSMD3	Protein-coding
PTBP2	Protein-coding
PTGFR	Protein-coding
PTK2B	Protein-coding
PTPN7	Protein-coding
PTPRD	Protein-coding
PTPRN	Protein-coding
PURG	Protein-coding
PXK	Protein-coding
QPCTL	Protein-coding
RAB21	Protein-coding
RAB27B	Protein-coding
RABEP1	Protein-coding
RABEP2	Protein-coding
RABGAP1L	Protein-coding
RAD52	Protein-coding
RALGAPA1	Protein-coding
RALYL	Protein-coding
RANBP17	Protein-coding
RAPGEF3	Protein-coding
RARB	Protein-coding
RASA2	Protein-coding
RASGRF1	Protein-coding
RBBP6	Protein-coding
RBFOX1	Protein-coding
RBM19	Protein-coding
RBM6	Protein-coding
RBMS1	Protein-coding
RCAN2	Protein-coding
REC114	Protein-coding
REEP3	Protein-coding
RELN	Protein-coding
RESP18	Protein-coding
REXO1	Protein-coding
RFLNA	Protein-coding
RFT1	Protein-coding
RFTN2	Protein-coding
RGS12	Protein-coding
RGS17	Protein-coding
RGS7BP	Protein-coding
RIT2	Protein-coding
RMDN1	Protein-coding
RN7SKP135	non-coding RNA
RN7SKP61 - MRPS17P3	non-coding RNA
RNA5SP173 - NDUFB5P1	non-coding RNA
RNA5SP30 - LINC02338	non-coding RNA
RNA5SP56 - PSMC1P12	non-coding RNA
RNA5SP94 - MIR4432HG	non-coding RNA

RNFT1P2 - MGC27382	non-coding RNA
RNU2-54P - LINC01109	non-coding RNA
RNU4ATAC7P	non-coding RNA
RNU6-144P - ZYXP1	non-coding RNA
RNU6-148P - BRWD1P2	non-coding RNA
RNU6-257P - MTND4LP25	non-coding RNA
RNU6-27P - RNU1-131P	non-coding RNA
RNU6-567P - RPS3AP49	non-coding RNA
RNU6-727P - LINC02488	non-coding RNA
RNU6-983P - LINC01724	non-coding RNA
ROBO1	Protein-coding
ROBO2	Protein-coding
RORA	Protein-coding
RPGRIP1L	Protein-coding
RPL10AP3 - LINC01288	Read-Through
RPL12P40 - RN7SKP182	Read-Through
RPL19P16 - LINC01153	Read-Through
RPL21P119 - LINC02177	Read-Through
RPL31P12 - RNU6-1246P	Read-Through
RPL36AP23	Protein-coding
RPS14P7 - FSTL5	Read-Through
RPS17P5 - FTH1P5	Read-Through
RPS24P8 - TMEM158	Read-Through
RPS6KA5	Protein-coding
RPSAP64	Protein-coding
RPTOR	Protein-coding
RSL24D1P4 - SEPTIN14P21	Read-Through
RSRC1	Protein-coding
RSU1	Protein-coding
RTN4	Protein-coding
RTN4RL1	Protein-coding
SAMMSON	Protein-coding
SBK1	Protein-coding
SCAMP4	Protein-coding
SCARB2	Protein-coding
SCHLAP1	Protein-coding
SCN2A	Protein-coding
SCUBE3	Protein-coding
SEC11C - GRP	Read-Through
SEC16B	Protein-coding
SEMA3F	Protein-coding
SEMA4D - GADD45G	Read-Through
SEMA6D	Protein-coding
SETBP1	Protein-coding
SETD5	Protein-coding
SF3A2	Protein-coding
SGCZ	Protein-coding
SGO1	Protein-coding
SH2B1	Protein-coding
SH3GL3	Protein-coding
SHTN1	Protein-coding

SIK3	Protein-coding
SINHCAF	Protein-coding
SKAP1	Protein-coding
SKOR1	Protein-coding
SLC10A7	Protein-coding
SLC14A2	Protein-coding
SLC17A1	Protein-coding
SLC22A12	Protein-coding
SLC22A3	Protein-coding
SLC25A37	Protein-coding
SLC25A44	Protein-coding
SLC2A2	Protein-coding
SLC2A9	Protein-coding
SLC35E2B	Protein-coding
SLC39A13	Protein-coding
SLC39A8	Protein-coding
SLC6A4	Protein-coding
SLC8A1	Protein-coding
SLCO3A1	Protein-coding
SLIT2	Protein-coding
SLITRK6 - MOB1AP1	Read-Through
SMARCC1	Protein-coding
SMG6	Protein-coding
SMIM30 - PPP1R3A	Read-Through
SMIM40 - MYL12BP3	Read-Through
SMIM7P1	Protein-coding
SNTB2	Protein-coding
SNX19	Protein-coding
SOX11	Protein-coding
SOX2-OT	Protein-coding
SOX5	Protein-coding
SPARC	Protein-coding
SPATA16	Protein-coding
SPATA19	Protein-coding
SPDYE18	Protein-coding
SPHKAP	Protein-coding
SQOR - MTND5P40	Read-Through
SRRM1P2	Protein-coding
SRRM2	Protein-coding
SSBP2	Protein-coding
SSBP3	Protein-coding
SSH2	Protein-coding
SSR3	Protein-coding
STAG1	Protein-coding
STARP1	Protein-coding
STK24	Protein-coding
STK33	Protein-coding
STOML1	Protein-coding
STX1B	Protein-coding
SUGP1	Protein-coding
SUPT3H	Protein-coding

SYNDIG1	Protein-coding
SYT14	Protein-coding
SYT16	Protein-coding
SYT4	Protein-coding
TAFA5	Protein-coding
TAL1	Protein-coding
TAOK2	Protein-coding
TBX15	Protein-coding
TBX4	Protein-coding
TCEA2	Protein-coding
TCF4	Protein-coding
TCF7L2	Protein-coding
TDRD15	Protein-coding
TDRG1	Protein-coding
TEF - TOB2	Read-Through
TERF1P3	Protein-coding
TEX10	Protein-coding
TEX29	Protein-coding
TEX46	Protein-coding
TFAP2B	Protein-coding
TLCD3B	Protein-coding
TLE1	Protein-coding
TM6SF2	Protein-coding
TMEM161B	Protein-coding
TMEM161B-DT	Read-Through
TMEM18	Protein-coding
TMEM219	Protein-coding
TNNI3K	Protein-coding
TNRC6A	Protein-coding
TNRC6B	Protein-coding
TOMM40	Protein-coding
TPBG - UBE3D	Read-Through
TRAF3	Protein-coding
TREM2	Protein-coding
TRIB1	Protein-coding
TRIM66	Protein-coding
TRIP12	Protein-coding
TRPM3	Protein-coding
TRPS1	Protein-coding
TSNARE1	Protein-coding
TTC17 - CTBP2P6	Read-Through
TTC19, NCOR1	Protein-coding
TTC26	Protein-coding
TTC34 - ACTRT2	Read-Through
TTC36	Protein-coding
TTLL4	Protein-coding
TUBAP15	Protein-coding
TULP1	Protein-coding
TUSC3	Protein-coding
TWF2	Protein-coding
UBASH3B	Protein-coding

UBE2R2	Protein-coding
UBE2WP1	Protein-coding
UBN1	Protein-coding
UBQLN4	Protein-coding
UBXN7	Protein-coding
UMO2P2	Protein-coding
UNC13D	Protein-coding
UNC5C	Protein-coding
UNC79	Protein-coding
USP3	Protein-coding
VEGFA	Protein-coding
VGLL4	Protein-coding
VPS11	Protein-coding
VPS13C	Protein-coding
WDR72	Protein-coding
WNK1	Protein-coding
WSCD2	Protein-coding
XKR6	Protein-coding
XXYLT1	Protein-coding
YWHAZ	Protein-coding
ZBTB20	Protein-coding
ZBTB7A	Protein-coding
ZBTB7B	Protein-coding
ZBTB7C	Protein-coding
ZC3H4	Protein-coding
ZCCHC4	Protein-coding
ZCCHC7	Protein-coding
ZCCHC8	Protein-coding
ZDBF2 - ACER2P1	Read-Through
ZFHX3	Protein-coding
ZFP64	Protein-coding
ZKSCAN5	Protein-coding
ZMIZ2	Protein-coding
ZMYM2	Protein-coding
ZNF131	Protein-coding
ZNF536	Protein-coding
ZPR1	Protein-coding
ZZZ3	Protein-coding
