# Supplementary figures and images for: A Complex Network of Obesity-Risk Genes Revealed by Systematic Bioinformatics and Single-Cell Transcriptomic Analyses
Source: J Obes. 2025 Mar 31;2025:7821115. doi: 10.1155/jobe/7821115 (PMC11976034; doi:10.1155/jobe/7821115)

## Data-mining of GWAS datasets of obesity traits (BMI)

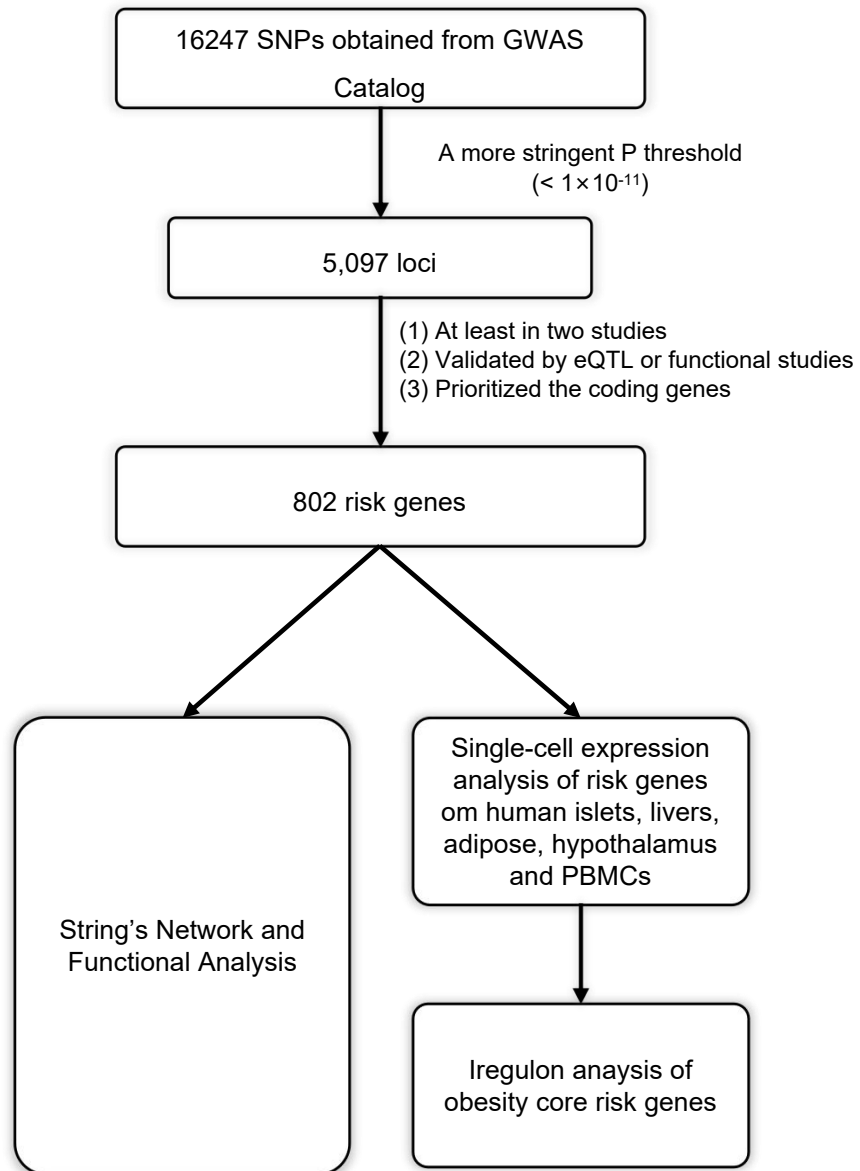

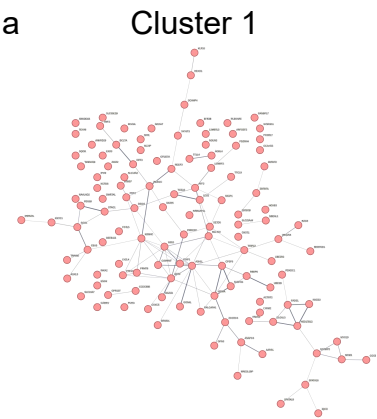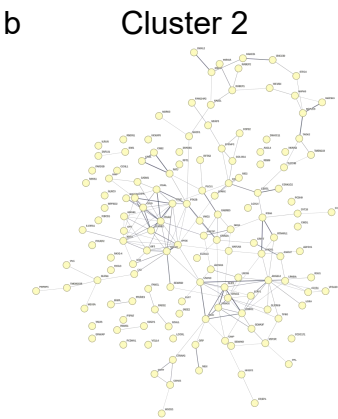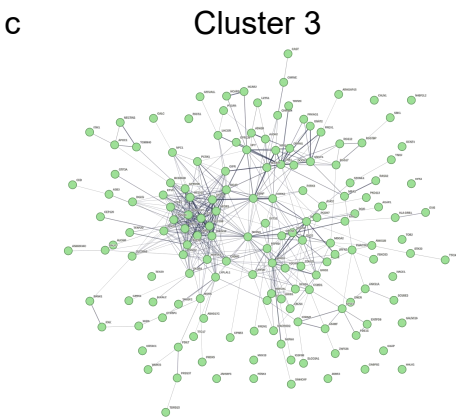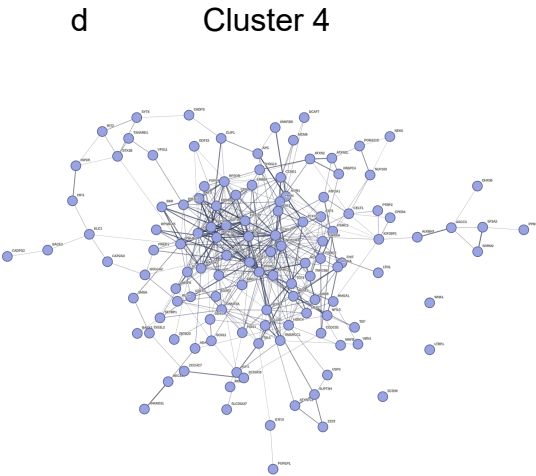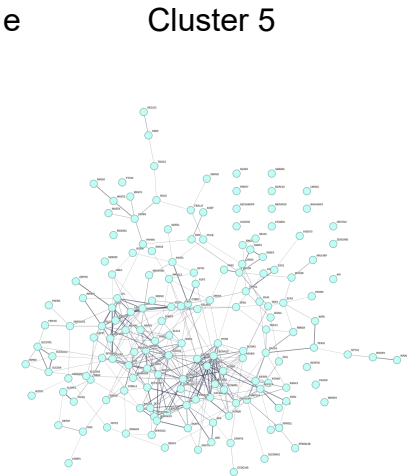

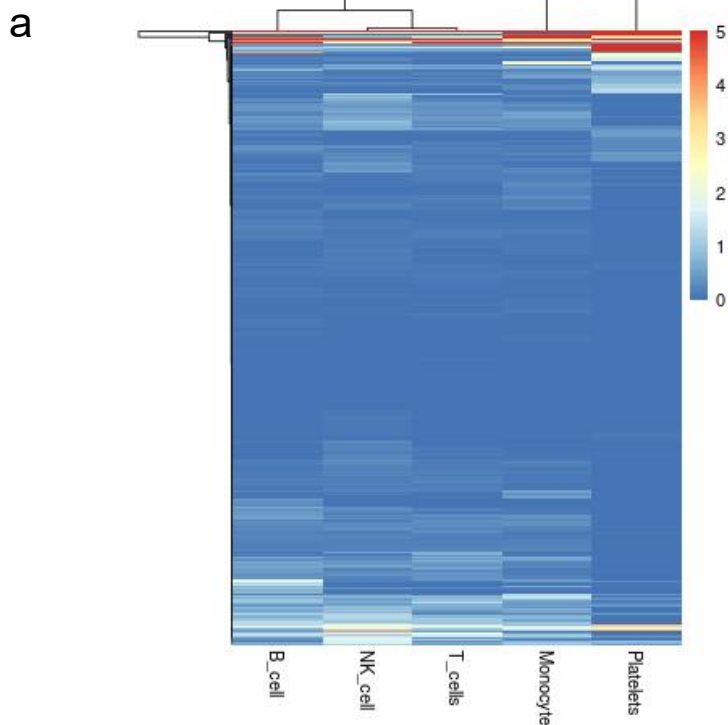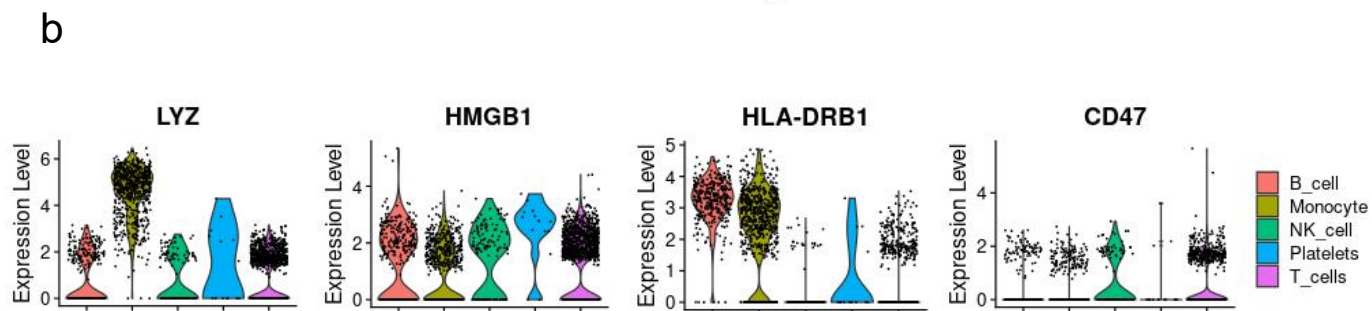

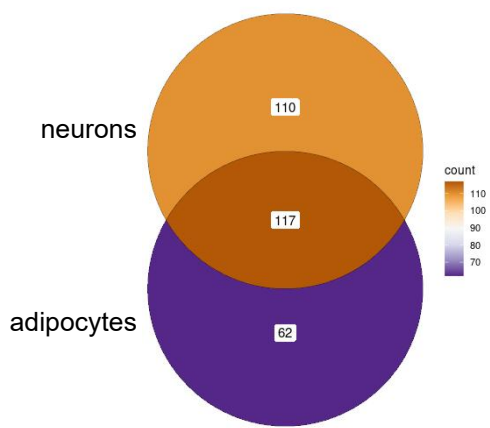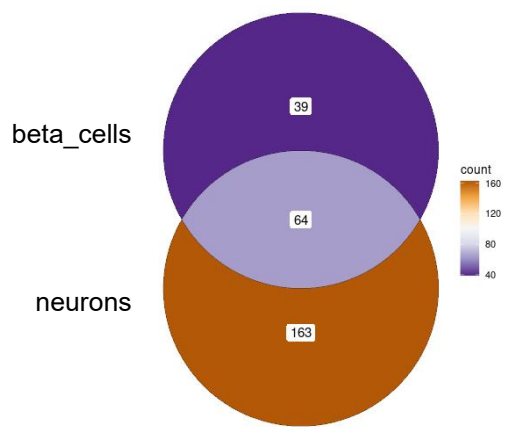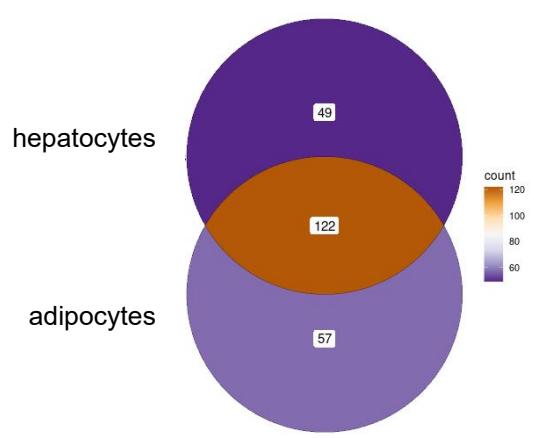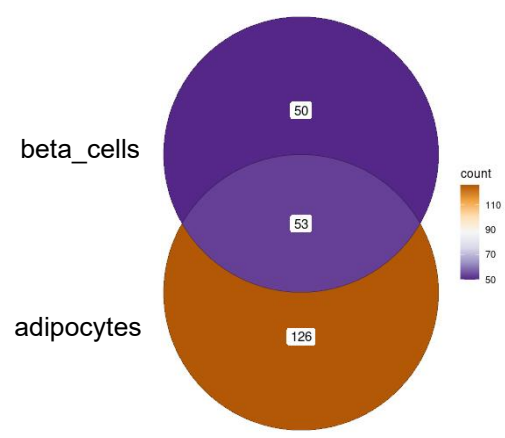

Supplement: Supporting Information — Additional supporting information can be found online in the Supporting Information section. [file 7821115.f1.zip › JO-SUPPLEMENTRY.pdf]
